# Comparative safety of different sodium-glucose transporter 2 inhibitors in patients with type 2 diabetes: a systematic review and network meta-analysis of randomized controlled trials

**DOI:** 10.3389/fendo.2023.1238399

**Published:** 2023-08-28

**Authors:** Chun Xing Li, Li Yan Liu, Chen Xiao Zhang, Xu Hua Geng, Si Meng Gu, Yu Qiao Wang, Hua Liu, Qing Xie, Shuo Liang

**Affiliations:** ^1^Department of Pharmacy, Aerospace Center Hospital, Beijing, China; ^2^Department of Pharmacy, Shengjing Hospital of China Medical University, Shenyang, China; ^3^Department of Gastroenterology, Aerospace Center Hospital, Peking University Aerospace School of Clinical Medicine, Beijing, China

**Keywords:** sodium-glucose transporter 2 inhibitors, reproductive tract infections, pollakiuria, hypovolemia, network meta-analysis

## Abstract

**Backgrounds:**

The safety of different sodium-glucose transporter 2 (SGLT-2) inhibitors remains uncertain due to the lack of head-to-head comparisons.

**Methods:**

This network meta-analysis (NMA) was performed to compare the safety of nine SGLT-2 inhibitors in patients with type 2 diabetes (T2DM). PubMed, Embase, Cochrane Central Register of Controlled Trials and ClinicalTrials.gov were searched for studies published in English before August 30, 2022. Published and unpublished randomized controlled trials (RCTs) comparing the safety of individual SGLT-2 inhibitors in patients with T2DM were included. A Bayesian NMA with random effects model was applied. Subgroup and sensitivity analyses were performed. The quality of the evidence was evaluated using the Confidence in Network Meta-Analysis framework.

**Results:**

Nine SGLT-2 inhibitors were evaluated in 113 RCTs (12 registries) involving 105,293 adult patients. Reproductive tract infections (RTIs) were reported in 1,967 (4.51%) and 276 (1.01%) patients in the SGLT-2 inhibitor and placebo groups, respectively. Furthermore, pollakiuria was reported in 233 (2.66%) and 45 (0.84%) patients, respectively. Compared to placebo, a significantly higher risk of RTIs was observed with canagliflozin, ertugliflozin, empagliflozin, remogliflozin, dapagliflozin, and sotagliflozin, but not with luseogliflozin and ipragliflozin, regardless of gender. An increased risk of pollakiuria was observed with dapagliflozin [odds ratio (OR) 10.40, 95% confidence interval (CI) 1.60-157.94) and empagliflozin (OR 5.81, 95%CI 1.79-32.97). Remogliflozin (OR 6.45, 95%CI 2.18-27.79) and dapagliflozin (OR 1.33, 95%CI 1.10-1.62) were associated with an increased risk of urinary tract infections (UTIs). Instead, the included SGLT-2 inhibitors had a protective effect against acute kidney injury (AKI). No significant differences were found for hypovolemia, renal impairment or failure, fracture, diabetic ketoacidosis (DKA), amputation, and severe hypoglycemia between the SGLT-2 inhibitor and the placebo groups.

**Conclusion:**

In patients with T2DM, dapagliflozin was associated with an increased risk of RTIs, pollakiuria, and UTIs. Empagliflozin increased the risk of RTIs and pollakiuria. Remogliflozin increased the risk of UTIs. None of the SGLT-2 inhibitors showed a significant difference from the placebo for hypovolemia, renal impairment or failure, fracture, DKA, amputation, and severe hypoglycemia. The findings guide the selection of SGLT-2 inhibitors for patients with T2DM based on the patient’s profiles to maximize safety.

**Systematic review registration:**

https://www.crd.york.ac.uk/prospero, identifier CRD42022334644.

## 1 Introduction

Sodium-glucose transporter 2 (SGLT2) inhibitors are a new class of oral anti-diabetic drugs with evidence of improvement in metabolic syndrome and cardiovascular outcomes. SGLT2 inhibitors are recommended as first-line treatment in patients with type 2 diabetes mellitus (T2DM) and heart failure, especially heart failure with decreased ejection fraction (1). SGLT2 inhibitors may be associated with an increased risk of reproductive tract infections (RTIs), pollakiuria, hypovolemia, urinary tract infections (UTIs), and other adverse effects. However, the evidence differed between trials (2–6), all of which compared a single SGLT2 inhibitor with a placebo. Head-to-head comparisons were published only in three studies and one registry, which found that empagliflozin had a lower risk of urinary and genital infection than dapagliflozin (7, 8) A network meta-analysis (NMA) conducted in 2016 showed that dapagliflozin (10 mg) was associated with an increased risk of UTI compared to empagliflozin (25 mg) (6) Furthermore, concerns have been raised about the potential safety of individual SGLT2 inhibitors.

Although three NMAs analyzed the safety differences between SGLT2 inhibitors, the number of drugs and related adverse reactions included was limited (6, 9, 10). Whether the class safety profiles can represent individual SGLT2 inhibitors remains to be clarified. Therefore, this systematic review and NMA of randomized controlled trials (RCTs) aimed to evaluate the relative safety of nine SGLT2 inhibitors regarding RTIs, pollakiuria, hypovolemia, renal impairment/failure, acute kidney injury (AKI), UTIs, fracture, diabetic ketoacidosis (DKA), amputation, and severe hypoglycemia in patients with T2DM.

## 2 Materials and methods

This NMA followed the Preferred Reporting Items for Systematic Reviews and Meta-Analyses (PRISMA) extension statement (11). The protocol was registered in the International Prospective Register of Systematic Review (PROSPERO, registration number CRD42022334644).

### 2.1 Data sources, search strategies, and study selection

We searched PubMed, Cochrane Central Register of Controlled Trials, Embase, and ClinicalTrials.gov for RCTs comparing SGLT2 inhibitors to placebo or other SGLT2 inhibitors in T2DM patients from the inception to August 30, 2022. The search consisted of three domains: intervention (SGLT2 inhibitor class or individual drugs), adverse events of particular interest, and RCTs. Details of the search strategy are provided in **Appendix 2**.

### 2.2 Selection of studies

The literature screening used PICOS (participants, intervention, comparison, outcomes and characteristics, and study design). RCTs were included if they met the following criteria: 1) T2DM patients ≥ 18 years old; 2) patients who received SGLT2 inhibitors (canagliflozin, dapagliflozin, empagliflozin, ertugliflozin, tofogliflozin, luseogliflozin, ipragliflozin, remogliflozin, or sotagliflozin) with doses equal or greater than the approved doses for at least 12 weeks; 3) the comparator was placebos or any of the nine SGLT2 inhibitors; and 4) studies evaluated safety outcomes. Only RCTs with a sample size in the single group (sum of all dose groups) greater than 50 were included.

Primary outcomes were the risks of RTIs and pollakiuria. Secondary outcomes were the risks of hypovolemia, renal impairment and failure, AKI, UTI, fracture, DKA, amputation, and severe hypoglycemia. Two authors (LY Liu and CX Zhang) independently performed the literature search and study selection using Endnote 18.0. Disagreements were resolved by a third author (CX Li).

### 2.3 Data extraction

Two authors (LY Liu and CX Zhang) independently completed the data extraction using a standardized form and verified by the third author. The following data were extracted: study characteristics (first author, year of publication, countries/regions, follow-up time, details of the interventions, number of patients, and outcomes) and participants’ characteristics [mean or median age, percentage of women, baseline body mass index (BMI), median hemoglobin A1c level, and stage of renal impairment].

### 2.4 Risk of bias and certainty of evidence assessment

Two authors independently assessed the risk of bias in eligible studies using the revised Cochrane Collaboration Risk of Bias 2 (RoB2) tool for RCTs (12). Any discrepancies were resolved by consensus with a third author. We assessed all endpoints from the five domains, and the overall risk of bias was rated as “low risk,” “some concerns,” and “high risk.”

We used the Confidence in Network Meta-Analysis (CINeMA) framework and the Web application to evaluate confidence in the NMA results. CINeMA offers a more comprehensive approach to assessing the quality of evidence in NMA (13). CINeMA considers six domains that affect the confidence level in the NMA results: within-study bias, reporting bias, indirectness, imprecision, heterogeneity, and incoherence. The level of concern for each relative treatment effect of NMA gives rise to “no concerns,” “some concerns,” or “major concerns” in each of the six domains. Then, the judgments in all domains are summarized into a single confidence rating (“high,” “moderate,” “low,” or “very low”). The percentage contribution matrix and the weighted average risk of bias were applied to assess the within-study bias and the indirectness of each comparison.

### 2.5 Data synthesis

We performed Bayesian random-effects NMA of all outcomes. Dichotomous outcomes were calculated as odds ratios (OR) and 95% confidence intervals (CI). Model convergence was monitored and visualized using trace, density, and Brooks-Gelman-Rubin diagnosis plots. Good model convergence was when the potential scale reduction factor (PSRF), the median value of the shrink factor, and the 97.5% value simultaneously approached 1. The network graphs scaled by the number of patient studies by each treatment node and the risk of bias were presented graphically.

The *I^2^* estimates and their 95% CIs were used to assess heterogeneity: low (0-29%), moderate (30-59%), substantial (60-89%), and high (>89%). Subgroup analyses were used to address heterogeneity. For inconsistency, we looked at the results of node splitting. The consistency was assessed by considering direct and indirect evidence separately with node splitting. Treatment rankings were evaluated using the surface under the cumulative ranking curve (SUCRA). SUCRA values range from 0 to 100%. The higher the SUCRA value, and the closer to 100%, the higher the probability that a therapy is in the top rank or one of the top ranks.

All analyses were performed using four Markov chains (50,000 iterations after a burn-in of 10,000 and a thinning of 10). For further verification, the results were reproduced by implementing the R software (version 4.0.3) with the gemtc package (version 0.8-8) and the JAGS software (version 4.3.0). The RTIs were calculated separately for males and females. Studies focusing on T2DM with chronic kidney disease (CKD) and studies that reported extended follow-ups (≥48 weeks) were not included in the overall analysis. They were only included in the subgroup analysis. Trials with zero events in both groups were omitted from the NMA.

### 2.6 Sensitivity and subgroup analysis

The dose-specific network model was conducted. Subgroup analyses were performed according to renal function (estimated glomerular filtration rate (eGFR) < 90 mL/min/m^2^), study countries/regions (Asia, Japan, or China), types of SGLT2 inhibitor treatment (an add-on to metformin-based therapy or monotherapy in drug-naïve patients), and duration of follow-up.

## 3 Results

The initial screening identified 113 RCTs (n=105,293) for inclusion in the systematic review (2–5, 7, 8, 14–119) (**Appendix 1**). Twelve trials were published in registries (107, 108, 110–119). After reviewing data collection, 21 RCTs were excluded from NMA: 13 focused on T2DM and CKD (96–106, 108, 120), and 11 reported extended follow-ups (21, 28, 69, 89–95, 121). Among the 11 studies, 8 had only extended follow-up data, and 3 had short (24-28 weeks) and extended follow-up results (only extended follow-up outcomes were excluded).

### 3.1 Characteristics of included studies

**Appendix 4** shows the characteristics of the included studies, including 78 (69.0%) multinational studies, 25 (22.1%) Japanese studies, and 6 (5.3%) conducted in other Asia regions. The median number of patients was 133 [33-8,582, interquartile range (IQR) 140], and the median age was 57.4 years (48.7-70.5). Twenty-three studies (20.35%) enrolled 50% or more women. The patients were primarily overweight, with a median baseline BMI of 30.7 (23.36-36.04). Most patients had a baseline median hemoglobin A1c of 8.1% (6.87-10.10). Among these studies, 90 (79.7%) studies were patients with T2DM alone, 13 (11.5%) were patients with T2DM with CKD, and 10 (8.9%) were patients with T2DM with cardiovascular disease and/or hypertension. Seventy-five studies (66.4%) (n=53,658) reported the average time since diabetes diagnosis, with a median of 7.2 years (0.25-20.70, IQR 5.67).

SGLT-2 inhibitors were used as monotherapy in drug-naive patients (20 trials; 5,714 patients) or as an addition to metformin-based therapy (25 trials; 9,957 patients). A total of 109 (96.5%) of the studies were placebo-controlled, and 4 (3.5%) had an active SGLT-2 inhibitor as a comparator. The types of intervention were dapagliflozin (5-50 mg/d, 34 studies), empagliflozin (10-50 mg/d, 26 studies), ipragliflozin (50-300 mg/d, 18 studies), canagliflozin (100-600 mg/d, 17 studies), sotagliflozin (200-400 mg/d, 11 studies), ertugliflozin (5-25 mg/d, 8 studies), luseogliflozin (2.5-10 mg/d, 8 studies), tofogliflozin (20-40 mg/d, 3 studies), and remogliflozin (200-2000 mg/d, 3 studies). The median duration of the intervention was 24 weeks (12-338). Eleven studies had a short follow-up of 24 to 28 weeks and an extended follow-up of 48 to 102 weeks.

### 3.2 Assessment of risk of bias

The quality of the studies varied (**Appendix 5**). No studies had high risks of bias for the randomization process, deviations from the intended intervention, the measurement of the outcome, and the selection of the reported result. Two studies (1.9%) had a high risk of missing outcome data. Overall, 77 studies (73.3%) had a low risk of bias (overall bias score of 1), 26 (24.8%) had some concerns (overall bias score of 2), and 2 (1.9%) had a high risk of bias (overall bias score of 1) (**Appendix 5.1**). The risk of bias for each outcome is shown in the RoB chart (**Appendix 5.2**).

### 3.3 Primary outcome: reproductive tract infections

Seventy-nine RCTs (n=70,850) (2–5, 7, 8, 14–24, 26–32, 34–76, 78–80, 83, 85–88, 112, 115, 117, 118) (four published in registries) reported 2, 243 (3.2%) cases of RTIs: 1,967 (4.5%) in the SGLT-2 inhibitor group and 276 (1.0%) in the placebo group. RTIs were reported in patients treated with canagliflozin (552, 6.7%), ertugliflozin (385, 5.5%), empagliflozin (536, 5.1%), sotagliflozin (21, 3.3%), remogliflozin (23, 2.9%), dapagliflozin (412, 2.9%), ipragliflozin (26, 2.2%), tofogliflozin (5, 1.3%), and luseogliflozin (7, 1.3%). Details are shown in **Table 1**.

**Table 1:** Summary table of the results for each outcome.

Outcomes	Included SGLT-2is	Included study	Participants n (SGLT-2is/Placebo)	Events n(%) (SGLT-2is/Placebo)	*I^2%^(pairwise and consistency)*	PSRF	SUCRA
Reproductive tract infections (2–5, 7, 8, 14–24, 26–32, 34–76, 78–80, 83, 85–88, 112, 115, 117, 118)	SGLT-2is	79(4 register),76 was included in NMA	43631/27219	1967(4.51)/276(1.01)	35.60/36.41	1.01	placebo 0.03
	canagliflozin	11(2 register)	8226/5585	552(6.71)/105(1.88)			0.41
	dapagliflozin	26	14377/12155	412(2.87)/51(0.42)			0.81
	empagliflozin	19(1 register)	10475/4701	536(5.12)/59(1.26)			0.59
	ertugliflozin	6	7026/3481	385(5.48)/52(1.49)			0.54
	ipragliflozin	8	1169/538	26(2.22)/5(0.93)			0.32
	luseogliflozin	4	528/264	7(1.33)/2(0.76)			0.33
	remogliflozin	3	798/84	23(2.88)/0(0.00)			0.70
	sotagliflozin	3(2 register)	645/219	21(3.26)/2(0.91)			0.76
	tofogliflozin#	3, 0 was included in NMA	387/192	5(1.29)/0(0.00)			NA
Pollakiurias (4, 7, 8, 14, 17, 18, 20, 24, 26, 27, 32, 56, 59, 65, 66, 69, 71, 75, 77, 79–81, 110, 117, 119)	SGLT-2is	25(3 register), 24 was included in NMA	8773/5344	233(2.66)/45(0.84)	41.40/45.56	1.00	placebo 0.09
	canagliflozin	5(1 register)	4105/3468	42(1.02)/15(0.43)			0.33
	dapagliflozin	3(1 register)	327/50	22((6.73)/0(0.00)			0.79
	empagliflozin	8	2443/715	95(3.89)/11(1.54)			0.65
	ertugliflozin	2	720/362	12(1.67)/1(0.28)			0.76
	ipragliflozin	5	559/405	33(5.90)/13(3.21)			0.29
	luseogliflozin	2	238/153	17(7.14)/4(2.61)			0.43
	sotagliflozin	1(1 register)	250/125	10(4.00)/1(0.80)			0.67
	tofogliflozin#	1, 0 was included in NMA	131/66	2((1.53)/0(0.00)			NA
Hypovolemia (2, 3, 8, 15–17, 20–24, 26–29, 31, 36, 37, 40, 43, 45, 47, 50, 51, 53–55, 61, 63, 65–67, 69–72, 80, 82, 84, 117, 118)	SGLT-2is	42(2 register), 40 was included in NMA	34764/23604	824(2.37)/477(2.02)	45.95/45.49	1.00	placebo 0.21
	ertugliflozin	5	6861/3427	246(3.59)/115(3.36)			0.17
	empagliflozin#	9, 8 was included in NMA	7467/3288	255(3.42)/119(3.62)			0.33
	dapagliflozin	11	11444/10932	254(2.22)/226(2.07)			0.55
	ipragliflozin#	3, 2 was included in NMA	322/225	7(2.17)/3(1.33)			0.57
	canagliflozin	9 (2 register)	7965/5421	35(0.44)/9(0.17)			0.67
	tofogliflozin	2	256/126	13(5.08)/3(2.38)			0.68
	luseogliflozin	3	449/185	14(3.12)/2(1.08)			0.81
Renal impairment or failure (14, 18, 36, 39, 40, 42–53, 55, 111, 117, 118)	SGLT-2is	21(3 register), 19 was included in NMA	10468/7855	177(1.69)/97(1.23)	18.91/19.00	1.00	placebo 0.36
	canagliflozin	2(2 register)	5790/4344	14(0.24)/10(0.23)			0.37
	dapagliflozin	16	4209/3107	157(3.73)/79(2.54)			0.84
	luseogliflozin	1	79/79	6(7.60)/6(7.60)			0.43
	tofogliflozin#	1, 0 was included in NMA	131/66	0(0.00)/1(1.51)			NA
	sotagliflozin#	1(1 register), 0 was included in NMA	259/259	0(0.00)/1(0.39)			NA
Acute kidney injury (23, 45, 54, 55, 67, 111, 114, 117, 118)	SGLT-2is	9(4 register) was included in NMA	25748/19195	506(1.97)/420(2.19)	76.08/70.65	1.00	placebo 0.60
	empagliflozin	1	4687/2333	246(5.25)/155(6.64)			0.45
	sotaglifozin	2(2 register)	513/512	1(0.19)/0(0.00)			0.52
	canagliflozin	2(2 register)	5790/4344	30(0.52)/28(0.64)			0.37
	ertugliflozin	1	5493/2745	101(1.84)/60(2.19)			0.50
	dapagliflozin	3	9265/9261	128(1.38)/176(1.90)			0.55
Urinary tract infections (2–5, 7, 8, 14–21, 23, 24, 26–76, 78–88, 107, 110–114, 116–119)	SGLT-2is	89 (10 register), 85 was included in NMA	46142/28438	3133(6.79)/1457(5.12)	2.78/4.87	1.02	placebo 0.25
	Tofogliflozin	3	387/192	4(1.03)/3(1.56)			0.28
	empagliflozin	20(2 register)	11351/4808	1287(11.34)/582(12.10)			0.33
	sotagliflozin	7(6 register)	1924/991	102(5.30)/54(5.45)			0.53
	canagliflozin	12(2 register)	8297/5654	425(5.12)/194(3.43)			0.47
	ertugliflozin	7	7174/3626	723(10.08)/312(8.60)			0.48
	ipragliflozin	11	1403/759	50(3.56)/22(2.90)			0.42
	dapagliflozin	27(1 register)	14280/12060	494(3.46)/290(2.40)			0.75
	luseogliflozin#	4, 0 was included in NMA	528/264	4(0.76)/0(0.00)			NA
	remogliflozin	3	798/84	44(5.51)/0(0.00)			1.00
Fracture (23, 32, 33, 39, 43, 46, 49, 52, 54, 62, 67, 69, 72, 107, 110, 113–115, 117, 118)	SGLT-2is	20(7 register) was included in NMA	28314/20316	957(3.38)/670(3.30)	60.58/60.77	1.00	placebo 0.48
	empagliflozin	3	5202/2591	186(3.58)/96(3.71)			0.43
	dapagliflozin	6	9919/9556	469(4.72)/451(4.72)			0.53
	sotagliflozin	5(5 register)	1655/831	10(0.60)/6(0.72)			0.54
	ertugliflozin	1	5493/2745	201(3.66)/98(3.57)			0.50
	canagliflozin	5(2 register)	6045/4593	91(1.51)/19(0.41)			0.53
Diabetic ketoacidosis (2, 23, 54, 67, 113, 114, 118)	SGLT-2is	7(3 register) was included in NMA	22295/15541	73(0.33)/18(0.12)	69.71/69.68	1.00	placebo 0.21
	sotagliflozin	2(2 register)	539/397	1(0.19)/1(0.25)			0.26
	empagliflozin	1	4687/2333	4(0.09)/1(0.04)			0.50
	dapagliflozin	1	8574/8569	27(0.31)/12(0.14)			0.45
	canagliflozin	1(1 register)	2886/1441	7(0.24)/1(0.07)			0.63
	ertugliflozin	1	5493/2745	19(0.35)/2(0.07)			0.66
	tofogliflozin	1	116/56	15(12.93)/1(1.79)			0.79
Amputation (23, 51, 54, 115)	SGLT-2is	4(1 register) was included in NMA	14768/11697	238(1.61)/160(1.37)	46.66/50.77	1.00	placebo 0.38
	sotagliflozin	1(1 register)	476/159	4(0.84)/1(0.63)			0.65
	dapagliflozin	2	8799/8793	123(1.40)/114(1.30)			0.38
	ertugliflozin	1	5493/2745	111(2.02)/45(1.64)			0.58
Severe hypoglycemia (7, 15, 20, 21, 23, 26, 28, 31, 40, 54, 63, 64, 67, 68, 114)	SGLT-2is	15(1 register), 13 was included in NMA	22645/15579	434(1.92)/292(1.87)	64.86/50.35	1.00	placebo 0.56
	empagliflozin	5	5792/2820	69(1.19)/36(1.28)			0.61
	sotagliflozin#	1(1 register), 0 was included in NMA	254/253	1(0.39)/0(0.00)			NA
	canagliflozin	3	1241/619	9(0.73)/8(1.29)			0.35
	ertugliflozin	3	6109/3051	286(4.68)/163(5.34)			0.46
	dapagliflozin	3	9109/8766	67(0.74)/85(0.99)			0.51
	tofogliflozin#	1, 0 was included in NMA	140/70	2(1.43)/0(0.00)			NA

SGLT-2is, Sodium-glucose transporter 2 inhibitors; eGFR, estimated glomerular filtration rate; unit is mL/min/1.73 m^2^, PSRF, The potential scale reduction factor; SUCRA, surface under the cumulative ranking scores; NMA, Network meta-analysis, #: Due to the wide confidence interval, studies related to this drug were not included in the network meta-analysis. NA, Not Applicable.

**Figure 1:**
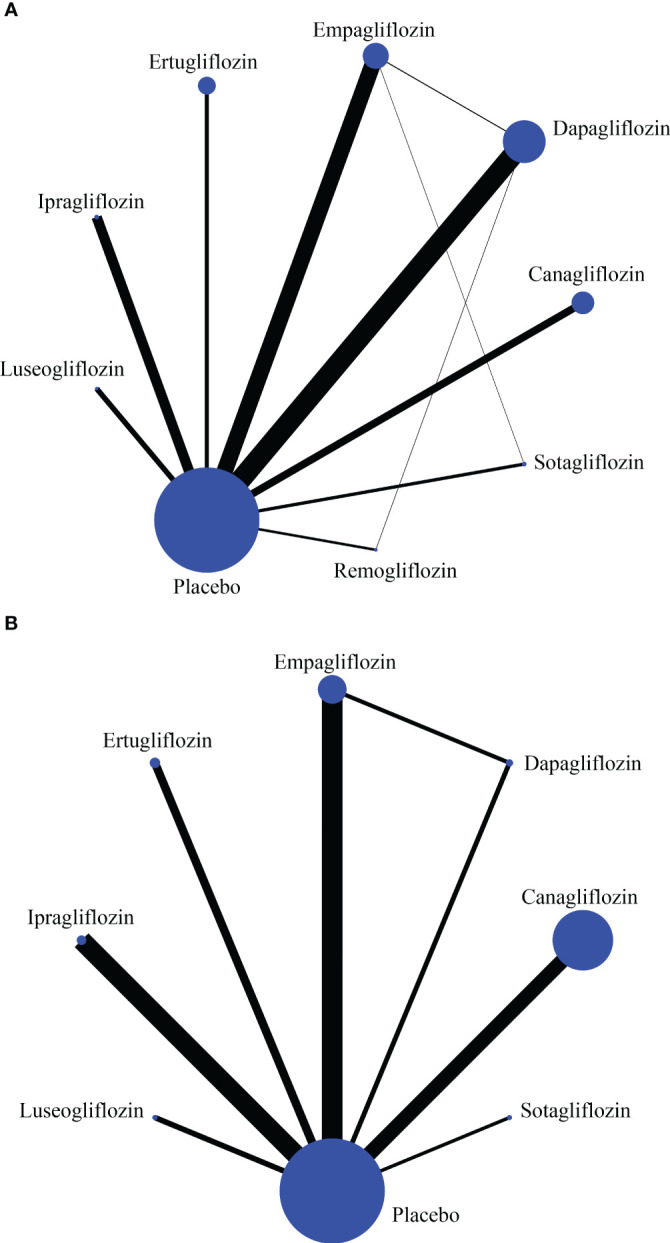
The network plot of reproductive tract infections **(A)** and pollakiuria **(B)**. Each circle indicates a treatment node. Lines connecting 2 nodes represent direct comparisons between 2 treatments. The size of the nodes is proportional to the number of trials evaluating each treatment. The thickness of the lines is proportional to the number of trials directly comparing the 2 connected treatments.

The pooled OR of tofogliflozin had a wide 95% CI, which reduced the confidence of the results. Therefore, related studies were excluded from the NMA (2, 14, 15). Head-to-head comparative studies (sotagliflozin and empagliflozin, empagliflozin and dapagliflozin, remogliflozin and dapagliflozin) are shown in the network plot (**Figure 1**), which contributed 6.3%, 2.5%, and 8.1% to the entire network, respectively (**Appendix 12.1**). The trace and density plots showed good model convergence (**Appendix 6.1**). The PSRF was 1.01 on the Brooks-Gelman-Rubin diagnosis plot (**Appendix 6.1**).

**Figure 2:**
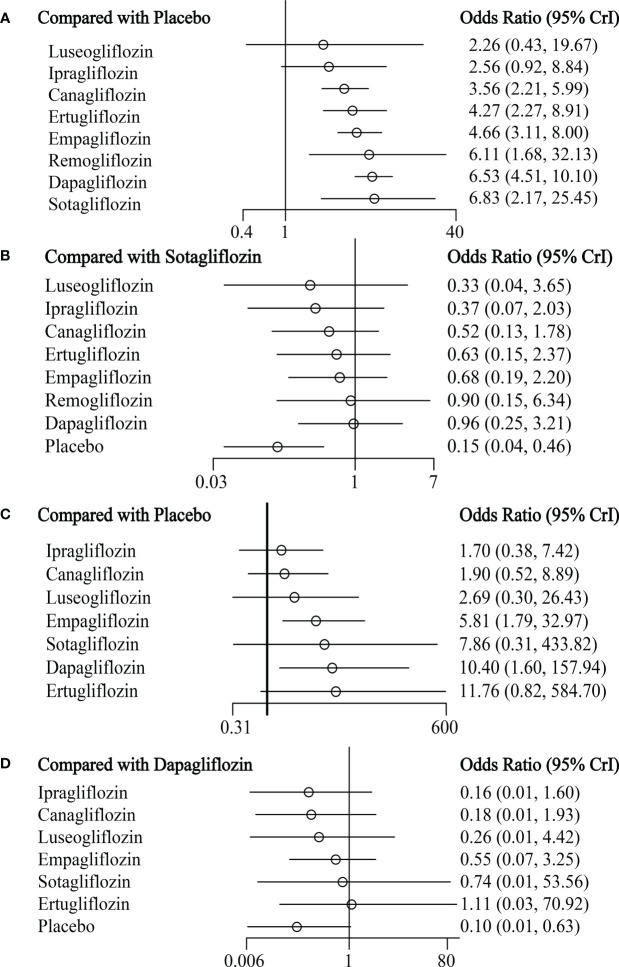
The forest plot of reproductive tract infections **(A, B)** and pollakiuria **(C, D)**.

Canagliflozin, ertugliflozin, empagliflozin, remogliflozin, dapagliflozin, and sotaglifozin were associated with a significant increase in RTIs compared to placebo. In contrast, luseogliflozin and ipragliflozin were unrelated to the risk of RTIs (**Figure 2A**; **Table 2**). The certainty of the evidence was low to high (**Appendix 17.1**). Compared to sotagliflozin or luseogliflozin, the risk of RTIs was not significantly different between individual SGLT-2 inhibitors (**Figure 2B**; **Table 2**).

**Table 2:** Network meta-analysis results for reproductive tract infections (left lower half) and pollakiuria (right upper half).

**luseogliflozin**	0.78 (0.02, 21.54)	0.49 (0, 72.98)	NA	0.24 (0, 10.70)	NA	NA	NA	2.76 (0.19, 45.72)
0.87 (0.11, 9.36)	**ipragliflozin**	0.63 (0, 79.71)	NA	0.29 (0, 10.41)	NA	NA	NA	3.48 (0.53, 34.27)
0.63 (0.11, 5.80)	0.72 (0.23, 2.71)	**canagliflozin**	NA	0.46 (0, 96.81)	NA	NA	NA	5.49 (0.09, 599.62)
0.52 (0.08, 5.13)	0.60 (0.17, 2.38)	0.84 (0.35, 1.87)	**ertugliflozin**	NA	NA	NA	NA	NA
0.48 (0.08, 4.31)	0.55 (0.17, 2.02)	0.77 (0.37, 1.44)	0.92 (0.39, 1.99)	**empagliflozin**	NA	NA	NA	11.88 (0.73, 556.48)
0.37 (0.03, 4.61)	0.42 (0.06, 2.51)	0.58 (0.10, 2.33)	0.70 (0.12, 3.10)	0.77 (0.15, 3.05)	**remogliflozin**	NA	NA	NA
0.35 (0.06, 3.14)	0.39 (0.13, 1.41)	0.54 (0.29, 1.02)	0.65 (0.30, 1.46)	0.71 (0.42, 1.29)	0.94 (0.26, 4.69)	**dapagliflozin**	NA	NA
0.33 (0.04, 3.65)	0.37 (0.07, 2.03)	0.52 (0.13, 1.78)	0.63 (0.15, 2.37)	0.68 (0.19, 2.20)	0.90 (0.15, 6.34)	0.96 (0.25, 3.21)	**sotagliflozin**	NA
2.26 (0.43, 19.67)	2.56 (0.92, 8.84)	***3.56 (2.21, 5.99)***	***4.27 (2.27, 8.91)***	***4.66 (3.11, 8.00)***	***6.11 (1.68, 32.13)***	***6.53 (4.51, 10.10)***	***6.83 (2.17, 25.45)***	**placebo**

Data are odds ratio with a 95% credible interval. The figure should be read from left to right: for comparisons of the reproductive tract infections (left lower half), the odds ratio <1 favors the column defining treatment, whereas for pollakiuria (right upper half), the odds ratio <1 favors the row describing the treatment. Reciprocals should be taken to obtain odds ratios for comparisons in the opposite direction. NA, Not Applicable.

**Figure 3:**
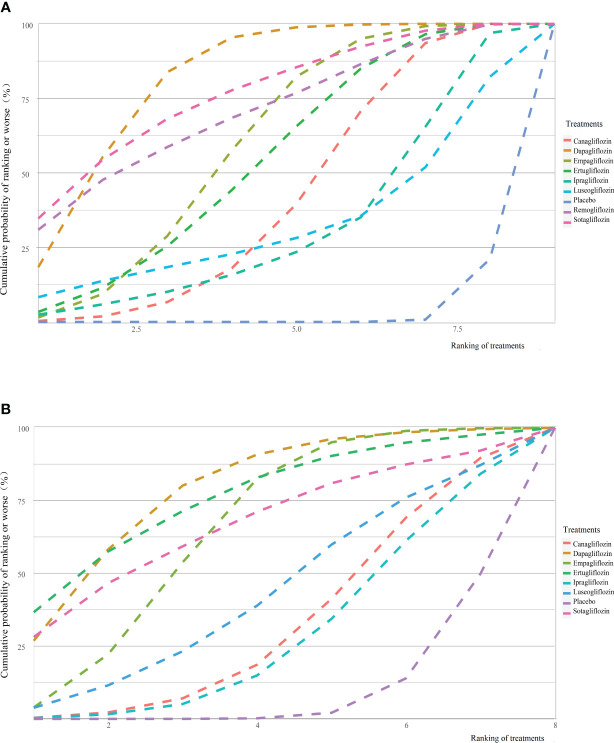
Cumulative ranking curves for reproductive tract infections **(A)** and pollakiuria **(B)**. Graphs show the cumulative probability of each intervention ranking, from worst (rank 1) to best (rank 9 or 8 depending on the number of treatments) for each outcome. A rank indicates the probability that an intervention is worst, second worst etc. For example, dapagliflozin probably ranked worst for increasing the risk of reproductive tract infections.

There was moderate heterogeneity between the studies (*I^2^* pairwise and consistency was 35.60% and 36.41%, and the *P-*value of inconsistency was > 0.1) (**Appendix 11.1**). The SUCRA value of dapagliflozin, sotagliflozin, remogliflozin, empagliflozin, ertugliflozin, canagliflozin, luseogliflozin, ipragliflozin, and placebo was 0.81, 0.76, 0.70, 0.59, 0.54, 0.41, 0.32, 0.31, and 0.03, respectively. Dapagliflozin was ranked highest for the increased risk of RTIs (**Figure 3A**). Comparison-adjusted funnel plots did not suggest the presence of small study bias (**Appendix 14.1**).

RTIs were reported in 519 (4.1%) of the men in the SGLT-2 inhibitor group compared to 61 (1.0%) in the placebo group. In contrast, 635 (8.4%) of the women reported RTIs in the SGLT-2 inhibitor group compared to 85 (2.22%) in the placebo group. Women had the same risk of RTIs between ipragliflozin, ertugliflozin, canagliflozin, empagliflozin, and dapagliflozin. However, higher ORs in RTIs were observed in men compared to women (Appendices 7.1-7.2 and 8.1-8.2).

### 3.4 Primary outcome: pollakiuria

Twenty-five RCTs (n=14,117) (4, 7, 8, 14, 17, 18, 20, 24, 26, 27, 32, 56, 59, 65, 66, 69, 71, 75, 77, 79–81, 110, 117, 119) (three published in the registries) reported 278 (1.9%) cases of pollakiuria: 233 (2.7%) in the SGLT-2 inhibitor group and 45 (0.8%) in the placebo group. Among the 233 cases in the SGLT-2 inhibitor group, the number of pollakiuria cases was luseogliflozin (17, 7.1%), dapagliflozin (22, 6.7%), ipragliflozin (33, 5.9%), sotagliflozin (10, 4.0%), empagliflozin (95, 3.9%), ertugliflozin (12, 1.7%), tofogliflozin (2, 1.5%), and canagliflozin (42, 1.0%) (**Table 1**).

The 95% CI of the tofogliflozin combined OR was wide, so the related study (14) was excluded from the NMA. Finally, 24 RCTs were included. Head-to-head comparative studies between empagliflozin and dapagliflozin were found in the network plot (**Figure 1**). The direct comparison contributed 88.5% and 12.4% to the mixed estimates and the entire network, respectively (**Appendix 12.2**).

The trace and density plots showed good model convergence, and the PSRF was 1.00 in the Brooks-Gelman-Rubin diagnosis plot (**Appendix 6.2**). Dapagliflozin (OR 10.40, 95%CI 1.60-157.94) and empagliflozin (OR 5.81, 95%CI 1.79-32.97) increased the risk of pollakiuria compared to placebo (**Figure 2C**; **Table 2**). The certainty of the evidence was low to high (**Appendix 17.2**). Canagliflozin, ertugliflozin, sotagliflozin, luseogliflozin, and ipragliflozin were not associated with the risk of pollakiuria (**Figure 2C**; **Table 2**). Compared to dapagliflozin, other SGLT-2 inhibitors had the same risk of pollakiuria **(** **Figure 2D**; **Table 2****)**.

There was moderate heterogeneity between the studies (*I^2^* pairwise and consistency was 41.40% and 45.56%, and the *P-*value of inconsistency was > 0.1) (**Appendix 11.2**). The SUCRA value of dapagliflozin, ertugliflozin, sotagliflozin, empagliflozin, luseogliflozin, canagliflozin, ipragliflozin, and placebo was 0.79, 0.76, 0.67, 0.65, 0.43, 0.33, 0.28, and 0.09, respectively. Dapagliflozin ranked highest for an increased risk of pollakiuria (**Figure 3B**).

### 3.5 Secondary outcomes

The numbers of cases reported for secondary outcomes were: hypovolemia [824 (2.4%) *vs*. 477 (2.0%)], renal impairment or failure [177 (1.7%) *vs*. 97 (1.2%)], AKI [506 (2.0%) *vs*. 420 (2.2%)], UTIs [3,133 (6.8%) *vs*. 1,457 (5.1%)], fracture [957 (3.4%) *vs*. 670 (3.3%)], DKA [73 (0.3%) *vs*. 18 (0.1%)], amputation [238 (1.6%) *vs*. 160 (1.4%)], and severe hypoglycemia [434 (1.9%) *vs*. 292 (1.9%)] in the SGLT-2 inhibitor and placebo groups, respectively. **Appendix 7.3–7.10** shows the network plot. The trace, density, and Brooks-Gelman-Rubin diagnosis plots showed good model convergence (**Appendix 6.3–6.10**). The details are shown in **Table 1**. The contribution graphs are shown in **Appendix 12.3–12.10**.

The SGLT-2 inhibitors included were not associated with the risk of hypovolemia, renal impairment or failure, fracture, DKA, amputation, or severe hypoglycemia compared to the placebo (Appendices 8.3**-**8.10 and 9.3**–**9.10). The certainty of the evidence was very low to low (**Appendix 17.3–17.10**). Furthermore, canagliflozin, dapagliflozin, empagliflozin, ertugliflozin, and sotagliflozin demonstrated a protective effect on AKI, but the difference was not statistically significant compared to placebo(Appendices 8.5 and 9.5).

Remogliflozin (OR 6.45, 95%CI 2.18-27.79) and dapagliflozin (OR 1.33, 95%CI 1.10-1.62) were associated with an increased risk of UTIs compared to placebo. In contrast, empagliflozin, sotagliflozin, canagliflozin, ertugliflozin, ipragliflozin, and tofogliflozin did not show significant differences with placebo. The certainty of the evidence was low to high (**Appendix 17.6**). Remogliflozin (OR 4.86, 95%CI 1.66-20.88) increased the risk of UTIs, while empagliflozin (OR 0.78, 95%CI 0.61-0.99) decreased the risk of UTIs when compared to dapagliflozin (Appendices 8.6 and 9.6).

Tofogliflozin and luseogliflozin ranked the worst for hypovolemia, with a value of SUCRA of 0.68 and 0.81, respectively. Dapagliflozin ranked worst for renal impairment or failure with a SUCRA value of 0.84. Remogliflozin and dapagliflozin ranked worst for UTIs, with a value of SUCRA 1.00 and 0.75, respectively (**Appendix 13.1**).

### 3.6 Subgroup analysis

#### 3.6.1 Subgroup analysis according to doses 

The risk of RTIs increased with increasing doses of dapagliflozin (5 to 50 mg/d) and ertugliflozin (5 to 20 mg/d, except 10 mg/d). The certainty of the evidence was very low to high. In split-dose studies, subgroup analysis of canagliflozin 600 mg/d (24), empagliflozin 50 mg/d (59, 61), ertugliflozin 10 mg/d (19), remogliflozin 250 mg/d (87), and remogliflozin 1000 mg/d (86, 87) was inconsistent with the overall analysis, suggesting that there was no increased risk of RTIs at these drug doses. Canagliflozin 200 mg/d in women (29, 61) and empagliflozin 50 mg/d (61) in men showed opposite results compared to the overall analysis (**Appendix 15.1**).

Subgroup analyses showed that, in split-dose studies, the risks of pollakiuria (except empagliflozin 50 mg/d (59) and ertugliflozin 15 mg/d (4, 20)), UTIs (except high dose dapagliflozin 20 mg/d (5, 35) and 50 mg/d (5), remogliflozin low dose 200 mg/d (86, 88) and 250 mg/d (87)), and fracture were consistent with the overall analyses. The certainty of the evidence was low to high (**Appendix 15.1**).

Subgroup analyses showed that the risks of hypovolemia (except canagliflozin 300 mg/d), AKI, and severe hypoglycemia were consistent with the overall analyses. The certainty of the evidence was low. Low- to moderate-quality evidence showed the same renal impairment or failure results. Very low to low-quality evidence showed similar results regarding DKA and amputation.

#### 3.6.2 Subgroup analysis according to different regions 

Twenty-five RCTs (n=7,162) reported 113 (2.2%) and 11(0.5%) cases of RTIs in Asia treated with SGLT-2 inhibitors and placebo, respectively (2, 3, 7, 8, 15–18, 22, 29, 31, 32, 41, 42, 49, 52, 53, 61, 69, 71, 76, 78–80, 88). NMA with 23 RCTs suggested empagliflozin and dapagliflozin were associated with an increased risk of RTIs, but not for luseogliflozin, ipragliflozin, canagliflozin, ertugliflozin, and remogliflozin. Similar results were obtained from NMA with 17 RCTs conducted in Japan (2, 3, 15–18, 29, 32, 41, 49, 61, 69, 71, 76, 78–80). Two RCTs from China (22, 42) suggested that dapagliflozin but not ertugliflozin was associated with an increased risk of RTIs (**Appendix 15.2**).

Twelve RCTs (n=2,602) reported 106 (5.9%) and 20 (2.5%) cases of pollakiuria in Asia treated with SGLT-2 inhibitors and placebo, respectively (7, 8, 17, 18, 32, 69, 71, 75, 77, 79–81). NMA with 12 RCTs from Asia and 9 RCTs from Japan (17, 18, 32, 69, 71, 75, 77, 79, 80) suggested that the SGLT-2 inhibitors (empagliflozin and dapagliflozin in Asia and empagliflozin in Japan) were not associated with an increased risk of pollakiuria. Subgroup analyses of other SGLT-2 inhibitors were consistent with the overall analysis (**Appendix 15.2**).

Fifteen RCTs (n=4,429) reported 52 (1.7%) and 13 (0.9%) cases of hypovolemia (2, 3, 8, 15–17, 22, 29, 31, 53, 61, 69, 71, 80, 84), five RCTs (n=1,450) reported 17 (1. 9%) and 15 (2.7%) cases of renal impairment or failure (18, 42, 49, 52, 53), and 27 RCTs (n=7,411) reported 172 (3.3%) and 63 (2.9%) cases of UTIs (2, 7, 8, 15, 17, 22, 29, 31–33, 41, 42, 49, 52, 53, 61, 69, 71, 75, 76, 78–82, 84, 88) in Asians treated with SGLT-2 inhibitors and placebo, respectively. NMAs of 26 RCTs in Asia and 16 from Japan demonstrated that dapagliflozin and remogliflozin were not associated with an increased risk of UTIs. Other outcomes in Asia and Japan were consistent with the overall analysis (**Appendix 15.2**).

#### 3.6.3 Subgroup analysis in patients with CKD 

Subgroup analyses were performed for the 13 studies focusing on T2DM with CKD (eGFR<90 ml/min/1.73m^2^) (96–106, 108, 120). Similar results were obtained regarding pollakiuria, renal impairment or failure, fracture, and severe hypoglycemia compared to the overall analyses. However, opposite results were obtained regarding RTIs for empagliflozin, dapagliflozin, and sotagliflozin; hypovolemia for ertugliflozin and luseogliflozin; AKIs for dapagliflozin and sotagliflozin; and UTIs, DKA, and amputation for dapagliflozin in the subgroup analyses compared to the overall analyses (**Appendix 15.3**).

### 3.7 Sensitivity analysis

#### 3.7.1 Sensitivity analysis according to the interventions

Sensitivity analyses of SGLT-2 inhibitors as an add-on therapy to metformin-based treatment showed similar results for hypovolemia and renal impairment or failure compared to the overall analyses. Fewer included SGLT-2 inhibitors (canagliflozin, dapagliflozin, empagliflozin, ertugliflozin, ipragliflozin, and remogliflozin) were associated with risk of RTIs [dapagliflozin (OR 5.06, 95%CI 1.21-54.57) and empagliflozin (OR 10.86, 95%CI 2.52-116.23)]. All included SGLT-2 inhibitors were not associated with a risk of pollakiuria (canagliflozin, empagliflozin, and ipragliflozin) and UTIs (canagliflozin, dapagliflozin, empagliflozin, ertugliflozin, ipragliflozin, sotaglifozin, tofogliflozin, and remogliflozin). Details are shown in **Appendix 16.1**.

Subgroup analyses of drug-naive patients showed similar outcome results for pollakiuria, hypovolemia, and renal impairment or failure as the overall analysis. The results were similar to metformin-based treatment (**Appendix 16.1**).

#### 3.7.2 Sensitivity analysis according to the follow-up period

The risks of RTIs (canagliflozin, dapagliflozin, ertugliflozin, empagliflozin), pollakiuria (canagliflozin and empagliflozin), hypovolemia (canagliflozin, dapagliflozin, ertugliflozin, empagliflozin) and UTIs (canagliflozin, dapagliflozin, ertugliflozin, empagliflozin, expect dapagliflozin in short-term follow-up studies) of the included SGL-2 inhibitors were consistent with the overall analysis regardless of the follow-up time (**Appendix 16.2**).

## 4 Discussion

Only four head-to-head RCTs (7, 8, 88, 112) compared individual SGLT-2 inhibitors in T2DM. The comparative safety of specific SGLT-2 inhibitors remains unclear. NMA is an increasingly popular tool for comparative effectiveness or safety research. Three NMAs have compared the safety profiles of different SGLT-2 inhibitors regarding UTIs, focusing mainly on canagliflozin, empagliflozin, and dapagliflozin (6, 9, 10). A study compared the risk of RTIs, UTIs, and hypoglycemia among dapagliflozin, canagliflozin, and empagliflozin in patients with T2DM (6). Another study compared the risk of hypovolemia among dapagliflozin, canagliflozin, and empagliflozin in T2DM (10). The third study compared the risk of UTIs among dapagliflozin, canagliflozin, empagliflozin, ertugliflozin, bexagliflozin, and sotagliflozin in T2DM patients with CKD (9). This is the first comprehensive analysis comparing safety evidence of nine SGLT-2 inhibitors in patients with T2DM regarding ten adverse events (especially new outcomes: pollakiuria, renal impairment or failure, and AKI).

Most of the included studies reported RTIs (79 studies), pollakiuria (25 studies), hypovolemia (42 studies), renal impairment or failure (21 studies), and UTIs (89 studies). Few included studies reported AKI (9 studies), fracture (20 studies), DKA (7 studies), amputation (4 studies), and severe hypoglycemia (15 studies). Most patients were treated with canagliflozin, dapagliflozin, empagliflozin, ipragliflozin, and ertugliflozin. Few included studies enrolled patients treated with remogliflozin, sotagliflozin, tofogliflozin, and luseogliflozin. Therefore, outcomes based on a few studies, especially those with wider confidence intervals, should be interpreted cautiously. Further verification from more high-quality and large-sample studies is necessary.

### 4.1 Primary outcomes: RTIs

Luseogliflozin and ipragliflozin were not associated with an increased risk of RTIs. Therefore, not all SGLT-2 inhibitors increased the risk of RTIs, which differed from a previous study (6). The participants in the luseogliflozin trial were all Japanese, and most of the participants included in the ipragliflozin trial were Japanese. SGLT-2 inhibitors seem safer in the Asian population, and the association of luseogliflozin and ipragliflozin with the risk of RTIs should be studied in more ethnic groups. The subgroup analysis indicated that individual SGLT-2 inhibitors were not associated with the risk of RTIs in Asia. A meta-analysis also showed that SGLT-2 inhibitors were associated with a similar risk of RTIs compared to placebo in Japanese patients with T2DM (122).

The subgroup analysis showed that the risk of RTIs increased with increasing doses of dapagliflozin or ertugliflozin. Due to the few included studies and the few events, several doses of SGLT-2 inhibitors showed inconsistent results with wide confidence intervals and, therefore, should be interpreted cautiously.

### 4.2 Primary outcomes: pollakiuria

Dapagliflozin and empagliflozin increased the risk of pollakiuria in T2DM compared to placebo (with incidence rates of 6.73% and 3.89%). Similar results were also shown in the subgroup analyses of CKD and drug-naive patients. However, individual SGLT-2 inhibitors were not associated with a risk of pollakiuria in Asia. Several real-world studies from Asia have also shown lower pollakiuria rates in patients treated with SGLT-2 inhibitors. A Korean post-marketing surveillance study of empagliflozin (10 and 25 mg) revealed that the most common adverse event was pollakiuria, with incidence rates of 0.59% (123). A Japanese post-marketing surveillance study of 100 mg of canagliflozin showed that the most common adverse event was pollakiuria, with an incidence rate of 0.79% (124). A 36-month post-marketing surveillance study found that the pollakiuria incidence rate of tofogliflozin was 1.3% in Japanese patients (125). A study found that the increase in urine output was transient, with a return to baseline on day 2 to day 5 of treatment (126). Therefore, pollakiuria may be tolerated over time.

### 4.3 Secondary outcomes

The nine SGLT-2 inhibitors were not associated with the risk of hypovolemia, renal impairment or failure, fracture, DKA, amputation, and severe hypoglycemia compared to the placebo. Two meta-analyses found that the SGLT-2 inhibitor class was associated with an increased risk of hypovolemia, which differs from our study (127, 128). The subgroup analysis showed that canagliflozin 300 mg was associated with a significantly increased risk of hypovolemia, consistent with a previous meta-analysis (10).

Remogliflozin and dapagliflozin were associated with an increased risk of UTIs. Dapagliflozin 10 mg/d increased the risk of UTIs compared to placebo and empagliflozin 25 mg/d (6). However, a meta-analysis of three RCTs suggested that remogliflozin was not associated with UTIs. Differences may be caused by effect sizes (relative risk *versus* OR) (129). The subgroup analysis indicated that individual SGLT-2 inhibitors were not associated with the risk of UTIs in Asia.

### 4.4 Sensitivity analyses

A 3-year Japanese post-marketing surveillance study showed that drug-naive patients had significantly lower incidences of adverse events (10.81% *vs*. 20.87%; *P* < 0.001) and serious adverse events (0.86% *vs*. 2.09%; *P* < 0.001) compared to non-naive patients, as well as significantly lower incidences of pollakiuria, volume depletion-related events, and kidney disorders (130). Sensitivity analysis showed that adverse drug reactions were similar between SGLT-2 inhibitor monotherapy and add-on therapies to metformin. Few SGLT-2i were associated with the risk of RTIs, pollakiuria and UTIs compared to overall analysis, which should be interpreted with caution, as it could be affected by the number of studies and sample sizes.

Although the included studies had follow-up times ranging from 12 to 338 weeks, the median duration was 24 weeks. The sensitivity analysis based on the follow-up time showed broadly consistent results. The incidence of related adverse events in extended follow-up studies was higher than in short-term studies because the sample size was unchanged and the number of events accumulated. A 3-year Japanese post-marketing surveillance study showed that the long-term safety profile of ipragliflozin treatment in routine clinical practice was consistent with previously reported interim data at 12 or 24 months and pre-approval clinical trials (131).

### 4.5 The limitations of our study

The present NMA included all available evidence on the safety outcomes of all SGLT-2 inhibitors in patients with T2DM. Considering the homogeneity of the included studies, we deliberately excluded studies that focus on specific populations (such as T2DM with heart failure or BMI≥35 kg/m^2^ or years of age > 65 or with CKD). Meanwhile, studies with a sample size of less than 50 or with a study period of fewer than 12 weeks were excluded. Our study has several limitations. First, most of the included studies were individual SGLT-2 inhibitors versus placebo, and only four studies were conducted to compare different SGLT-2 inhibitors. Direct comparisons contributed little to these NMA results, resulting in wider CIs, greater uncertainty, and lower confidence in the evidence evaluated by CINeMA. Second, most of the included studies had a relatively small number of patients (less than 300), only ten studies had a larger sample size of 500-9,000. Third, given the limited availability of data on individual SGLT-2 inhibitors or subgroup analysis, caution is recommended when interpreting these results.

## 5 Conclusions

Not all SGLT-2 inhibitors increased the risk of RTIs. Luseogliflozin and ipragliflozin were not associated with an increased risk of RTIs. Dapagliflozin ranked first in increasing the risk of RTIs. Dapagliflozin and empagliflozin increased the risk of pollakiuria. Remogliflozin and dapagliflozin increased the risk of UTIs, and remogliflozin ranked first. SGLT-2 inhibitors were not associated with the risk of hypovolemia, renal impairment or failure, fracture, DKA, amputation, and severe hypoglycemia compared to placebo. Active-controlled trials comparing SGLT2 inhibitors are urgently needed to validate the estimates of the comparative safety produced in this network meta-analysis.

## Data availability statement

The original contributions presented in the study are included in the article/**Supplementary Material**. Further inquiries can be directed to the corresponding authors.

## Author contributions

CXL, HL, LYL and SL designed the study. CXL, LYL and CXZ set up the database. LYL, CXZ, XHG, QX, SMG, and YQW screened the literature search, acquired reports of relevant trials, selected included studies, and extracted data. CXL, LYL and CXZ did all statistical analyses, analyzed and interpreted the data and drafted the report. All authors critically reviewed the report for important intellectual content and approved the final submitted version.
