# Supplementary material for: Comparative safety of different sodium-glucose transporter 2 inhibitors in patients with type 2 diabetes: a systematic review and network meta-analysis of randomized controlled trials
Source: Front Endocrinol (Lausanne). 2023 Aug 28;14:1238399. doi: 10.3389/fendo.2023.1238399 (PMC10494439; doi:10.3389/fendo.2023.1238399)
Supplement: Supplementary file 1 [file DataSheet_1.docx]

**Supplementary Material**

**Content**

**[Appendix 1: Flow chart of literature screening 1](#_Toc15270)**

**[Appendix 2: Search strategy 2](#_Toc10066)**

**[Appendix 3: Citations of Included Studies 13](#_Toc13371)**

**[Citations for the 113 eligible studies 13](#_Toc24613)**

**[Appendix 4: Characteristics of Included Studies 22](#_Toc23846)**

**[Appendix 5: Risk of Bias Assessment 29](#_Toc14812)**

**[5.1 The results of risk of bias assessment for each study 29](#_Toc26300)**

**[5.2 The results of risk of bias assessment for each outcome 32](#_Toc7301)**

**[5.2.1 Reproductive tract infections_RoB_chart 32](#_Toc30114)**

**[5.2.2 Pollakiuria_RoB_chart 33](#_Toc11220)**

**[5.2.3 Hypovolemia_RoB_chart 34](#_Toc29135)**

**[5.2.4 Renal impairment or failure_RoB_chart 35](#_Toc7829)**

**[5.2.5 Acute kidney injury_RoB_chart 36](#_Toc13008)**

**[5.2.6 Urinary tract infections_RoB_chart 37](#_Toc8805)**

**[5.2.7 Amputation_RoB_chart 38](#_Toc6919)**

**[5.2.8 Diabetic ketoacidosis_RoB_chart 39](#_Toc13937)**

**[5.2.9 Fracture_RoB_chart 40](#_Toc500)**

**[5.2.10 Severe hypoglycemia_RoB_chart 41](#_Toc32025)**

**[Appendix 6 Trace plot and density plot, and Brooks-Gelman-Rubin diagnosis plot 41](#_Toc3538)**

**[6.1 The trace and density plot of reproductive tract infections 42](#_Toc17309)**

**[6.1 The Brooks-Gelman-Rubin diagnosis plot of reproductive tract infections 44](#_Toc24331)**

**[6.2 The trace and density plot of pollakiuria 45](#_Toc14401)**

**[6.2 The Brooks-Gelman-Rubin diagnosis plot of pollakiuria 47](#_Toc17486)**

**[6.3 The trace and density plot of hypovolemia 48](#_Toc19085)**

**[6.3 The Brooks-Gelman-Rubin diagnosis plot of hypovolemia 50](#_Toc9289)**

**[6.4 The trace and density plot of renal impairment or failure 51](#_Toc29278)**

**[6.4 The Brooks-Gelman-Rubin diagnosis plot of renal impairment or failure 51](#_Toc875)**

**[6.5 The trace and density plot of acute kidney failure 52](#_Toc28102)**

**[6.5 The Brooks-Gelman-Rubin diagnosis plot of acute kidney failure 53](#_Toc24096)**

**[6.6 The trace and density plot of urinary tract infections 54](#_Toc17940)**

**[6.6 The Brooks-Gelman-Rubin diagnosis plot of urinary tract infections 56](#_Toc25973)**

**[6.7 The trace and density plot of diabetic ketoacidosis 56](#_Toc5207)**

**[6.7 The Brooks-Gelman-Rubin diagnosis plot of diabetic ketoacidosis 57](#_Toc6099)**

**[6.8 The trace and density plot of amputation 59](#_Toc30564)**

**[6.8 The Brooks-Gelman-Rubin diagnosis plot of amputation 59](#_Toc2901)**

**[6.9 The trace and density plot of fracture 59](#_Toc9609)**

**[6.9 The Brooks-Gelman-Rubin diagnosis plot of fracture 61](#_Toc19206)**

**[6.10 The trace and density plot of severe hypoglycemia 62](#_Toc23475)**

**[6.10 The Brooks-Gelman-Rubin diagnosis plot of severe hypoglycemia 63](#_Toc19947)**

**[Appendix 7: Network plot 63](#_Toc21371)**

**[7.1 Reproductive tract infections in male 64](#_Toc4947)**

**[7.2 Reproductive tract infections in female 64](#_Toc29343)**

**[7.3 Hypovolemia 65](#_Toc1775)**

**[7.4 Renal impairment or failure 65](#_Toc18259)**

**[7.5 Acute kidney injury 66](#_Toc4701)**

**[7.6 Urinary tract infections 66](#_Toc16396)**

**[7.7 Diabetic ketoacidosis 67](#_Toc12358)**

**[7.8 Amputation 67](#_Toc17077)**

**[7.9 Fracture 68](#_Toc13637)**

**[7.10 Severe hypoglycemia 68](#_Toc9834)**

**[Appendix 8: Forest plot for network meta-analysis 69](#_Toc25904)**

**[8.1 Reproductive tract infections in male 69](#_Toc27693)**

**[8.2 Reproductive tract infections in female 69](#_Toc24442)**

**[8.3 Hypovolemia 69](#_Toc13879)**

**[8.4 Renal impairment or failure 70](#_Toc963)**

**[8.5 Acute kidney injury 70](#_Toc15053)**

**[8.6 Urinary tract infections 70](#_Toc14291)**

**[8.7 Diabetic ketoacidosis 71](#_Toc5828)**

**[8.8 Amputation 72](#_Toc664)**

**[8.9 Fracture 72](#_Toc17687)**

**[8.10 Severe hypoglycemia 72](#_Toc19419)**

**[Appendix 9: league table for network meta-analysis 74](#_Toc27707)**

**[9.1 Network meta-analysis results for reproductive tract infections in male 74](#_Toc20164)**

**[9.2 Network meta-analysis results for reproductive tract infections in female 74](#_Toc30493)**

**[9.3 Network meta-analysis results for hypovolemia 75](#_Toc32108)**

**[9.4 Network meta-analysis results for renal impairment or failure 75](#_Toc24289)**

**[9.5 Network meta-analysis results for acute kidney injury 75](#_Toc17929)**

**[9.6 Network meta-analysis results for urinary tract infections 76](#_Toc29670)**

**[9.7 Network meta-analysis results for amputation 76](#_Toc28287)**

**[9.8 Network meta-analysis results for diabetic ketoacidosis 77](#_Toc10137)**

**[9.9 Network meta-analysis results for fracture 77](#_Toc8526)**

**[9.10 Network meta-analysis results for severe hypoglycemia 77](#_Toc9748)**

**[Appendix 10: Inconsistency Analysis 78](#_Toc24386)**

**[10.1 Node-splitting: reproductive tract infections 78](#_Toc17834)**

**[10.2 Node-splitting: pollakiuria 79](#_Toc17348)**

**[10.3 Node-splitting: hypovolemia 80](#_Toc12907)**

**[10.4 Node-splitting: urinary tract infections 81](#_Toc16833)**

**[10.5 Node-splitting: severe hypoglycemia 82](#_Toc8076)**

**[Appendix 11: Heterogeneity Assessment 82](#_Toc18870)**

**[11.1 Reproductive tract infections Model 83](#_Toc3020)**

**[11.1 Heterogeneity score by each comparison for main model of reproductive tract infections 83](#_Toc8762)**

**[11.2 Pollakiuria Model 84](#_Toc11662)**

**[11.2 Heterogeneity score by each comparison for main model of pollakiuria 84](#_Toc12718)**

**[11.3 Hypovolemia Model 85](#_Toc15656)**

**[11.3 Heterogeneity score by each comparison for main model of hypovolemia 85](#_Toc29851)**

**[11.4 Renal impairment or failure Model 86](#_Toc7242)**

**[11.4 Heterogeneity score by each comparison for main model of renal impairment or failure 86](#_Toc26649)**

**[11.5 Acute kidney injury Model 87](#_Toc30022)**

**[11.5 Heterogeneity score by each comparison for main model of acute kidney injury 87](#_Toc4193)**

**[11.6 Urinary tract infections Model 88](#_Toc31196)**

**[11.6 Heterogeneity score by each comparison for main model of urinary tract infections 88](#_Toc17652)**

**[11.7 Fracture Model 89](#_Toc25923)**

**[11.7 Heterogeneity score by each comparison for main model of fracture 89](#_Toc30464)**

**[11.8 Diabetic ketoacidosis Model 90](#_Toc17275)**

**[11.8 Heterogeneity score by each comparison for main model of diabetic ketoacidosis 90](#_Toc3050)**

**[11.9 severe hypoglycemia Model 91](#_Toc20524)**

**[11.9 Heterogeneity score by each comparison for main model of severe hypoglycemia 91](#_Toc29989)**

**[11.10 Amputation Model 92](#_Toc4523)**

**[11.10 Heterogeneity score by each comparison for main model of amputation 92](#_Toc14268)**

**[Appendix 12: Contribution plots by study outcome 93](#_Toc14541)**

**[12.1 Contribution plot for reproductive tract infections 93](#_Toc26942)**

**[12.2 Contribution plot for pollakiuria 94](#_Toc21683)**

**[12.3 Contribution plot for hypovolemia 95](#_Toc5350)**

**[12.4 Contribution plot for renal impairment or failure 96](#_Toc8643)**

**[12.5 Contribution plot for acute kidney injury 97](#_Toc21308)**

**[12.6 Contribution plot for urinary tract infections 98](#_Toc13650)**

**[12.7 Contribution plot for amputation 99](#_Toc28544)**

**[12.8 Contribution plot for diabetic ketoacidosis 100](#_Toc5081)**

**[12.9 Contribution plot for fracture 101](#_Toc4503)**

**[12.10 Contribution plot for severe hypoglycemia 102](#_Toc28078)**

**[12.11 Contribution plot for reproductive tract infections in male 103](#_Toc22626)**

**[12.12 Contribution plot for reproductive tract infections in female 104](#_Toc24209)**

**[Appendix 13: Treatment Ranking using SUCRA 105](#_Toc28563)**

**[13.1 Cumulative ranking curves for hypovolemia 105](#_Toc11917)**

**[13.2 Cumulative ranking curves for renal impairment or failure 106](#_Toc17803)**

**[13.3 Cumulative ranking curves for acute kidney injury 107](#_Toc18176)**

**[13.4 Cumulative ranking curves for urinary tract infections 108](#_Toc26819)**

**[13.5 Cumulative ranking curves for amputation 109](#_Toc25799)**

**[13.6 Cumulative ranking curves for diabetic ketoacidosis 110](#_Toc4548)**

**[13.7 Cumulative ranking curves for fracture 111](#_Toc6367)**

**[13.8 Cumulative ranking curves for severe hypoglycemia 112](#_Toc17867)**

**[Appendix 14: Comparison-adjusted funnel plots 113](#_Toc29436)**

**[14.1 Comparison adjusted funnel plot for reproductive tract infections 113](#_Toc8064)**

**[14.2 Comparison adjusted funnel plot for pollakiuria 114](#_Toc19936)**

**[14.3 Comparison adjusted funnel plot for hypovolemia 115](#_Toc30313)**

**[14.4 Comparison adjusted funnel plot for renal impairment or failure 116](#_Toc13186)**

**[14.5 Comparison adjusted funnel plot for acute kidney injury 117](#_Toc8967)**

**[14.6 Comparison adjusted funnel plot for urinary tract infections 118](#_Toc16190)**

**[14.7 Comparison adjusted funnel plot for amputation 119](#_Toc19639)**

**[14.8 Comparison adjusted funnel plot for diabetic ketoacidosis 120](#_Toc22177)**

**[14.9 Comparison adjusted funnel plot for fracture 121](#_Toc22483)**

**[14.10 Comparison adjusted funnel plot for severe hypoglycemia 122](#_Toc5528)**

**[14.11 Comparison adjusted funnel plot for reproductive tract infections in male 123](#_Toc32170)**

**[14.12 Comparison adjusted funnel plot for reproductive tract infections in female 124](#_Toc28931)**

**[Appendix 15: Subgroup analysis 125](#_Toc9530)**

**[15.1 Subgroup according to different dose 125](#_Toc27391)**

**[15.1.1 Network plots for reproductive tract infections in different dose 125](#_Toc2783)**

**[15.1.1 Forest for reproductive tract infections in different dose 126](#_Toc20070)**

**[15.1.2 Network plots for pollakiuria in different dose 127](#_Toc30286)**

**[15.1.2 Forest for pollakiuria in different dose 127](#_Toc21326)**

**[15.1.3 Network plots for hypovolemia in different dose 128](#_Toc31127)**

**[15.1.3 Forest for hypovolemia in different dose 128](#_Toc23700)**

**[15.1.4 Network plots for renal impairment or failure in different dose 129](#_Toc18690)**

**[15.1.4 Forest for renal impairment or failure in different dose 129](#_Toc16057)**

**[15.1.5 Network plots for acute kidney injury in different dose 130](#_Toc31488)**

**[15.1.5 Forest for acute kidney injury in different dose 130](#_Toc17045)**

**[15.1.6 Network plots for urinary tract infections in different dose 131](#_Toc14478)**

**[15.1.6 Forest for urinary tract infections in different dose 131](#_Toc27305)**

**[15.1.7 Network plots for amputation in different dose 132](#_Toc27062)**

**[15.1.7 Forest for amputation in different dose 132](#_Toc30755)**

**[15.1.8 Network plots for diabetic ketoacidosis in different dose 133](#_Toc1812)**

**[15.1.8 Forest for diabetic ketoacidosis in different dose 133](#_Toc21704)**

**[15.1.9 Network plots for fracture in different dose 134](#_Toc16521)**

**[15.1.9 Forest for fracture in different dose 134](#_Toc29022)**

**[15.1.10 Network plots for severe hypoglycemia in different dose 135](#_Toc27267)**

**[15.1.10 Forest for severe hypoglycemia in different dose 135](#_Toc25687)**

**[15.2 Subgroup according to different regions 136](#_Toc28289)**

**[15.2.1 Forest for reproductive tract infections in Asia 136](#_Toc12993)**

**[15.2.2 Forest for reproductive tract infections in China 136](#_Toc8380)**

**[15.2.3 Forest for reproductive tract infections in Japan 136](#_Toc15816)**

**[15.2.4 Forest for pollakiuria in Asia 138](#_Toc11295)**

**[15.2.5 Forest for pollakiuria in Japan 138](#_Toc24917)**

**[15.2.6 Forest for hypovolemia in Asia 139](#_Toc3827)**

**[15.2.7 Forest for hypovolemia in Japan 139](#_Toc29833)**

**[15.2.8 Forest for renal impairment or failure in Asia 140](#_Toc13798)**

**[15.2.9 Forest for renal impairment or failure in Japan 140](#_Toc8996)**

**[15.2.10 Forest for urinary tract infections in Asia 141](#_Toc28760)**

**[15.2.11 Forest for urinary tract infections in China 141](#_Toc3870)**

**[15.2.12 Forest for urinary tract infections in Japan 141](#_Toc4122)**

**[15.2.13 Forest for fracture in Asia 142](#_Toc25585)**

**[15.2.14 Forest for fracture in Japan 142](#_Toc22519)**

**[15.3 Subgroup in patients with chronic kidney disease (CKD) 143](#_Toc4466)**

**[15.3.1 Network meta-analysis results for each outcome in patients with chronic kidney disease 143](#_Toc14280)**

**[15.3.2 Forest for reproductive tract infection in patients with CKD 144](#_Toc21030)**

**[15.3.3 Forest for pollakiuria in patients with CKD 145](#_Toc2525)**

**[15.3.4 Forest for hypovolemia in patients with CKD 146](#_Toc367)**

**[15.3.5 Forest for renal impairment or failure in patients with CKD 147](#_Toc25862)**

**[15.3.6 Forest for acute kidney injury in patients with CKD 148](#_Toc10254)**

**[15.3.7 Forest for urinary tract infections in patients with CKD 149](#_Toc28541)**

**[15.3.8 Forest for fracture in patients with CKD 150](#_Toc6604)**

**[15.3.9 Forest for amputation in patients with CKD 151](#_Toc17020)**

**[15.3.10 Forest for severe hypoglycemia in patients with CKD 152](#_Toc5444)**

**[15.3.11 Forest for diabetic ketoacidosis in patients with CKD 153](#_Toc11090)**

**[Appendix 16: Sensitivity analysis 153](#_Toc7974)**

**[16.1 According to the Interventions 154](#_Toc15907)**

**[16.1.1 Network meta-analysis results for each outcome in patients on metformin-based background therapy 154](#_Toc23660)**

**[16.1.1.1 Forest for reproductive tract infection in patients treated with metformin-based background therapy 155](#_Toc4037)**

**[16.1.1.2 Forest for pollakiuria in patients treated with metformin-based background therapy 156](#_Toc2899)**

**[16.1.1.4 Forest for renal impairment or failure in patients treated with metformin-based background therapy 158](#_Toc22349)**

**[16.1.1.5 Forest for urinary tract infections in patients treated with metformin-based background therapy 159](#_Toc31008)**

**[16.1.1.6 Forest for fracture in patients treated with metformin-based background therapy 160](#_Toc32080)**

**[16.1.2 Network meta-analysis results for each outcome in drug-naive patients 161](#_Toc3558)**

**[16.1.2.1 Forest for reproductive tract infection in drug-naive patients 162](#_Toc25133)**

**[16.1.2.2 Forest for pollakiuria in drug-naive patients 163](#_Toc27620)**

**[16.1.2.3 Forest for hypovolaemia in drug-naive patients 164](#_Toc18759)**

**[16.1.2.4 Forest for renal impairment or failure in drug-naive patients 165](#_Toc22265)**

**[16.1.2.5 Forest for urinary tract infections in drug-naive patients 166](#_Toc15360)**

**[16.2 According to the follow-up period 167](#_Toc9096)**

**[16.2.1 Network meta-analysis results according to the follow-up period 167](#_Toc9799)**

**[16.2.2 Forest for reproductive tract infection in short term patients 169](#_Toc14965)**

**[16.2.3 Forest for reproductive tract infection in long term patients 169](#_Toc3931)**

**[16.2.4 Forest for pollakiuria in short term patients 170](#_Toc26692)**

**[16.2.5 Forest for pollakiuria in long term patients 170](#_Toc29422)**

**[16.2.6 Forest for hypovolemia in short term patients 171](#_Toc32306)**

**[16.2.7 Forest for hypovolemia in long term patients 171](#_Toc22605)**

**[16.2.8 Forest for urinary tract infections in short term patients 172](#_Toc7485)**

**[16.2.9 Forest for urinary tract infections in long term patients 172](#_Toc16345)**

**[16.2.10 Forest for severe hypoglycemia in short term patients 173](#_Toc559)**

**[16.2.11 Forest for severe hypoglycemia in long term patients 173](#_Toc352)**

**[Appendix 17: CINeMA Assessment 174](#_Toc8993)**

**[17.1 Confidence in effect estimates for reproductive tract infections 174](#_Toc18960)**

**[17.2 Confidence in effect estimates for pollakiuria 175](#_Toc12830)**

**[17.3 Confidence in effect estimates for hypovolemia 176](#_Toc7956)**

**[17.4 Confidence in effect estimates for renal impairment or failure 177](#_Toc30149)**

**[17.5 Confidence in effect estimates for Acute kidney injury 178](#_Toc12836)**

**[17.6 Confidence in effect estimates for urinary tract infections 179](#_Toc12322)**

**[17.7 Confidence in effect estimates for amputation 180](#_Toc32652)**

**[17.8 Confidence in effect estimates for diabetic ketoacidosis 181](#_Toc19725)**

**[17.9 Confidence in effect estimates for fracture 182](#_Toc10618)**

**[17.10 Confidence in effect estimates for severe hypoglycemia 183](#_Toc7383)**

**[17.11 Confidence in effect estimates for reproductive tract infections in male 184](#_Toc25224)**

**[17.12 Confidence in effect estimates for reproductive tract infections in female 185](#_Toc17677)**

# Appendix 1: Flow chart of literature screening

# **
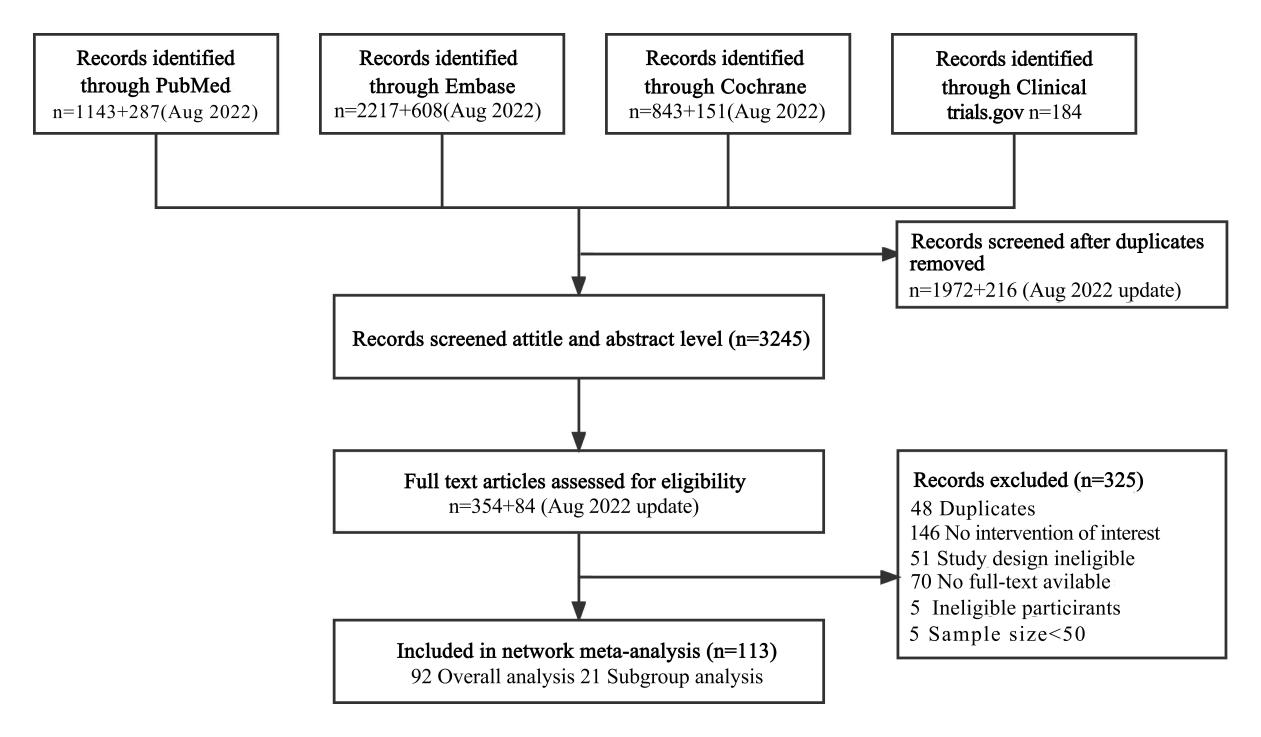
**

# Appendix 2: Search strategy

Pubmed, Embase, the Cochrane Controlled Register of Trials and ClinicalTrials.gov, from database inception to August 30, 2022. The detailed search strategy for each database was presented below:

***Pubmed***

**Intervention (sodium-glucose transporter 2 inhibitors) related**

#1"sodium-glucose transporter 2 inhibitors"[MeSH Terms]

#2 "sodium-dependent glucose co-transporter 2 inhibitors"[Title/Abstract]

#3"sodium-glucose co-transporter 2 inhibitors"[Title/Abstract]

#4 "sodium/glucose cotransporter 2 inhibitors"[Title/Abstract]

#5 "sodium-glucose cotransporter 2 inhibitors"[Title/Abstract]

#6 "sodium glucose transporter 2 inhibitors"[Title/Abstract]

#7 "sodium-glucose transporter 2 inhibitor"[Title/Abstract]

#8 "sodium glucose transporter 2 inhibitor"[Title/Abstract]

#9 "sglt 2 inhibitors"[Title/Abstract]

#10 "sglt2 inhibitors"[Title/Abstract]

#11 "sglt-2 inhibitor"[Title/Abstract]

#12 "sglt 2 inhibitor"[Title/Abstract]

#13 "sglt2 inhibitor"[Title/Abstract]

#14 "sglt-2 inhibitors"[Title/Abstract]

#15 canagliflozin[MeSH Terms]

#16 Invokana[Title/Abstract]

#17 dapagliflozin[Supplementary Concept]

#18 Farxiga[Title/Abstract]

#19 Forxiga[Title/Abstract]

#20 "BMS 512148"[Title/Abstract]

#21 "BMS512148"[Title/Abstract]

#22 "BMS-512148"[Title/Abstract]

#23 empagliflozin[Supplementary Concept]

#24 "BI 10773"[Title/Abstract]

#25 "BI10773"[Title/Abstract]

#26 "BI-10773"[Title/Abstract]

#27 "Jardiance"[Title/Abstract]

#28 ertugliflozin[Supplementary Concept]

#29 Steglatro[Title/Abstract]

#30 "PF 04971729"[Title/Abstract]

#31 "PF04971729"[Title/Abstract]

#32 "PF-04971729"[Title/Abstract]

#33 luseogliflozin[Title/Abstract]

#34 Lusefi[Title/Abstract]

#35 "TS 071"[Title/Abstract]

#36 "TS-071"[Title/Abstract]

#37 "CSG452"[Title/Abstract]

#38 Apleway[Title/Abstract]

#39 Deberza[Title/Abstract].

#40 tofogliflozin[Title/Abstract]

#41 ipragliflozin[Supplementary Concept].

#42 Suglat[Title/Abstract]

#43 "ASP1941"[Title/Abstract]

#44 "ASP-1941"[Title/Abstract]

#45 sotagliflozin[Title/Abstract]

#46 "LX4211"[Title/Abstract]

#47 "LX-4211"[Title/Abstract]

#48 "remogliflozin etabonate"[Supplementary Concept]

#49 "Remo"[Title/Abstract]

#50 "Remozen"[Title/Abstract]

#51 OR #1-50

**Outcome (safety) related**

#52 safety[MeSH Terms]

#53 safeties[Title/Abstract]

#54 "Drug-Related Side Effects and Adverse Reactions"[MeSH Terms]

#55 "Drug-Related Side Effects"[Title/Abstract]

#56"Adverse Reaction"[Title/Abstract]

#57 "Drug Related Side Effects"[Title/Abstract]

#58 "Adverse Reaction"[Title/Abstract])

#59 "Drug Side Effects"[Title/Abstract]

#60 "Drug Side Effect"[Title/Abstract]

#61 "adverse effects"[Title/Abstract]

#62 "side effects"[Title/Abstract]

#63 "adverse reaction"[Title/Abstract]

#64 "adverse reactions"[Title/Abstract]

#65 "Adverse Drug Reaction"[Title/Abstract]

#66 "ADR"[Title/Abstract]

#67 "Drug-Related Side Effects"[Title/Abstract]

#68 "severe adverse reactions"[Title/Abstract]

#69 Hypovolemia[MeSH Terms]

#70 Hypovolemias[Title/Abstract]

#71 Hypovolemic[Title/Abstract]

#72 Hypovolemics[Title/Abstract]

#73 "Volume depletion"[Title/Abstract]

#74 "Fractures, Bone"[MeSH Terms]

#75 fracture[Title/Abstract]

#76 fractures[Title/Abstract]

#77 "bone fracture"[Title/Abstract]

#78 "Reproductive Tract Infections"[MeSH Terms]

#79 "RTI"[Title/Abstract]

#80 "RTIs"[Title/Abstract]

#81 "genital tract infection"[Title/Abstract]

#82 "GTI"[Title/Abstract]

#83 "GTIs"[Title/Abstract]

#84 "reproductive tract infections"[Title/Abstract]

#85 "genital tract infections"[Title/Abstract]

#86 "genital infection"[Title/Abstract]

#87 "urinary tract infections"[MeSH Terms]

#88 "urinary infection"[Title/Abstract]

#89 "urinary tract infection"[Title/Abstract]

#90"urinary system infection"[Title/Abstract]

#91 "UTI"[Title/Abstract]

#92 "UTIs"[Title/Abstract]

#93 "urinary infections"[Title/Abstract]

#94 amputation[MeSH Terms]

#95 "lower limb amputation"[Title/Abstract]

#96 "lower extremity amputation"[Title/Abstract]

#97 amputations[Title/Abstract]

#98 amputate[Title/Abstract]

#99 "BKLE"[Title/Abstract]

#100 "BKA"[Title/Abstract]

#101 "diabetic ketoacidosis"[MeSH Terms]

#102 "diabetic ketosis"[Title/Abstract]

#103 "diabetic acidosis"[Title/Abstract]

#104 "ketoacidosis"[Title/Abstract]

#105 "diabetic ketoacidoses"[Title/Abstract]

#106 ketoacidoses[Title/Abstract]

#107 ketosis[Title/Abstract]

#108 acidosis[Title/Abstract]

#109 acidoses[Title/Abstract]

#110 "diabetic ketosis"[Title/Abstract]

#111 "DKA"[Title/Abstract]

#112 "diabetes mellitus ketoacidosis"[Title/Abstract]

#113 Hypoglycemia[MeSH Terms]

#114 "Postprandial Hypoglycemia" [Title/Abstract]

#115 "Fasting Hypoglycemia" [Title/Abstract]

#116 "Postabsorptive Hypoglycemia" [Title/Abstract]

#117 "Reactive Hypoglycemia"[Title/Abstract]

#118 "Acute Kidney Injury"[MeSH Terms]

#119 "Acute Kidney Injuries"[Title/Abstract]

#120 "Acute Renal Injury"[Title/Abstract]

#121 "Acute Renal Injuries"[Title/Abstract]

#122 "Renal Injuries Acute"[Title/Abstract]

#123 "Renal Injury Acute"[Title/Abstract]

#124 "Renal Insufficiency Acute"[Title/Abstract]

#125 "insulin degludec"(Supplementary Concept)

#126 "Renal Insufficiencies"[Title/Abstract]

#127 "Acute Renal Insufficiency"[Title/Abstract]

#128 "Acute Kidney Insufficiencies"[Title/Abstract]

#129 "Acute Kidney Insufficiency"[Title/Abstract]

#130 "Acute Kidney Failures"[Title/Abstract]

#131 "Acute Renal Failure"[Title/Abstract]

#132 "Acute Renal Failures"[Title/Abstract]

#133 "Acute Kidney Failure"[Title/Abstract]

#134 "Renal Insufficiencies"[Title/Abstract]

#135 "Kidney Insufficiency"[Title/Abstract]

#136 "Kidney Insufficiencies"[Title/Abstract]

#137 "Kidney Failure"[Title/Abstract]

#138 "Kidney Failures"[Title/Abstract]

#139 "Renal Failure"[Title/Abstract]

#140 "Renal Failures"[Title/Abstract]

#141 Polyuria[MeSH Terms]

#142 Polyurias[Title/Abstract]

#143 OR #52-142

**Studies (randomized controlled trial) related**

#144 animals[MeSH Terms]) NOT (humans[MeSH Terms]

#145 "randomized controlled trial" [Publication Type]

#146 "controlled clinical trial" [Publication Type]

#147 randomized[Title/Abstract]

#148 placebo[Title/Abstract]

#149 "drug therapy" [MeSH Subheading]

#150 randomly[Title/Abstract]

#151 trial[Title/Abstract]

#152 groups[Title/Abstract]

#153 OR #145-152

#154 #144 NOT #153

#155 #51 AND #143 AND #154

***Embase***

**Intervention (sodium-glucose transporter 2 inhibitors) related**

#1 'sodium glucose cotransporter 2 inhibitor'/de

#2 'polyuria'/de

#3 'dapagliflozin'/de

#4 'empagliflozin'/de

#5 'ertugliflozin'/de

#6 'tofogliflozin'/de

#7 'luseogliflozin'/de

#8 'sodium glucose cotransporter 2 inhibitor':ti,ab,kw

#9 canagliflozin:ti,ab,kw

#10 empagliflozin:ti,ab,kw

#11 ertugliflozin:ti,ab,kw

#12 luseogliflozin:ti,ab,kw

#13 apleway:ti,ab,kw

#14 deberza:ti,ab,kw

#15 tofogliflozin:ti,ab,kw

#16 'ipragliflozin'/de

#17 'sotagliflozin'/de

#18 'remogliflozin etabonate'/de

#19 remogliflozin:ti,ab,kw

#20 OR #1-19

**Outcome (safety) related**

#21 'safety'/de

#22 'adverse drug reaction'/de

#23 'hypovolemia'/de

#24 'fracture'/de

#25 'genital tract infection'/de

#26 'urinary tract infection'/de

#27 'amputation'/de

#28 'diabetic ketoacidosis'/de

#29 'hypoglycemia'/de

#30 'acute kidney failure'/de

#31 'kidney failure'/de

#32 'polyuria'/de

#33 safeties:ti,ab,kw.

#34 'adverse event':ti,ab,kw

#35 'adverse drug reaction':ti,ab,kw

#36 hypovolemia:ti,ab,kw

#37 hypovolemics:ti,ab,kw

#38 'volume depletion':ti,ab,kw

#39 'limb fracture':ti,ab,kw

#40 'leg fracture':ti,ab,kw

#41 'rtis':ti,ab,kw

#42 'genital tract infection':ti,ab,kw

#43 'urinary tract infection':ti,ab,kw

#44 'leg amputation':ti,ab,kw

#45 amputations:ti,ab,kw

#46 amputate:ti,ab,kw

#47 'bkle':ti,ab,kw.

#48 'bka':ti,ab,kw

#49 'diabetic ketoacidosis':ti,ab,kw

#50 'ketoacidosis':ti,ab,kw

#51 ketoacidoses:ti,ab,kw

#52 ketosis:ti,ab,kw

#53 acidoses:ti,ab,kw

#54 'dka':ti,ab,kw

#55 'kidney failure':ti,ab,kw

#56 'acute kidney failure':ti,ab,kw

#57 'kidney injury':ti,ab,kw

#58 polyurias:ti,ab,kw

#59 polyuria:ti,ab,kw

#60 pollakisuria:ti,ab,kw

#61 OR#21-60

**Studies (randomized controlled trial) related**

#62 'crossover procedure'/de

#63 'double-blind procedure'/de

#64 'randomized controlled trial'/de

#65 'single-blind procedure'/de

#66 (random* OR factorial* OR crossover* OR cross NEXT/1 over* OR placebo* OR doubl* NEAR/1 blind* OR singl* NEAR/1 blind* OR assign* OR allocat* OR volunteer*):ti,ab,kw

#67 OR#62-66

#68 #20 AND #61 AND #67

***Cochrane Controlled Register of Trials***

***(CENTRAL)***

**Intervention (sodium-glucose transporter 2 inhibitors) related**

#1 MeSH descriptor: [Canagliflozin] this term only

#2 ("Canagliflozin Hemihydrate"):ti,ab,kw

#3 ("Canagliflozin, Anhydrous"):ti,ab,kw

#4 ("Invokana"):ti,ab,kw

#5 MeSH descriptor: [Sodium-Glucose Transporter 2 Inhibitors] this term only

#6 ("Sodium Glucose Transporter 2 Inhibitor"):ti,ab,kw

#7 ("Sodium-Glucose Transporter 2 Inhibitor"):ti,ab,kw

#8 ("Sodium Glucose Transporter 2 Inhibitors"):ti,ab,kw

#9 ("SGLT 2 Inhibitor"):ti,ab,kw

#10 ("SGLT-2 Inhibitor"):ti,ab,kw

#11 ("SGLT 2 Inhibitors"):ti,ab,kw

#12 ("SGLT-2 Inhibitors"):ti,ab,kw

#13 ("Inhibitor, SGLT-2"):ti,ab,kw

#14 ("SGLT2 Inhibitor"):ti,ab,kw

#15 ("Inhibitor, SGLT2"):ti,ab,kw

#16 ("SGLT2 Inhibitors"):ti,ab,kw

#17 (Gliflozin):ti,ab,kw

#18 (Gliflozins):ti,ab,kw

#19 ("Dapagliflozin"):ti,ab,kw

#20 (empagliflozin):ti,ab,kw

#21 (ertugliflozin):ti,ab,kw

#22 (luseogliflozin):ti,ab,kw

#23 (tofogliflozin):ti,ab,kw

#24 (Farxiga):ti,ab,kw

#25(Forxiga):ti,ab,kw

#26 ("BMS 512148"):ti,ab,kw

#27 ("BMS512148"):ti,ab,kw

#28 ("BMS-512148"):ti,ab,kw

#29 ("BI 10773"):ti,ab,kw

#30 ("BI10773"):ti,ab,kw

#31 ("BI-10773"):ti,ab,kw

#32 ("Jardiance"):ti,ab,kw

#33 (Steglatro):ti,ab,kw

#34 ("PF 04971729"):ti,ab,kw

#35 ("PF04971729"):ti,ab,kw

#36 ("PF-04971729"):ti,ab,kw

#37 (Lusefi):ti,ab,kw

#38 ("TS 071"):ti,ab,kw

#39 ("TS-071"):ti,ab,kw

#40 ("CSG452"):ti,ab,kw

#41 (Apleway):ti,ab,kw

#42 (Deberza):ti,ab,kw

#43 ("remogliflozin etabonate"):ti,ab,kw

#44 (ipragliflozin):ti,ab,kw

#45 (Suglat):ti,ab,kw

#46 ("ASP1941"):ti,ab,kw

#47 ("ASP-1941"):ti,ab,kw

#48 (sotagliflozin):ti,ab,kw

#49 OR/#1-#48

**Outcome (safety) related**

#50 MeSH descriptor: [Safety] this term only

#51 (Safeties):ti,ab,kw

#52 MeSH descriptor: [Drug-Related Side Effects and Adverse Reactions] this term only

#53 ("Drug Related Side Effects and Adverse Reaction"):ti,ab,kw

#54 ("Drug Related Side Effects and Adverse Reactions"):ti,ab,kw

#55 ("Side Effects of Drugs"):ti,ab,kw

#56 ("Drug Reaction, Adverse"):ti,ab,kw

#57 ("Drug Event, Adverse"):ti,ab,kw

#58 ("Adverse Drug Reactions"):ti,ab,kw

#59 ("Adverse Drug Event"):ti,ab,kw

#60 ("Drug Events, Adverse"):ti,ab,kw

#61 ("Drug-Related Side Effects and Adverse Reaction"):ti,ab,kw

#62 ("Adverse Drug Reactions"):ti,ab,kw

#63 ("Adverse Drug Event"):ti,ab,kw

#64("Drug Events, Adverse"):ti,ab,kw

#65 ("Drug Side Effects"):ti,ab,kw

#66 ("Drug Reactions, Adverse"):ti,ab,kw

#67 ("Reactions, Adverse Drug"):ti,ab,kw

#68 ("Drug Side Effect"):ti,ab,kw

#69 ("Effects, Drug Side"):ti,ab,kw

#70 ("Side Effect, Drug"):ti,ab,kw

#71 ("Adverse Drug Events"):ti,ab,kw

#72 ("Side Effects, Drug"):ti,ab,kw

#73 ("Adverse Drug Reaction"):ti,ab,kw

#74 ("Drug Toxicities"):ti,ab,kw

#75 ("Toxicities, Drug"):ti,ab,kw

#76 ("Toxicity, Drug"):ti,ab,kw

#77 ("Drug Toxicity"):ti,ab,kw

#78 MeSH descriptor: [Hypovolemia] this term only

#79 (Hypovolemias):ti,ab,kw

#80 (Hypovolemic):ti,ab,kw

#81 (Hypovolemics):ti,ab,kw

#82 MeSH descriptor: [Fractures, Bone] this term only

#83("Broken Bone"):ti,ab,kw

#84 ("Bone, Broken"):ti,ab,kw

#85 ("Broken Bones"):ti,ab,kw

#86 ("Bone Fractures"):ti,ab,kw

#87 ("Bones, Broken"):ti,ab,kw

#88 ("Bone Fracture"):ti,ab,kw

#89 ("Fracture, Bone"):ti,ab,kw

#90 ("Torsion Fracture"):ti,ab,kw

#91 ("Fractures, Torsion"):ti,ab,kw

#92 ("Fractures, Torsion"):ti,ab,kw

#93 ("Fracture, Spiral"):ti,ab,k

#94 ("Fractures, Spiral"):ti,ab,kw

#95 ("Spiral Fracture"):ti,ab,kw

#96 ("Torsion Fractures"):ti,ab,kw

#97 ("Fracture, Torsion"):ti,ab,kw

#98 MeSH descriptor: [Reproductive Tract Infections] this term only

#99 ("Genital Tract Infections"):ti,ab,kw

#100 ("Reproductive Tract Infection"):ti,ab,kw

#101 ("Infection, Genital Tract"):ti,ab,kw

#102 ("Infections, Reproductive Tract"):ti,ab,kw

#103 ("Infection, Reproductive Tract"):ti,ab,kw

#104 ("Infections, Genital Tract"):ti,ab,kw

#105 ("Genital Tract Infection"):ti,ab,kw

#106 MeSH descriptor: [Urinary Tract Infections] this term only

#107 ("Tract Infections, Urinary"):ti,ab,kw

#108 ("Infection, Urinary Tract"):ti,ab,kw

#109 ("Tract Infection, Urinary"):ti,ab,kw

#110 ("Urinary Tract Infection"):ti,ab,kw

#111 ("Infections, Urinary Tract"):ti,ab,kw

#112 MeSH descriptor: [Amputation] this term only

#113 (Amputations):ti,ab,kw

#114 MeSH descriptor: [Diabetic Ketoacidosis] this term only

#115 ("Diabetic Ketoacidosis, DKA"):ti,ab,kw

#116 ("Ketoacidosis, DKA Diabetic"):ti,ab,kw

#117 ("DKA Diabetic Ketoacidoses"):ti,ab,kw

#118 ("DKA Diabetic Ketoacidosis"):ti,ab,kw

#119 ("Diabetic Acidoses"):ti,ab,kw

#120 ("Diabetic Ketoacidoses"):ti,ab,kw

#121 ("Diabetic Ketoses"):ti,ab,kw

#122 ("Ketoacidoses, Diabetic"):ti,ab,kw

#123 ("Acidosis, Diabetic"):ti,ab,kw

#124 ("Diabetic Ketosis"):ti,ab,kw

#125("Acidoses, Diabetic"):ti,ab,kw

#126 ("Ketosis, Diabetic"):ti,ab,kw

#127 ("Ketoacidosis, Diabetic"):ti,ab,kw

#128 ("Diabetic Acidosis"):ti,ab,kw

#129 ("Ketoses, Diabetic"):ti,ab,kw

#130 MeSH descriptor: [Hypoglycemia] this term only

#131 ("Hypoglycemia, Fasting"):ti,ab,kw

#132 ("Fasting Hypoglycemia"):ti,ab,kw

#133 ("Hypoglycemia, Postabsorptive"):ti,ab,kw

#134 ("Postabsorptive Hypoglycemia"):ti,ab,kw

#135 ("Reactive Hypoglycemia"):ti,ab,kw

#136("Hypoglycemia, Postprandial"):ti,ab,kw

#137 ("Postprandial Hypoglycemia"):ti,ab,kw

#138 ("Hypoglycemia, Reactive"):ti,ab,kw

#139 MeSH descriptor: [Acute Kidney Injury] this term only

#140 ("Renal Injury, Acute"):ti,ab,kw

#141("Kidney Injury, Acute"):ti,ab,kw

#142 ("Renal Injuries, Acute"):ti,ab,kw

#143 ("Acute Kidney Injuries"):ti,ab,kw

#144 ("Acute Renal Injury"):ti,ab,kw

#145 ("Acute Renal Injuries"):ti,ab,kw

#146 ("Kidney Injuries, Acute"):ti,ab,kw

#147 ("Acute Renal Failures"):ti,ab,kw

#148 ("Kidney Failure, Acute"):ti,ab,kw

#149 ("Acute Renal Failure"):ti,ab,kw

#150 ("Acute Kidney Failure"):ti,ab,kw

#151 ("Acute Kidney Failures"):ti,ab,kw

#152 ("Kidney Failures, Acute"):ti,ab,kw

#153 ("Renal Failure, Acute"):ti,ab,kw

#154 ("Renal Failures, Acute"):ti,ab,kw

#155 ("Acute Kidney Insufficiencies"):ti,ab,kw

#156 ("Acute Renal Insufficiency"):ti,ab,kw

#157 ("Acute Renal Insufficiencies"):ti,ab,kw

#158 ("Renal Insufficiencies, Acute"):ti,ab,kw

#159 ("Renal Insufficiency, Acute"):ti,ab,kw

#160 ("Kidney Insufficiency, Acute"):ti,ab,kw

#161 ("Kidney Insufficiencies, Acute"):ti,ab,kw

#162("Acute Kidney Insufficiency"):ti,ab,kw

#163 MeSH descriptor: [Renal Insufficiency] this term only

#164 ("Kidney Failures"):ti,ab,kw

#165 ("Renal Failure"):ti,ab,kw

#166 ("Failure, Renal"):ti,ab,kw

#167 ("Renal Failures"):ti,ab,kw

#168 ("Failures, Renal"):ti,ab,kw

#169 ("Kidney Failure"):ti,ab,kw

#170 ("Failures, Kidney"):ti,ab,kw

#171 ("Failure, Kidney"):ti,ab,kw

#172 ("Renal Insufficiencies"):ti,ab,kw

#173 ("Kidney Insufficiency"):ti,ab,kw

#174 ("Kidney Insufficiencies"):ti,ab,kw

#175 ("Insufficiency, Kidney"):ti,ab,kw

#176 MeSH descriptor: [Polyuria] this term only

#177 (Polyurias):ti,ab,kw

#178 OR/#50-#177

**Studies (randomized controlled trial) related**

#179 MeSH descriptor: [Randomized Controlled Trial] this term only

#180 ("randomized control trial"):ti,ab,kw

#181 ("Randomized controlled trials"):ti,ab,kw

#182 ("RCT"):ti,ab,kw

#183 ("RCTs"):ti,ab,kw

#184("Randomized Controlled Trial"):ti,ab,kw

#185 OR/#179-#184

#186 #49 AND #178 AND #185

***ClinicalTrials.gov***

#1 Condition or disease: Type 2 diabetes

#2 Study Results: Studies With Results

#3 Intervention/treatment: SGLT2

Appendix 3: Citations of Included Studies

## Citations for the 113 eligible studies

1. List JF, Woo V, Morales E, Tang W, Fiedorek FT. Sodium-glucose cotransport inhibition with dapagliflozin in type 2 diabetes. Diabetes Care. 2009;32(4):650-7.

2. Bailey CJ, Gross JL, Pieters A, Bastien A, List JF. Effect of dapagliflozin in patients with type 2 diabetes who have inadequate glycaemic control with metformin: a randomised, double-blind, placebo-controlled trial. Lancet. 2010;375(9733):2223-33.

3. Ferrannini E, Ramos SJ, Salsali A, Tang W, List JF. Dapagliflozin monotherapy in type 2 diabetic patients with inadequate glycemic control by diet and exercise: a randomized, double-blind, placebo-controlled, phase 3 trial. Diabetes Care. 2010;33(10):2217-24.

4. Strojek K, Yoon KH, Hruba V, Elze M, Langkilde AM, Parikh S. Effect of dapagliflozin in patients with type 2 diabetes who have inadequate glycaemic control with glimepiride: a randomized, 24-week, double-blind, placebo-controlled trial. Diabetes Obes Metab. 2011;13(10):928-38.

5. Bailey CJ, Iqbal N, T'Joen C, List JF. Dapagliflozin monotherapy in drug-naÃ¯ve patients with diabetes: a randomized-controlled trial of low-dose range. Diabetes, obesity & metabolism. 2012;14(10):951‐9.

6. Bolinder J, Ljunggren Ö, Kullberg J, Johansson L, Wilding J, Langkilde AM, et al. Effects of dapagliflozin on body weight, total fat mass, and regional adipose tissue distribution in patients with type 2 diabetes mellitus with inadequate glycemic control on metformin. The Journal of clinical endocrinology and metabolism. 2012;97(3):1020-31.

7. Rosenstock J, Aggarwal N, Polidori D, Zhao Y, Arbit D, Usiskin K, et al. Dose-ranging effects of canagliflozin, a sodium-glucose cotransporter 2 inhibitor, as add-on to metformin in subjects with type 2 diabetes. Diabetes Care. 2012;35(6):1232-8.

8. Rosenstock J, Vico M, Wei L, Salsali A, List JF. Effects of dapagliflozin, an SGLT2 inhibitor, on HbA(1c), body weight, and hypoglycemia risk in patients with type 2 diabetes inadequately controlled on pioglitazone monotherapy. Diabetes Care. 2012;35(7):1473-8.

9. Wilding JPH, Woo V, Soler NG, Pahor AP, Sugg J, Rohwedder K, et al. Long-term efficacy of dapagliflozin in patients with type 2 diabetes mellitus receiving high doses of insulin a randomized trial. Annals of Internal Medicine. 2012;156(6):405-15.

10. Inagaki N, Kondo K, Yoshinari T, Maruyama N, Susuta Y, Kuki H. Efficacy and safety of canagliflozin in Japanese patients with type 2 diabetes: a randomized, double-blind, placebo-controlled, 12-week study. Diabetes, obesity & metabolism. 2013;15(12):1136‐45.

11. Bode B, Stenlöf K, Sullivan D, Fung A, Usiskin K. Efficacy and safety of canagliflozin treatment in older subjects with type 2 diabetes mellitus: a randomized trial. Hospital practice (1995). 2013;41(2):72-84.

12. Ferrannini E, Seman L, Seewaldt-Becker E, Hantel S, Pinnetti S, Woerle HJ. A Phase IIb, randomized, placebo-controlled study of the SGLT2 inhibitor empagliflozin in patients with type 2 diabetes. Diabetes, obesity & metabolism. 2013;15(8):721‐8.

13. Fonseca VA, Ferrannini E, Wilding JP, Wilpshaar W, Dhanjal P, Ball G, et al. Active- and placebo-controlled dose-finding study to assess the efficacy, safety, and tolerability of multiple doses of ipragliflozin in patients with type 2 diabetes mellitus. J Diabetes Complications. 2013;27(3):268-73.

14. Häring HU, Merker L, Seewaldt-Becker E, Weimer M, Meinicke T, Woerle HJ, et al. Empagliflozin as add-on to metformin plus sulfonylurea in patients with type 2 diabetes: a 24-week, randomized, double-blind, placebo-controlled trial. Diabetes care. 2013;36(11):3396‐404.

15. Kaku K, Inoue S, Matsuoka O, Kiyosue A, Azuma H, Hayashi N, et al. Efficacy and safety of dapagliflozin as a monotherapy for type 2 diabetes mellitus in Japanese patients with inadequate glycaemic control: a phase II multicentre, randomized, double-blind, placebo-controlled trial. Diabetes Obes Metab. 2013;15(5):432-40.

16. Roden M, Weng J, Eilbracht J, Delafont B, Kim G, Woerle HJ, et al. Empagliflozin monotherapy with sitagliptin as an active comparator in patients with type 2 diabetes: a randomised, double-blind, placebo-controlled, phase 3 trial. The lancet Diabetes & endocrinology. 2013;1(3):208‐19.

17. Rosenstock J, Seman LJ, Jelaska A, Hantel S, Pinnetti S, Hach T, et al. Efficacy and safety of empagliflozin, a sodium glucose cotransporter 2 (SGLT2) inhibitor, as add-on to metformin in type 2 diabetes with mild hyperglycaemia. Diabetes, obesity & metabolism. 2013;15(12):1154‐60.

18. Stenlöf K, Cefalu WT, Kim KA, Alba M, Usiskin K, Tong C, et al. Efficacy and safety of canagliflozin monotherapy in subjects with type 2 diabetes mellitus inadequately controlled with diet and exercise. Diabetes Obes Metab. 2013;15(4):372-82.

19. Wilding JP, Ferrannini E, Fonseca VA, Wilpshaar W, Dhanjal P, Houzer A. Efficacy and safety of ipragliflozin in patients with type 2 diabetes inadequately controlled on metformin: a dose-finding study. Diabetes Obes Metab. 2013;15(5):403-9.

20. Wilding JPH, Charpentier G, Hollander P, González-Gálvez G, Mathieu C, Vercruysse F, et al. Efficacy and safety of canagliflozin in patients with type 2 diabetes mellitus inadequately controlled with metformin and sulphonylurea: A randomised trial. International Journal of Clinical Practice. 2013;67(12):1267-82.

21. Yale JF, Bakris G, Cariou B, Yue D, David-Neto E, Xi L, et al. Efficacy and safety of canagliflozin in subjects with type 2 diabetes and chronic kidney disease. Diabetes Obes Metab. 2013;15(5):463-73.

22. Barnett AH, Mithal A, Manassie J, Jones R, Rattunde H, Woerle HJ, et al. Efficacy and safety of empagliflozin added to existing antidiabetes treatment in patients with type 2 diabetes and chronic kidney disease: a randomised, double-blind, placebo-controlled trial. The lancet Diabetes & endocrinology. 2014;2(5):369‐84.

23. Häring HU, Merker L, Seewaldt-Becker E, Weimer M, Meinicke T, Broedl UC, et al. Empagliflozin as add-on to metformin in patients with type 2 diabetes: a 24-week, randomized, double-blind, placebo-controlled trial. Diabetes care. 2014;37(6):1650‐9.

24. Inagaki N, Kondo K, Yoshinari T, Takahashi N, Susuta Y, Kuki H. Efficacy and safety of canagliflozin monotherapy in Japanese patients with type 2 diabetes inadequately controlled with diet and exercise: a 24-week, randomized, double-blind, placebo-controlled, Phase III study. Expert opinion on pharmacotherapy. 2014;15(11):1501‐15.

25. Ji L, Ma J, Li H, Mansfield TA, T'Joen CL, Iqbal N, et al. Dapagliflozin as monotherapy in drug-naive Asian patients with type 2 diabetes mellitus: a randomized, blinded, prospective phase III study. Clinical therapeutics. 2014;36(1):84‐100.e9.

26. Kadowaki T, Haneda M, Inagaki N, Terauchi Y, Taniguchi A, Koiwai K, et al. Empagliflozin monotherapy in Japanese patients with type 2 diabetes mellitus: a randomized, 12-week, double-blind, placebo-controlled, phase II trial. Adv Ther. 2014;31(6):621-38.

27. Kaku K, Watada H, Iwamoto Y, Utsunomiya K, Terauchi Y, Tobe K, et al. Efficacy and safety of monotherapy with the novel sodium/glucose cotransporter-2 inhibitor tofogliflozin in Japanese patients with type 2 diabetes mellitus: a combined Phase 2 and 3 randomized, placebo-controlled, double-blind, parallel-group comparative study. Cardiovascular diabetology. 2014;13(1).

28. Kashiwagi A, Kazuta K, Yoshida S, Nagase I. Randomized, placebo-controlled, double-blind glycemic control trial of novel sodium-dependent glucose cotransporter 2 inhibitor ipragliflozin in Japanese patients with type 2 diabetes mellitus. J Diabetes Investig. 2014;5(4):382-91.

29. Kohan DE, Fioretto P, Tang W, List JF. Long-term study of patients with type 2 diabetes and moderate renal impairment shows that dapagliflozin reduces weight and blood pressure but does not improve glycemic control. Kidney international. 2014;85(4):962‐71.

30. Kovacs CS, Seshiah V, Swallow R, Jones R, Rattunde H, Woerle HJ, et al. Empagliflozin improves glycaemic and weight control as add-on therapy to pioglitazone or pioglitazone plus metformin in patients with type 2 diabetes: a 24-week, randomized, placebo-controlled trial. Diabetes, obesity & metabolism. 2014;16(2):147‐58.

31. Leiter LA, Cefalu WT, de Bruin TW, Gause-Nilsson I, Sugg J, Parikh SJ. Dapagliflozin added to usual care in individuals with type 2 diabetes mellitus with preexisting cardiovascular disease: a 24-week, multicenter, randomized, double-blind, placebo-controlled study with a 28-week extension. Journal of the American Geriatrics Society. 2014;62(7):1252‐62.

32. Qiu R, Capuano G, Meininger G. Efficacy and safety of twice-daily treatment with canagliflozin, a sodium glucose co-transporter 2 inhibitor, added on to metformin monotherapy in patients with type 2 diabetes mellitus. Journal of clinical and translational endocrinology. 2014;1(2):54‐60.

33. Seino Y, Sasaki T, Fukatsu A, Sakai S, Samukawa Y. Efficacy and safety of luseogliflozin monotherapy in Japanese patients with type 2 diabetes mellitus: a 12-week, randomized, placebo-controlled, phase II study. Current medical research and opinion. 2014;30(7):1219‐30.

34. Seino Y, Sasaki T, Fukatsu A, Ubukata M, Sakai S, Samukawa Y. Dose-finding study of luseogliflozin in Japanese patients with type 2 diabetes mellitus: a 12-week, randomized, double-blind, placebo-controlled, phase II study. Current medical research and opinion. 2014;30(7):1231‐44.

35. Seino Y, Sasaki T, Fukatsu A, Ubukata M, Sakai S, Samukawa Y. Efficacy and safety of luseogliflozin as monotherapy in Japanese patients with type 2 diabetes mellitus: a randomized, double-blind, placebo-controlled, phase 3 study. Current medical research and opinion. 2014;30(7):1245‐55.

36. Amin NB, Wang X, Jain SM, Lee DS, Nucci G, Rusnak JM. Dose-ranging efficacy and safety study of ertugliflozin, a sodium-glucose co-transporter 2 inhibitor, in patients with type 2 diabetes on a background of metformin. Diabetes Obes Metab. 2015;17(6):591-8.

37. Bailey CJ, Morales Villegas EC, Woo V, Tang W, Ptaszynska A, List JF. Efficacy and safety of dapagliflozin monotherapy in people with Type 2 diabetes: a randomized double-blind placebo-controlled 102-week trial. Diabetic medicine. 2015;32(4):531‐41.

38. Cefalu WT, Leiter LA, de Bruin TW, Gause-Nilsson I, Sugg J, Parikh SJ. Dapagliflozin's Effects on Glycemia and Cardiovascular Risk Factors in High-Risk Patients With Type 2 Diabetes: A 24-Week, Multicenter, Randomized, Double-Blind, Placebo-Controlled Study With a 28-Week Extension. Diabetes Care. 2015;38(7):1218-27.

39. Ikeda S, Takano Y, Cynshi O, Tanaka R, Christ AD, Boerlin V, et al. A novel and selective sodium-glucose cotransporter-2 inhibitor, tofogliflozin, improves glycaemic control and lowers body weight in patients with type 2 diabetes mellitus. Diabetes Obes Metab. 2015;17(10):984-93.

40. Ji L, Han P, Liu Y, Yang G, Dieu Van NK, Vijapurkar U, et al. Canagliflozin in Asian patients with type 2 diabetes on metformin alone or metformin in combination with sulphonylurea. Diabetes, obesity & metabolism. 2015;17(1):23‐31.

41. Kashiwagi A, Akiyama N, Shiga T, Kazuta K, Utsuno A, Yoshida S, et al. Efficacy and safety of ipragliflozin as an add-on to a sulfonylurea in Japanese patients with inadequately controlled type 2 diabetes: results of the randomized, placebo-controlled, double-blind, phase III EMIT study. Diabetology International. 2015;6(2):125-38.

42. Kashiwagi A, Kazuta K, Goto K, Yoshida S, Ueyama E, Utsuno A. Ipragliflozin in combination with metformin for the treatment of Japanese patients with type 2 diabetes: ILLUMINATE, a randomized, double-blind, placebo-controlled study. Diabetes Obes Metab. 2015;17(3):304-8.

43. Kashiwagi A, Kazuta K, Takinami Y, Yoshida S, Utsuno A, Nagase I. Ipragliflozin improves glycemic control in Japanese patients with type 2 diabetes mellitus: the BRIGHTEN study: BRIGHTEN: double-blind randomized study of ipragliflozin to show its efficacy as monotherapy in T2DM patients. Diabetology International. 2015;6(1):8-18.

44. Kashiwagi A, Shiga T, Akiyama N, Kazuta K, Utsuno A, Yoshida S, et al. Efficacy and safety of ipragliflozin as an add-on to pioglitazone in Japanese patients with inadequately controlled type 2 diabetes: a randomized, double-blind, placebo-controlled study (the SPOTLIGHT study). Diabetology International. 2015;6(2):104-16.

45. Kashiwagi A, Takahashi H, Ishikawa H, Yoshida S, Kazuta K, Utsuno A, et al. A randomized, double-blind, placebo-controlled study on long-term efficacy and safety of ipragliflozin treatment in patients with type 2 diabetes mellitus and renal impairment: results of the long-term ASP1941 safety evaluation in patients with type 2 diabetes with renal impairment (LANTERN) study. Diabetes Obes Metab. 2015;17(2):152-60.

46. Mathieu C, Ranetti AE, Li D, Ekholm E, Cook W, Hirshberg B, et al. Randomized, Double-Blind, Phase 3 Trial of Triple Therapy With Dapagliflozin Add-on to Saxagliptin Plus Metformin in Type 2 Diabetes. Diabetes care. 2015;38(11):2009‐17.

47. Matthaei S, Bowering K, Rohwedder K, Grohl A, Parikh S. Dapagliflozin improves glycemic control and reduces body weight as add-on therapy to metformin plus sulfonylurea: a 24-week randomized, double-blind clinical trial. Diabetes Care. 2015;38(3):365-72.

48. Merker L, Häring HU, Christiansen AV, Roux F, Salsali A, Kim G, et al. Empagliflozin as add-on to metformin in people with Type 2 diabetes. Diabetic medicine : a journal of the British Diabetic Association. 2015;32(12):1555-67.

49. Rosenstock J, Cefalu WT, Lapuerta P, Zambrowicz B, Ogbaa I, Banks P, et al. Greater dose-ranging effects on A1C levels than on glucosuria with LX4211, a dual inhibitor of SGLT1 and SGLT2, in patients with type 2 diabetes on metformin monotherapy. Diabetes care. 2015;38(3):431‐8.

50. Rosenstock J, Jelaska A, Zeller C, Kim G, Broedl UC, Woerle HJ. Impact of empagliflozin added on to basal insulin in type 2 diabetes inadequately controlled on basal insulin: a 78-week randomized, double-blind, placebo-controlled trial. Diabetes Obes Metab. 2015;17(10):936-48.

51. Ross S, Thamer C, Cescutti J, Meinicke T, Woerle HJ, Broedl UC. Efficacy and safety of empagliflozin twice daily versus once daily in patients with type 2 diabetes inadequately controlled on metformin: a 16-week, randomized, placebo-controlled trial. Diabetes Obes Metab. 2015;17(7):699-702.

52. Schumm-Draeger PM, Burgess L, Korányi L, Hruba V, Hamer-Maansson JE, de Bruin TW. Twice-daily dapagliflozin co-administered with metformin in type 2 diabetes: a 16-week randomized, placebo-controlled clinical trial. Diabetes, obesity & metabolism. 2015;17(1):42‐51.

53. Sykes AP, Kemp GL, Dobbins R, O'Connor-Semmes R, Almond SR, Wilkison WO, et al. Randomized efficacy and safety trial of once-daily remogliflozin etabonate for the treatment of type 2 diabetes. Diabetes, obesity & metabolism. 2015;17(1):98‐101.

54. Sykes AP, O'Connor-Semmes R, Dobbins R, Dorey DJ, Lorimer JD, Walker S, et al. Randomized trial showing efficacy and safety of twice-daily remogliflozin etabonate for the treatment of type 2 diabetes. Diabetes, obesity & metabolism. 2015;17(1):94‐7.

55. Tikkanen I, Narko K, Zeller C, Green A, Salsali A, Broedl UC, et al. Empagliflozin reduces blood pressure in patients with type 2 diabetes and hypertension. Diabetes Care. 2015;38(3):420-8.

56. Zinman B, Wanner C, Lachin JM, Fitchett D, Bluhmki E, Hantel S, et al. Empagliflozin, cardiovascular outcomes, and mortality in type 2 diabetes. New England Journal of Medicine. 2015;373(22):2117‐28.

57. Araki E, Onishi Y, Asano M, Kim H, Ekholm E, Johnsson E, et al. Efficacy and safety of dapagliflozin in addition to insulin therapy in Japanese patients with type 2 diabetes: results of the interim analysis of 16-week double-blind treatment period. Journal of diabetes investigation. 2016;7(4):555‐64.

58. Frías JP, Guja C, Hardy E, Ahmed A, Dong F, Öhman P, et al. Exenatide once weekly plus dapagliflozin once daily versus exenatide or dapagliflozin alone in patients with type 2 diabetes inadequately controlled with metformin monotherapy (DURATION-8): a 28 week, multicentre, double-blind, phase 3, randomised controlled trial. The lancet Diabetes & endocrinology. 2016;4(12):1004‐16.

59. Haneda M, Seino Y, Inagaki N, Kaku K, Sasaki T, Fukatsu A, et al. Influence of Renal Function on the 52-Week Efficacy and Safety of the Sodium Glucose Cotransporter 2 Inhibitor Luseogliflozin in Japanese Patients with Type 2 Diabetes Mellitus. Clinical therapeutics. 2016;38(1):66‐88.e20.

60. Inagaki N, Harashima S, Maruyama N, Kawaguchi Y, Goda M, Iijima H. Efficacy and safety of canagliflozin in combination with insulin: a double-blind, randomized, placebo-controlled study in Japanese patients with type 2 diabetes mellitus. Cardiovasc Diabetol. 2016;15:89.

61. Ishihara H, Yamaguchi S, Nakao I, Okitsu A, Asahina S. Efficacy and safety of ipragliflozin, an SGLT2 inhibitor, add-on to insulin in Japanese patients: results of a double-blind, placebo-controlled study. Diabetes. 2016;65:A64‐.

62. Lu CH, Min KW, Chuang LM, Kokubo S, Yoshida S, Cha BS. Efficacy, safety, and tolerability of ipragliflozin in Asian patients with type 2 diabetes mellitus and inadequate glycemic control with metformin: Results of a phase 3 randomized, placebo-controlled, double-blind, multicenter trial. J Diabetes Investig. 2016;7(3):366-73.

63. Rodbard HW, Seufert J, Aggarwal N, Cao A, Fung A, Pfeifer M, et al. Efficacy and safety of titrated canagliflozin in patients with type 2 diabetes mellitus inadequately controlled on metformin and sitagliptin. Diabetes, obesity & metabolism. 2016;18(8):812‐9.

64. Wanner C, Inzucchi SE, Lachin JM, Fitchett D, von Eynatten M, Mattheus M, et al. Empagliflozin and Progression of Kidney Disease in Type 2 Diabetes. The New England journal of medicine. 2016;375(4):323-34.

65. Weber MA, Mansfield TA, Alessi F, Iqbal N, Parikh S, Ptaszynska A. Effects of dapagliflozin on blood pressure in hypertensive diabetic patients on renin-angiotensin system blockade. Blood pressure. 2016;25(2):93‐103.

66. Weber MA, Mansfield TA, Cain VA, Iqbal N, Parikh S, Ptaszynska A. Blood pressure and glycaemic effects of dapagliflozin versus placebo in patients with type 2 diabetes on combination antihypertensive therapy: a randomised, double-blind, placebo-controlled, phase 3 study. Lancet Diabetes Endocrinol. 2016;4(3):211-20.

67. Yang W, Han P, Min KW, Wang B, Mansfield T, T'Joen C, et al. Efficacy and safety of dapagliflozin in Asian patients with type 2 diabetes after metformin failure: a randomized controlled trial. Journal of diabetes. 2016;8(6):796‐808.

68. Kadowaki T, Inagaki N, Kondo K, Nishimura K, Kaneko G, Maruyama N, et al. Efficacy and safety of canagliflozin as add-on therapy to teneligliptin in Japanese patients with type 2 diabetes mellitus: results of a 24-week, randomized, double-blind, placebo-controlled trial. Diabetes, obesity & metabolism. 2017;19(6):874‐82.

69. Søfteland E, Meier JJ, Vangen B, Toorawa R, Maldonado-Lutomirsky M, Broedl UC. Empagliflozin as Add-on Therapy in Patients With Type 2 Diabetes Inadequately Controlled With Linagliptin and Metformin: a 24-Week Randomized, Double-Blind, Parallel-Group Trial. Diabetes care. 2017;40(2):201‐9.

70. Terauchi Y, Tamura M, Senda M, Gunji R, Kaku K. Efficacy and safety of tofogliflozin in Japanese patients with type 2 diabetes mellitus with inadequate glycaemic control on insulin therapy (J-STEP/INS): results of a 16-week randomized, double-blind, placebo-controlled multicentre trial. Diabetes, obesity & metabolism. 2017;19(10):1397‐407.

71. Terra SG, Focht K, Davies M, Frias J, Derosa G, Darekar A, et al. Phase III, efficacy and safety study of ertugliflozin monotherapy in people with type 2 diabetes mellitus inadequately controlled with diet and exercise alone. Diabetes, obesity & metabolism. 2017;19(5):721‐8.

72. Fioretto P, Del Prato S, Goldenberg R, Giorgino F, Reyner D, Langkilde AM, et al. Eficacy and safety of dapagliflozin in patients with type 2 diabetes and moderate renal impairment (chronic kidney disease Stage 3A): The DERIVE Study. Endocrine Reviews. 2018;39(2).

73. Grunberger G, Camp S, Johnson J, Huyck S, Terra SG, Mancuso JP, et al. Ertugliflozin in Patients with Stage 3 Chronic Kidney Disease and Type 2 Diabetes Mellitus: the VERTIS RENAL Randomized Study. Diabetes therapy. 2018;9(1):49‐66.

74. Han KA, Chon S, Chung CH, Lim S, Lee KW, Baik S, et al. Efficacy and safety of ipragliflozin as an add-on therapy to sitagliptin and metformin in Korean patients with inadequately controlled type 2 diabetes mellitus: A randomized controlled trial. Diabetes Obes Metab. 2018;20(10):2408-15.

75. Kawamori R, Haneda M, Suzaki K, Cheng G, Shiki K, Miyamoto Y, et al. Empagliflozin as add-on to linagliptin in a fixed-dose combination in Japanese patients with type 2 diabetes: glycaemic efficacy and safety profile in a 52-week, randomized, placebo-controlled trial. Diabetes, obesity & metabolism. 2018;20(9):2200‐9.

76. Rosenstock J, Frias J, Pall D, Charbonnel B, Pascu R, Saur D, et al. Effect of ertugliflozin on glucose control, body weight, blood pressure and bone density in type 2 diabetes mellitus inadequately controlled on metformin monotherapy (VERTIS MET). Diabetes, obesity & metabolism. 2018;20(3):520‐9.

77. Seino Y, Sasaki T, Fukatsu A, Imazeki H, Ochiai H, Sakai S. Efficacy and safety of luseogliflozin added to insulin therapy in Japanese patients with type 2 diabetes: a multicenter, 52-week, clinical study with a 16-week, double-blind period and a 36-week, open-label period. Current medical research and opinion. 2018;34(6):981‐94.

78. Shestakova MV, Wilding JPH, Wilpshaar W, Tretter R, Orlova VL, Verbovoy AF. A phase 3 randomized placebo-controlled trial to assess the efficacy and safety of ipragliflozin as an add-on therapy to metformin in Russian patients with inadequately controlled type 2 diabetes mellitus. Diabetes research and clinical practice. 2018;146:240‐50.

79. Yang W, Ma J, Li Y, Zhou Z, Kim JH, Zhao J, et al. Dapagliflozin as add-on therapy in Asian patients with type 2 diabetes inadequately controlled on insulin with or without oral antihyperglycemic drugs: a randomized controlled trial. Journal of diabetes. 2018;(no pagination).

80. Ferdinand KC, Izzo JL, Lee J, Meng L, George J, Salsali A, et al. Antihyperglycemic and Blood Pressure Effects of Empagliflozin in Black Patients With Type 2 Diabetes Mellitus and Hypertension. Circulation. 2019;139(18):2098‐109.

81. Ji L, Liu Y, Miao H, Xie Y, Yang M, Wang W, et al. Safety and efficacy of ertugliflozin in Asian patients with type 2 diabetes mellitus inadequately controlled with metformin monotherapy: VERTIS Asia. Diabetes Obes Metab. 2019;21(6):1474-82.

82. Perkovic V, Jardine MJ, Neal B, Bompoint S, Heerspink HJL, Charytan DM, et al. Canagliflozin and Renal Outcomes in Type 2 Diabetes and Nephropathy. The New England journal of medicine. 2019;380(24):2295-306.

83. Pollock C, Stefánsson B, Reyner D, Rossing P, Sjöström CD, Wheeler DC, et al. Albuminuria-lowering effect of dapagliflozin alone and in combination with saxagliptin and effect of dapagliflozin and saxagliptin on glycaemic control in patients with type 2 diabetes and chronic kidney disease (DELIGHT): a randomised, double-blind, placebo-controlled trial. The lancet Diabetes & endocrinology. 2019;7(6):429‐41.

84. Wiviott SD, Raz I, Bonaca MP, Mosenzon O, Kato ET, Cahn A, et al. Dapagliflozin and Cardiovascular Outcomes in Type 2 Diabetes. The New England journal of medicine. 2019;380(4):347-57.

85. Cannon CP, Pratley R, Dagogo-Jack S, Mancuso J, Huyck S, Masiukiewicz U, et al. Cardiovascular Outcomes with Ertugliflozin in Type 2 Diabetes. New England journal of medicine. 2020;383(15):1425‐35.

86. Dharmalingam M, Aravind SR, Thacker H, Paramesh S, Mohan B, Chawla M, et al. Efficacy and Safety of Remogliflozin Etabonate, a New Sodium Glucose Co-Transporter-2 Inhibitor, in Patients with Type 2 Diabetes Mellitus: a 24-Week, Randomized, Double-Blind, Active-Controlled Trial. Drugs. 2020;80(6):587‐600.

87. Sone H, Kaneko T, Shiki K, Tachibana Y, Pfarr E, Lee J, et al. Efficacy and safety of empagliflozin as add-on to insulin in Japanese patients with type 2 diabetes: a randomized, double-blind, placebo-controlled trial. Diabetes, obesity & metabolism. 2020;22(3):417‐26.

88. Babar M, Hussain M, Ahmad M, Akhtar L. Comparison Of Efficacy And Safety Profile Of Empagliflozin As A Combination Therapy In Obese Type 2 Diabetic Patients. Journal of Ayub Medical College, Abbottabad. 2021;33(2):188‐91.

89. Bhatt DL, Szarek M, Pitt B, Cannon CP, Leiter LA, McGuire DK, et al. Sotagliflozin in patients with diabetes and chronic kidney disease. New England Journal of Medicine. 2021;384(2):129-39.

90. Cherney DZI, Ferrannini E, Umpierrez GE, Peters AL, Rosenstock J, Carroll AK, et al. Efficacy and safety of sotagliflozin in patients with type 2 diabetes and severe renal impairment. Diabetes Obes Metab. 2021;23(12):2632-42.

91. Dagogo-Jack S, Pratley RE, Cherney DZI, McGuire DK, Cosentino F, Shih WJ, et al. Glycemic efficacy and safety of the SGLT2 inhibitor ertugliflozin in patients with type 2 diabetes and stage 3 chronic kidney disease: an analysis from the VERTIS CV randomized trial. BMJ open diabetes research & care. 2021;9(1).

92. Hussain M, Atif M, Babar M, Akhtar L. Comparison Of Efficacy And Safety Profile Of Empagliflozin Versus Dapagliflozin As Add On Therapy In Type 2 Diabetic Patients. Journal of Ayub Medical College, Abbottabad. 2021;33(4):593‐7.

93. Kaku K, Kadowaki T, Seino Y, Okamoto T, Shirakawa M, Sato A, et al. Efficacy and safety of ipragliflozin in Japanese patients with type 2 diabetes and inadequate glycaemic control on sitagliptin. Diabetes Obes Metab. 2021;23(9):2099-108.

94. Bailey CJ, Gross JL, Hennicken D, Iqbal N, Mansfield TA, List JF. Dapagliflozin add-on to metformin in type 2 diabetes inadequately controlled with metformin: a randomized, double-blind, placebo-controlled 102-week trial. BMC medicine. 2013;11:43.

95. Strojek K, Yoon KH, Hruba V, Sugg J, Langkilde AM, Parikh S. Dapagliflozin Added to Glimepiride in Patients with Type 2 Diabetes Mellitus Sustains Glycemic Control and Weight Loss Over 48 Weeks: a Randomized, Double-Blind, Parallel-Group, Placebo-Controlled Trial. Diabetes therapy. 2014;5(1):267‐83.

96. Wilding JPH, Woo V, Rohwedder K, Sugg J, Parikh S. Dapagliflozin in patients with type 2 diabetes receiving high doses of insulin: Efficacy and safety over 2 years. Diabetes, Obesity and Metabolism. 2014;16(2):124-36.

97. Bode B, Stenlöf K, Harris S, Sullivan D, Fung A, Usiskin K, et al. Long-term efficacy and safety of canagliflozin over 104 weeks in patients aged 55-80 years with type 2 diabetes. Diabetes, obesity & metabolism. 2015;17(3):294‐303.

98. Haering HU, Merker L, Christiansen AV, Roux F, Salsali A, Kim G, et al. Empagliflozin as add-on to metformin plus sulphonylurea in patients with type 2 diabetes. Diabetes Res Clin Pract. 2015;110(1):82-90.

99. Kovacs CS, Seshiah V, Merker L, Christiansen AV, Roux F, Salsali A, et al. Empagliflozin as Add-on Therapy to Pioglitazone With or Without Metformin in Patients With Type 2 Diabetes Mellitus. Clin Ther. 2015;37(8):1773-88.e1.

100. Roden M, Merker L, Christiansen AV, Roux F, Salsali A, Kim G, et al. Safety, tolerability and effects on cardiometabolic risk factors of empagliflozin monotherapy in drug-naïve patients with type 2 diabetes: a double-blind extension of a Phase III randomized controlled trial. Cardiovascular diabetology. 2015;14:154.

101. Jabbour SA, Frías JP, Ahmed A, Hardy E, Choi J, Sjöström CD, et al. Efficacy and Safety Over 2 Years of Exenatide Plus Dapagliflozin in the DURATION-8 Study: a Multicenter, Double-Blind, Phase 3, Randomized Controlled Trial. Diabetes care. 2020;43(10):2528‐36.

102. ClinicalTrials.gov, Efficacy and Safety of Sotagliflozin Versus Placebo in Patients With Type 2 Diabetes Mellitus Not Currently Treated With Antidiabetic Therapy, https://clinicaltrials.gov/ct2/show/NCT02926937?id=NCT02926937&draw=2&rank=1&load=cart(accessed 29 Dec 2022).

103. ClinicalTrials.gov, Efficacy and Bone Safety of Sotagliflozin 400 and 200 mg Versus Placebo in Participants With Type 2 Diabetes Mellitus Who Have Inadequate Glycemic Control (SOTA-BONE), https://clinicaltrials.gov/ct2/show/NCT03386344?term=NCT03386344&draw=2&rank=1(accessed 29 Dec 2022).

104. ClinicalTrials.gov, Efficacy and Safety of Sotagliflozin Versus Placebo in Patients With Type 2 Diabetes Mellitus on Background of Metformin, https://clinicaltrials.gov/ct2/show/NCT02926950?term=NCT02926950&draw=1&rank=1(accessed 29 Dec 2022).

105. ClinicalTrials.gov, Efficacy and Safety of Sotagliflozin Versus Placebo and Empagliflozin in Participants With Type 2 Diabetes Mellitus Who Have Inadequate Glycemic Control While Taking a DPP4 Inhibitor Alone or With Metformin (SOTA-EMPA), https://clinicaltrials.gov/ct2/show/NCT03351478?term=NCT03351478&draw=2&rank=1(accessed 29 Dec 2022).

106. ClinicalTrials.gov, Efficacy and Safety of Sotagliflozin Versus Placebo in Participants With Type 2 Diabetes Mellitus Who Have Inadequate Glycemic Control While Taking Insulin Alone or With Other Oral Antidiabetic Agents (SOTA-INS), https://clinicaltrials.gov/ct2/show/NCT03285594?term=NCT03285594&draw=2&rank=1(accessed 29 Dec 2022).

107. ClinicalTrials.gov, Efficacy and Safety of Sotagliflozin Versus Placebo in Participants With Type 2 Diabetes Mellitus on Background of Sulfonylurea Alone or With Metformin, https://clinicaltrials.gov/ct2/show/NCT03066830?term=NCT03066830&draw=2&rank=1(accessed 29 Dec 2022).

108. ClinicalTrials.gov, Efficacy and Safety of Sotagliflozin Versus Glimepiride and Placebo in Participants With Type 2 Diabetes Mellitus That Are Taking Metformin Monotherapy (SOTA-GLIM), https://clinicaltrials.gov/ct2/show/NCT03332771?term=NCT03332771&draw=1&rank=1(accessed 29 Dec 2022).

109. ClinicalTrials.gov, A 16 Weeks Study on Efficacy and Safety of Two Doses of Empagliflozin (BI 10773) (Once Daily Versus Twice Daily) in Patients With Type 2 Diabetes Mellitus and Preexisting Metformin Therapy, https://clinicaltrials.gov/ct2/show/NCT01649297?term=NCT01649297&draw=2&rank=1(accessed 29 Dec 2022).

110. ClinicalTrials.gov, A Study of the Effects of Canagliflozin (JNJ-28431754) on Renal Endpoints in Adult Participants With Type 2 Diabetes Mellitus (CANVAS-R), https://clinicaltrials.gov/ct2/show/NCT01989754?term=NCT01989754&draw=2&rank=1(accessed 29 Dec 2022).

111. ClinicalTrials.gov, CANVAS - CANagliflozin cardioVascular Assessment Study (CANVAS), https://clinicaltrials.gov/ct2/show/NCT01032629?term=NCT01032629&draw=1&rank=1(accessed 29 Dec 2022).

112. ClinicalTrials.gov, Dapagliflozin Effects on Epicardial Fat, https://clinicaltrials.gov/ct2/show/NCT02235298?term=NCT02235298&draw=2&rank=1(accessed 29 Dec 2022).

113. ClinicalTrials.gov, Safety and Efficacy Study of Sotagliflozin on Glucose Control in Participants With Type 2 Diabetes, Moderate Impairment of Kidney Function, and Inadequate Blood Sugar Control (SOTA-CKD3), https://clinicaltrials.gov/ct2/show/NCT03242252?term=NCT03242252&draw=2&rank=1(accessed 29 Dec 2022).

Appendix 4: Characteristics of Included Studies

| **Study** | **Trial registration** | **Duration**  **of follow‐up** | **Country** | **Setting** | **The oGLD** | **Randomised**  **treatments + dose** | **No of**  **patients**  **randomized** | **Mean**  **age(years)** | **Mean**  **HbA1c**  **(%)** | **Proportion**  **of female**  **participants(%)** | **Mean**  **BMI**  **(kg/m^2^)** | **Mean**  **diabetes**  **duration(years)** |
| --- | --- | --- | --- | --- | --- | --- | --- | --- | --- | --- | --- | --- |
| Kaku K 2014 | Japic CTI-101349 | 24w | Japan | T2D | None | Tofogliflozin 20mg QD | 58 | 56.6 | 8.34 | 32.8 | 24.99 | 6.4 |
|  |  |  |  |  |  | Tofogliflozin 40mg QD | 58 | 57 | 8.37 | 32.8 | 25.78 | 6.7 |
|  |  |  |  |  |  | Placebo | 56 | 56.8 | 8.41 | 33.9 | 26 | 6 |
| Ikeda S 2015 | NCT00800176 | 12w | Multinational | T2D | MET | Tofogliflozin 20mg QD | 64 | 56.3 | 7.92 | 32.8 | 30.09 | 5.21 |
|  |  |  |  |  |  | Tofogliflozin 40mg QD | 67 | 57.5 | 7.92 | 53.7 | 30.36 | 6.44 |
|  |  |  |  |  |  | Placebo | 66 | 53.9 | 7.88 | 45.5 | 30.37 | 5.98 |
| Terauchi Y 2017 | NCT02201004 | 16w | Japan | T2D | Ins±DPP-4i | Tofogliflozin 20mg QD | 140 | 59.1 | 8.53 | 36.2 | 25.8 | 15.02 |
|  |  |  |  |  |  | Placebo | 70 | 56.4 | 8.4 | 31.4 | 26.9 | 12.39 |
| Seino Y2014Ⅰ | JapicCTI-090908 | 12w | Japan | T2D | None | Luseogliflozin 2.5mg QD | 61 | 58.3 | 8.07 | 42.6 | 24.8 | 6.15 |
|  |  |  |  |  |  | Luseogliflozin 5mg QD | 61 | 56.8 | 8.16 | 27.9 | 24.5 | 5.77 |
|  |  |  |  |  |  | Placebo | 54 | 57.6 | 7.88 | 25.9 | 25.2 | 7.3 |
| Seino Y2014Ⅱ | Japic CTI-101191 | 12w | Japan | T2D | None | Luseogliflozin 2.5mg QD | 56 | 57.4 | 8.05 | 32.1 | 24.79 | 4.6 |
|  |  |  |  |  |  | Luseogliflozin 5mg QD | 54 | 57.3 | 7.86 | 24.1 | 26.43 | 4.5 |
|  |  |  |  |  |  | Luseogliflozin 10mg QD | 58 | 59.6 | 7.95 | 36.2 | 23.36 | 6.2 |
|  |  |  |  |  |  | Placebo | 57 | 57.1 | 7.92 | 28.1 | 25.15 | 5.1 |
| Seino Y 2018 | JapicCTI-142582 | 16w | Japan | T2D | Ins | Luseogliflozin 2.5mg QD | 159 | 57.4 | 8.7 | 29.6 | 25.42 | 11.7 |
|  |  |  |  |  |  | Placebo | 74 | 57.1 | 8.84 | 31.3 | 25.15 | 12.1 |
| Seino Y 2014Ⅲ | JapicCTI111661 | 24w | Japan | T2D | None | Luseogliflozin 2.5mg QD | 79 | 58.9 | 8.14 | 24.1 | 25.98 | 6.5 |
|  |  |  |  |  |  | Placebo | 79 | 59.6 | 8.17 | 29.1 | 25.34 | 6.1 |
| Amin NB 2015 | NCT01059825 | 12w | Multinational | T2D | MET | Ertugliflozin 5mg QD | 55 | 54.7 | 7.88 | 25.5 | 30.7 | 6.7 |
|  |  |  |  |  |  | Ertugliflozin 10mg QD | 55 | 57.3 | 8.13 | 43.6 | 30.7 | 6.1 |
|  |  |  |  |  |  | Ertugliflozin 25mg QD | 55 | 54.2 | 8.3 | 32.7 | 29.8 | 6 |
|  |  |  |  |  |  | Placebo | 54 | 54 | 8.08 | 44.4 | 30.6 | 6.4 |
| Terra SG 2017 | NCT01958671 | 26w | Multinational | T2D | None | Ertugliflozin 5mg QD | 156 | 56.8 | 8.16 | 42.9 | 33.2 | 5.11 |
|  |  |  |  |  |  | Ertugliflozin 15 mg QD | 152 | 56.2 | 8.35 | 40.8 | 32.5 | 5.22 |
|  |  |  |  |  |  | Placebo | 153 | 56.1 | 8.11 | 46.4 | 33.3 | 4.63 |
| Dagogo-Jack S 2018Ⅰ | NCT02036515 | 26w | Multinational | T2D | MET+Sitagliptin | Ertugliflozin 5mg QD | 156 | 59.2 | 8.1 | 48.1 | 31.2 | 9.9 |
|  |  |  |  |  |  | Ertugliflozin 15 mg QD | 153 | 59.7 | 8 | 46.4 | 30.9 | 9.2 |
|  |  |  |  |  |  | Placebo | 153 | 58.3 | 8 | 34.6 | 30.3 | 9.4 |
| Rosenstock J 2018 | NCT02033889 | 26w | Multinational | T2D | MET | Ertugliflozin 5mg QD | 207 | 56.6 | 8.1 | 52.1 | 30.8 | 7.9 |
|  |  |  |  |  |  | Ertugliflozin 15 mg QD | 205 | 56.9 | 8.1 | 54.5 | 31.1 | 8.1 |
|  |  |  |  |  |  | Placebo | 209 | 56.5 | 8.2 | 52.1 | 30.7 | 8.0 |
| Ji LN 2019 | NCT02630706 | 26w | Asian | T2D | MET | Ertugliflozin 5mg QD | 170 | 56.1 | 8.1 | 44.1 | 26.0 | 7.0 |
|  |  |  |  |  |  | Ertugliflozin 15 mg QD | 169 | 56.3 | 8.1 | 42 | 25.7 | 7.5 |
|  |  |  |  |  |  | Placebo | 167 | 56.9 | 8.1 | 47.3 | 26.1 | 6.4 |
| Cannon CP 2020 | NCT01986881 | 26w | Multinational | T2D+CVD | None/oGLD | Ertugliflozin 5/15mg QD | 5499 | 64.4 | 8.2 | 30.7 | 32.0 | 12.9 |
|  |  |  |  |  |  | Placebo | 2747 | 64.4 | 8.2 | 29.7 | 31.9 | 13.1 |
| LIST JF 2009 | NCT00263276 | 12w | Multinational | T2D | None | Dapagliflozin 5mg QD | 58 | 55 | 8.0 | 52 | 32 | NA |
|  |  |  |  |  |  | Dapagliflozin 10 mg QD | 47 | 55 | 8.0 | 47 | 31 | NA |
|  |  |  |  |  |  | Dapagliflozin 20mg QD | 59 | 55 | 7.7 | 46 | 31 | NA |
|  |  |  |  |  |  | Dapagliflozin 50mg QD | 56 | 53 | 7.8 | 55 | 32 | NA |
|  |  |  |  |  |  | Placebo | 54 | 53 | 7.9 | 44 | 32 | NA |
| Rosenstock J 2012 | NCT00642278 | 12w | Multinational | T2D | MET | Canagliflozin 100mg QD | 64 | 51.7 | 7.83 | 44 | 31.7 | 6.1 |
|  |  |  |  |  |  | Canagliflozin 200mg QD | 65 | 52.9 | 7.61 | 49 | 31.4 | 6.4 |
|  |  |  |  |  |  | Canagliflozin 300mg QD | 64 | 52.3 | 7.69 | 44 | 31.6 | 5.9 |
|  |  |  |  |  |  | Canagliflozin 300mg BID | 64 | 55.2 | 7.73 | 56 | 31.8 | 5.8 |
|  |  |  |  |  |  | Placebo | 65 | 53.3 | 7.75 | 52 | 30.6 | 6.4 |
| Inagaki N 2013 | NCT01022112 | 12w | Japan | T2D | None | Canagliflozin 100mg QD | 74 | 57.7 | 8.05 | 29.3 | 25.61 | NA |
|  |  |  |  |  |  | Canagliflozin 200mg QD | 76 | 57.0 | 8.11 | 45.5 | 25.51 | NA |
|  |  |  |  |  |  | Canagliflozin 300mg QD | 75 | 57.1 | 8.17 | 26.7 | 25.89 | NA |
|  |  |  |  |  |  | Placebo | 75 | 57.7 | 7.99 | 28 | 26.41 | NA |
| Bode B 2013 | NCT01106651 | 26w | Multinational | T2D(55-80years) | None/oGLD | Canagliflozin 100mg QD | 241 | 64.3 | 7.8 | 48.5 | 31.4 | 12.3 |
|  |  |  |  |  |  | Canagliflozin 300mg QD | 236 | 63.4 | 7.7 | 45.3 | 31.5 | 11.3 |
|  |  |  |  |  |  | Placebo | 237 | 63.2 | 7.8 | 39.7 | 31.8 | 11.4 |
| Stenlöf K 2013 | NCT01081834 | 26w | Multinational | T2D | None | Canagliflozin 100mg QD | 195 | 55.1 | 8.1 | 58.5 | 31.3 | 4.5 |
|  |  |  |  |  |  | Canagliflozin 300mg QD | 197 | 55.3 | 8.0 | 58.4 | 31.7 | 4.3 |
|  |  |  |  |  |  | Placebo | 192 | 55.7 | 8.0 | 54.2 | 31.8 | 4.2 |
| Wilding JPH 2013 | NCT01106625 | 26w | Multinational | T2D | MET+SU | Canagliflozin 100mg QD | 157 | 57.4 | 8.1 | 51.6 | 33.3 | 9.1 |
|  |  |  |  |  |  | Canagliflozin 300mg QD | 156 | 56.1 | 8.1 | 44.2 | 33.2 | 9.4 |
|  |  |  |  |  |  | Placebo | 156 | 56.8 | 8.1 | 51.3 | 32.7 | 10.3 |
| Inagaki N 2014 | NCT014 13204 | 24w | Japan | T2D | None | Canagliflozin 100mg QD | 90 | 58.4 | 7.98 | 35.4 | 25.59 | 4.72 |
|  |  |  |  |  |  | Canagliflozin 200mg QD | 88 | 57.4 | 8.04 | 18.2 | 25.43 | 5.88 |
|  |  |  |  |  |  | Placebo | 93 | 58.2 | 8.04 | 35.4 | 25.85 | 5.63 |
| Qiu R 2014 | NCT01340664 | 18w | Multinational | T2D | MET | Canagliflozin 50mg BID | 93 | 58.6 | 7.6 | 57 | 33 | 6.7 |
|  |  |  |  |  |  | Canagliflozin 150mg BID | 93 | 56.7 | 7.6 | 52.7 | 32.3 | 7.3 |
|  |  |  |  |  |  | Placebo | 93 | 57.0 | 7.7 | 50.5 | 32.3 | 7 |
| Ji LN 2015 | NCT01381900 | 18w | Asian | T2D | MET±SU | Canagliflozin 100mg QD | 223 | 56.5 | 8 | 44.4 | 25.6 | 6.8 |
|  |  |  |  |  |  | Canagliflozin 300mg QD | 227 | 56.4 | 8 | 50.2 | 26 | 6.9 |
|  |  |  |  |  |  | Placebo | 226 | 55.8 | 7.9 | 44.7 | 25.5 | 6.4 |
| Inagaki N 2016 | NCT02220920 | 16w | Japan | T2D | Ins | Canagliflozin 100mg QD | 76 | 59.7 | 8.89 | 42.1 | 26.88 | 15.18 |
|  |  |  |  |  |  | Placebo | 70 | 56.1 | 8.85 | 30 | 25.99 | 12.34 |
| Kadowaki T 2017 | NCT02354235 | 24w | Japan | T2D | Teneligliptin | Canagliflozin 100mg QD | 70 | 58.4 | 8.18 | 22.9 | 25.53 | 8.34 |
|  |  |  |  |  |  | Placebo | 68 | 56.0 | 7.87 | 22.1 | 26.44 | 6.5 |
| Bailey CJ 2010 | NCT00528879 | 24w | Multinational | T2D | MET | Dapagliflozin 5mg QD | 137 | 54.3 | 8.17 | 50 | 31.4 | 6.4 |
|  |  |  |  |  |  | Dapagliflozin 10 mg QD | 135 | 52.7 | 7.92 | 43 | 31.2 | 6.1 |
|  |  |  |  |  |  | Placebo | 137 | 53.7 | 8.11 | 45 | 31.8 | 5.8 |
| Ferranninie E 2010 | NCT00528372 | 24w | Multinational | T2D | None | Dapagliflozin 2.5mg BID | 65 | 53.0 | 7.92 | 44.6 | 32.6 | 0.5 |
|  |  |  |  |  |  | Dapagliflozin 5mg BID | 64 | 52.6 | 7.86 | 51.6 | 31.9 | 0.25 |
|  |  |  |  |  |  | Dapagliflozin 10mg BID | 70 | 50.6 | 8.01 | 51.4 | 33.6 | 0.45 |
|  |  |  |  |  |  | Placebo | 75 | 52.7 | 7.84 | 58.7 | 32.3 | 0.5 |
| Strojek K 2011 | NCT00680745 | 24w | Multinational | T2D | Glimepiride | Dapagliflozin 5mg QD | 142 | 60.2 | 8.12 | 50 | NA | 7.4 |
|  |  |  |  |  |  | Dapagliflozin 10 mg QD | 151 | 58.9 | 8.07 | 56.3 | NA | 7.2 |
|  |  |  |  |  |  | Placebo | 145 | 60.3 | 8.15 | 51 | NA | 7.4 |
| Bailey CJ 2012 | NA | 24w | Multinational | T2D | None | Dapagliflozin 5mg QD | 68 | 51.3 | 7.9 | 52.9 | 30.97 | 0.3 |
|  |  |  |  |  |  | Placebo | 68 | 53.5 | 7.8 | 45.6 | 32.47 | 0.4 |
| Bolinder J 2012 | NCT00855166 | 24w | Multinational | T2D | MET | Dapagliflozin 10 mg QD | 89 | 60.6 | 7.19 | 44.9 | 32.1 | 6.0 |
|  |  |  |  |  |  | Placebo | 91 | 60.8 | 7.16 | 44 | 31.7 | 5.5 |
| Rosenstock J 2012 | NCT00642278 | 48w | Multinational | T2D | PIO | Dapagliflozin 5mg QD | 141 | 53.2 | 8.4 | 57.9 | NA | 5.64 |
|  |  |  |  |  |  | Dapagliflozin 10 mg QD | 140 | 53.8 | 8.37 | 44.7 | NA | 5.75 |
|  |  |  |  |  |  | Placebo | 139 | 53.5 | 8.34 | 48.9 | NA | 5.07 |
| Wilding JPH 2012 | NCT00673231 | 24w | Multinational | T2D | Ins±oGLD | Dapagliflozin 5mg QD | 211 | 59.3 | 8.62 | 52.6 | 33 | 13.1 |
|  |  |  |  |  |  | Dapagliflozin 10 mg QD | 194 | 59.3 | 8.57 | 55.2 | 33.4 | 14.2 |
|  |  |  |  |  |  | Placebo | 193 | 58.8 | 8.47 | 50.8 | 33.1 | 13.5 |
| Kaku K 2013 | NCT00972244 | 12w | Japan | T2D | None | Dapagliflozin 5mg QD | 58 | 58 | 8.05 | 19 | NA | 4.34 |
|  |  |  |  |  |  | Dapagliflozin 10 mg QD | 52 | 56.5 | 8.18 | 25 | NA | 4.37 |
|  |  |  |  |  |  | Placebo | 54 | 58.4 | 8.12 | 20.4 | NA | 4.74 |
| Ji LN 2014 | NCT01095653 | 24w | Asian | T2D | None | Dapagliflozin 5mg QD | 128 | 53 | 8.14 | 34.4 | 25.17 | 1.15 |
|  |  |  |  |  |  | Dapagliflozin 10 mg QD | 133 | 51.2 | 8.28 | 35.3 | 25.76 | 1.67 |
|  |  |  |  |  |  | Placebo | 132 | 49.9 | 8.35 | 34.1 | 25.93 | 1.3 |
| Leiter LA 2014 | NCT01042977 | 28w | Multinational | T2D+CVD | None/oGLD | Dapagliflozin 10 mg QD | 482 | 63.9 | 8 | 33.1 | 33 | 13.5 |
|  |  |  |  |  |  | Placebo | 483 | 63.6 | 8.1 | 33.0 | 32.7 | 13 |
|  |  |  |  | T2D+CVD(age＜65) | None/oGLD | Dapagliflozin 10 mg QD | 253 | 58.0 | 8.1 | 32.8 | 33.5 | 12 |
|  |  |  |  |  |  | Placebo | 258 | 58.3 | 8.1 | 31.8 | 33.2 | 11.7 |
|  |  |  |  | T2D+CVD(age≥65) | None/oGLD | Dapagliflozin 10 mg QD | 227 | 70.5 | 8 | 33.5 | 32.5 | 15.2 |
|  |  |  |  |  |  | Placebo | 224 | 69.7 | 8 | 34.4 | 32.1 | 14.5 |
| Bailey CJ 2015 | NCT00528372 | 102w | Canada | T2D | MET | Dapagliflozin 5mg QD | 64 | 52.6 | 7.86 | 51.6 | NA | 1.0 |
|  |  |  |  |  |  | Dapagliflozin 10 mg QD | 70 | 50.6 | 8.01 | 51.4 | NA | 2.3 |
|  |  |  |  |  |  | Placebo | 75 | 52.7 | 7.84 | 58.7 | NA | 2.1 |
| Cefalu WT 2015 | NCT01031680 | 52w | Multinational | T2D+CVD+hypertension | Ins | Dapagliflozin 10 mg QD | 455 | 62.8 | 8.18 | 32.1 | 32.6 | 12.6 |
|  |  |  |  |  |  | Placebo | 159 | 63.0 | 8.08 | 31.4 | 32.9 | 12.3 |
| Mathieu C 2015 | NCT01646320 | 24w | Multinational | T2D | MET+Saxagliptin | Dapagliflozin 10 mg QD | 160 | 55.2 | 8.24 | 56.3 | 31.2 | 7.2 |
|  |  |  |  |  |  | Placebo | 160 | 55.0 | 8.17 | 56.2 | 32.2 | 8.0 |
| Matthaei S 2015 | NCT01392677 | 24w | Multinational | T2D | MET+SU | Dapagliflozin 10 mg QD | 108 | 61.1 | 8.08 | 57.4 | 31.9 | 9.3 |
|  |  |  |  |  |  | Placebo | 108 | 60.9 | 8.24 | 44.4 | 32.0 | 9.6 |
| Schumm-Draeger PM 2015 | NCT01217892 | 16w | Multinational | T2D | MET | Dapagliflozin 2.5mg BID | 100 | 58.3 | 7.77 | 63 | 33.16 | 4.8 |
|  |  |  |  |  |  | Dapagliflozin 5mg BID | 99 | 55.3 | 7.78 | 53.5 | 33.09 | 5.12 |
|  |  |  |  |  |  | Dapagliflozin 10 mg QD | 99 | 58.5 | 7.71 | 50.5 | 33.25 | 5.45 |
|  |  |  |  |  |  | Placebo | 101 | 58.5 | 7.94 | 53.5 | 31.74 | 5.53 |
| Araki E 2016 | NCT02157298 | 16w | Japan | T2D | Ins | Dapagliflozin 5mg QD | 122 | 58.3 | 8.26 | 27 | 26.89 | 15.32 |
|  |  |  |  |  |  | Placebo | 60 | 57.6 | 8.52 | 33.3 | 26.12 | 14.24 |
| Michael A 2016 | NCT01137474 | 12w | Multinational | T2D | oGLD | Dapagliflozin 10 mg QD | 302 | 55.6 | 8.1 | 40.7 | NA | 8.2 |
|  |  |  |  |  |  | Placebo | 311 | 56.2 | 8 | 45 | NA | 7.6 |
| Weber MA 2016 | NCT01195662 | 12w | Multinational | T2D + hypertension | oGLD±Ins | Dapagliflozin 10 mg QD | 225 | 56 | 8.1 | 48 | NA | 7.7 |
|  |  |  |  |  |  | Placebo | 224 | 57 | 8 | 42 | NA | 7.3 |
| Yang WY 2016 | NCT01095666 | 24w | Asian | T2D | MET | Dapagliflozin 5mg QD | 147 | 53.1 | 8.09 | 40.7 | 25.7 | 4.2 |
|  |  |  |  |  |  | Dapagliflozin 10 mg QD | 152 | 54.6 | 8.17 | 42.1 | 26.2 | 5.3 |
|  |  |  |  |  |  | Placebo | 145 | 53.5 | 8.13 | 40.7 | 26.4 | 5.3 |
| Yang WY 2018 | NCT02096705 | 24w | Asian | T2D | Ins±oGLD | Dapagliflozin 10 mg QD | 139 | 56.5 | 8.52 | 52.2 | 26.4 | 12.7 |
|  |  |  |  |  |  | Placebo | 133 | 58.6 | 8.58 | 51.9 | 26.7 | 12.2 |
| Wiviott SD 2019 | NCT01730534 | 216w | Multinational | T2D+CVD | oGLD | Dapagliflozin 10 mg QD | 8582 | 64 | 8.3 | 37.9 | 32 | 11 |
|  |  |  |  |  |  | Placebo | 8578 | 63.9 | 8.3 | 36.9 | 32.1 | 10 |
| Fr´ıas JP 2016 | NCT02229396 | 28w | Multinational | T2D | Exenatide+MET | Dapagliflozin 10 mg QD | 231 | 54 | 7.21 | 49 | 32 | 7.6 |
|  |  |  |  |  |  | Placebo | 230 | 54 | 6.87 | 55 | 33.2 | 7.4 |
| Ferrannini E 2013 | NCT00789035 | 12w | Multinational | T2D | None | Empagliflozin 10mg QD | 81 | 58 | 8 | 50.6 | 28.1 | NA |
|  |  |  |  |  |  | Empagliflozin 25mg QD | 82 | 57 | 7.8 | 50 | 28.3 | NA |
|  |  |  |  |  |  | Placebo | 82 | 58 | 7.8 | 45.1 | 28.8 | NA |
| Haring HU 2013 | NCT01159600 | 24w | Multinational | T2D | MET+SU | Empagliflozin 10mg QD | 225 | 57.4 | 8.07 | 50 | 28.3 | NA |
|  |  |  |  |  |  | Empagliflozin 25mg QD | 216 | 57 | 8.1 | 47 | 28.3 | NA |
|  |  |  |  |  |  | Placebo | 225 | 56.9 | 8.15 | 50 | 27.9 | NA |
| Häring HU 2014 | NCT01159600 | 24w | Multinational | T2D | MET | Empagliflozin 10mg QD | 217 | 55.5 | 7.94 | 42 | 29.1 | NA |
|  |  |  |  |  |  | Empagliflozin 25mg QD | 213 | 55.6 | 7.86 | 44 | 29.7 | NA |
|  |  |  |  |  |  | Placebo | 207 | 56 | 7.9 | 44 | 28.7 | NA |
| Roden M 2013 | NCT01177813 | 24w | Multinational | T2D | None | Empagliflozin 10mg QD | 224 | 56.2 | 7.87 | 37 | 28.3 | NA |
|  |  |  |  |  |  | Empagliflozin 25mg QD | 224 | 53.8 | 7.86 | 35 | 28.2 | NA |
|  |  |  |  |  |  | Placebo | 228 | 54.9 | 7.91 | 46 | 28.7 | NA |
| Rosenstock J 2013 | NCT00749190 | 12w | Multinational | T2D | MET±oGLD | Empagliflozin 10mg QD | 71 | 59 | 7.9 | 54 | 31.4 | NA |
|  |  |  |  |  |  | Empagliflozin 25mg QD | 70 | 59 | 8.1 | 47 | 31.5 | NA |
|  |  |  |  |  |  | Empagliflozin 50mg QD | 70 | 56 | 7.9 | 44 | 31.8 | NA |
|  |  |  |  |  |  | Placebo | 71 | 60 | 8 | 54 | 31.3 | NA |
| Rosenstock J 2015 | NCT01011868 | 78w | Multinational | T2D | Ins | Empagliflozin 10mg QD | 169 | 58.6 | 8.3 | 45 | 32.1 | NA |
|  |  |  |  |  |  | Empagliflozin 25mg QD | 155 | 59.9 | 8.3 | 40 | 32.7 | NA |
|  |  |  |  |  |  | Placebo | 170 | 58.1 | 8.2 | 47 | 32.8 | NA |
| Kadowaki T 2014 | NCT01193218 | 12w | Japan | T2D | None/oGLD | Empagliflozin 10mg QD | 109 | 57.9 | 7.93 | 29.4 | 25.3 | NA |
|  |  |  |  |  |  | Empagliflozin 25mg QD | 109 | 57.3 | 7.93 | 22.9 | 25.1 | NA |
|  |  |  |  |  |  | Empagliflozin 50mg QD | 110 | 56.6 | 8.02 | 22.7 | 25 | NA |
|  |  |  |  |  |  | Placebo | 109 | 58.7 | 7.94 | 26.6 | 25.6 | NA |
| Kovacs CS 2014 | NCT01210001 | 24w | Multinational | T2D | PIO±MET | Empagliflozin 10mg QD | 165 | 54.7 | 8.1 | 49.7 | 29.2 | NA |
|  |  |  |  |  |  | Empagliflozin 25mg QD | 168 | 54.2 | 8.1 | 49.4 | 29.1 | NA |
|  |  |  |  |  |  | Placebo | 165 | 54.6 | 8.2 | 55.8 | 29.3 | NA |
| Merker L 2015 | NCT01159600 | 76w | Multinational | T2D | MET | Empagliflozin 10mg QD | 217 | 55.5 | 7.94 | 42 | 29.1 | NA |
|  |  |  |  |  |  | Empagliflozin 25mg QD | 213 | 55.6 | 7.86 | 44 | 29.7 | NA |
|  |  |  |  |  |  | Placebo | 207 | 56 | 7.9 | 44 | 28.7 | NA |
| Ross S 2015 | 2012000905-53 | 16w | Multinational | T2D | MET | Empagliflozin 12.5mg BID | 215 | 57.6 | 7.78 | 42.8 | 31.6 | NA |
|  |  |  |  |  |  | Empagliflozin 25mg QD | 214 | 58.2 | 7.73 | 46.7 | 32.1 | NA |
|  |  |  |  |  |  | Empagliflozin 5mg BID | 215 | 58.8 | 7.79 | 44.2 | 31.5 | NA |
|  |  |  |  |  |  | Empagliflozin 10mg QD | 214 | 58.5 | 7.84 | 49.5 | 31.9 | NA |
|  |  |  |  |  |  | Placebo | 107 | 57.9 | 7.69 | 48.6 | 32 | NA |
| Tikkanen I 2015 | NCT01370005 | 12w | Multinational | T2D + hypertension | None/oGLD | Empagliflozin 10mg QD | 276 | 60.6 | 7.87 | 38 | 32.4 | NA |
|  |  |  |  |  |  | Empagliflozin 25mg QD | 276 | 59.9 | 7.92 | 43.5 | 33 | NA |
|  |  |  |  |  |  | Placebo | 271 | 60.3 | 7.9 | 38 | 32.4 | NA |
| Zinman B 2015 | NCT01131676 | 156 | Multinational | T2D+CVD | None/oGLD | Empagliflozin 10mg QD | 2345 | 63 | 8.07 | 29.5 | 30.6 | NA |
|  |  |  |  |  |  | Empagliflozin 25mg QD | 2342 | 63.2 | 8.06 | 28.1 | 30.6 | NA |
|  |  |  |  |  |  | Placebo | 2333 | 63.2 | 8.08 | 28 | 30.7 | NA |
| Søftel+ E 2017 | NCT01734785 | 24w | Multinational | T2D | Linagliptin+MET | Empagliflozin 10mg QD | 109 | 54.3 | 7.97 | 39.4 | 31.2 | NA |
|  |  |  |  |  |  | Empagliflozin 25mg QD | 110 | 55.4 | 7.97 | 35.5 | 29.9 | NA |
|  |  |  |  |  |  | Placebo | 108 | 55.9 | 7.97 | 44.4 | 29.6 | NA |
| Kawamori R 2018Ⅰ | NCT02453555 | 24w | Japan | T2D | Linagliptin | Empagliflozin 5-10mg QD | 182 | 60 | 8.27 | 22 | 26 | NA |
|  |  |  |  |  |  | Placebo | 93 | 59.8 | 8.36 | 22.6 | 26.6 | NA |
| Ferdin+ KC 2019 | NCT02182830 | 24w | African  Americans | T2D + hypertension | None | Empagliflozin 10–25 mg QD | 78 | 56.5 | 8.66 | 44.9 | 36.04 | 9.3 |
|  |  |  |  |  |  | Placebo | 72 | 57.2 | 8.51 | 50 | 35.12 | 9.3 |
| Sone H 2020 | NCT02589639 | 52w | Japan | T2D | Ins±oGLD | Empagliflozin 10mg QD | 86 | 58.3 | 8.8 | 26.7 | 27 | NA |
|  |  |  |  |  |  | Empagliflozin 25mg QD | 90 | 58.6 | 8.7 | 32.2 | 26.8 | NA |
|  |  |  |  |  |  | Placebo | 90 | 59.1 | 8.7 | 23.3 | 26.9 | NA |
| Hussain M 2021Ⅰ | NA | 12w | Pakistan | T2D | oGLD | Empagliflozin 10–25 mg QD | 128 | 48.7 | 9.8 | 34 | 27 | 8.6 |
|  |  |  |  |  |  | Dapagliflozin 5-10mg QD | 127 | 55 | 8.9 | 28 | 27.8 | 10.2 |
| Hussain M 2021Ⅱ | NA | 12w | Pakistan | T2D | oGLD | Empagliflozin 10–25 mg QD | 155 | 52.9 | 10.1 | 30 | 28.5 | 9.2 |
|  |  |  |  |  |  | Dapagliflozin 5-10mg QD | 150 | 49.4 | 9.5 | 33 | 29 | 8.9 |
| Rodbard HW 2016 | NA | 26w | Multinational | T2D | MET+Sitagliptin | Canagliflozin 100mg QD | 107 | 57.4 | 8.5 | 38.3 | 32.3 | 9.8 |
|  |  |  |  |  |  | Placebo | 106 | 57.5 | 8.4 | 48.1 | 31.7 | 10.1 |
| Fonseca VA 2013 | NCT01071850 | 12w | Multinational | T2D | oGLD | Ipragliflozin 50mg QD | 67 | 52.6 | 8.05 | 49.3 | 32.2 | 4.61 |
|  |  |  |  |  |  | Ipragliflozin 150mg QD | 68 | 54.2 | 7.83 | 57.4 | 30.9 | 5.11 |
|  |  |  |  |  |  | Ipragliflozin 300mg QD | 68 | 54.3 | 7.9 | 45.6 | 30.7 | 4.48 |
|  |  |  |  |  |  | Placebo | 69 | 53.4 | 7.84 | 53.6 | 30.9 | 4.64 |
| Wilding JPH 2013 | NCT01117584 | 12w | Multinational | T2D | MET | Ipragliflozin 50mg QD | 68 | 58.6 | 7.76 | 52.9 | 31.1 | 6 |
|  |  |  |  |  |  | Ipragliflozin 150mg QD | 67 | 58.1 | 7.73 | 43.3 | 31.8 | 5.7 |
|  |  |  |  |  |  | Ipragliflozin 300mg QD | 72 | 56.6 | 7.87 | 50 | 31.8 | 5.5 |
|  |  |  |  |  |  | Placebo | 66 | 57.3 | 7.68 | 45.5 | 32 | 5.7 |
| Kashiwagi K 2014 | NCT00621868 | 12w | Japan | T2D | None | Ipragliflozin 50mg QD | 72 | 55.9 | 8.33 | 31.9 | 25.8 | 6.64 |
|  |  |  |  |  |  | Ipragliflozin 100mg QD | 72 | 56 | 8.25 | 27.8 | 25.9 | 7.8 |
|  |  |  |  |  |  | Placebo | 69 | 55.2 | 8.36 | 43.5 | 25.1 | 6.31 |
| Kashiwagi A 2015 Ⅰ | NCT01242215 | 24w | Japan | T2D | SU | Ipragliflozin 50mg QD | 165 | 59.6 | 8.38 | 32.7 | 25.81 | 10.32 |
|  |  |  |  |  |  | Placebo | 75 | 59.8 | 8.34 | 37.7 | 24.18 | 10.75 |
| Kashiwagi K 2015 | NCT01135433 | 24w | Japan | T2D | MET | Ipragliflozin 50mg QD | 112 | 56.2 | 8.25 | 41.1 | 25.96 | 8.05 |
|  |  |  |  |  |  | Placebo | 56 | 57.7 | 8.38 | 41.1 | 25.47 | 7.49 |
| Kashiwagi A 2015 Ⅱ | NCT01057628 | 16w | Japan | T2D | oGLD | Ipragliflozin 50mg QD | 67 | 60.6 | 8.4 | 32.3 | 25.3 | 7.53 |
|  |  |  |  |  |  | Placebo | 62 | 58.3 | 8.25 | 28.4 | 25.6 | 5.9 |
| Kashiwagi A 2015 Ⅲ | NCT01225081 | 24w | Japan | T2D | PIO | Ipragliflozin 50mg QD | 97 | 56.2 | 8.24 | 22.7 | 27.11 | 6.3 |
|  |  |  |  |  |  | Placebo | 54 | 56.1 | 8.39 | 31.5 | 27.13 | 7.7 |
| Ishihara H 2016 | NCT02175784 | 16w | Japan | T2D | Ins | Ipragliflozin 50mg QD | 168 | 58.7 | 8.67 | 37.5 | 25.61 | 12.58 |
|  |  |  |  |  |  | Placebo | 87 | 59.2 | 8.62 | 41.4 | 26.42 | 14.25 |
| Lu CH 2016 | NCT01505426 | 24w | Asian | T2D | MET | Ipragliflozin 50mg QD | 87 | 53.4 | 7.75 | 60.2 | 27.04 | 6.49 |
|  |  |  |  |  |  | Placebo | 83 | 53.9 | 7.74 | 49.4 | 26.57 | 5.82 |
| Han KA 2018 | NCT02452632 | 24w | Korean | T2D | MET+Sitagliptin | Ipragliflozin 50mg QD | 73 | 57.62 | 7.9 | 49.3 | 26.05 | 11.62 |
|  |  |  |  |  |  | Placebo | 66 | 57.44 | 7.92 | 51.5 | 32.8 | 11.33 |
| Shestakova MV 2018 | NCT02794792 | 12w | Russian | T2D | MET | Ipragliflozin 50mg QD | 110 | 58.9 | 8.39 | 56.4 | 25.5 | 6.65 |
|  |  |  |  |  |  | Placebo | 55 | 58 | 8.46 | 60 | 31.95 | 6.56 |
| Kaku K 2021 | NCT02452632 | 24w | Japan | T2D | Sitagliptin | Ipragliflozin 50mg QD | 73 | 61 | 8.1 | 26 | 26 | 9.6 |
|  |  |  |  |  |  | Placebo | 70 | 60 | 8 | 24.3 | 25.7 | 9 |
| Rosenstock J 2014 | NCT01376557 | 12w | Multinational | T2D | MET | Sotagliflozin 200 mg QD | 60 | 56.1 | 8.3 | 71.7 | 34.2 | NA |
|  |  |  |  |  |  | Sotagliflozin 200 mg BID | 60 | 56.4 | 8.4 | 51.7 | 32.9 | NA |
|  |  |  |  |  |  | Sotagliflozin 400 mg QD | 60 | 56.1 | 8.1 | 51.7 | 32.7 | NA |
|  |  |  |  |  |  | Placebo | 60 | 55.1 | 7.9 | 56.7 | 32.2 | NA |
| Sykes AP 2014 Ⅰ | NCT00500331 | 12w | Multinational | T2D | None | Remogliflozin 100 mg BID | 48 | 56 | 8.08 | 34 | 29.79 | 2.48 |
|  |  |  |  |  |  | Remogliflozin 250 mg BID | 48 | 55.3 | 8.14 | 36 | 31.53 | 1.83 |
|  |  |  |  |  |  | Remogliflozin 500 mg BID | 48 | 54.3 | 8.03 | 43 | 31.15 | 2.73 |
|  |  |  |  |  |  | Remogliflozin 1000 mg BID | 47 | 52.4 | 8.11 | 43 | 32.22 | 2.43 |
|  |  |  |  |  |  | Placebo | 47 | 55.8 | 8.17 | 38 | 31.02 | 1.71 |
| Sykes AP 2014 Ⅱ | NCT00495469 | 12w | Multinational | T2D | None | Remogliflozin 250 mg QD | 34 | 54.3 | 8.19 | 42 | 31.41 | 1.66 |
|  |  |  |  |  |  | Remogliflozin 500 mg QD | 36 | 53.2 | 7.96 | 68 | 32.94 | 2.70 |
|  |  |  |  |  |  | Remogliflozin 1000 mg QD | 36 | 54.3 | 7.91 | 37 | 29.86 | 2.44 |
|  |  |  |  |  |  | Remogliflozin 250 mg BID | 36 | 50.2 | 8.05 | 57 | 30.78 | 2.3 |
|  |  |  |  |  |  | Placebo | 33 | 52.1 | 8.19 | 58 | 32.32 | 2.36 |
| Dharmalingam M 2020 | CTRI/2017/07/009121 | 24w | India | T2D | MET | Remogliflozin 100 mg BID | 224 | 50.86 | 8.19 | 42.9 | 28.19 | NA |
|  |  |  |  |  |  | Remogliflozin 250 mg BID | 241 | 50.87 | 8.28 | 47.3 | 27.82 | NA |
|  |  |  |  |  |  | Dapagliflozin 10 mg QD | 146 | 50.15 | 8.26 | 41.8 | 28 | NA |
| NCT03386344 2021 | NCT03386344 | 106w | Multinational | T2D | None | Sotagliflozin 200 mg QD | 125 | 66.1 | 8.32 | 44.8 | NA | NA |
|  |  |  |  |  |  | Sotagliflozin 400 mg QD | 125 | 66.5 | 8.32 | 44 | NA | NA |
|  |  |  |  |  |  | Placebo | 125 | 66.3 | 8.38 | 44.4 | NA | NA |
| NCT02926937 2021 | NCT02926937 | 26w | Multinational | T2D | None | Sotagliflozin 400 mg QD | 142 | 52.3 | 8 | 49.5 | NA | NA |
|  |  |  |  |  |  | Sotagliflozin 200 mg QD | 107 | 54.7 | 8.14 | 47.9 | NA | NA |
|  |  |  |  |  |  | Placebo | 150 | 55.3 | 8.11 | 48 | NA | NA |
| NCT02926950 2021 | NCT02926950 | 53w | Multinational | T2D | MET | Sotagliflozin 400 mg QD | 259 | 60 | 8.2 | 45.2 | NA | NA |
|  |  |  |  |  |  | Placebo | 259 | 59.9 | 8.19 | 43.6 | NA | NA |
| NCT03351478 2021 | NCT03351478 | 26w | Multinational | T2D | DPP-4i/MET | Sotagliflozin 400 mg QD | 307 | 58.9 | 8.23 | 45.9 | NA | NA |
|  |  |  |  |  |  | Empagliflozin 25mg QD | 309 | 59.7 | 8.21 | 51.1 | NA | NA |
|  |  |  |  |  |  | Placebo | 154 | 59.8 | 8.21 | 48.7 | NA | NA |
| NCT03285594 2021 | NCT03285594 | 55.7w | Multinational | T2D | Ins±oGLD | Sotagliflozin 200 mg QD | 141 | 62.1 | 8.76 | 46.1 | NA | NA |
|  |  |  |  |  |  | Sotagliflozin 400 mg QD | 285 | 62.7 | 8.69 | 46.2 | NA | NA |
|  |  |  |  |  |  | Placebo | 144 | 62.2 | 8.76 | 40.3 | NA | NA |
| NCT03066830 2021 | NCT03066830 | 79w | Multinational | T2D | SU±MET | Sotagliflozin 400 mg QD | 254 | 63.3 | 8.2 | 41.1 | NA | NA |
|  |  |  |  |  |  | Placebo | 253 | 63 | 8.18 | 48.8 | NA | NA |
| NCT03332771 2021 | NCT03332771 | 52w | Multinational | T2D | MET | Sotagliflozin 200 mg QD | 160 | 58.6 | 8.11 | 46.9 | NA | NA |
|  |  |  |  |  |  | Sotagliflozin 400 mg QD | 317 | 59.7 | 8.02 | 49.5 | NA | NA |
|  |  |  |  |  |  | Placebo | 159 | 58.8 | 8.12 | 48.4 | NA | NA |
| NCT01649297 2015 | NCT01649297 | 16w | Multinational | T2D | MET | Empagliflozin 12.5mg BID | 219 | 57.6 | NA | 42.8 | NA | NA |
|  |  |  |  |  |  | Empagliflozin 25mg QD | 218 | 58.2 | NA | 46.7 | NA | NA |
|  |  |  |  |  |  | Empagliflozin 5mg BID | 219 | 58.8 | NA | 44.2 | NA | NA |
|  |  |  |  |  |  | Empagliflozin 10mg QD | 220 | 58.5 | NA | 49.5 | NA | NA |
|  |  |  |  |  |  | Placebo | 107 | 57.9 | NA | 48.6 | NA | NA |
| NCT01989754 2018 | NCT01989754 | 156w | Multinational | T2D+CVD | None/oGLD | Canagliflozin 100-300mg QD | 2904 | 63.9 | NA | 36.2 | NA | NA |
|  |  |  |  |  |  | Placebo | 2903 | 64 | NA | 37.2 | NA | NA |
| NCT01032629 2018 | NCT01032629 | 338w | Multinational | T2D+CVD | None | Canagliflozin 100mg QD | 1445 | 62.2 | NA | 33.5 | NA | NA |
|  |  |  |  |  |  | Canagliflozin 300mg QD | 1443 | 62.8 | NA | 34.6 | NA | NA |
|  |  |  |  |  |  | Placebo | 1442 | 62.3 | NA | 33.7 | NA | NA |
| NCT02235298 2021 | NCT02235298 | 24w | Multinational | T2D | MET | Dapagliflozin 10 mg QD | 50 | 52 | NA | 58 | NA | NA |
|  |  |  |  |  |  | Placebo | 50 | 51 | NA | 60 | NA | NA |
| **Studies in patients with renal insufficiency** | | | | | | | | | | | | |
| Bhatt DL 2020Ⅱ | NCT03315143 | 64w | Multinational | T2D+CKD(eGFR 25- 60) | None | Sotagliflozin 200-400mg QD | 5292 | 69 | 8.3 | 44.3 | 31.9 | NA |
|  |  |  |  |  |  | Placebo | 5292 | 69 | 8.3 | 45.5 | 31.7 | NA |
| Haneda M 2016 | JapicCTI-111543 | 24w | Japan | T2D+CKD(eGFR 30- 60) | None/oGLD | Luseogliflozin 2.5mg QD | 95 | 67.9 | 7.72 | 24.2 | 25.45 | 10.4 |
|  |  |  |  |  |  | Placebo | 50 | 68.4 | 7.69 | 22 | 25.81 | 12.6 |
| Grunberger G 2018Ⅰ | NCT01986855 | 26w | Multinational | T2D+CKD(eGFR 30- 60) | Ins±SU | Ertugliflozin 5mg QD | 158 | 66.7 | 8.2 | 46.8 | 32.6 | 14.9 |
|  |  |  |  |  |  | Ertugliflozin 15mg QD | 155 | 67.5 | 8.2 | 52.6 | 31.7 | 14.5 |
|  |  |  |  |  |  | Placebo | 154 | 67.5 | 8.1 | 53.2 | 33.2 | 13.1 |
| Yale JF 2013 | NCT01064414 | 52w | Multinational | T2D+CKD(eGFR 30- 50) | oGLD | Canagliflozin 100mg QD | 90 | 69.5 | 7.9 | 35.6 | 32.4 | 15.6 |
|  |  |  |  |  |  | Canagliflozin 300mg QD | 89 | 67.9 | 8 | 46.1 | 33.4 | 17 |
|  |  |  |  |  |  | Placebo | 90 | 68.2 | 8 | 36.7 | 33.1 | 16.4 |
| Perkovic V 2019 | NCT01064414 | 136w | Multinational | T2D+CKD(eGFR 30- 90) | None | Canagliflozin 100mg QD | 2202 | 62.9 | 8.3 | 34.6 | 31.4 | 15.5 |
|  |  |  |  |  |  | Placebo | 2199 | 63.6 | 8.3 | 33.3 | 31.3 | 16 |
| Kohan DE 2014 | NA | 104w | Multinational | T2D+CKD(eGFR 30- 60) | oGLD | Dapagliflozin 5 mg QD | 83 | 66 | 8.3 | 33.7 | NA | 16.9 |
|  |  |  |  |  |  | Dapagliflozin 10 mg QD | 85 | 68 | 8.22 | 34.1 | NA | 18.2 |
|  |  |  |  |  |  | Placebo | 84 | 67 | 8.53 | 36.9 | NA | 15.7 |
| Fioretto P 2018 | NCT02413398 | 24w | Multinational | T2D+CKD(eGFR 45- 59) | oGLD | Dapagliflozin 10 mg QD | 160 | 65.3 | 8.33 | 43.1 | 32.6 | 14.3 |
|  |  |  |  |  |  | Placebo | 161 | 66.2 | 8.03 | 43.5 | 31.6 | 14.5 |
| Pollock C 2019 | NCT02547935 | 24w | Multinational | T2D+CKD(eGFR 25- 75) | Saxagliptin | Dapagliflozin 10 mg QD | 145 | 64.7 | 8.44 | 30 | 30.19 | 17.55 |
|  |  |  |  |  |  | Placebo | 148 | 64.7 | 8.57 | 29 | 30.34 | 17.71 |
| Barnett AH 2014 | NCT01164501 | 52w | Multinational | T2D+CKD(eGFR 60- 90) | oGLD | Empagliflozin 10mg QD | 98 | 63.2 | 8.02 | 38.8 | 32.4 | NA |
|  |  |  |  |  |  | Empagliflozin 25mg QD | 97 | 62 | 7.96 | 37.1 | 31.3 | NA |
|  |  |  |  |  |  | Placebo | 95 | 62.6 | 8.09 | 41.1 | 30.8 | NA |
|  |  |  |  | T2D+CKD(eGFR 30- 60) |  | Empagliflozin 25mg QD | 187 | 64.4 | 8.02 | 42.8 | 30.2 | NA |
|  |  |  |  |  |  | Placebo | 187 | 65.1 | 8.09 | 43.3 | 30.3 | NA |
|  |  |  |  | T2D+CKD  (eGFR 15-30) |  | Empagliflozin 25mg QD | 37 | 65.4 | 8.06 | 43.2 | 29 | NA |
|  |  |  |  |  |  | Placebo | 37 | 62.9 | 8.16 | 48.6 | 31.8 | NA |
| Wanner C 2016 | NCT01131676 | 156w | Multinational | T2D+CKD  (eGFR<59) | None | Empagliflozin 10-25mg QD | 1212 | 67.1 | 8.07 | 32.7 | 31 | NA |
|  |  |  |  |  |  | Placebo | 607 | 67.1 | 8.03 | 31.1 | 30.9 | NA |
|  |  |  |  | T2D+CKD (eGFR ≥ 60) |  | Empagliflozin 10-25mg QD | 3473 | 61.7 | 8.07 | 27.5 | 30.5 | NA |
|  |  |  |  |  |  | Placebo | 1726 | 61.9 | 8.1 | 26.9 | 30.6 | NA |
| Kashiwagi A 2015 | NCT01316094 | 24w | Japan | T2D+CKD (eGFR 60- 90) | oGLD | Ipragliflozin 50mg QD | 118 | 63.9 | 7.53 | 22 | 25.84 | 9.53 |
|  |  |  |  |  |  | Placebo | 46 | 65.7 | 7.55 | 21.7 | 24.96 | 9.42 |
| NCT03242252 2021 | NCT03242252 | 54w | Multinational | T2D+CKD (eGFR 30- 60) | None/oGLD | Sotagliflozin 200 mg QD | 263 | 69.6 | 8.33 | 45.6 | NA | NA |
|  |  |  |  |  |  | Sotagliflozin 400 mg QD | 264 | 69.5 | 8.31 | 42.4 | NA | NA |
|  |  |  |  |  |  | Placebo | 260 | 69.3 | 8.33 | 42.7 | NA | NA |
| Cherney DZI 2021 | NCT03242018 | 52w | Multinational | T2D+CKD (eGFR 15-30) | oGLD | Sotagliflozin 200 mg QD | 92 | 66.8 | 8.3 | 52.2 | 30.9 | 19.6 |
|  |  |  |  |  |  | Sotagliflozin 400 mg QD | 92 | 67.3 | 8.3 | 46.7 | 32.1 | 18.5 |
|  |  |  |  |  |  | Placebo | 93 | 68 | 8.4 | 54.8 | 31.7 | 20.7 |
| **Extention studies** | | | | | | | | | | | | |
| Bode B 2015 | NA | 104w | Multinational | T2D(55-80years) | None/oGLD | Canagliflozin 100mg QD | 241 | 64.3 | 7.8 | 48.5 | 31.4 | 12.3 |
|  |  |  |  |  |  | Canagliflozin 300mg QD | 236 | 63.4 | 7.7 | 45.3 | 31.5 | 11.3 |
|  |  |  |  |  |  | Placebo | 237 | 63.2 | 7.8 | 39.7 | 31.8 | 11.4 |
| Jabbour SA 2020 | NCT02229396 | 104w | Multinational | T2D | Exenatide+MET | Dapagliflozin 10 mg QD | 228 | 53.8 | 9.3 | 49 | 33.2 | 7.6 |
|  |  |  |  |  |  | Placebo | 227 | 54.2 | 9.3 | 55 | 32 | 7.4 |
| Haering HU 2015 | NCT01289990 | 76w | Multinational | T2D | MET+SU | Empagliflozin 10mg QD | 225 | 56.9 | 8.1 | 50.2 | 28.3 | NA |
|  |  |  |  |  |  | Empagliflozin 25mg QD | 216 | 57 | 8.1 | 49.8 | 28.3 | NA |
|  |  |  |  |  |  | Placebo | 225 | 57.4 | 8.2 | 47.2 | 27.9 | NA |
| Dagogo-Jack S 2018Ⅱ | NCT02036515 | 52w | Multinational | T2D | MET+Sitagliptin | Ertugliflozin 5mg QD | 156 | 59.2 | 8.1 | 48.1 | 31.2 | 9.9 |
|  |  |  |  |  |  | Ertugliflozin 15 mg QD | 153 | 59.7 | 8 | 46.4 | 30.9 | 9.2 |
|  |  |  |  |  |  | Placebo | 153 | 58.3 | 8 | 34.6 | 30.3 | 9.4 |
| Wilding JPH 2013 | NCT01106625 | 52w | Multinational | T2D | MET+SU | Canagliflozin 100mg QD | 157 | 57.4 | 8.1 | 51.6 | 33.3 | 9.1 |
|  |  |  |  |  |  | Canagliflozin 300mg QD | 156 | 56.1 | 8.1 | 44.2 | 33.2 | 9.4 |
|  |  |  |  |  |  | Placebo | 156 | 56.8 | 8.1 | 51.3 | 32.7 | 10.3 |
| Bailey CJ 2013 | NCT00528879 | 102w | Multinational | T2D | MET | Dapagliflozin 5mg QD | 137 | 54.3 | 8.17 | 50 | 31.4 | 6.4 |
|  |  |  |  |  |  | Dapagliflozin 10 mg QD | 135 | 52.7 | 7.92 | 43 | 31.2 | 6.1 |
|  |  |  |  |  |  | Placebo | 137 | 53.7 | 8.11 | 45 | 31.8 | 5.8 |
| Strojek K 2014 | NCT00680745 | 48w | Multinational | T2D | Glimepiride | Dapagliflozin 5mg QD | 142 | 60.2 | 8.12 | 50 | 29.8 | 7.4 |
|  |  |  |  |  |  | Dapagliflozin 10 mg QD | 151 | 58.9 | 8.07 | 56.3 | 29.7 | 7.2 |
|  |  |  |  |  |  | Placebo | 145 | 60.3 | 8.15 | 51 | 29.8 | 7.4 |
| Wilding JPH 2014 | NCT00673231 | 104w | Multinational | T2D | Ins±oGLD | Dapagliflozin 5mg QD | 211 | 59.3 | 8.62 | 52.6 | 33 | 13.1 |
|  |  |  |  |  |  | Dapagliflozin 10 mg QD | 194 | 59.3 | 8.57 | 55.2 | 33.4 | 14.2 |
|  |  |  |  |  |  | Placebo | 193 | 58.8 | 8.47 | 50.8 | 33.1 | 13.5 |
| Roden M 2015 | NCT01177813 | 76w | Multinational | T2D | None | Empagliflozin 10mg QD | 224 | 56.2 | 7.87 | 37 | 28.3 | NA |
|  |  |  |  |  |  | Empagliflozin 25mg QD | 224 | 53.8 | 7.86 | 35 | 28.2 | NA |
|  |  |  |  |  |  | Placebo | 228 | 54.9 | 7.91 | 46 | 28.7 | NA |
| Kovacs CS 2015 | NCT01210001 | 76w | Multinational | T2D | PIO±MET | Empagliflozin 10mg QD | 165 | 54.7 | 8.1 | 49.7 | 29.2 | NA |
|  |  |  |  |  |  | Empagliflozin 25mg QD | 168 | 54.2 | 8.1 | 49.4 | 29.1 | NA |
|  |  |  |  |  |  | Placebo | 165 | 54.6 | 8.2 | 55.8 | 29.3 | NA |
| Kawamori R 2018Ⅱ | NCT02453555 | 52w | Japan | T2D | Linagliptin | Empagliflozin 5-10mg QD | 182 | 60 | 8.27 | 22 | 26 | NA |
|  |  |  |  |  |  | Placebo | 93 | 59.8 | 8.36 | 22.6 | 26.6 | NA |

HbA1c: Hemoglobin A1c, BMI: Body Mass Index, oGLD: the other glucose lowering drugs, Ins:Insulin, DPP-4i: Dipeptidyl peptidase-4 inhibitors, CVD: [Cardiovascular disease](javascript:;), T2D: Type 2 diabetes, QD: Quaque die, BID: Bis in die, SU: Sulfonylurea, PIO: Pioglitazone, MET: Metformin, CKD: Chronic kidney disease, eGFR: Estimated glomerular filtration rate, unit is mL/min/1.73 m^2^, w: Weeks, NA: Not applicable

# Appendix 5: Risk of Bias Assessment

## 5.1 The results of risk of bias assessment for each study

| **Study** | **Randomization process** | **Deviations from intended interventions** | **Mising outcome data** | **Measurement of the outcome** | **Selection of the reported result** | **Overall Bias** |
| --- | --- | --- | --- | --- | --- | --- |
| Kaku K 2014 | Low | Low | Low | Low | Low | Low |
| Ikeda S 2015 | Low | Low | Low | Low | Some concerns | Some concerns |
| Terauchi Y 2017 | Some concerns | Low | Low | Low | Some concerns | Some concerns |
| Seino Y2014Ⅰ | Low | Low | Low | Low | Low | Low |
| Seino Y2014Ⅱ | Low | Low | Low | Low | Low | Low |
| Haneda M 2016 | Some concerns | Low | Low | Low | Some concerns | Some concerns |
| Seino Y 2018 | Low | Low | Low | Low | Low | Low |
| Seino Y 2014Ⅲ | Low | Low | Low | Low | Low | Low |
| Amin NB 2015 | Low | Low | Low | Low | Low | Low |
| Terra SG 2017 | Low | Low | Low | Low | Some concerns | Some concerns |
| Dagogo-Jack S 2018Ⅰ | Low | Low | Low | Low | Some concerns | Some concerns |
| Grunberger G 2018Ⅰ | Low | Low | Low | Low | Low | Low |
| Rosenstock J 2018 | Low | Low | Low | Low | Some concerns | Some concerns |
| Ji LN 2019 | Some concerns | Some concerns | Low | Some concerns | Low | Some concerns |
| Cannon CP 2020 | Low | Low | Low | Low | Some concerns | Some concerns |
| LIST JF 2009 | Low | Low | Low | Low | Low | Low |
| Rosenstock J 2012 | Low | Low | Low | Low | Low | Low |
| Inagaki N 2013 | Low | Low | Low | Low | Some concerns | Some concerns |
| Bode B 2013 | Low | Low | Low | Low | Low | Low |
| Stenlöf K 2013 | Low | Low | Low | Low | Low | Low |
| Wilding JPH 2013Ⅰ | Low | Low | Low | Low | Low | Low |
| Yale JF 2013 | Low | Low | Low | Low | Low | Low |
| Inagaki N 2014 | Some concerns | Low | Low | Low | Low | Some concerns |
| Qiu R 2014 | Low | Low | Low | Low | Some concerns | Some concerns |
| Ji LN 2015 | Low | Low | Low | Low | Low | Low |
| Inagaki N 2016 | Low | Low | Low | Low | Low | Low |
| Kadowaki T 2017 | Low | Low | Low | Low | Low | Low |
| Perkovic V 2019 | Low | Low | Low | Low | Low | Low |
| Bailey CJ 2010 | Low | Low | Low | Low | Some concerns | Some concerns |
| Ferranninie E 2010 | Low | Low | Low | Low | Low | Low |
| Strojek K 2011 | Low | Low | Low | Low | Some concerns | Some concerns |
| Bailey CJ 2012 | Low | Low | Low | Low | Some concerns | Some concerns |
| Bolinder J 2012 | Low | Low | Low | Low | Some concerns | Some concerns |
| Rosenstock J 2012 D | Low | Low | Low | Low | Some concerns | Some concerns |
| Wilding JPH 2012 | Low | Low | Low | Low | Some concerns | Some concerns |
| Kaku K 2013 | Low | Low | Low | Low | Some concerns | Some concerns |
| Ji LN 2014 | Low | Low | Low | Low | Low | Low |
| Kohan DE 2014 | Some concerns | Low | Low | Low | Low | Some concerns |
| Leiter LA 2014 | Low | Low | Low | Low | Some concerns | Some concerns |
| Bailey CJ 2015 | Low | Low | Low | Low | Low | Low |
| Cefalu WT 2015 | Low | Low | Low | Low | Some concerns | Some concerns |
| Mathieu C 2015 | Low | Low | Low | Low | Low | Low |
| Matthaei S 2015 | Low | Low | Low | Low | Low | Low |
| Schumm-Draeger PM 2015 | Low | Low | Low | Low | Low | Low |
| Araki E 2016 | Low | Low | Low | Low | Low | Low |
| Michael A 2016 | Low | Low | Low | Low | Low | Low |
| Weber MA 2016 | Low | Low | Low | Low | Some concerns | Some concerns |
| Yang WY 2016 | Low | Low | Low | Low | Low | Low |
| Fioretto P 2018 | Low | Low | Low | Low | Low | Low |
| Yang WY 2018 | Low | Low | Low | Low | Low | Low |
| Pollock C 2019 | Low | Low | Low | Low | Low | Low |
| Wiviott SD 2019 | Low | Low | Low | Low | Low | Low |
| Fr´ıas JP 2016 | Low | Low | Low | Low | Low | Low |
| Ferrannini E 2013 | Low | Low | Low | Low | Low | Low |
| Haring HU 2013 | Low | Low | Low | Low | Low | Low |
| Häring HU 2014 | Low | Low | Low | Low | Low | Low |
| Roden M 2013 | Low | Low | Low | Low | Low | Low |
| Rosenstock J 2013 | Low | Low | Low | Low | Low | Low |
| Rosenstock J 2015 | Low | Low | Low | Low | Low | Low |
| Barnett AH 2014 | Low | Low | Low | Low | Low | Low |
| Kadowaki T 2014 | Low | Low | Low | Low | Some concerns | Some concerns |
| Kovacs CS 2014 | Low | Low | Low | Low | Low | Low |
| Merker L 2015 | Low | Low | Low | Low | Low | Low |
| Ross S 2015 | Some concerns | Low | Low | Low | Low | Some concerns |
| Tikkanen I 2015 | Low | Low | Low | Low | Low | Low |
| Zinman B 2015 | Low | Low | Low | Low | Low | Low |
| Wanner C 2016 | Low | Low | Low | Low | Low | Low |
| Søfteland E 2017 | Low | Low | Low | Low | Low | Low |
| Kawamori R 2018Ⅰ | Low | Low | Low | Low | Low | Low |
| Ferdinand KC 2019 | Low | Low | Low | Low | Low | Low |
| Sone H 2020 | Low | Low | Low | Low | Low | Low |
| Hussain M 2021Ⅰ | Some concerns | Low | High | Low | Low | High |
| Hussain M 2021Ⅱ | Some concerns | Low | Low | Low | Low | Some concerns |
| Rodbard HW 2016 | Low | Low | Low | Low | Low | Low |
| Fonseca VA 2013 | Low | Low | Low | Low | Low | Low |
| Wilding JPH 2013 | Low | Low | Low | Low | Low | Low |
| Kashiwagi K 2014 | Low | Low | Low | Low | Low | Low |
| Kashiwagi A 2015 Ⅰ | Low | Low | Low | Low | Low | Low |
| Kashiwagi K 2015 | Low | Low | Low | Low | Low | Low |
| Kashiwagi A 2015 Ⅱ | Low | Low | Low | Low | Low | Low |
| Kashiwagi A 2015 Ⅲ | Low | Low | Low | Low | Low | Low |
| Kashiwagi A 2015 | Low | Low | Low | Low | Low | Low |
| Ishihara H 2016 | Low | Low | Low | Low | Low | Low |
| Lu CH 2016 | Low | Low | Low | Low | Low | Low |
| Han KA 2018 | Low | Low | Low | Low | Low | Low |
| Shestakova MV 2018 | Low | Low | Low | Low | Low | Low |
| Kaku K 2021 | Low | Low | Low | Low | Low | Low |
| Rosenstock J 2014 | Low | Low | Low | Low | Low | Low |
| Cherney DZI 2021 | Low | Low | Low | Low | Low | Low |
| Bhatt DL 2020Ⅱ | Low | Low | Low | Low | Low | Low |
| Sykes AP 2014 Ⅰ | Low | Low | Low | Low | Low | Low |
| Sykes AP 2014 Ⅱ | Some concerns | Low | High | Low | Low | High |
| Dharmalingam M 2020 | Low | Low | Low | Low | Low | Low |
| NCT03386344 2021 | Low | Low | Low | Low | Low | Low |
| NCT02926937 2021 | Low | Low | Low | Low | Low | Low |
| NCT02926950 2021 | Low | Low | Low | Low | Low | Low |
| NCT03242252 2021 | Low | Low | Low | Low | Some concerns | Some concerns |
| NCT03351478 2021 | Low | Low | Low | Low | Low | Low |
| NCT03285594 2021 | Low | Low | Low | Low | Low | Low |
| NCT03066830 2021 | Low | Low | Low | Low | Low | Low |
| NCT03332771 2021 | Low | Low | Low | Low | Low | Low |
| NCT01649297 2015 | Low | Low | Low | Low | Low | Low |
| NCT01989754 2018 | Low | Low | Low | Low | Low | Low |
| NCT01032629 2018 | Low | Low | Low | Low | Low | Low |
| NCT02235298 2021 | Low | Low | Low | Low | Low | Low |

5.2 The results of risk of bias assessment for each outcome

### 5.2.1 Reproductive tract infections_RoB_chart


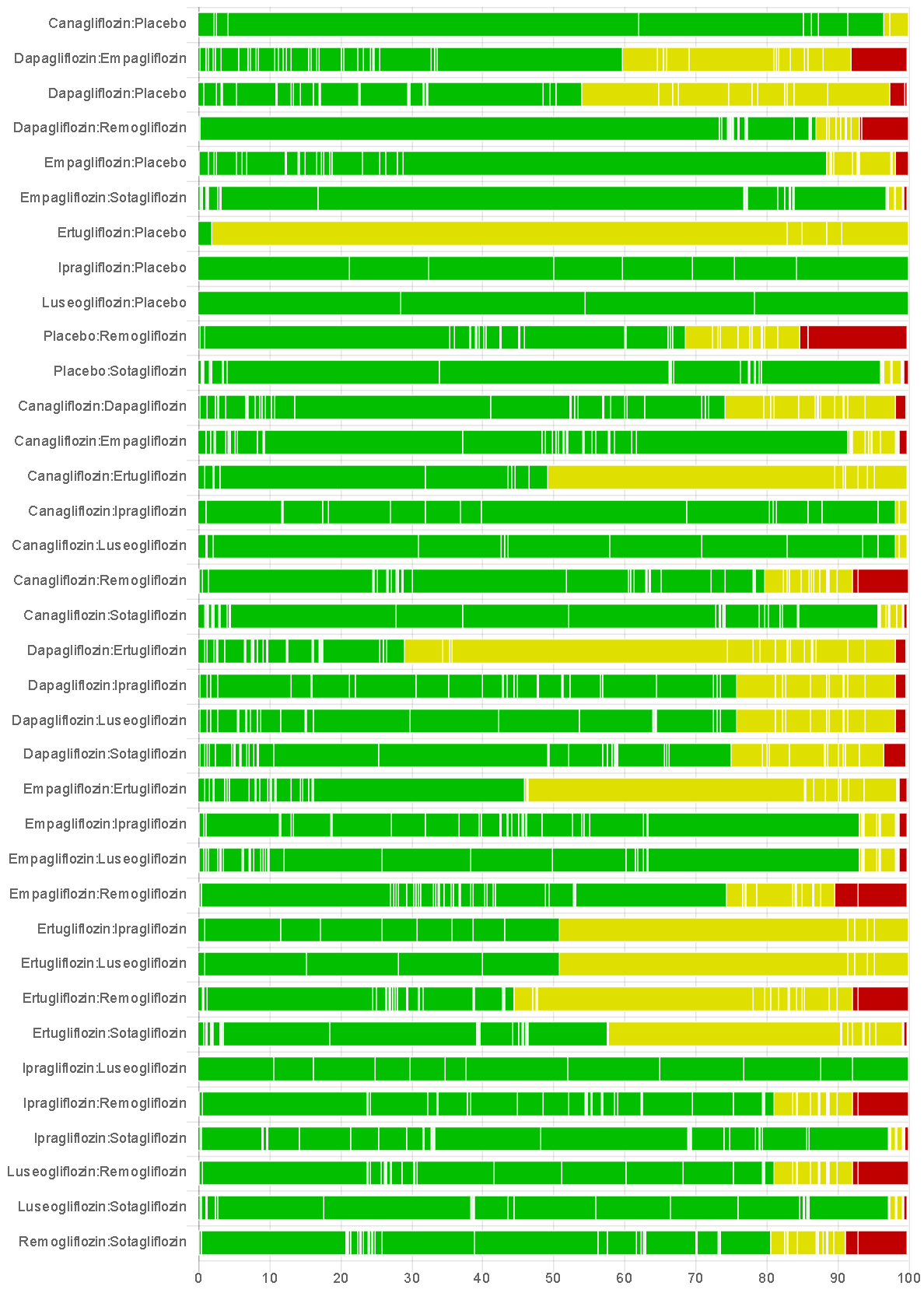


5.2.2 Pollakiuria_RoB_chart


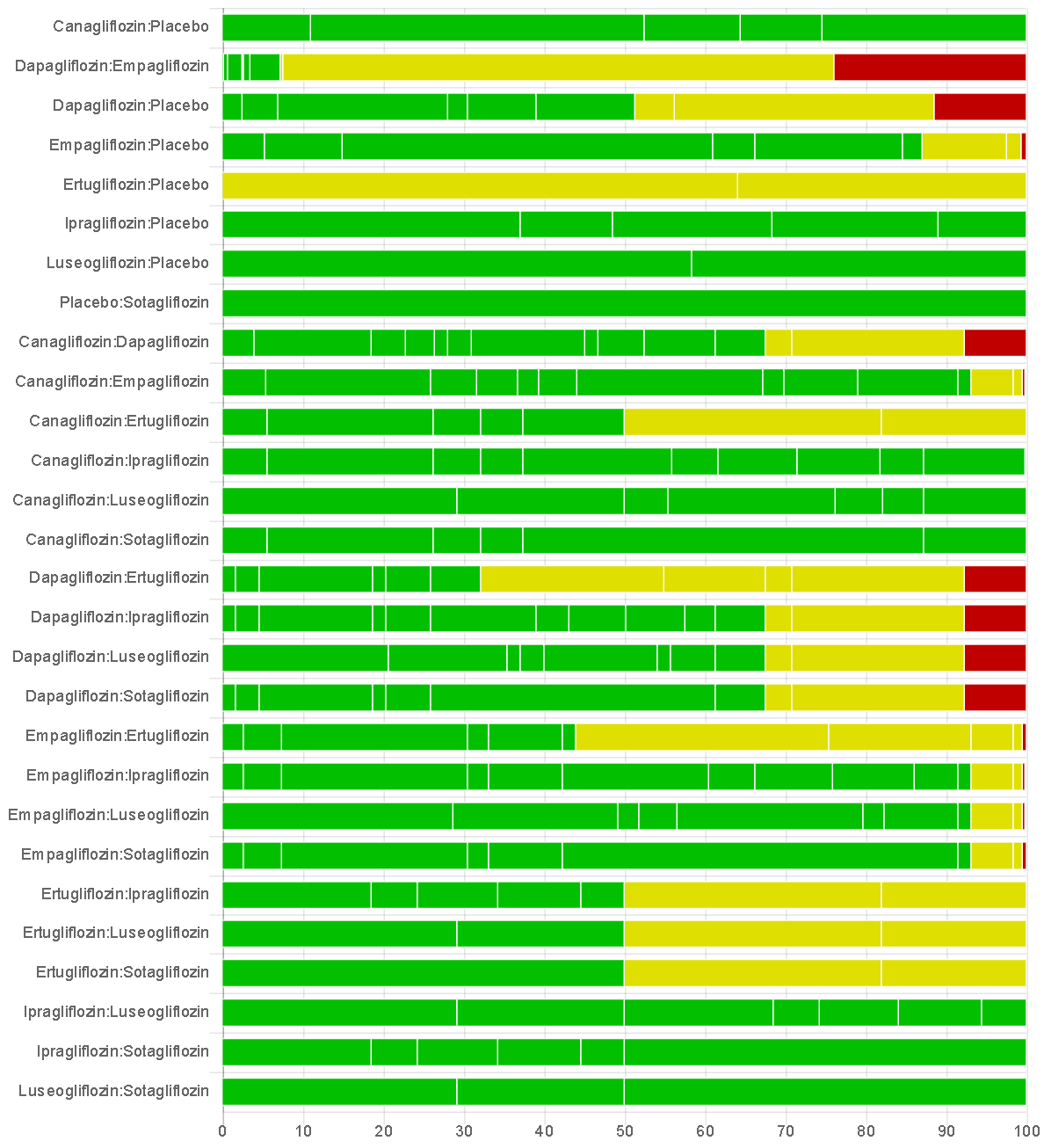


**5.2.3 Hypovolemia_RoB_chart**


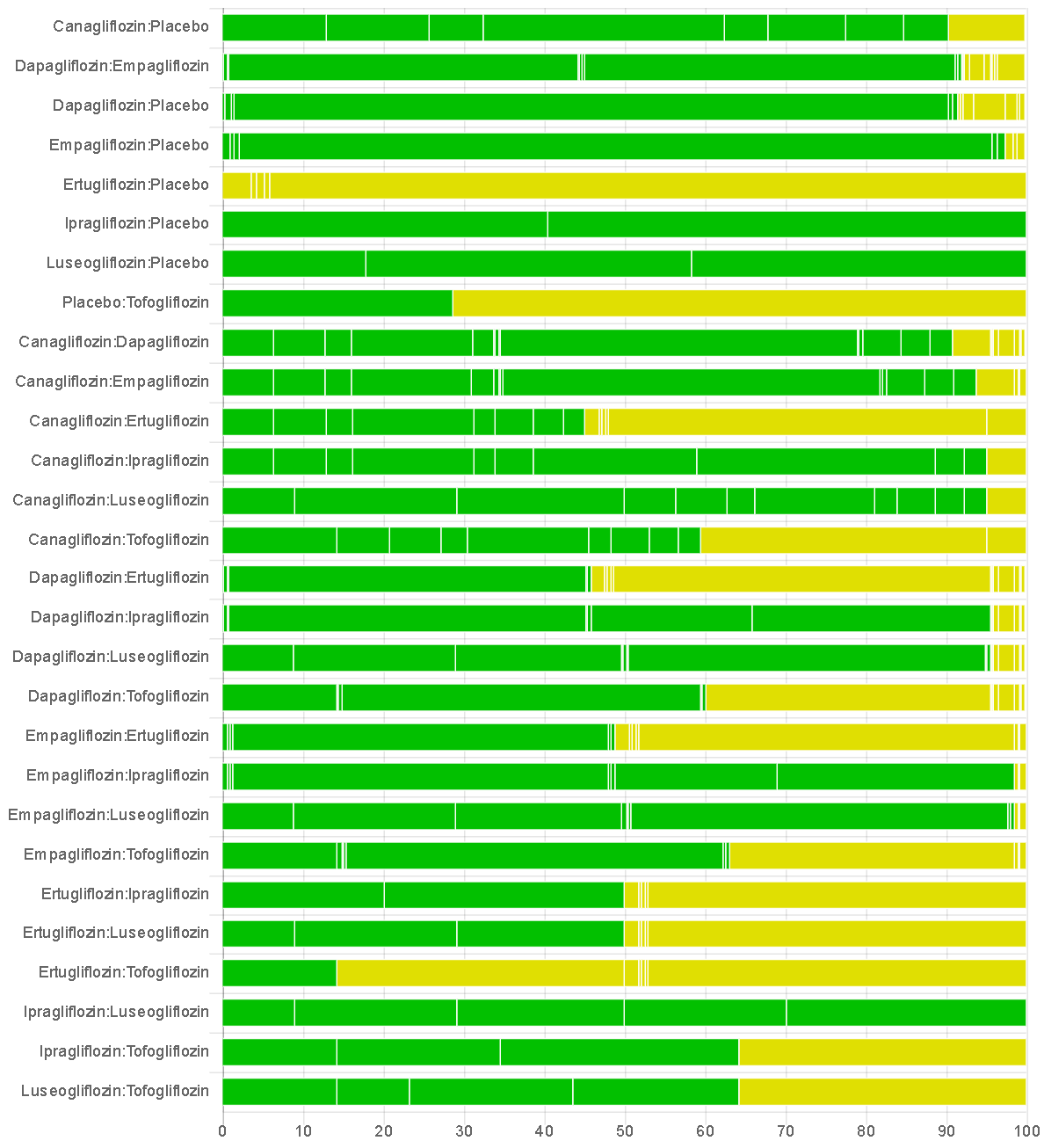


**5.2.4 Renal impairment or failure_RoB_chart**


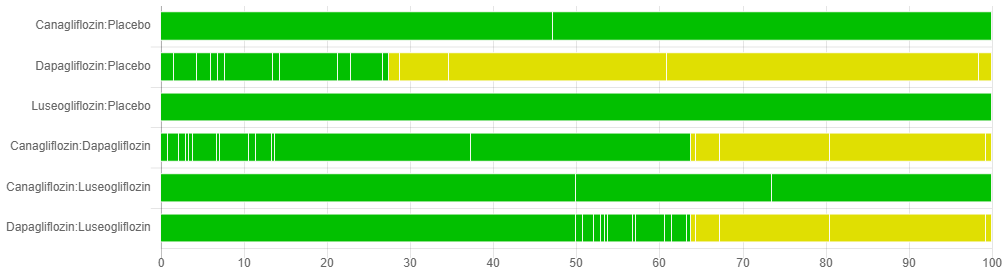


5.2.5 Acute kidney injury_RoB_chart


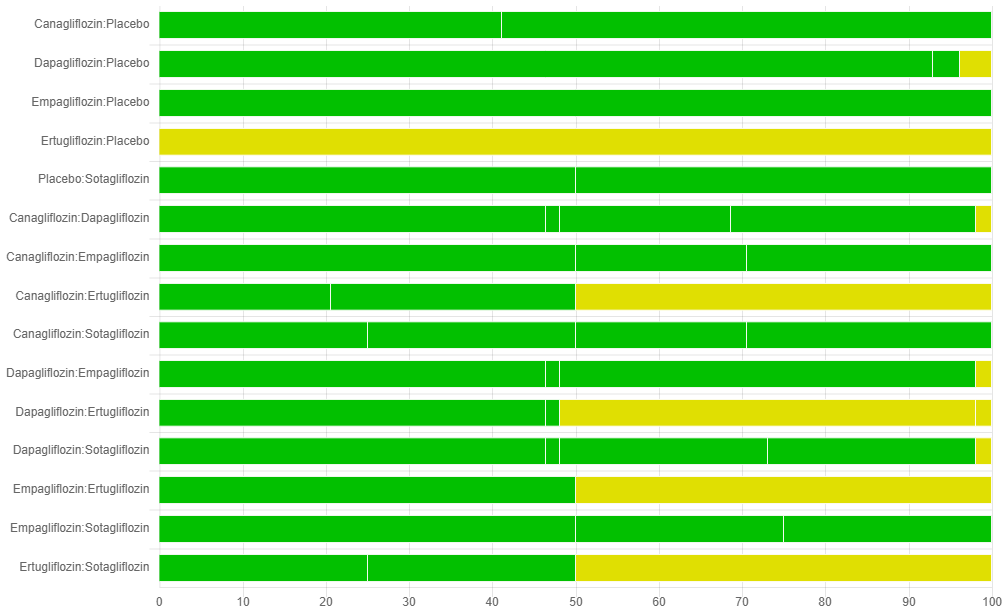


5.2.6 Urinary tract infections_RoB_chart


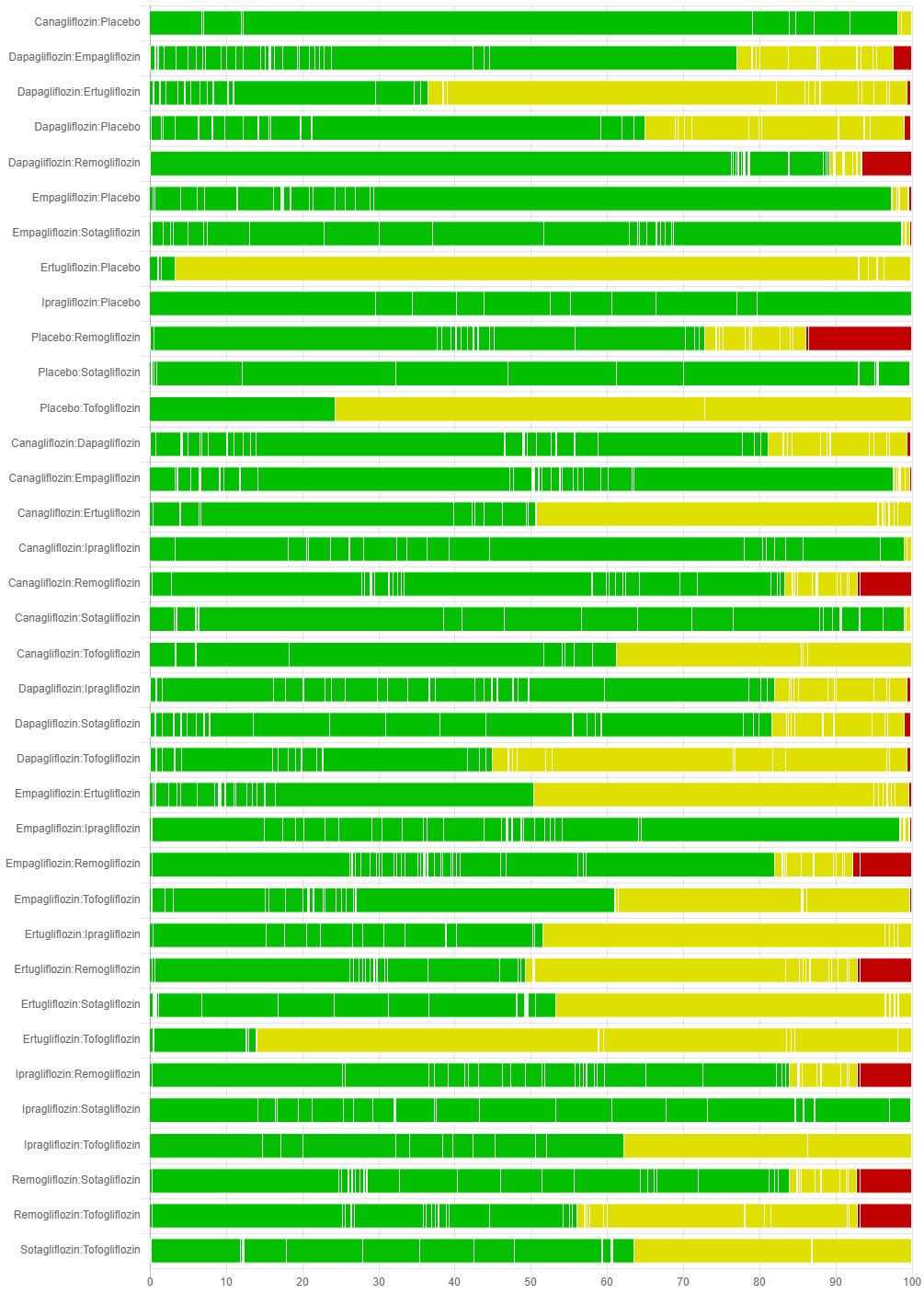


5.2.7 Amputation_RoB_chart


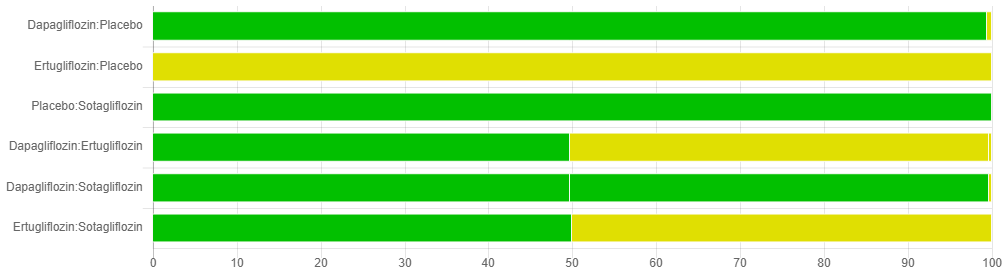


5.2.8 [Diabetic ketoacidosis](javascript:;)_RoB_chart


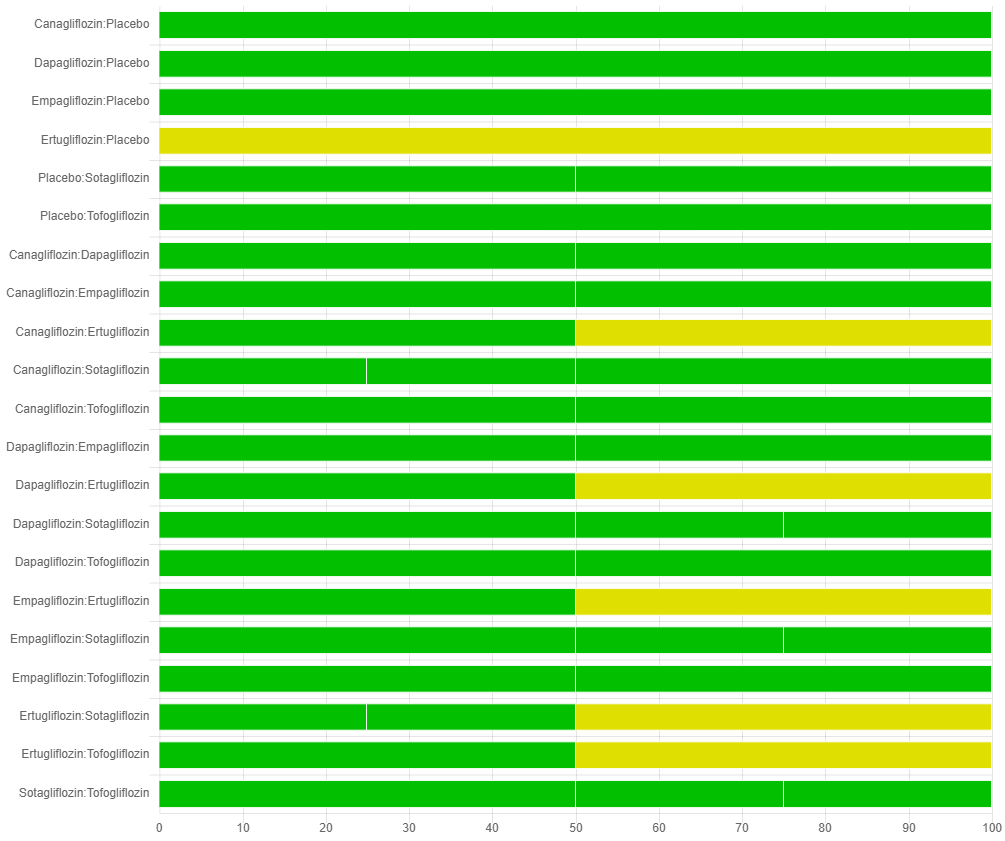


5.2.9 Fracture_RoB_chart


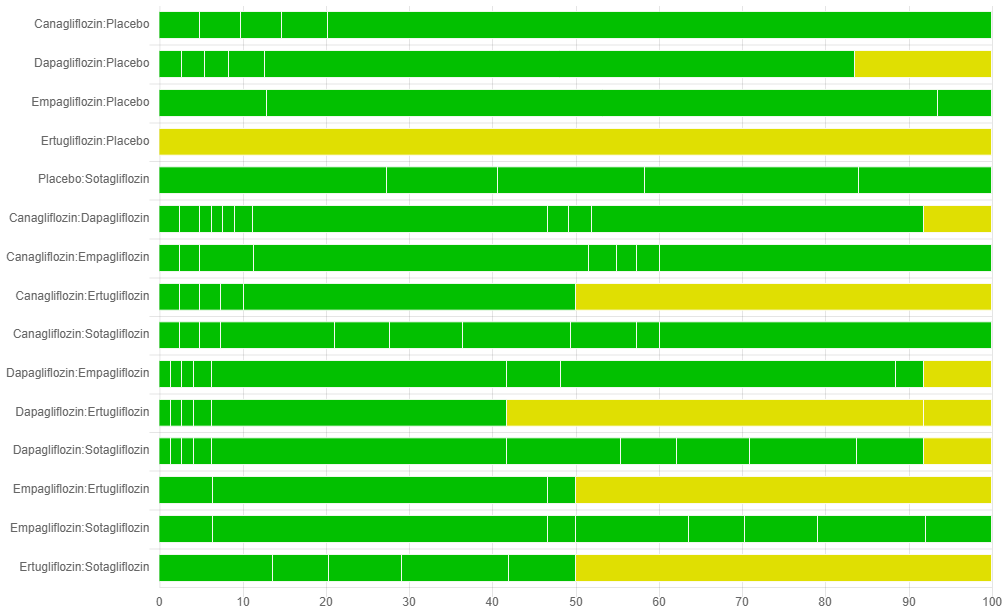


5.2.10 Severe hypoglycemia_RoB_chart


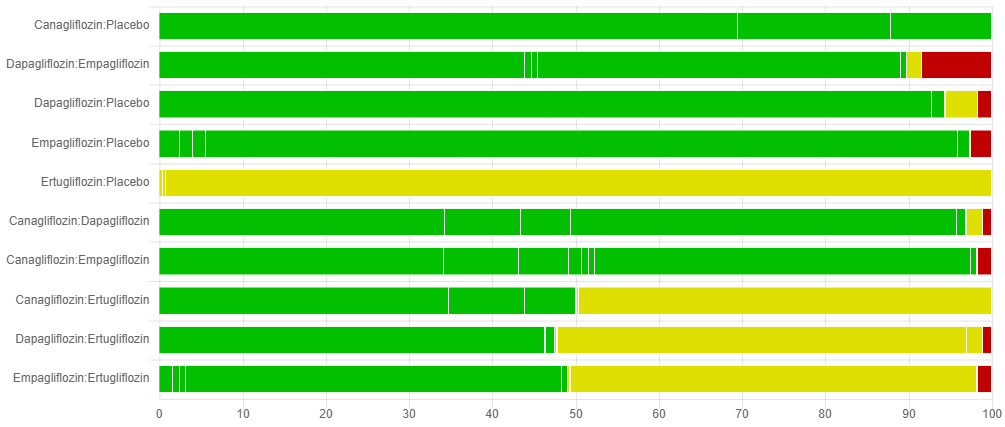
Appendix 6 Trace plot and density plot, and Brooks-Gelman-Rubin diagnosis plot

## 6.1 The trace and density plot of reproductive tract infections


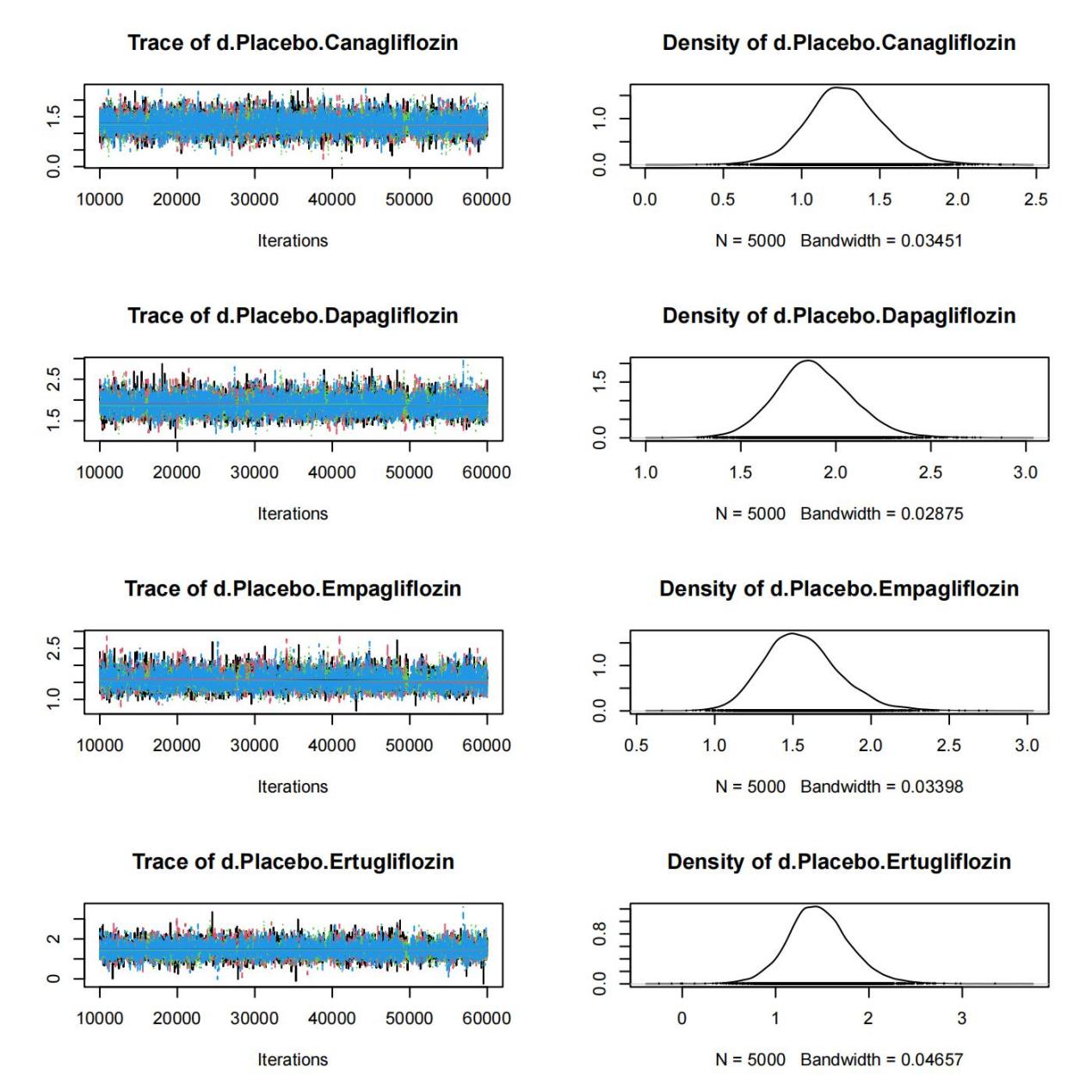

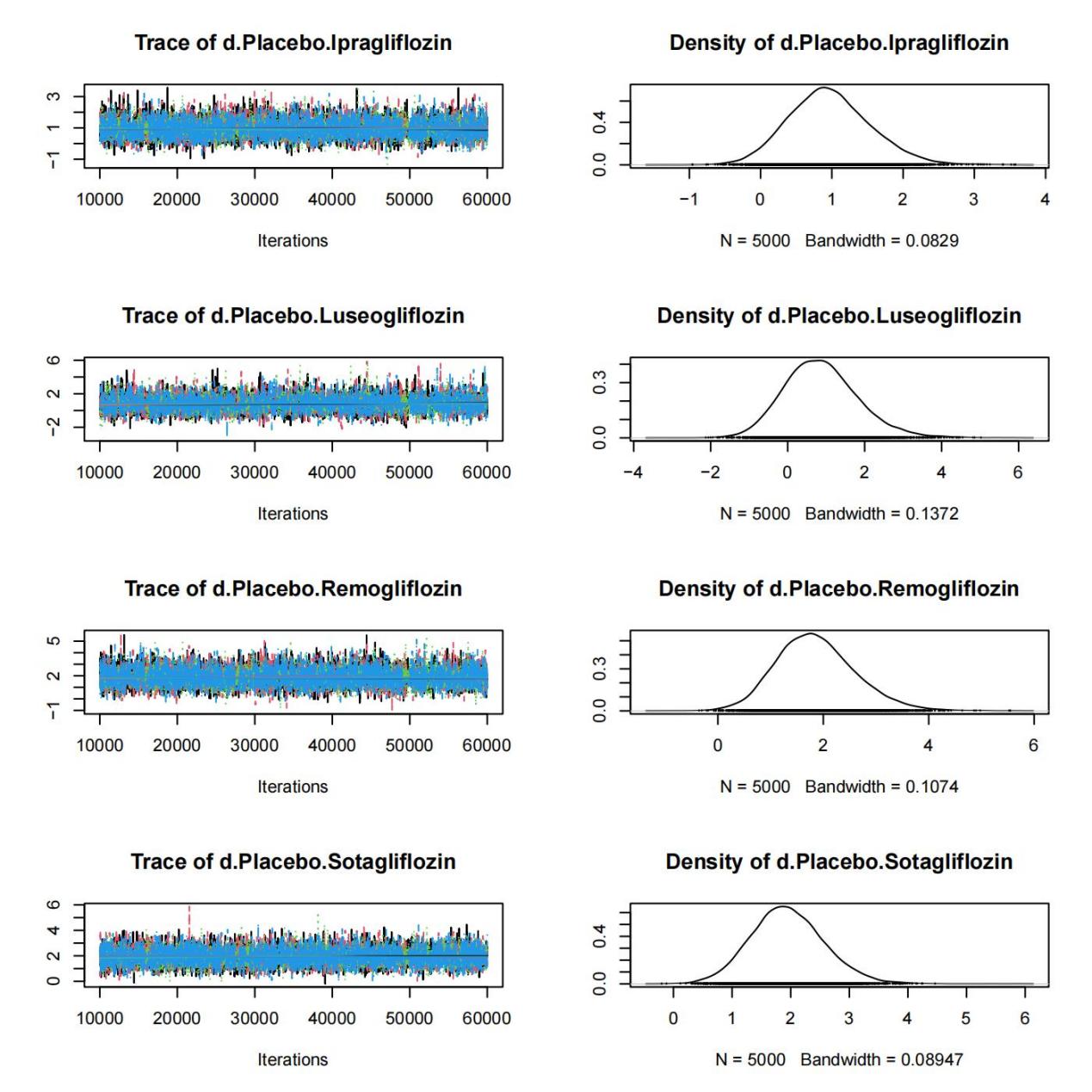

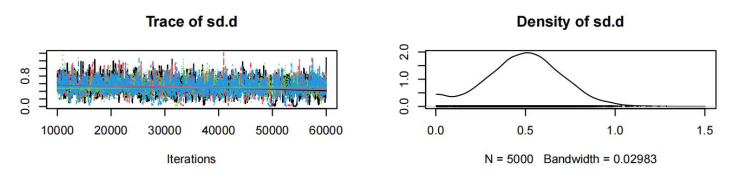


## 6.1 The Brooks-Gelman-Rubin diagnosis plot of reproductive tract infections


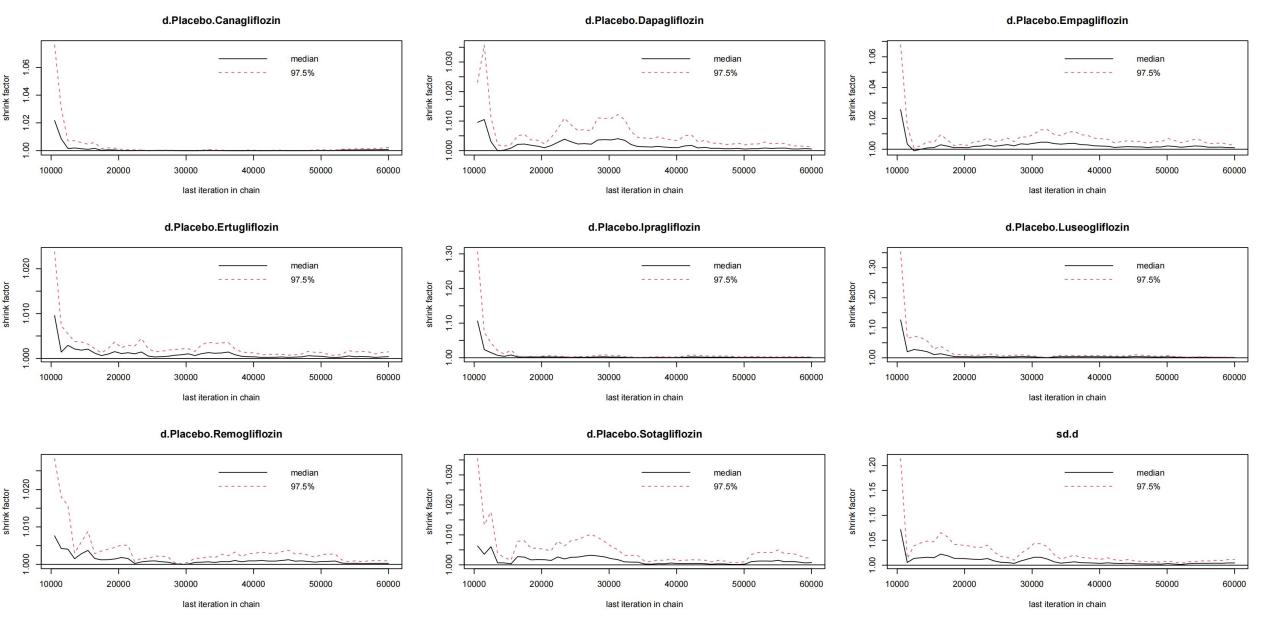


## 6.2 The trace and density plot of pollakiuria


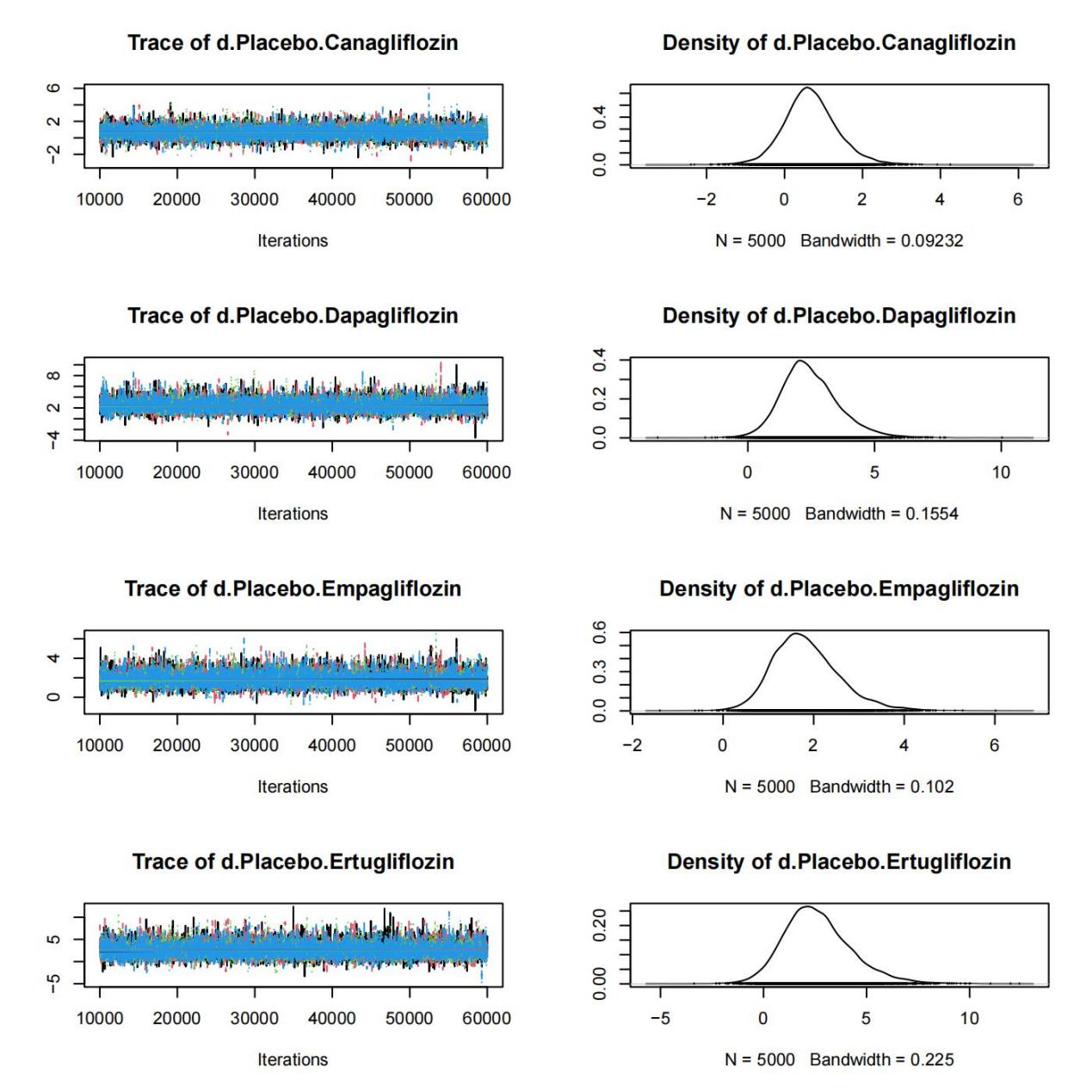

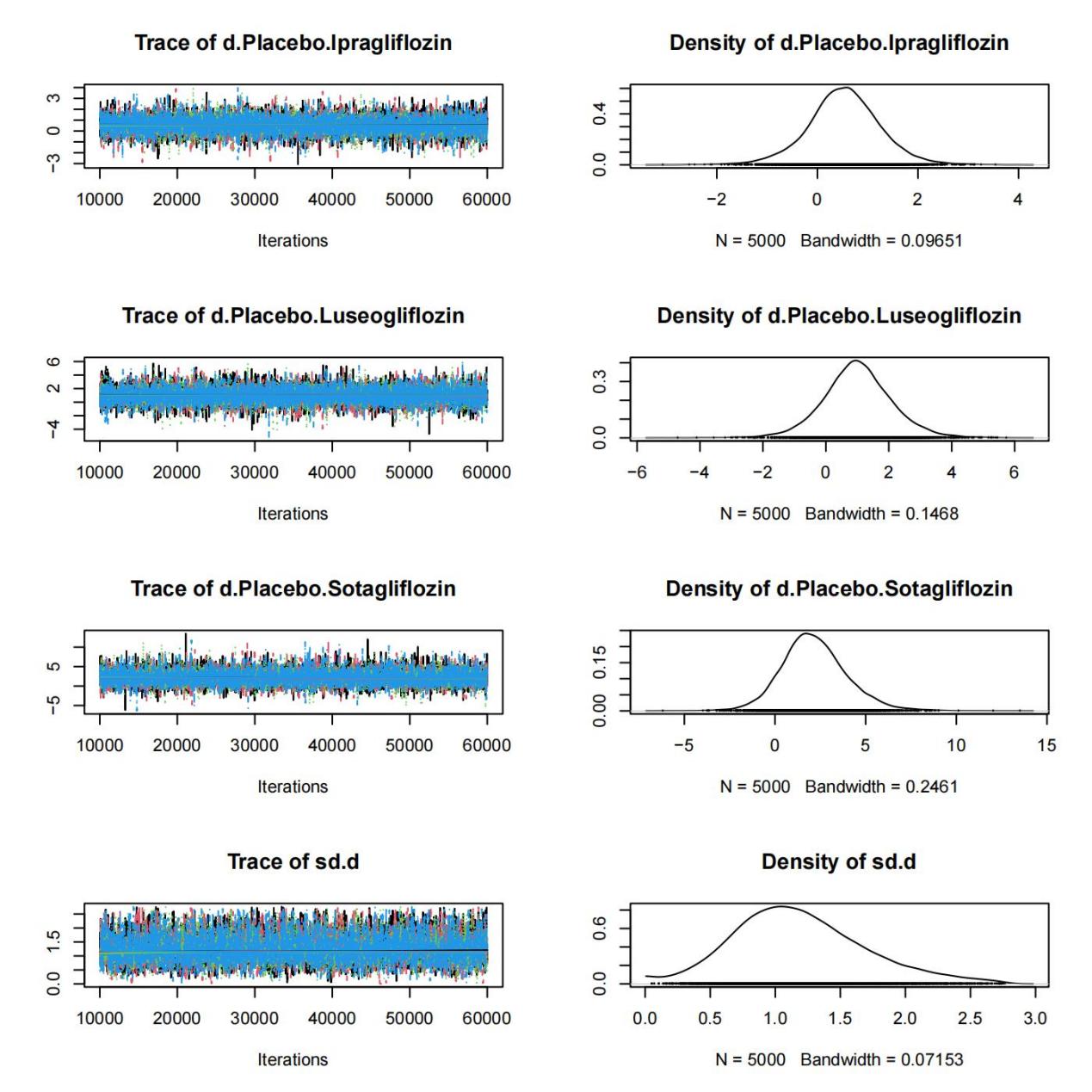


6.2 The Brooks-Gelman-Rubin diagnosis plot of pollakiuria


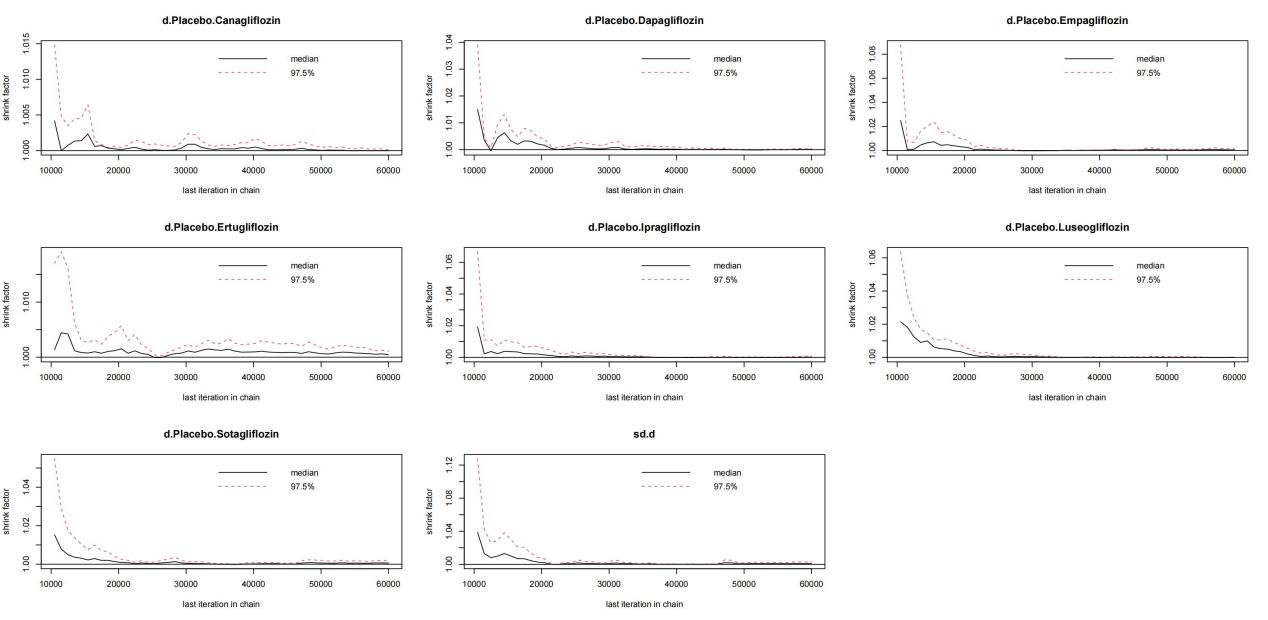


## **6.3 The trace and density plot of hypovolemia**


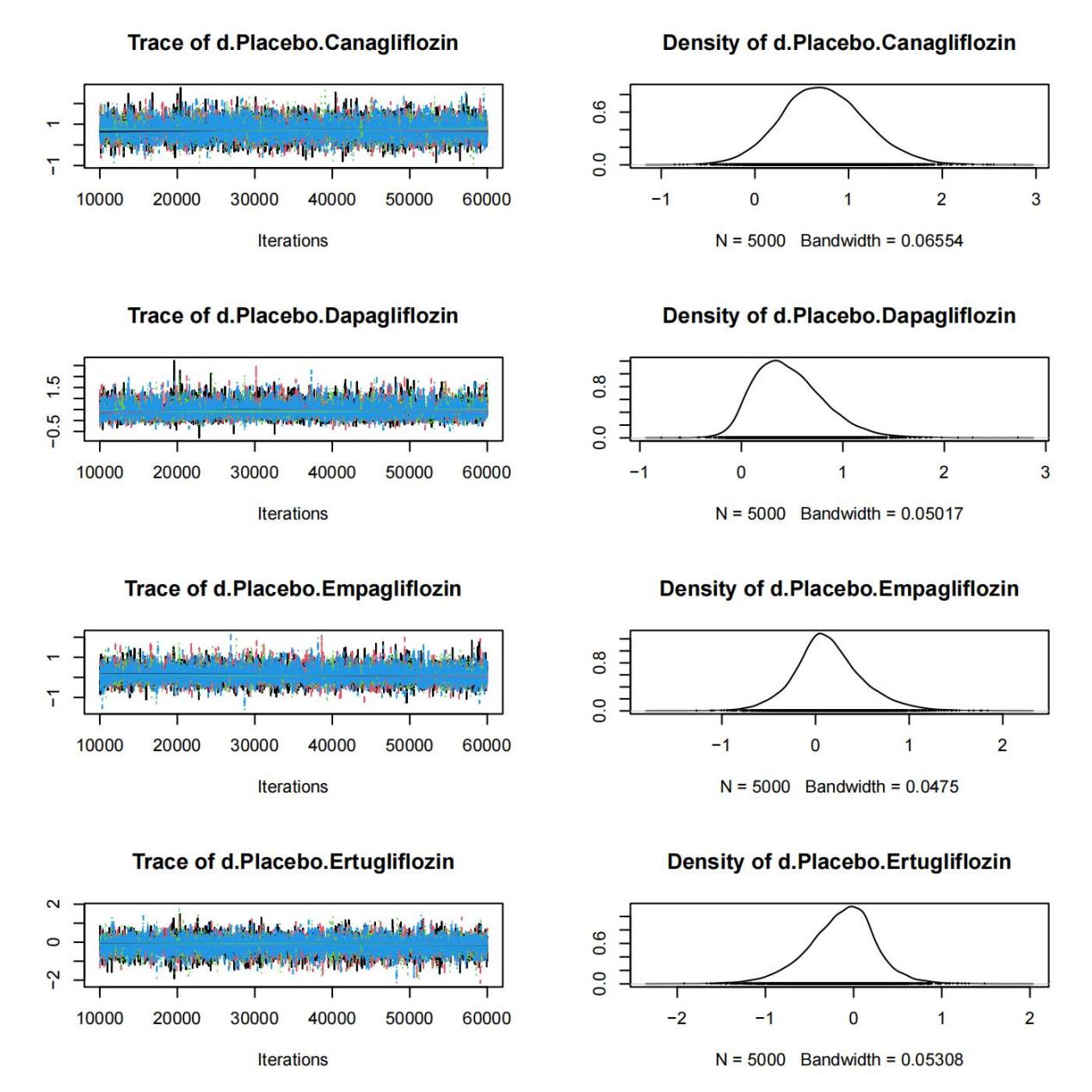

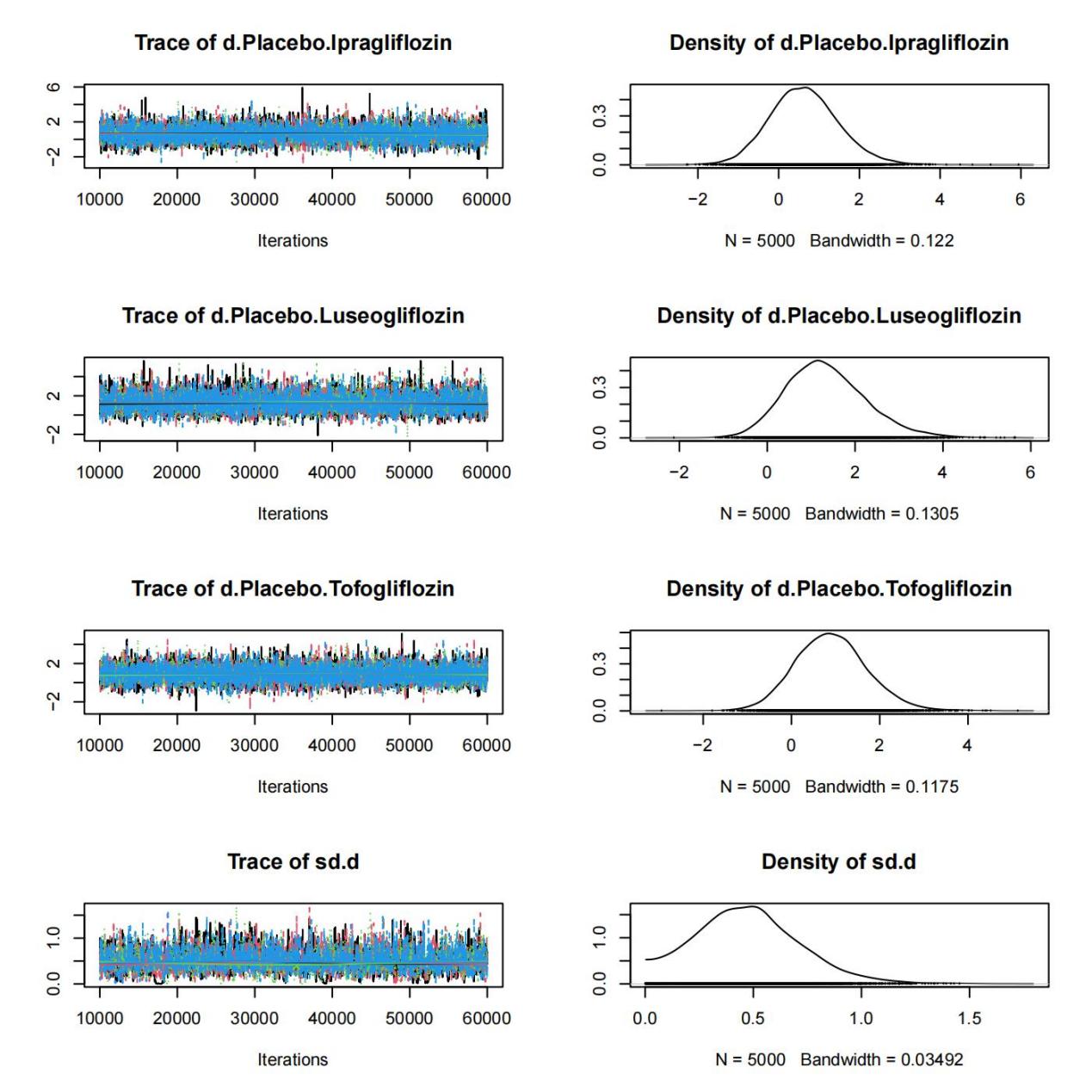


## 6.3 The Brooks-Gelman-Rubin diagnosis plot of hypovolemia

##
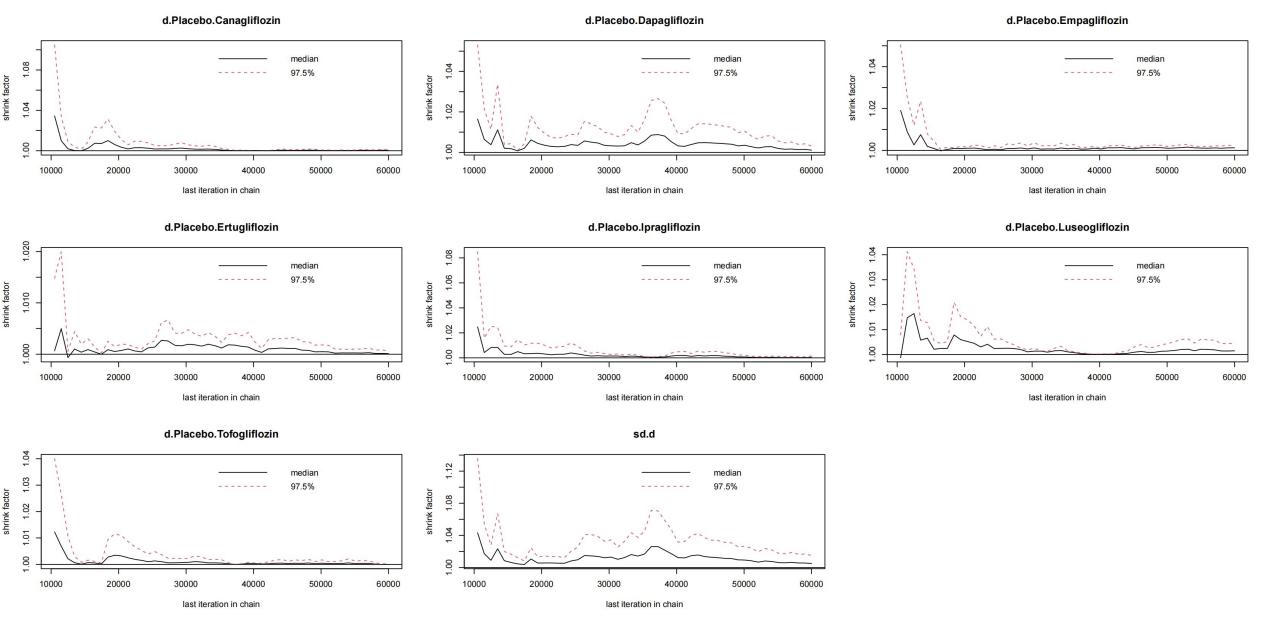


## 6.4 The trace and density plot of renal impairment or failure


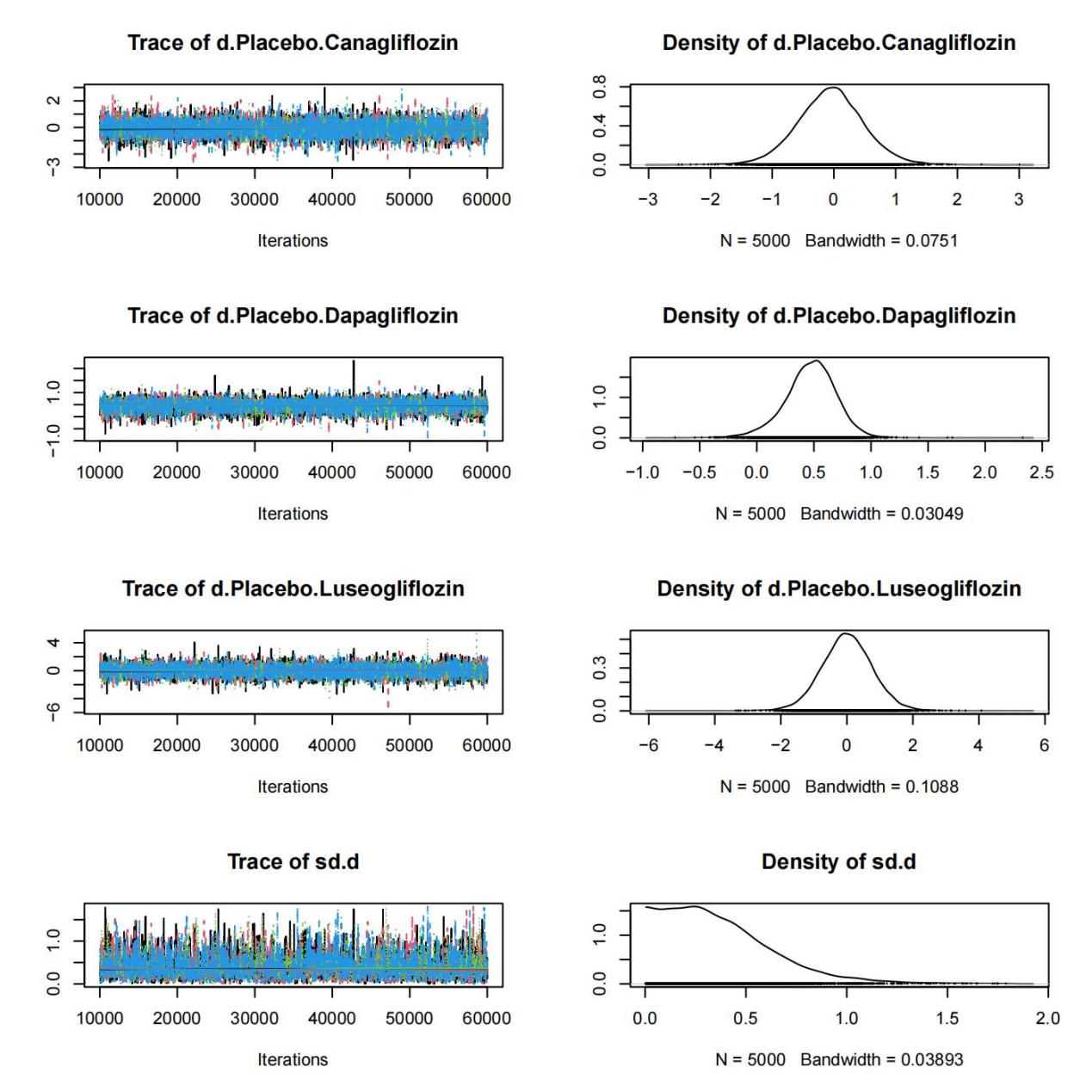


## 6.4 The Brooks-Gelman-Rubin diagnosis plot of renal impairment or failure


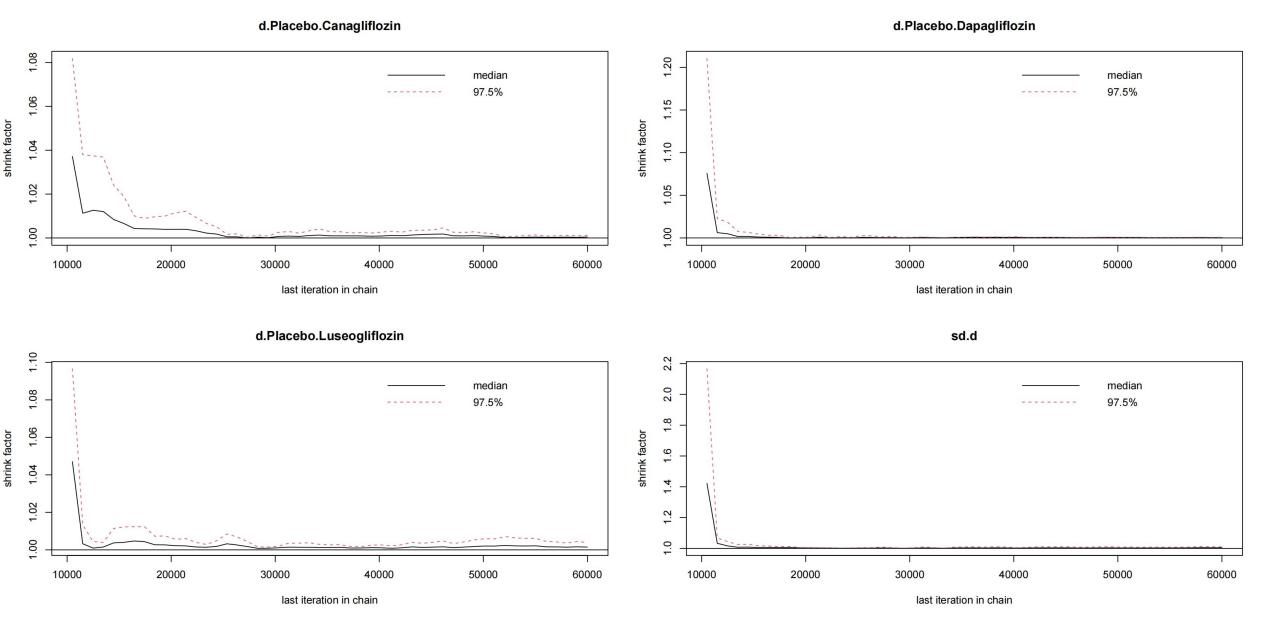


## 6.5 The trace and density plot of acute kidney failure


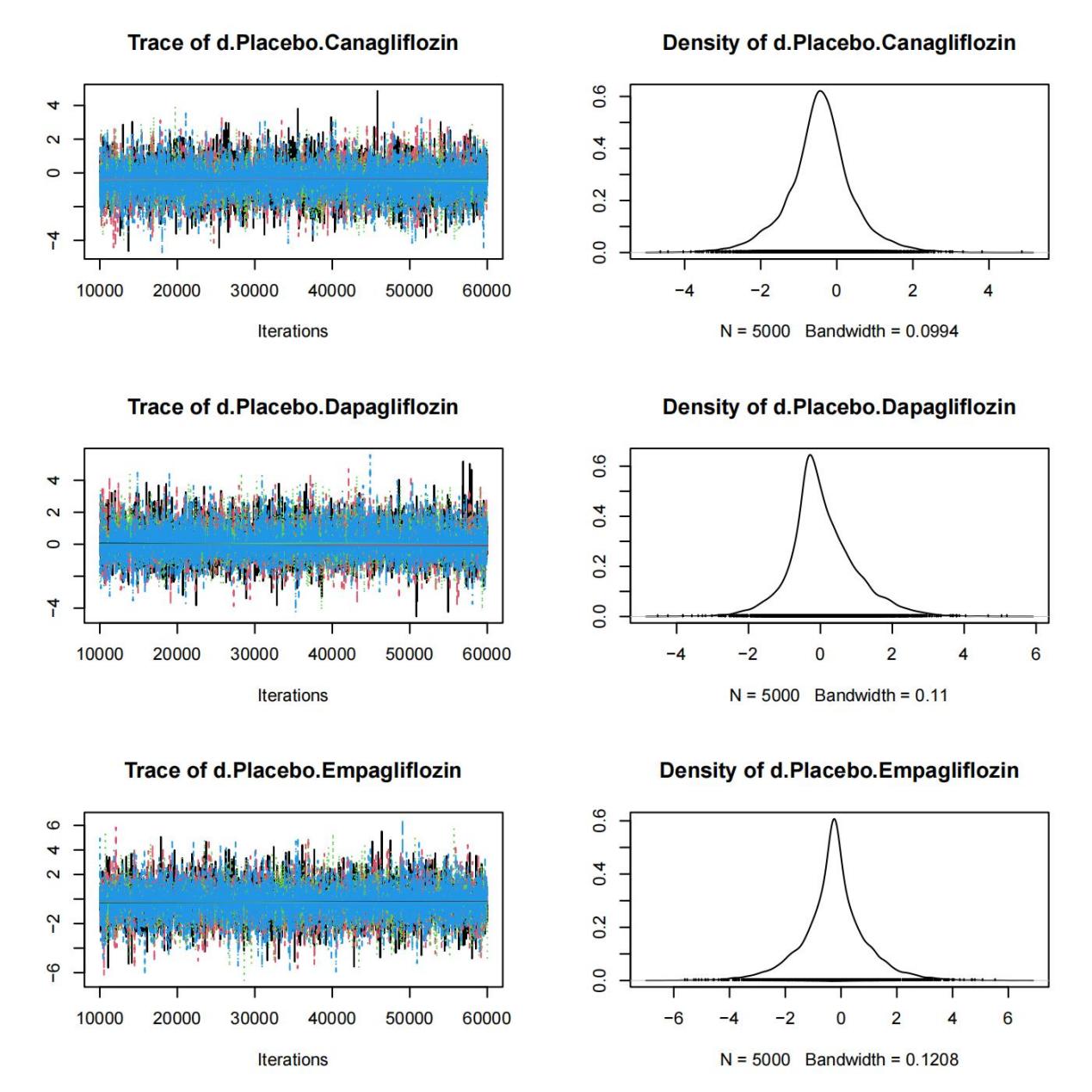

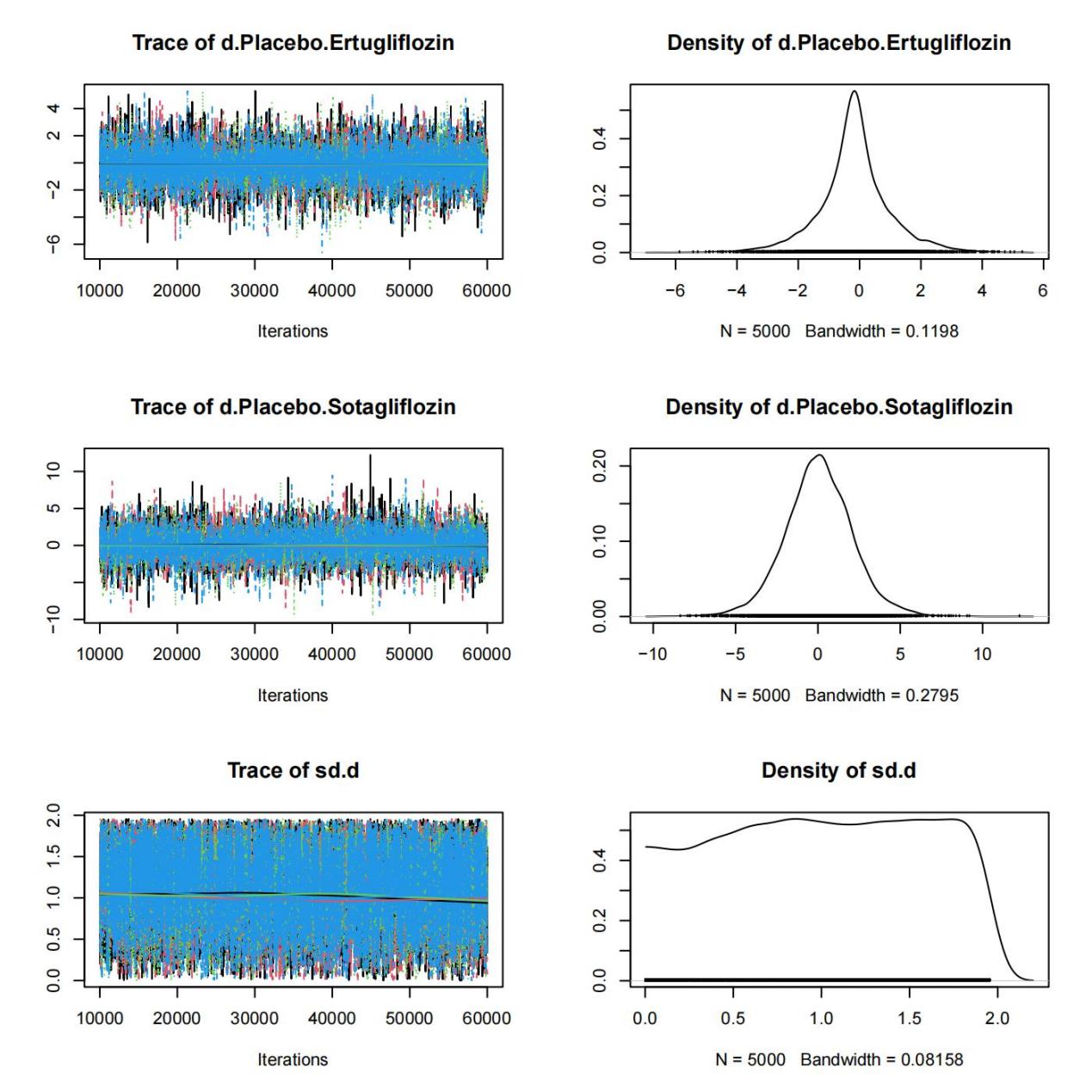


## 6.5 The Brooks-Gelman-Rubin diagnosis plot of acute kidney failure

##
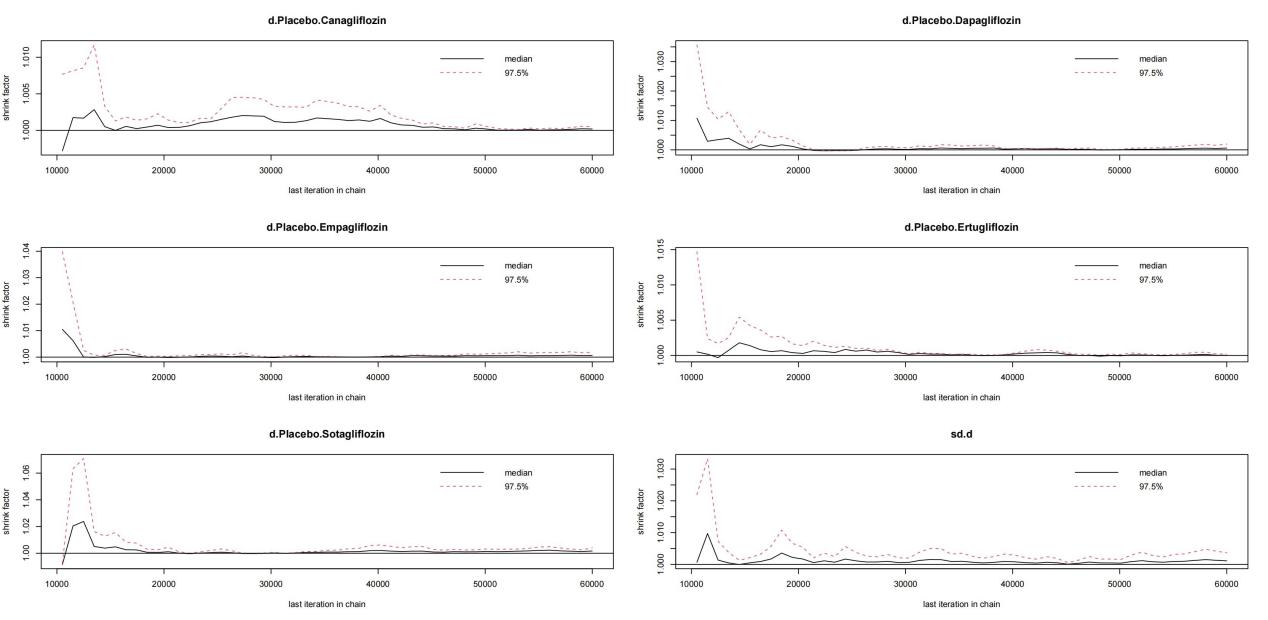


## **6.6 The trace and density plot of [urinary tract infection](javascript:;)s**
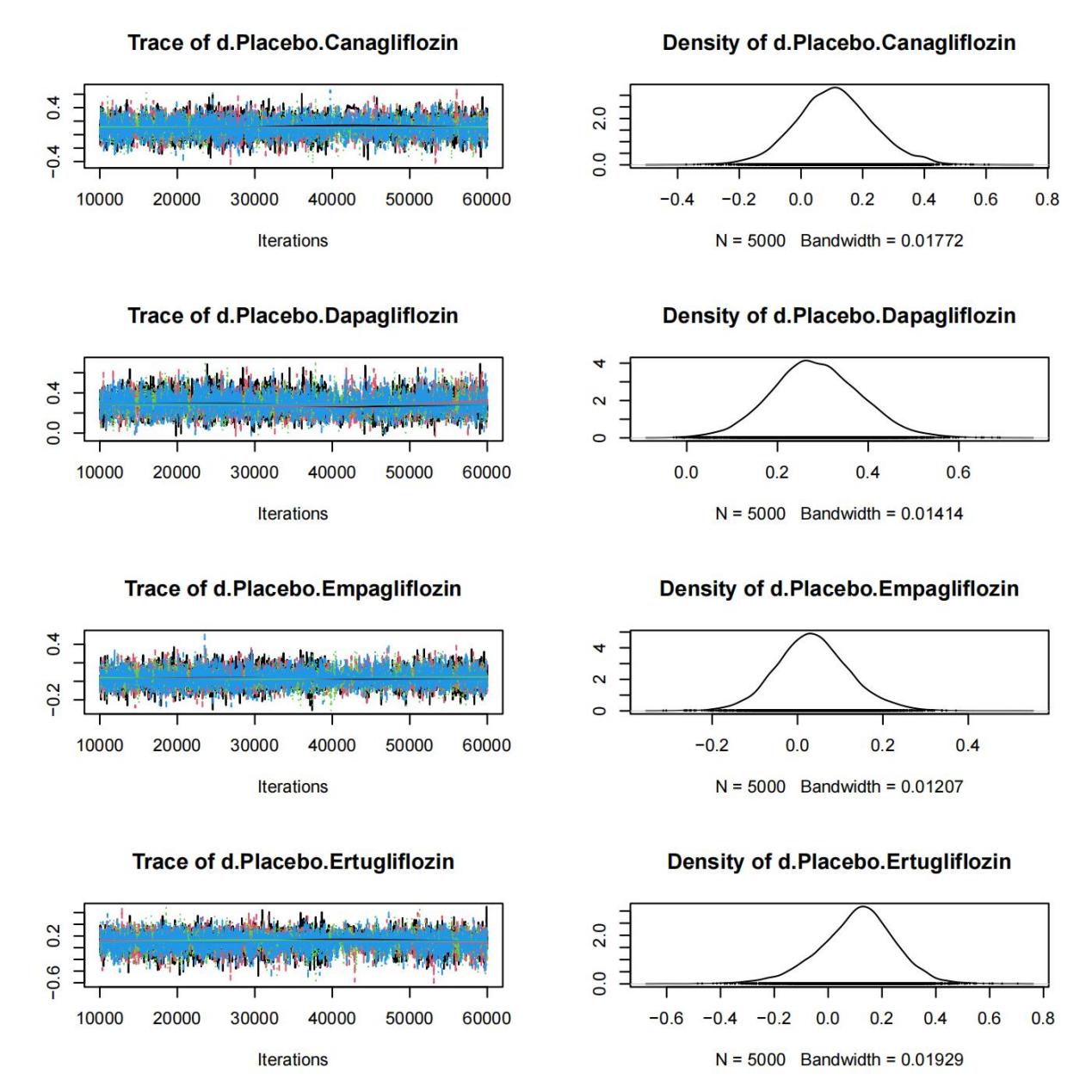

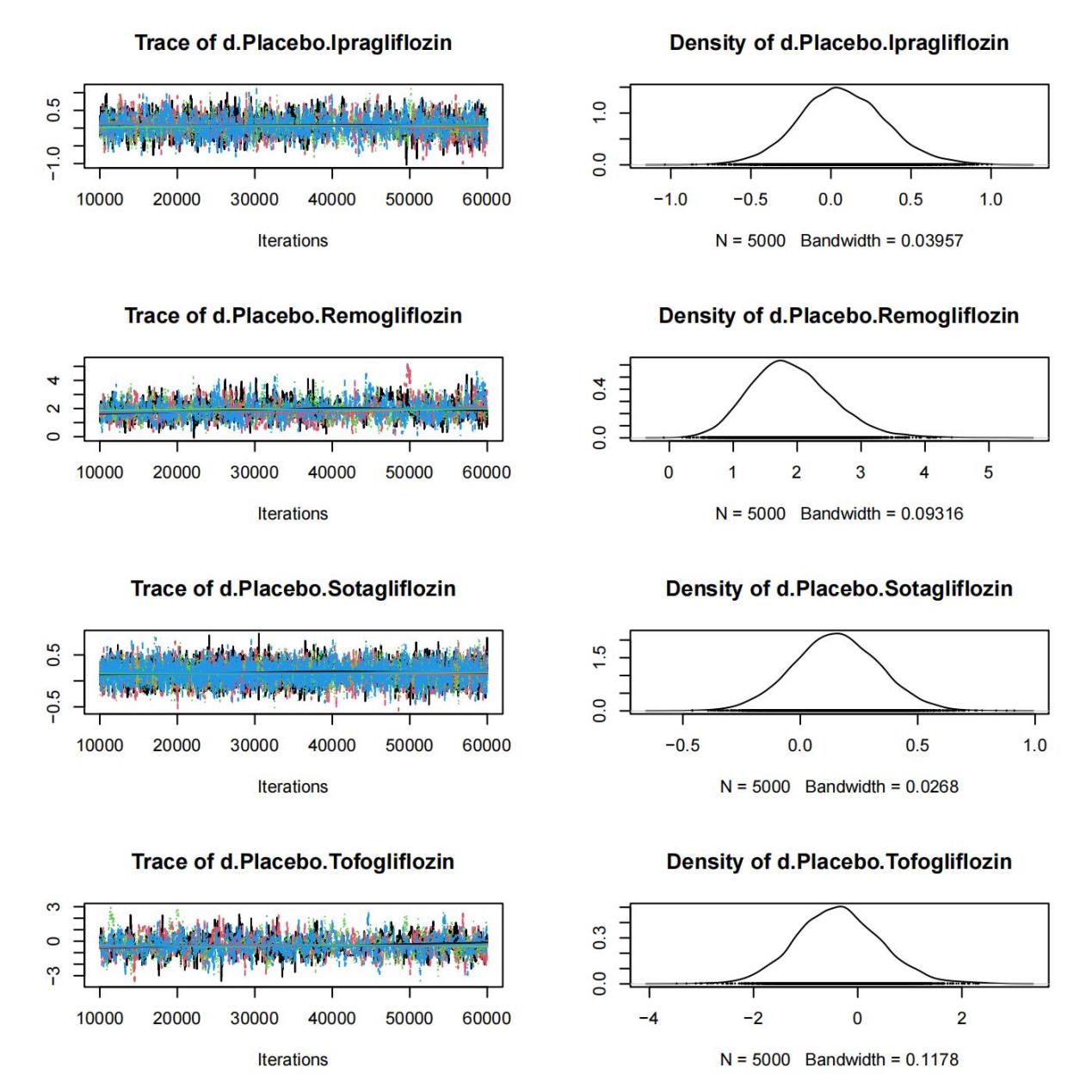

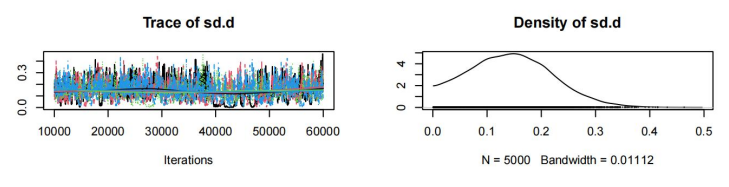


## 6.6 The Brooks-Gelman-Rubin diagnosis plot of [urinary tract infection](javascript:;)s

##
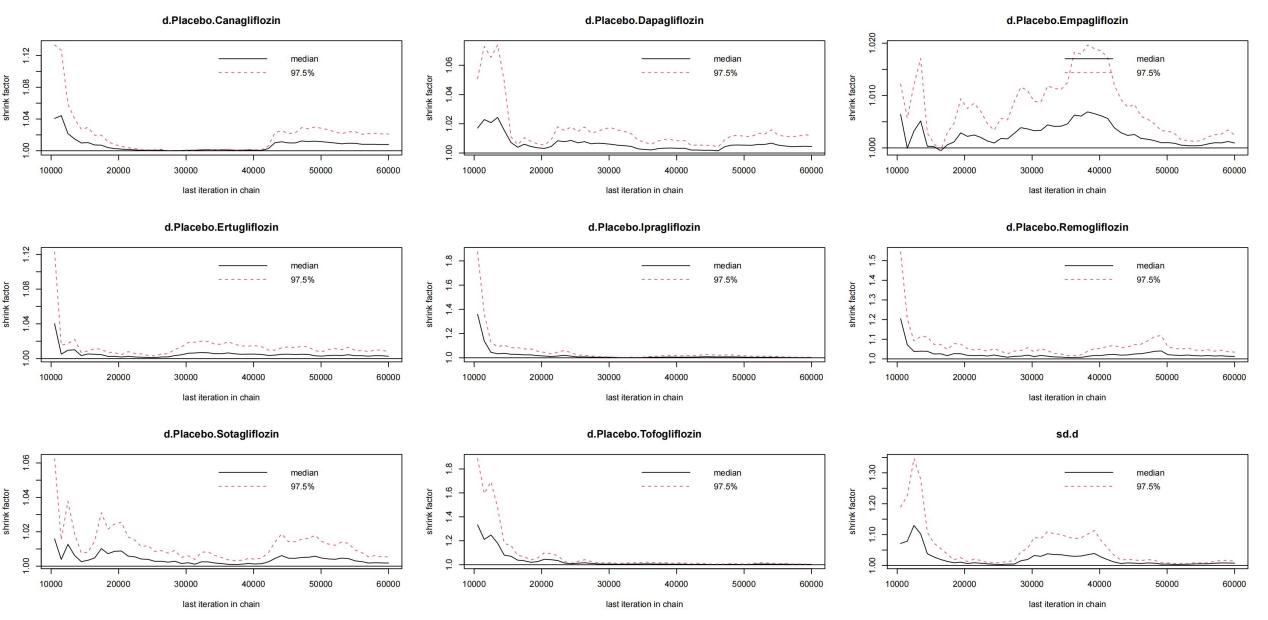
**6.7 The trace and density plot of [diabetic ketoacidosis](javascript:;)**


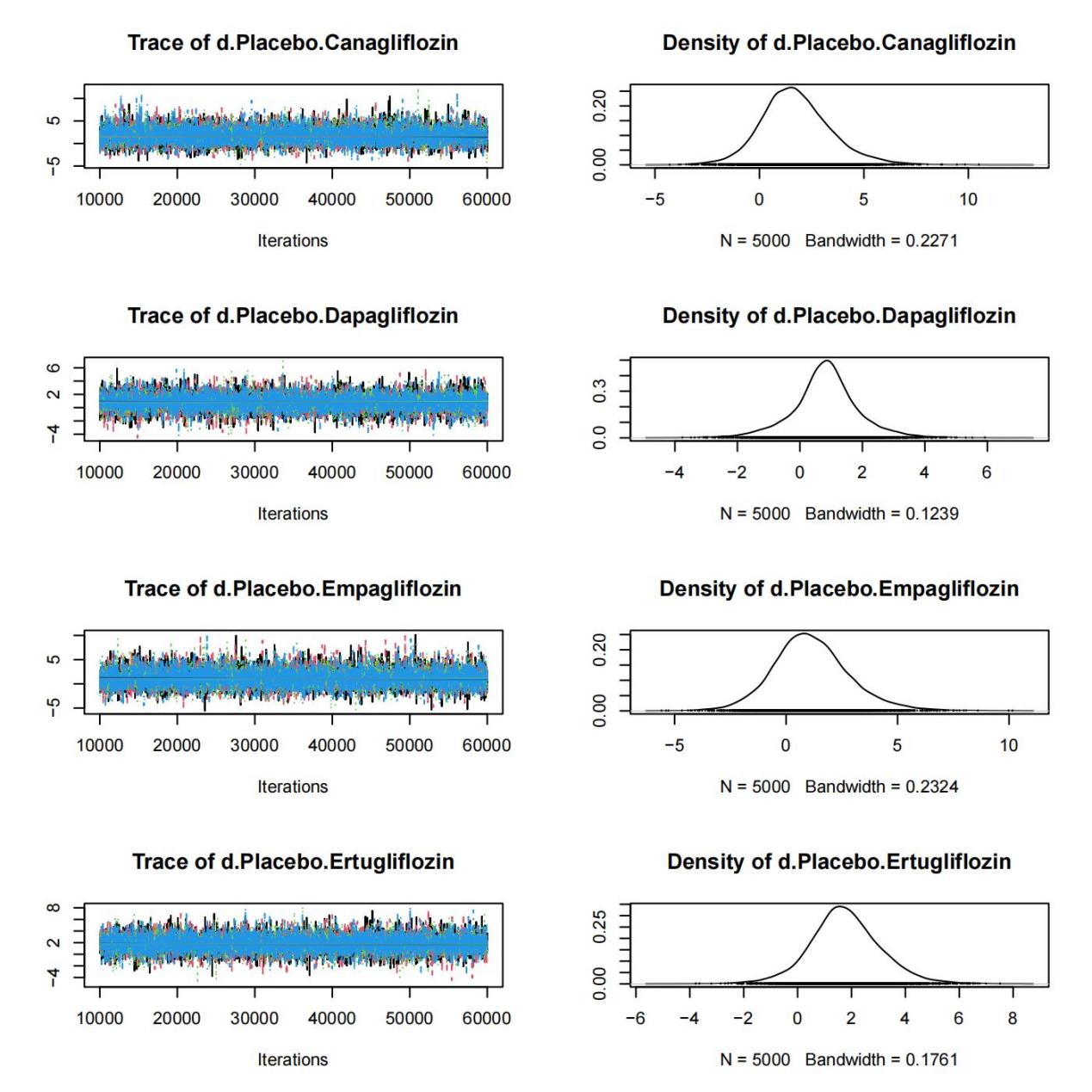

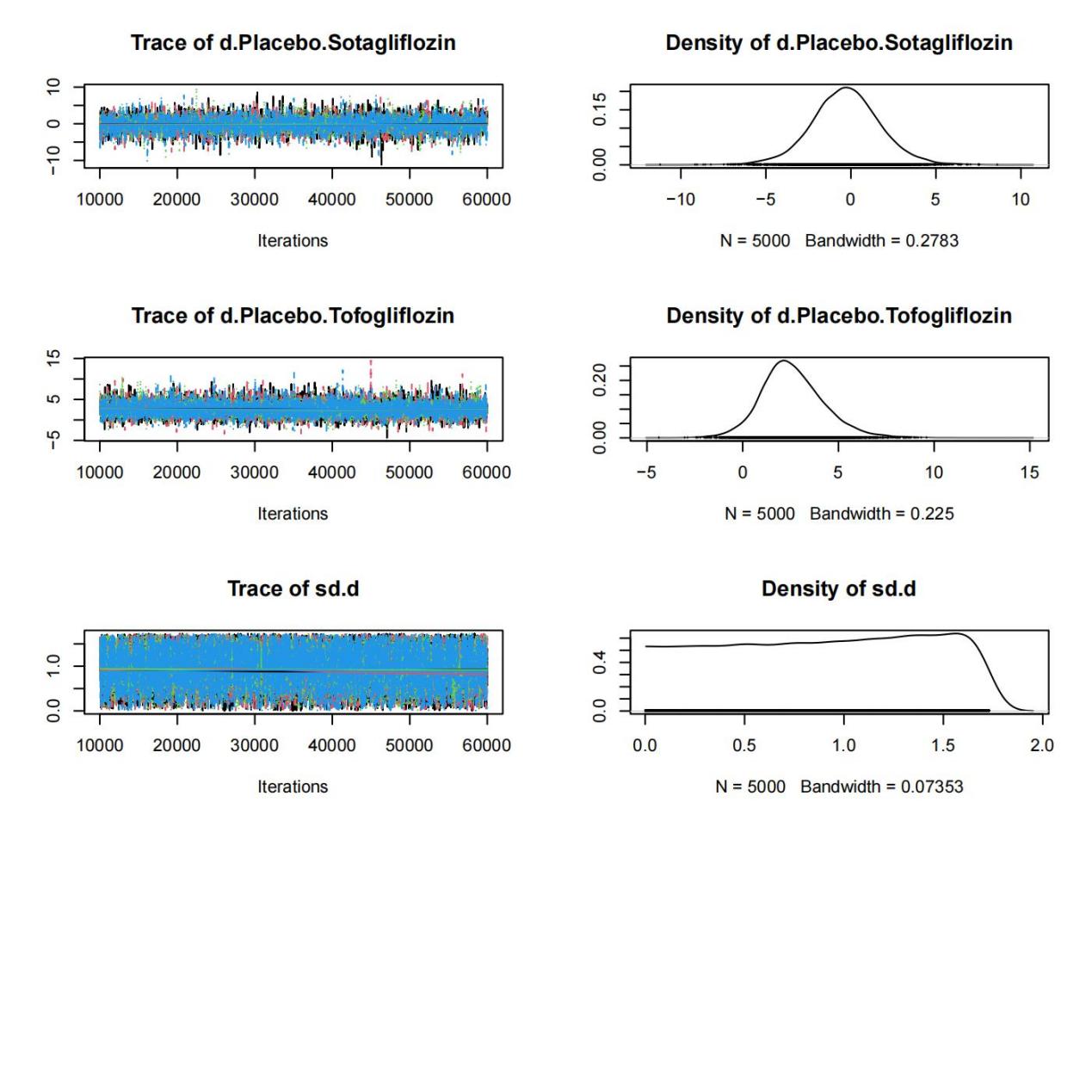
6.7 The Brooks-Gelman-Rubin diagnosis plot of [diabetic ketoacidosis](javascript:;)


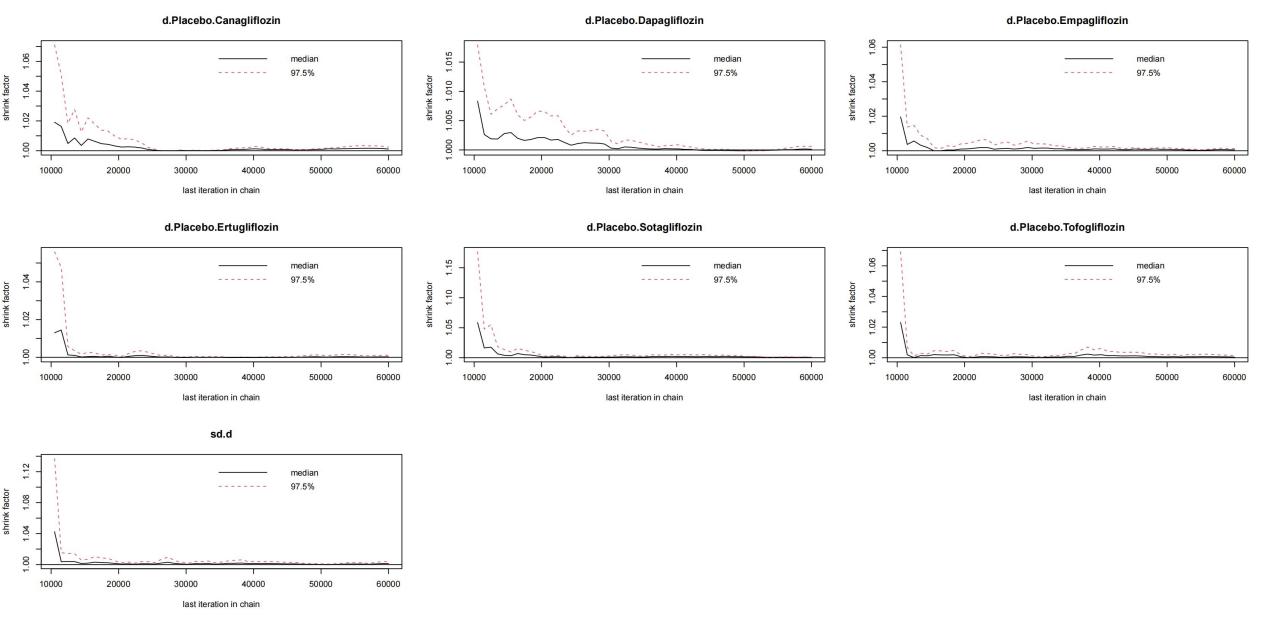


6.8 The trace and density plot of [amputation](javascript:;)


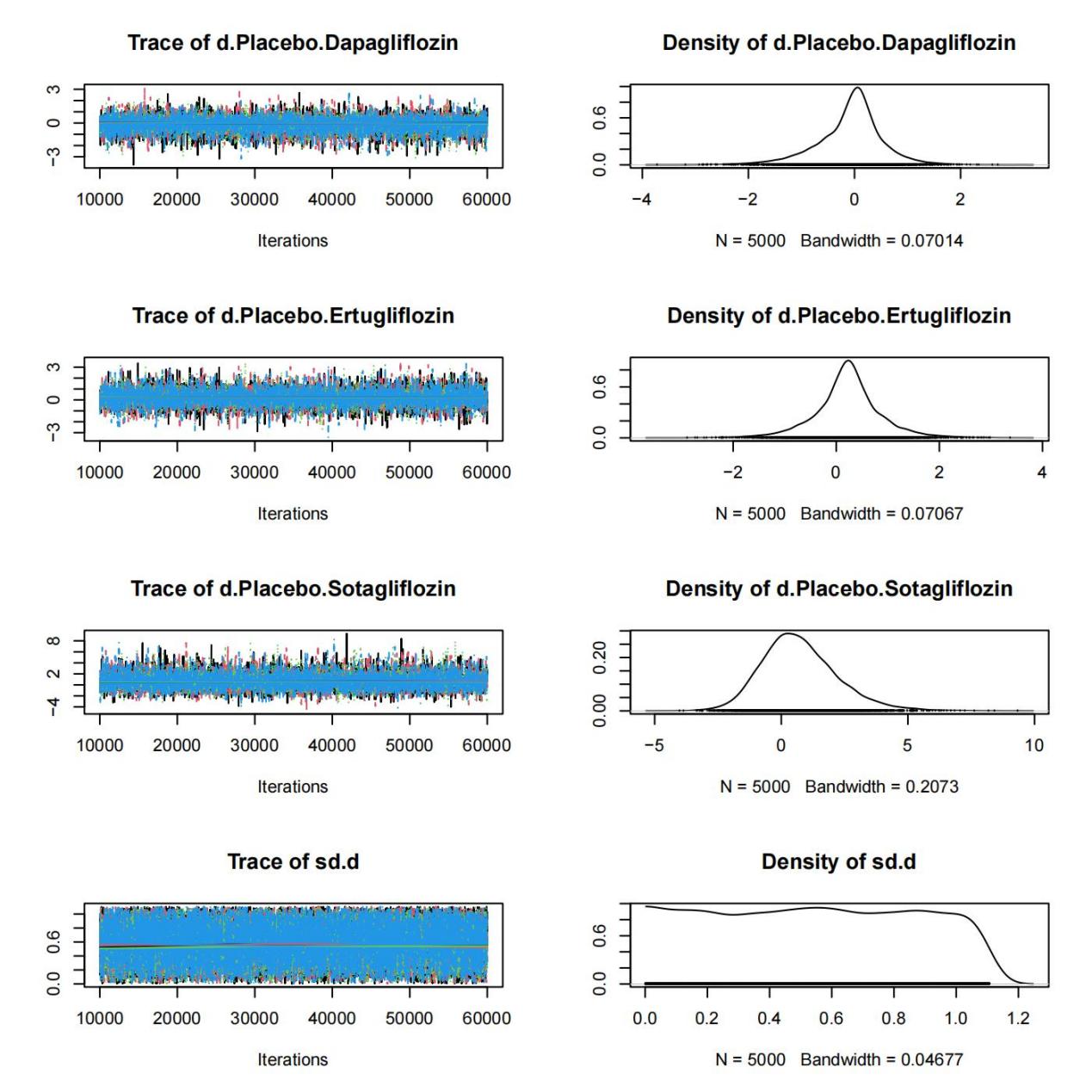


## 6.8 The Brooks-Gelman-Rubin diagnosis plot of [amputation](javascript:;)


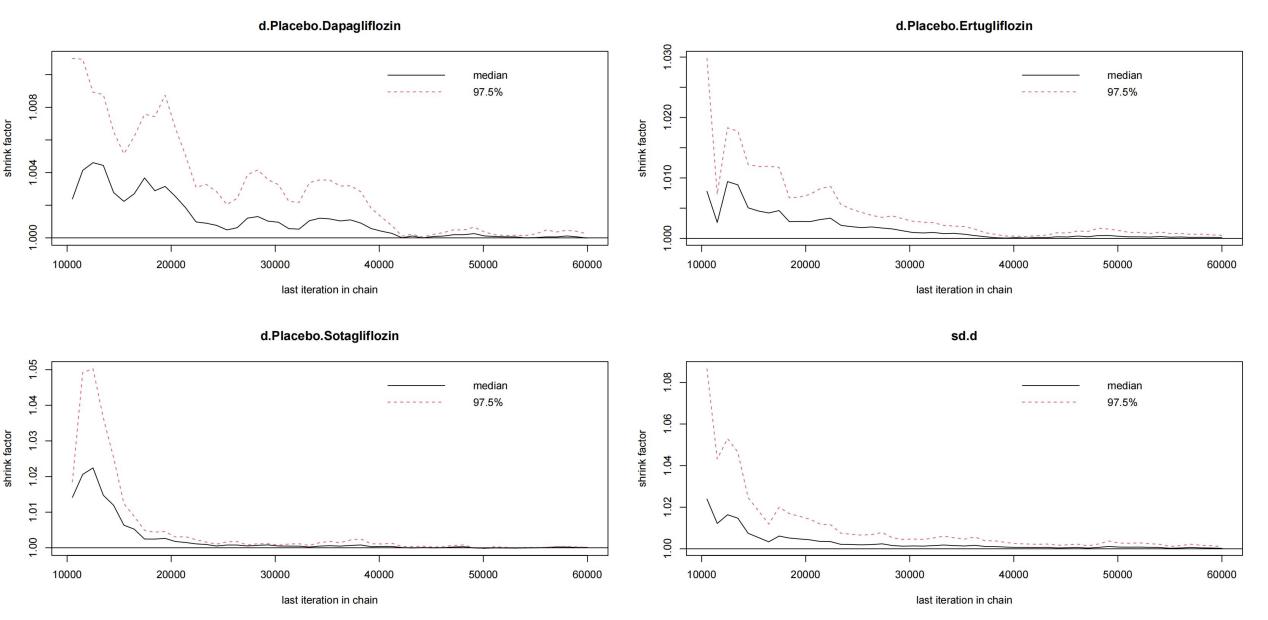
6.9 The trace and density plot of fracture


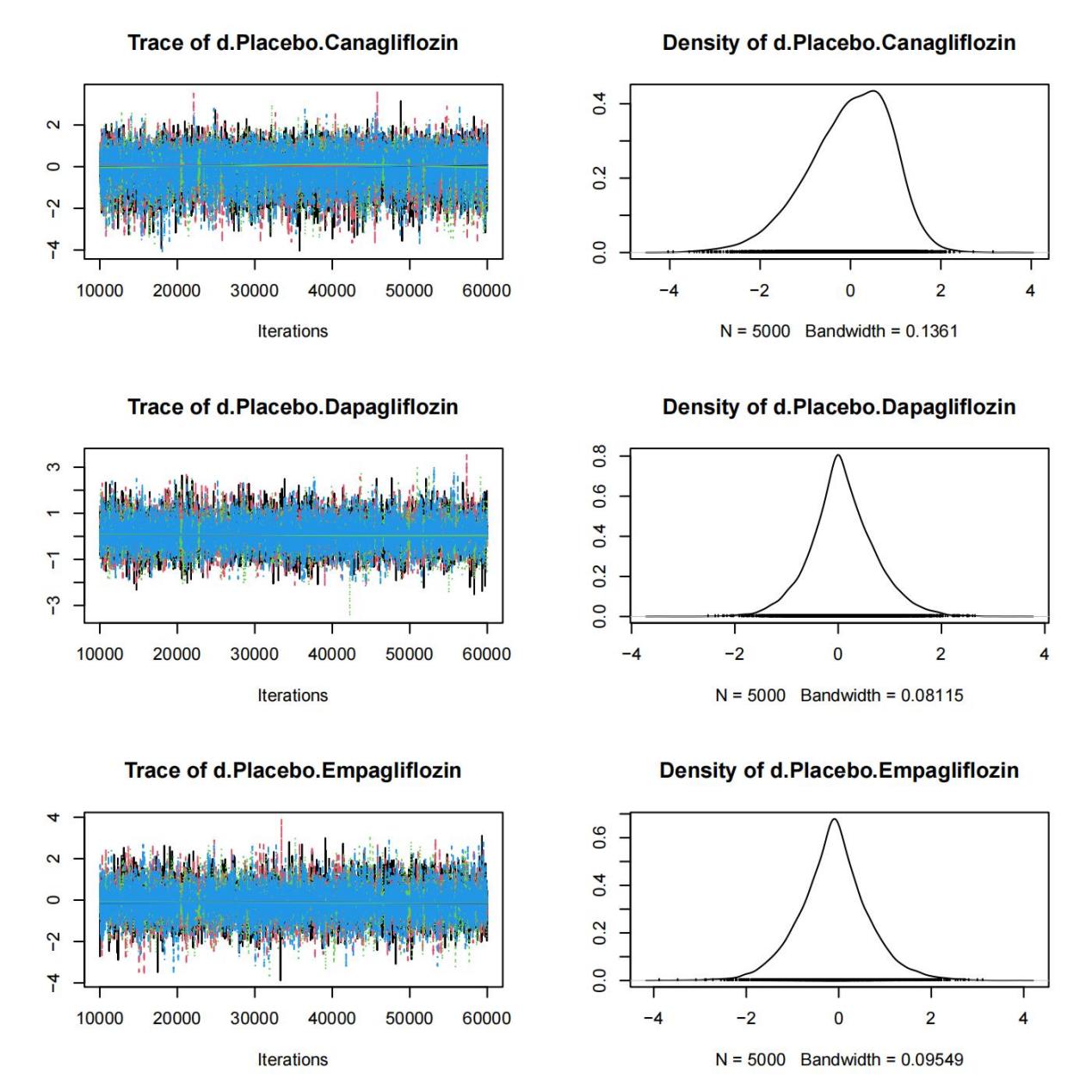

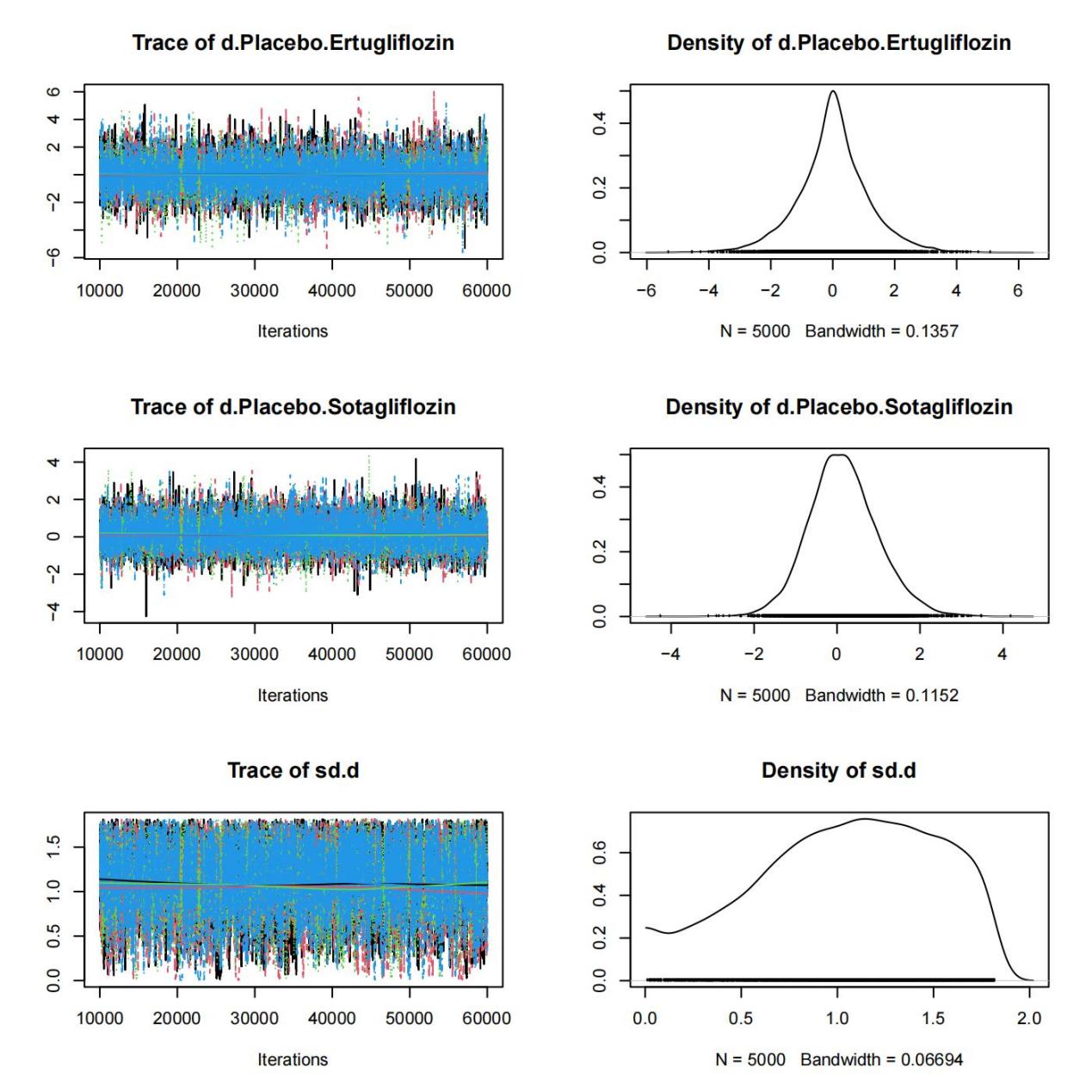


## 6.9 The Brooks-Gelman-Rubin diagnosis plot of fracture


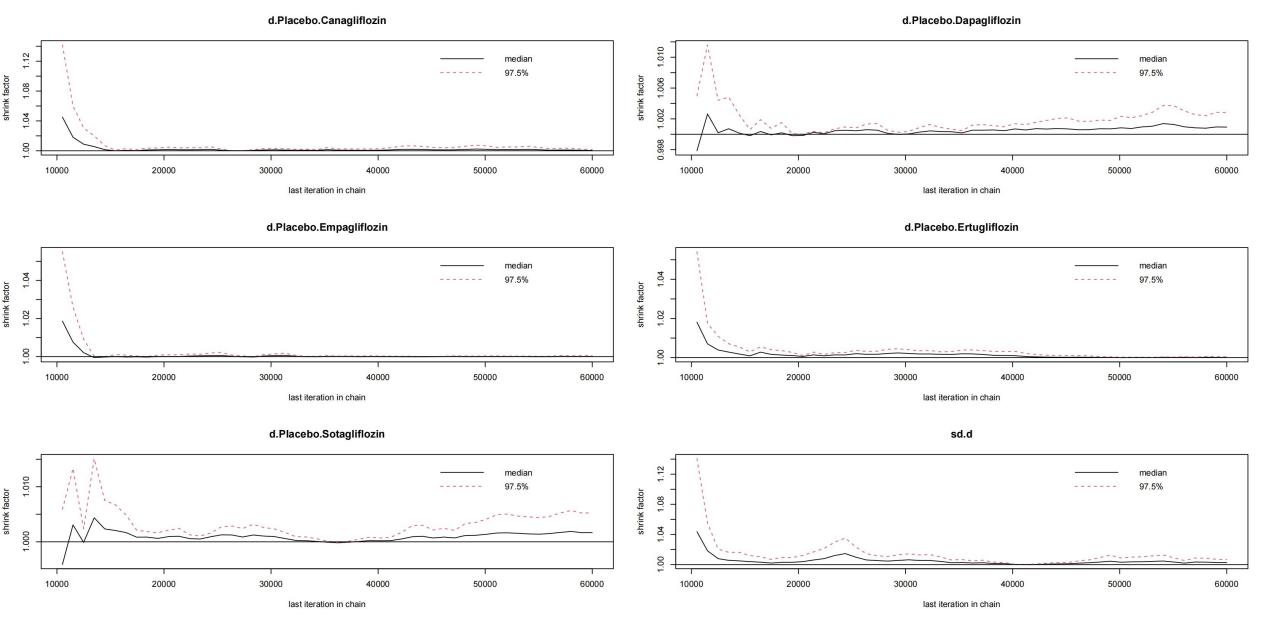


## 6.10 The trace and density plot of severe hypoglycemia


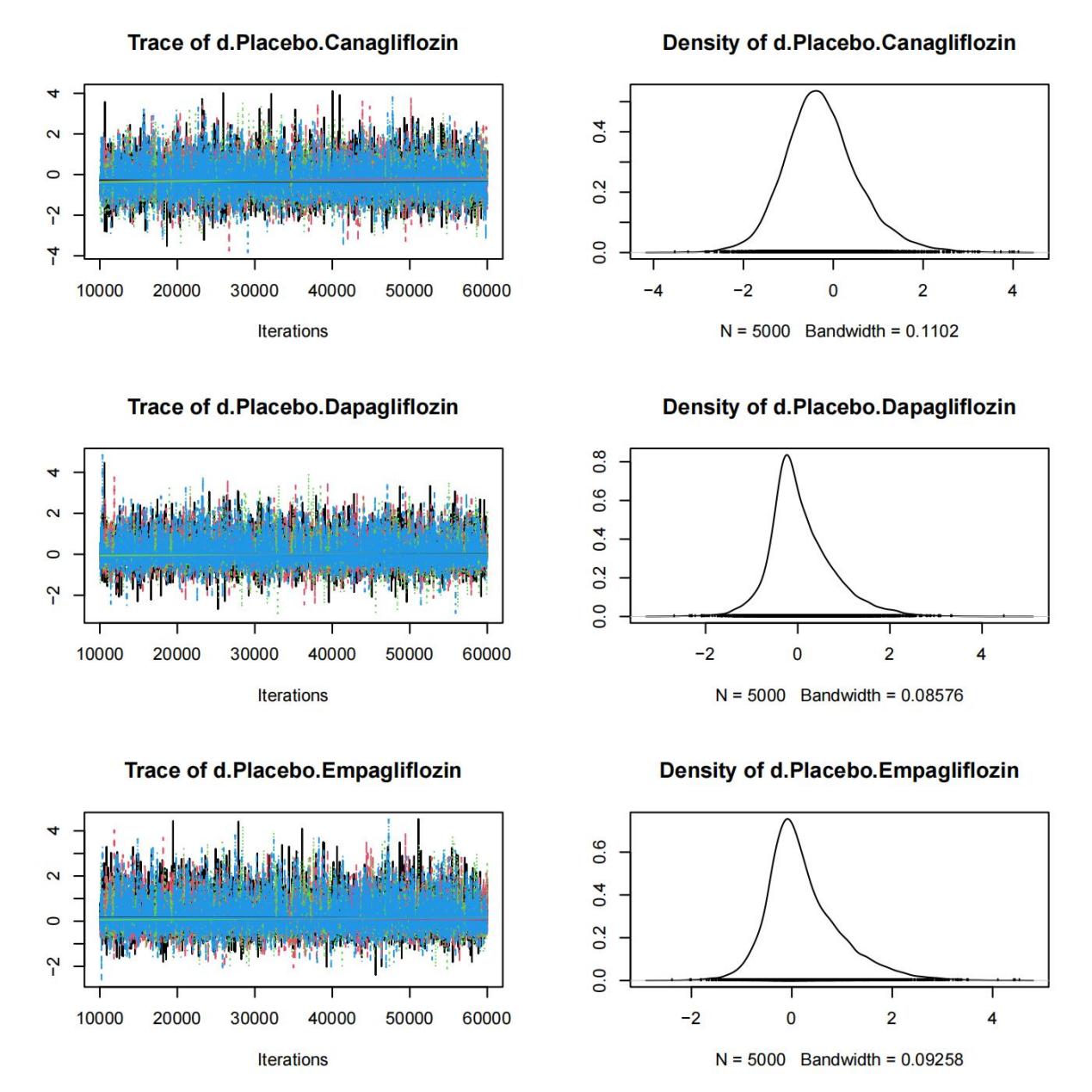


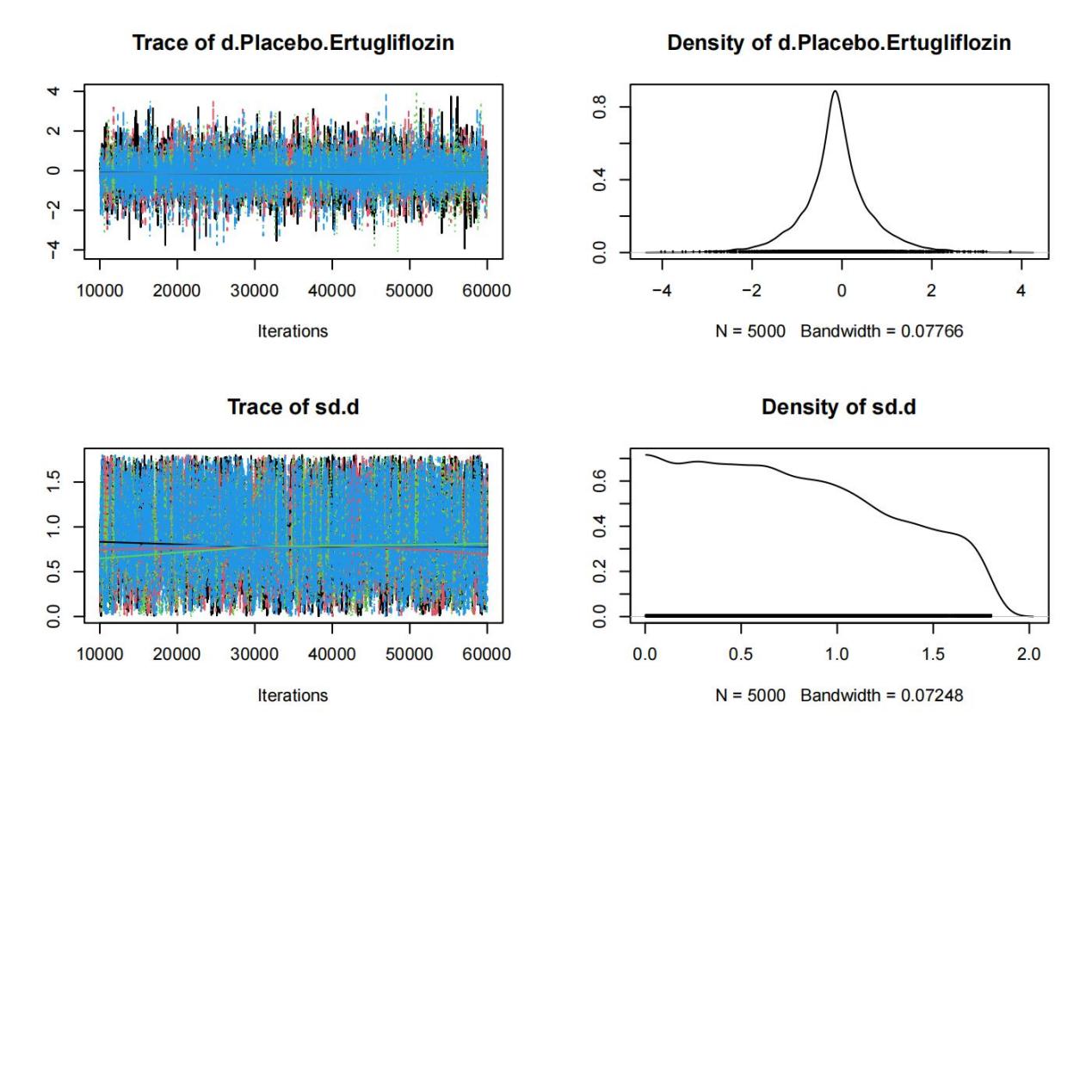
6.10 The Brooks-Gelman-Rubin diagnosis plot of severe hypoglycemia


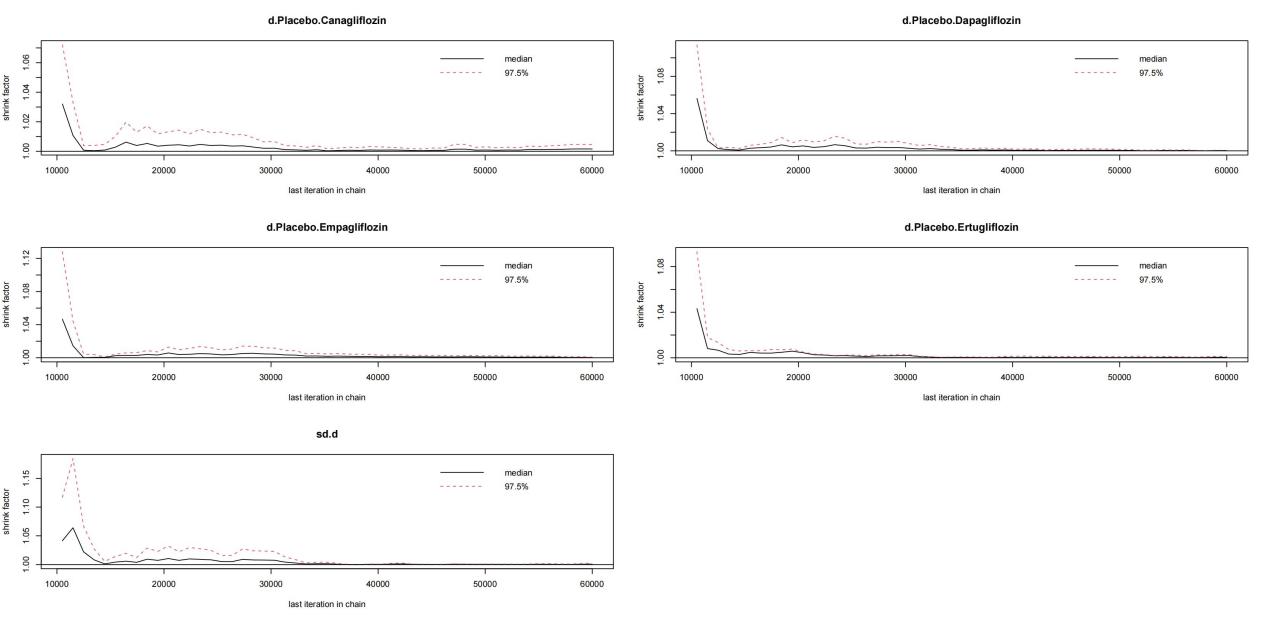
Appendix 7: Network plot

## 7.1 Reproductive tract infections in male

**
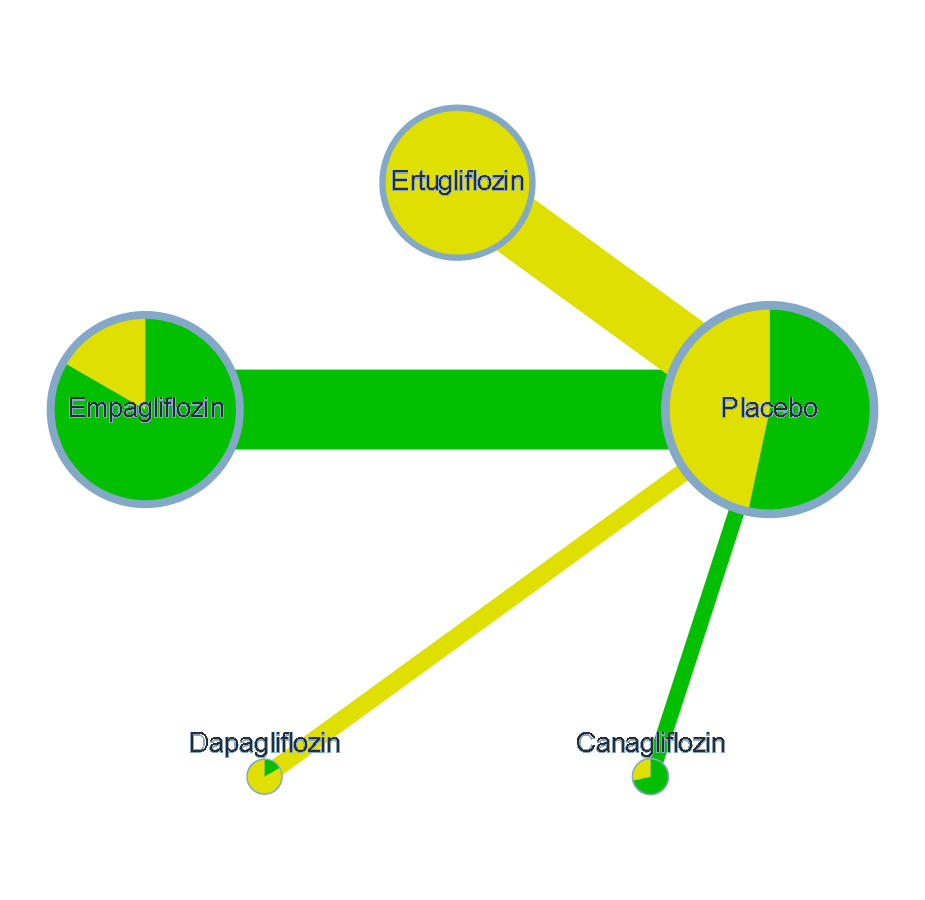
**

## 7.2 Reproductive tract infections in female

**
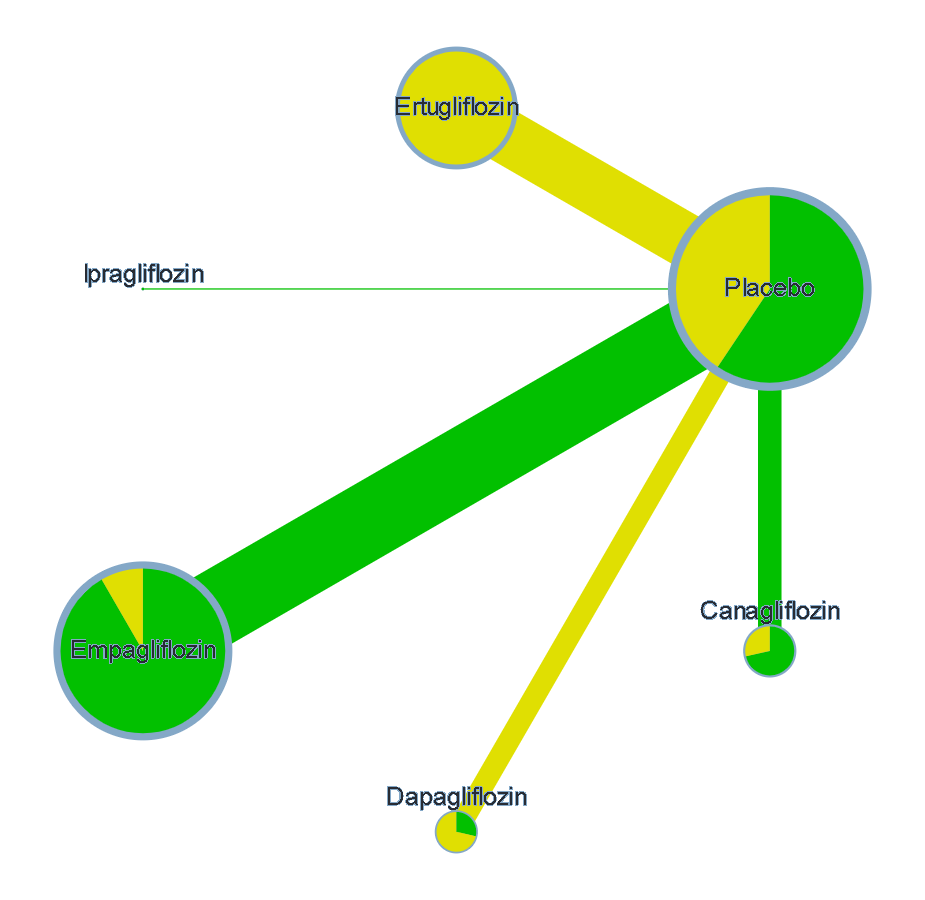
**

## 7.3 Hypovolemia

**
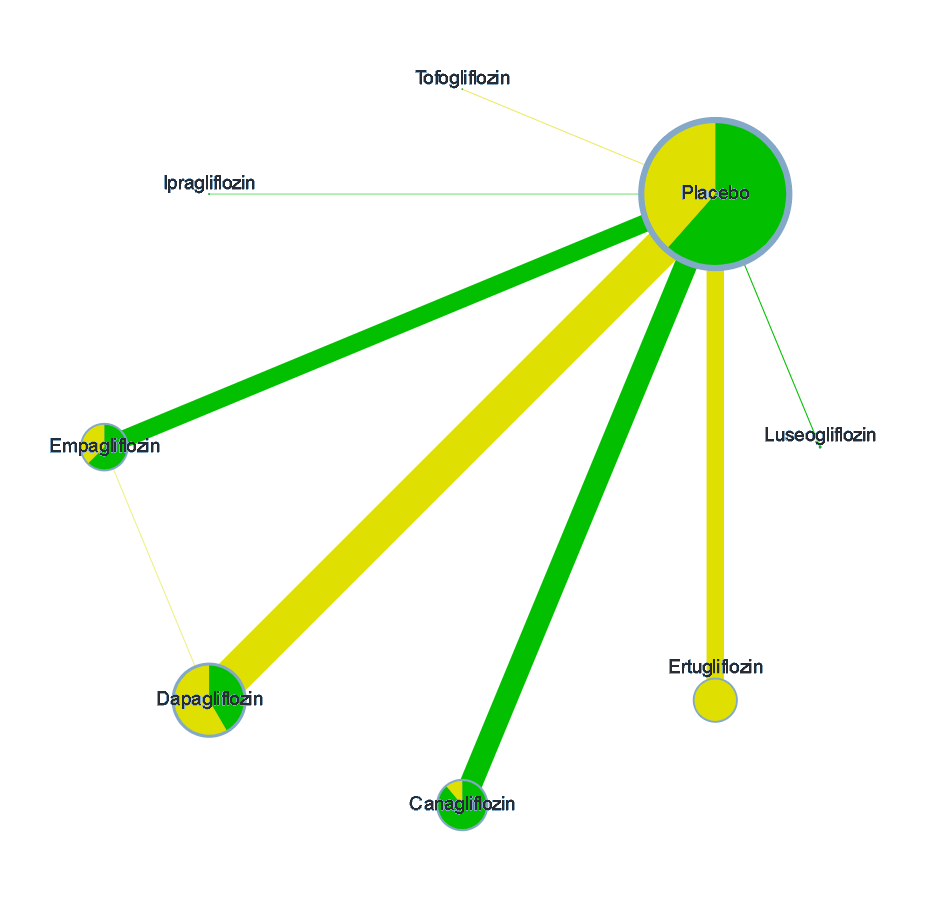
**

## 7.4 Renal impairment or failure

**
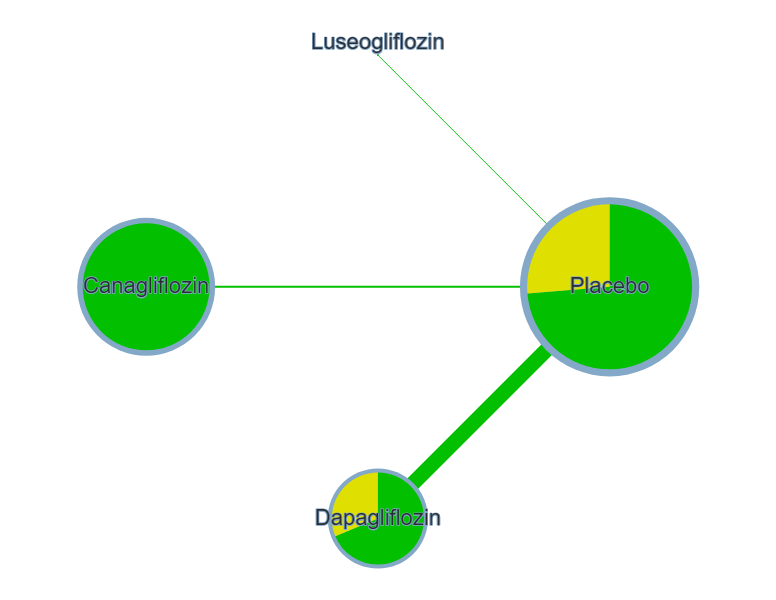
**

## 7.5 Acute kidney injury

**
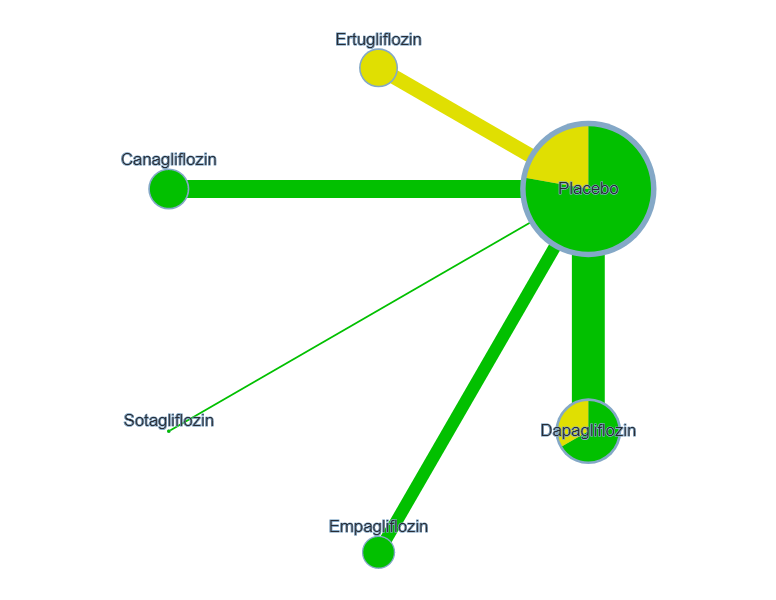
**

## 7.6 Urinary tract infections

**
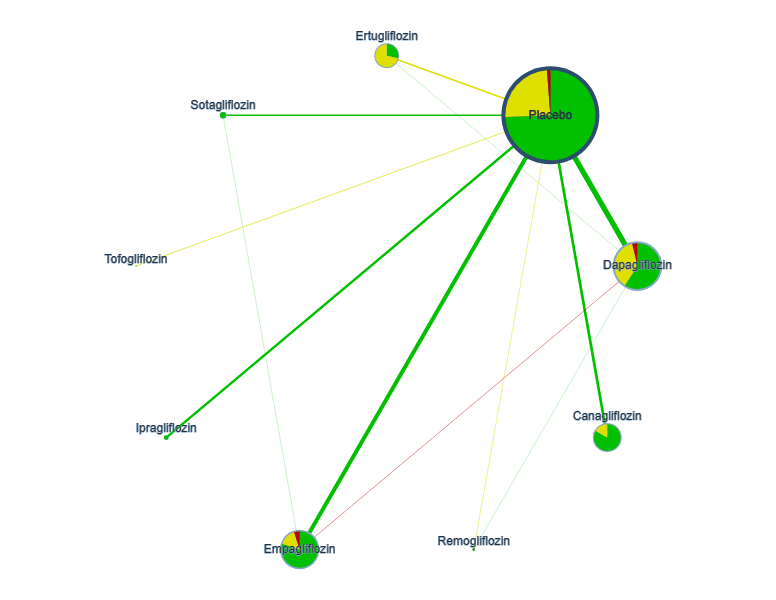
**

## 7.7 [Diabetic ketoacidosis](javascript:;)

**
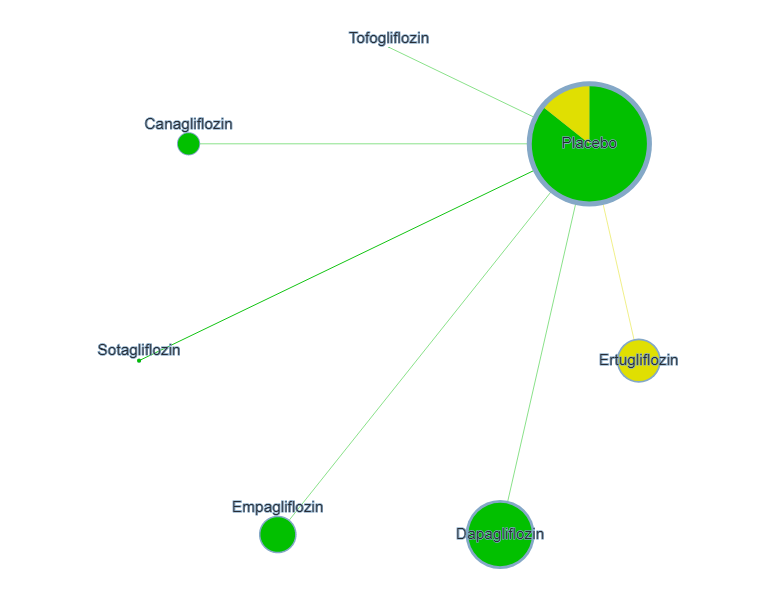
**

## 7.8 Amputation

**
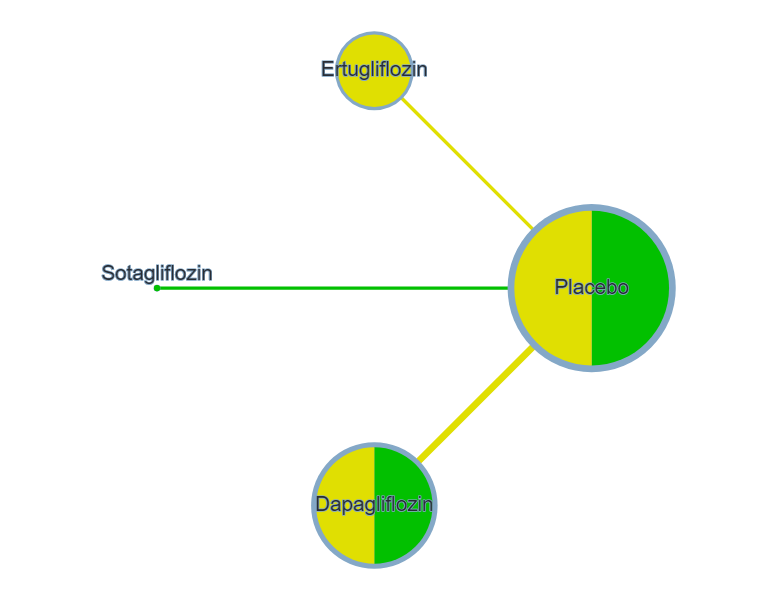
**

## 7.9 Fracture

**
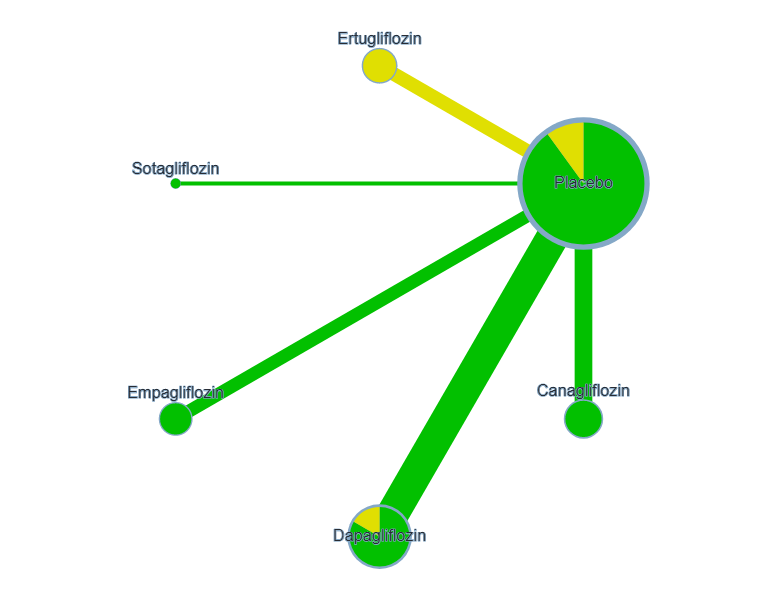
**

## 7.10 Severe hypoglycemia

**
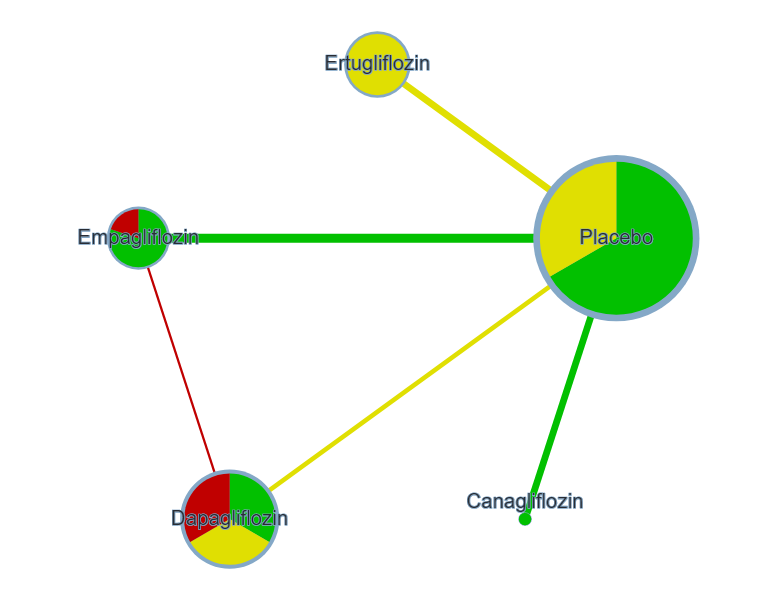
**

Appendix 8: Forest plot for network meta-analysis

## 8.1 Reproductive tract infections in male


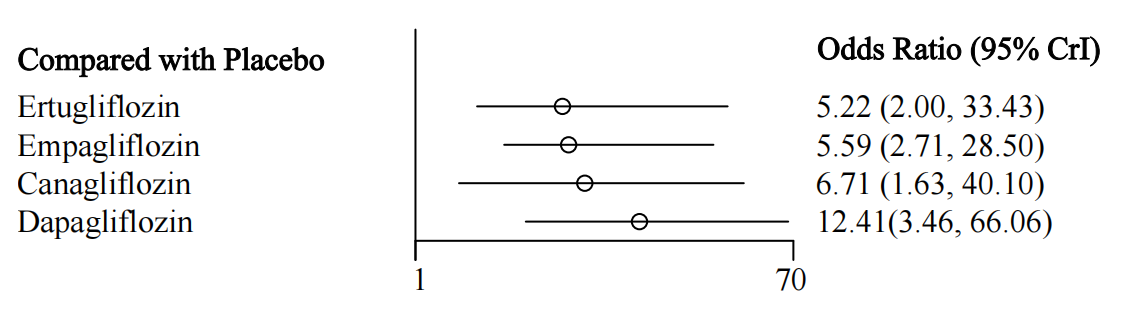


## 8.2 Reproductive tract infections in female


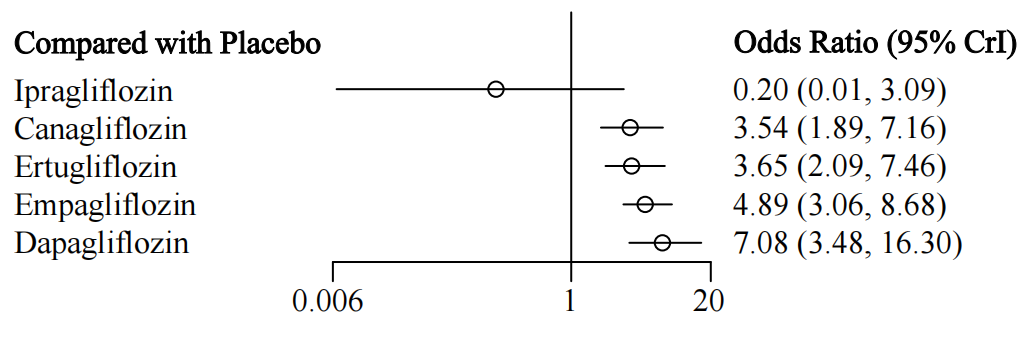


## 8.3 Hypovolemia


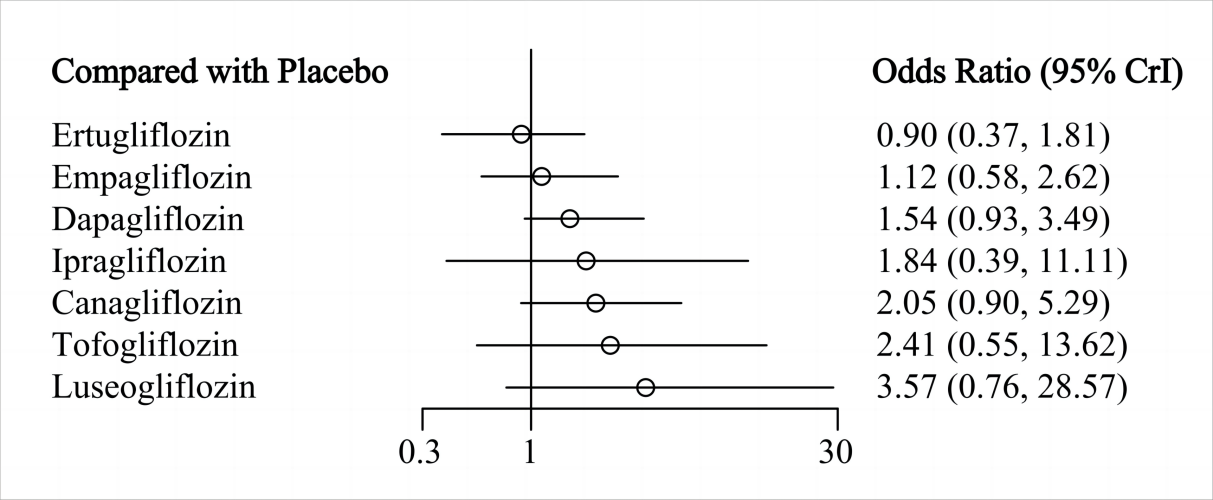


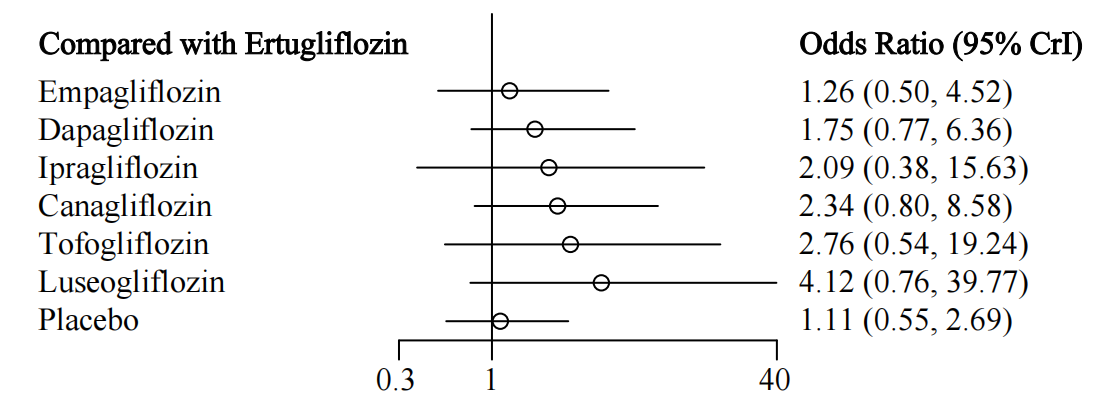


## 8.4 Renal impairment or failure


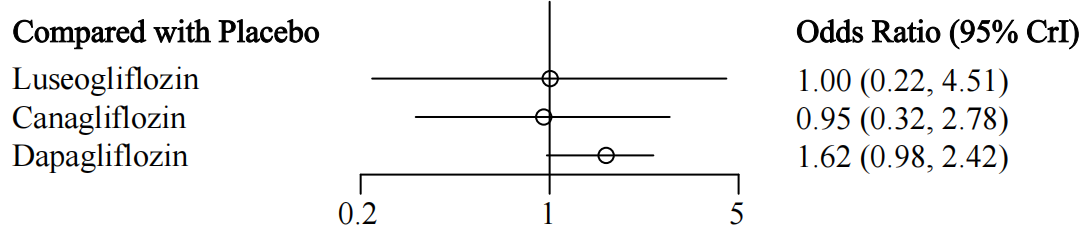


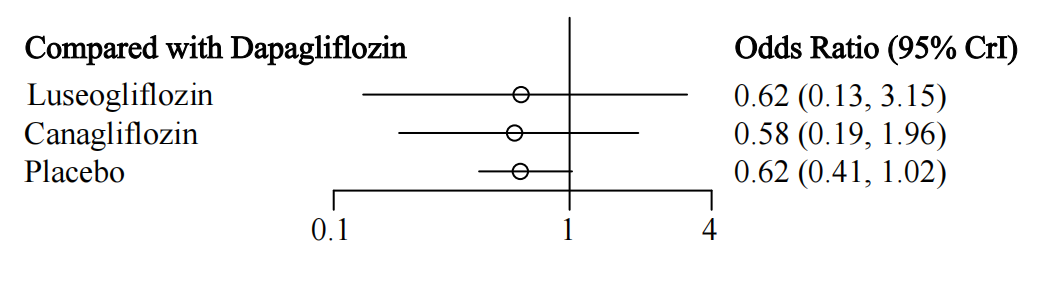


## 8.5 Acute kidney injury


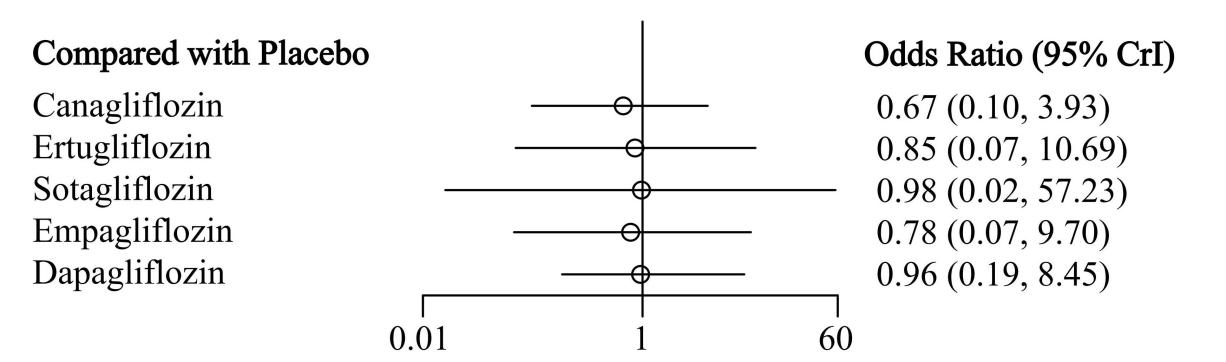


## 8.6 Urinary tract infections


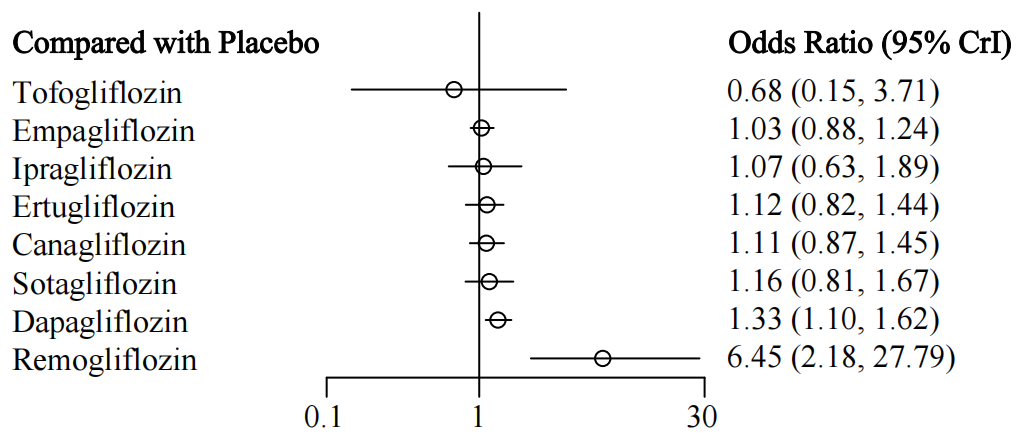


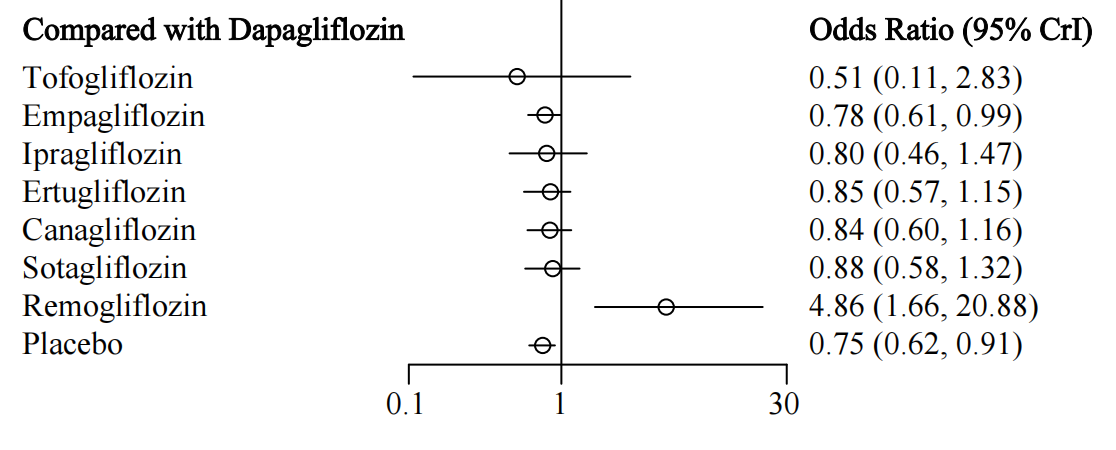


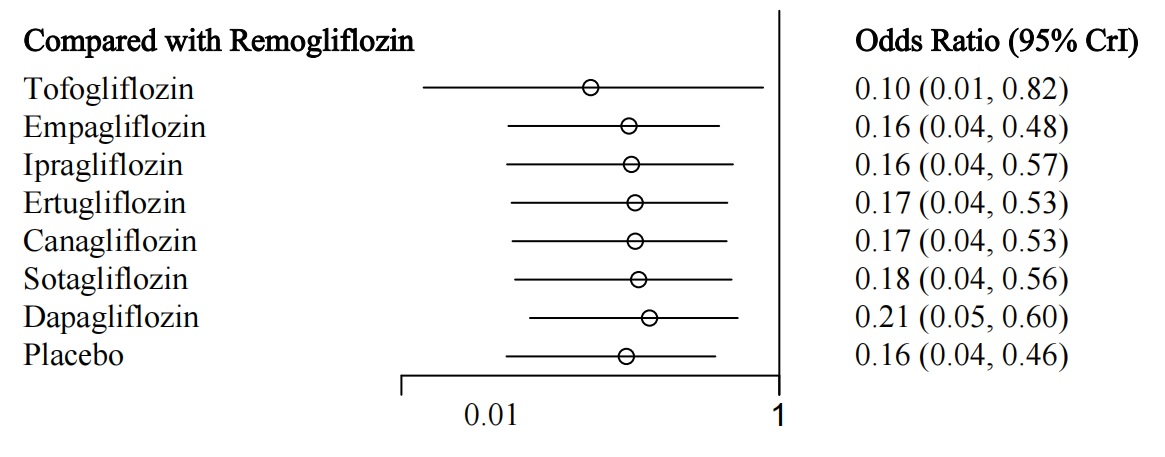

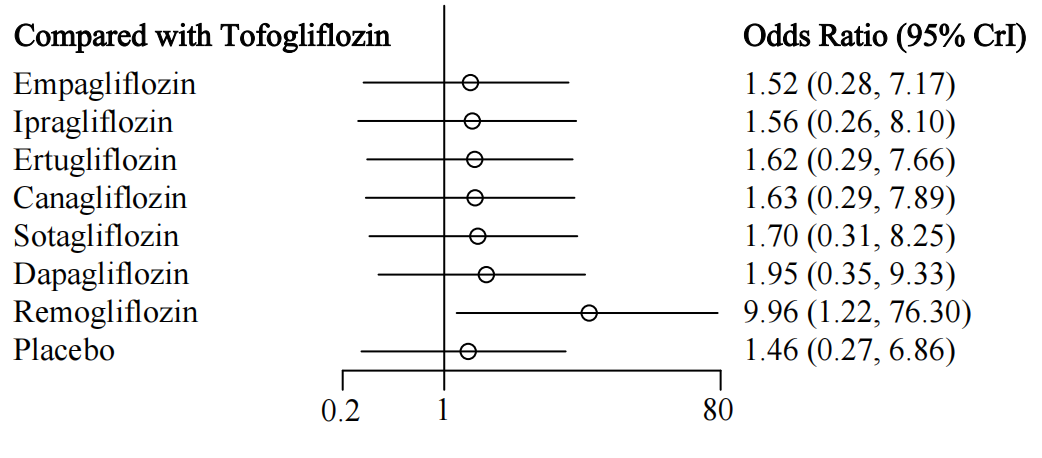


## 8.7 [Diabetic ketoacidosis](javascript:;)


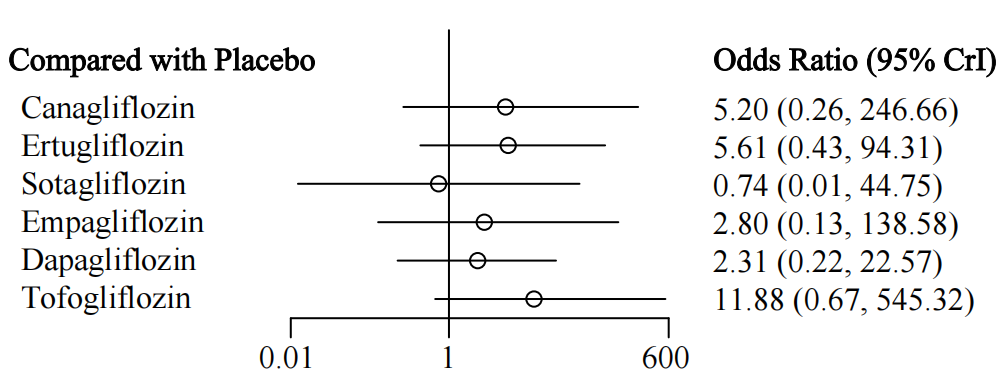


## 8.8 Amputation


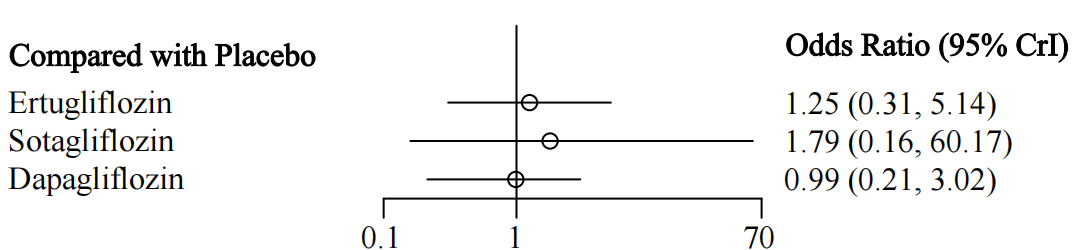


## 8.9 Fracture


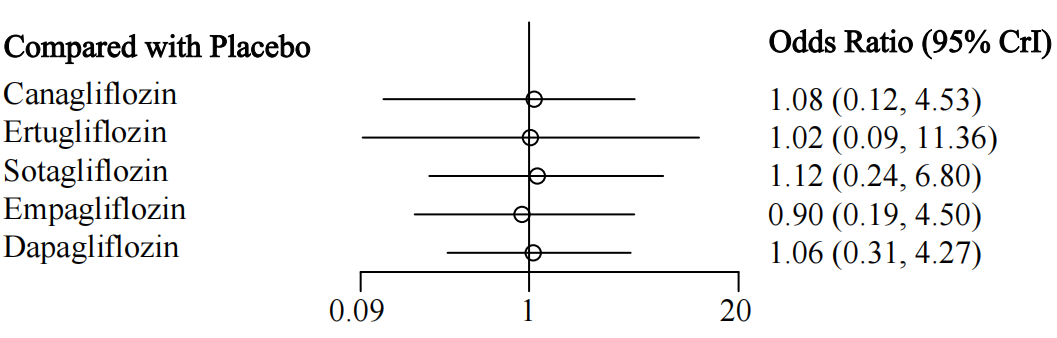


## 8.10 Severe hypoglycemia


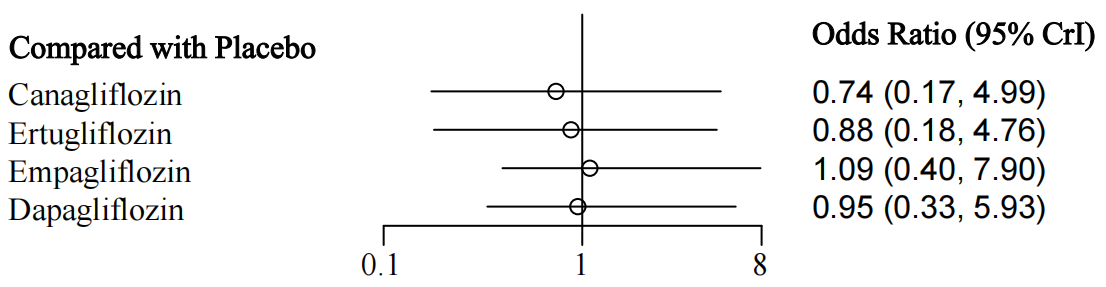


# Appendix 9: league table for network meta-analysis

## 9.1 Network meta-analysis results for reproductive tract infections in male

| **Ertugliflozin** | 1.03 (0.22, 6.25) | 1.23 (0.12, 8.56) | 2.24 (0.27, 14.65) | ***0.19 (0.03, 0.50)*** |
| --- | --- | --- | --- | --- |
| 0.97 (0.16, 4.61) | **Empagliflozin** | 1.15 (0.13, 7.00) | 2.12 (0.28, 11.37) | ***0.18 (0.04, 0.37)*** |
| 0.81 (0.12, 8.14) | 0.87 (0.14, 7.57) | **Canagliflozin** | 1.86 (0.21, 17.16) | ***0.15 (0.02, 0.61)*** |
| 0.45 (0.07, 3.71) | 0.47 (0.09, 3.56) | 0.54 (0.06, 4.71) | **Dapagliflozin** | ***0.08 (0.02, 0.29)*** |
| ***5.22 (2.00, 33.43)*** | ***5.59 (2.71, 28.5)*** | ***6.71 (1.63, 40.1)*** | ***12.41 (3.46, 66.06)*** | **Placebo** |

Treatment estimates are ORs and 95% CIs. Signifificant results are italicized and highlighted in bold；OR = odds ratio.

## 9.2 Network meta-analysis results for reproductive tract infections in female

| **Ipragliflozin** | ***17.7 (1.08, 595.81)*** | ***18.59 (1.18, 642.01)*** | ***24.64 (1.60, 830.75)*** | ***36.19 (2.21, 1236.11)*** | 5.03 (0.32, 153.93) |
| --- | --- | --- | --- | --- | --- |
| 0.06 (0.00, 0.93) | **Canagliflozin** | 1.04 (0.42, 2.66) | 1.39 (0.60, 3.22) | 2.00 (0.75, 5.69) | ***0.28 (0.14, 0.53)*** |
| 0.05 (0.00, 0.85) | 0.96 (0.38, 2.36) | **Ertugliflozin** | 1.34 (0.59, 2.93) | 1.93 (0.72, 5.20) | ***0.27 (0.13, 0.48)*** |
| 0.04 (0.00, 0.62) | 0.72 (0.31, 1.66) | 0.75 (0.34, 1.69) | **Empagliflozin** | 1.44 (0.60, 3.65) | ***0.20 (0.12, 0.33)*** |
| 0.03 (0.00, 0.45) | 0.50 (0.18, 1.33) | 0.52 (0.19, 1.39) | 0.70 (0.27, 1.66) | **Dapagliflozin** | ***0.14 (0.06, 0.29)*** |
| 0.20 (0.01, 3.09) | ***3.54 (1.89, 7.16)*** | ***3.65 (2.09, 7.46)*** | ***4.89 (3.06, 8.68)*** | ***7.08 (3.48, 16.30)*** | **Placebo** |

Treatment estimates are ORs and 95% CIs. Signifificant results are italicized and highlighted in bold；OR = odds ratio.

## 9.3 Network meta-analysis results for hypovolemia

| **Ertugliflozin** | 1.26 (0.50, 4.52) | 1.74 (0.77, 6.36) | 2.09 (0.38, 15.63) | 2.34 (0.80, 8.58) | 2.76 (0.54, 19.24) | 4.12 (0.76, 39.77) | 1.11 (0.55, 2.69) |
| --- | --- | --- | --- | --- | --- | --- | --- |
| 0.80 (0.22, 2.01) | **Empagliflozin** | 1.34 (0.61, 3.71) | 1.61 (0.28, 11.00) | 1.80 (0.58, 5.60) | 2.13 (0.37, 13.10) | 3.15 (0.55, 26.29) | 0.89 (0.38, 1.73) |
| 0.57 (0.16, 1.31) | 0.75 (0.27, 1.65) | **Dapagliflozin** | 1.16 (0.20, 7.47) | 1.31 (0.42, 3.70) | 1.54 (0.27, 8.95) | 2.26 (0.40, 18.27) | 0.65 (0.29, 1.07) |
| 0.48 (0.06, 2.64) | 0.62 (0.09, 3.60) | 0.86 (0.13, 5.10) | **Ipragliflozin** | 1.12 (0.16, 7.01) | 1.32 (0.13, 14.14) | 2.00 (0.19, 25.40) | 0.54 (0.09, 2.55) |
| 0.43 (0.12, 1.25) | 0.56 (0.18, 1.74) | 0.76 (0.27, 2.39) | 0.89 (0.14, 6.41) | **Canagliflozin** | 1.18 (0.20, 7.92) | 1.75 (0.28, 16.05) | 0.49 (0.19, 1.12) |
| 0.36 (0.05, 1.84) | 0.47 (0.08, 2.68) | 0.65 (0.11, 3.74) | 0.76 (0.07, 7.81) | 0.85 (0.13, 5.09) | **Tofogliflozin** | 1.52 (0.15, 18.91) | 0.42 (0.07, 1.82) |
| 0.24 (0.03, 1.32) | 0.32 (0.04, 1.80) | 0.44 (0.05, 2.50) | 0.50 (0.04, 5.33) | 0.57 (0.06, 3.51) | 0.66 (0.05, 6.88) | **Luseogliflozin** | 0.28 (0.03, 1.32) |
| 0.90 (0.37, 1.81) | 1.12 (0.58, 2.62) | 1.54 (0.93, 3.49) | 1.84 (0.39, 11.11) | 2.05 (0.9, 5.29) | 2.41 (0.55, 13.62) | 3.57 (0.76, 28.57) | **Placebo** |

Treatment estimates are ORs and 95% CIs. Signifificant results are italicized and highlighted in bold；OR = odds ratio.

## 9.4 Network meta-analysis results for renal impairment or failure

| **Luseogliflozin** | 0.94 (0.15, 6.11) | 1.61 (0.32, 7.50) | 1.00 (0.22, 4.53) |
| --- | --- | --- | --- |
| 1.06 (0.16, 6.89) | **Canagliflozin** | 1.71 (0.51, 5.28) | 1.05 (0.36, 3.13) |
| 0.62 (0.13, 3.15) | 0.58 (0.19, 1.96) | **Dapagliflozin** | 0.62 (0.41, 1.02) |
| 1.00 (0.22, 4.51) | 0.95 (0.32, 2.78) | 1.62 (0.98, 2.42) | **Placebo** |

Treatment estimates are ORs and 95% CIs. Signifificant results are italicized and highlighted in bold；OR = odds ratio.

## 9.5 Network meta-analysis results for acute kidney injury

| **Canagliflozin** | 1.26 (0.06, 31.02) | 1.48 (0.02, 125.46) | 1.17 (0.06, 27.76) | 1.46 (0.14, 28.15) | 1.5 (0.25, 10.19) |
| --- | --- | --- | --- | --- | --- |
| 0.79 (0.03, 16.18) | **Ertugliflozin** | 1.14 (0.01, 125.77) | 0.92 (0.02, 31.04) | 1.09 (0.06, 35.12) | 1.17 (0.09, 14.31) |
| 0.67 (0.01, 56.05) | 0.88 (0.01, 102.12) | **Sotagliflozin** | 0.81 (0.01, 91.57) | 1.07 (0.01, 107.85) | 1.02 (0.02, 62.52) |
| 0.85 (0.04, 17.64) | 1.09 (0.03, 41.24) | 1.24 (0.01, 141.66) | **Empagliflozin** | 1.20 (0.07, 36.92) | 1.28 (0.10, 14.80) |
| 0.68 (0.04, 7.04) | 0.91 (0.03, 16.85) | 0.93 (0.01, 76.95) | 0.84 (0.03, 15.12) | **Dapagliflozin** | 1.04 (0.12, 5.40) |
| 0.67 (0.10, 3.93) | 0.85 (0.07, 10.69) | 0.98 (0.02, 57.23) | 0.78 (0.07, 9.70) | 0.96 (0.19, 8.45) | **Placebo** |

Treatment estimates are ORs and 95% CIs. Signifificant results are italicized and highlighted in bold；OR = odds ratio.

## 9.6 Network meta-analysis results for [urinary tract infection](javascript:;)s

| **Tofogliflozin** | 1.52 (0.28, 7.17) | 1.56 (0.26, 8.10) | 1.62 (0.29, 7.66) | 1.63 (0.29, 7.88) | 1.70 (0.31, 8.25) | 1.95 (0.35, 9.33) | ***9.96 (1.22, 76.30)*** | 1.46 (0.27, 6.86) |
| --- | --- | --- | --- | --- | --- | --- | --- | --- |
| 0.66 (0.14, 3.59) | **Empagliflozin** | 1.03 (0.59, 1.88) | 1.09 (0.75, 1.46) | 1.08 (0.78, 1.48) | 1.13 (0.76, 1.66) | ***1.28 (1.01, 1.65)*** | ***6.25 (2.08, 27.15)*** | 0.97 (0.80, 1.14) |
| 0.64 (0.12, 3.91) | 0.97 (0.53, 1.68) | **Ipragliflozin** | 1.04 (0.55, 1.85) | 1.05 (0.56, 1.84) | 1.09 (0.55, 2.05) | 1.25 (0.68, 2.19) | ***6.06 (1.76, 27.60)*** | 0.94 (0.53, 1.58) |
| 0.62 (0.13, 3.39) | 0.92 (0.68, 1.34) | 0.96 (0.54, 1.82) | **Ertugliflozin** | 1.00 (0.70, 1.48) | 1.04 (0.67, 1.70) | 1.18 (0.87, 1.76) | ***5.80 (1.88, 26.11)*** | 0.89 (0.69, 1.22) |
| 0.61 (0.13, 3.47) | 0.93 (0.68, 1.27) | 0.96 (0.54, 1.80) | 1.00 (0.68, 1.42) | **Canagliflozin** | 1.04 (0.68, 1.64) | 1.19 (0.86, 1.67) | ***5.79 (1.90, 25.82)*** | 0.90 (0.69, 1.15) |
| 0.59 (0.12, 3.27) | 0.89 (0.60, 1.32) | 0.92 (0.49, 1.81) | 0.96 (0.59, 1.49) | 0.96 (0.61, 1.48) | **Sotagliflozin** | 1.14 (0.76, 1.73) | ***5.55 (1.79, 25.03)*** | 0.86 (0.60, 1.23) |
| 0.51 (0.11, 2.83) | 0.78 (0.61, 0.99) | 0.80 (0.46, 1.47) | 0.85 (0.57, 1.15) | 0.84 (0.60, 1.16) | 0.88 (0.58, 1.32) | **Dapagliflozin** | ***4.86 (1.66, 20.88)*** | ***0.75 (0.62, 0.91)*** |
| ***0.10 (0.01, 0.82)*** | ***0.16 (0.04, 0.48)*** | ***0.16 (0.04, 0.57)*** | ***0.17 (0.04, 0.53)*** | ***0.17 (0.04, 0.53)*** | ***0.18 (0.04, 0.56)*** | ***0.21 (0.05, 0.60)*** | **Remogliflozin** | ***0.16 (0.04, 0.46)*** |
| 0.68 (0.15, 3.71) | 1.03 (0.88, 1.24) | 1.07 (0.63, 1.89) | 1.12 (0.82, 1.44) | 1.11 (0.87, 1.45) | 1.16 (0.81, 1.67) | ***1.33 (1.1, 1.62)*** | ***6.45 (2.18, 27.79)*** | **Placebo** |

Treatment estimates are ORs and 95% CIs. Signifificant results are italicized and highlighted in bold；OR = odds ratio.

## 9.7 Network meta-analysis results for amputation

| **Ertugliflozin** | 1.44 (0.09, 62.78) | 0.79 (0.09, 4.56) | 0.80 (0.19, 3.27) |
| --- | --- | --- | --- |
| 0.69 (0.02, 11.20) | **Sotagliflozin** | 0.52 (0.01, 7.48) | 0.56 (0.02, 6.30) |
| 1.26 (0.22, 10.98) | 1.93 (0.13, 81.85) | **Dapagliflozin** | 1.01 (0.33, 4.69) |
| 1.25 (0.31, 5.14) | 1.79 (0.16, 60.17) | 0.99 (0.21, 3.02) | **Placebo** |

9.8 Network meta-analysis results for diabetic ketoacidosis

| **Canagliflozin** | 1.06 (0.01, 71.97) | 0.14 (0.00, 20.16) | 0.54 (0.00, 65.89) | 0.44 (0.00, 17.73) | 2.34 (0.02, 282.22) | 0.19 (0.00, 3.77) |
| --- | --- | --- | --- | --- | --- | --- |
| 0.94 (0.01, 86.13) | **Ertugliflozin** | 0.13 (0.00, 16.47) | 0.50 (0.01, 50.59) | 0.41 (0.01, 12.43) | 2.18 (0.04, 205.10) | 0.18 (0.01, 2.30) |
| 7.3 (0.05, 1546.24) | 7.85 (0.06, 1070.90) | **Sotagliflozin** | 4.11 (0.02, 921.59) | 3.13 (0.03, 303.20) | 17.34 (0.11, 4282.47) | 1.35 (0.02, 81.23) |
| 1.84 (0.02, 253.39) | 1.99 (0.02, 132.57) | 0.24 (0.00, 44.05) | **Empagliflozin** | 0.81 (0.01, 37.09) | 4.3 (0.04, 566.60) | 0.36 (0.01, 7.87) |
| 2.29 (0.06, 205.00) | 2.46 (0.08, 94.33) | 0.32 (0.00, 36.09) | 1.24 (0.03, 109.59) | **Dapagliflozin** | 5.44 (0.13, 437.32) | 0.43 (0.04, 4.46) |
| 0.43 (0.00, 50.20) | 0.46 (0.00, 25.85) | 0.06 (0.00, 8.90) | 0.23 (0.00, 28.34) | 0.18 (0.00, 7.52) | **Tofogliflozin** | 0.08 (0.00, 1.49) |
| 5.2 (0.26, 246.66) | 5.61 (0.43, 94.31) | 0.74 (0.01, 44.75) | 2.80 (0.13, 138.58) | 2.31 (0.22, 22.57) | 11.88 (0.67, 545.32) | **Placebo** |

Treatment estimates are ORs and 95% CIs. Signifificant results are italicized and highlighted in bold；OR = odds ratio.

## 9.9 Network meta-analysis results for fracture

| **Canagliflozin** | 0.88 (0.07, 29.16) | 1.06 (0.13, 19.37) | 0.80 (0.12, 13.18) | 0.99 (0.17, 14.24) | 0.93 (0.22, 8.02) |
| --- | --- | --- | --- | --- | --- |
| 1.14 (0.03, 13.93) | **Ertugliflozin** | 1.06 (0.07, 23.75) | 0.89 (0.05, 16.10) | 1.04 (0.07, 17.51) | 0.98 (0.09, 10.80) |
| 0.94 (0.05, 7.65) | 0.94 (0.04, 13.76) | **Sotagliflozin** | 0.80 (0.07, 6.76) | 0.97 (0.11, 6.99) | 0.89 (0.15, 4.16) |
| 1.25 (0.08, 8.37) | 1.13 (0.06, 20.35) | 1.24 (0.15, 13.48) | **Empagliflozin** | 1.18 (0.16, 9.65) | 1.11 (0.22, 5.13) |
| 1.01 (0.07, 6.04) | 0.96 (0.06, 13.85) | 1.03 (0.14, 8.88) | 0.85 (0.10, 6.24) | **Dapagliflozin** | 0.94 (0.23, 3.21) |
| 1.08 (0.12, 4.53) | 1.02 (0.09, 11.36) | 1.12 (0.24, 6.80) | 0.90 (0.19, 4.50) | 1.06 (0.31, 4.27) | **Placebo** |

## 9.10 Network meta-analysis results for severe hypoglycemia

| **Canagliflozin** | 1.24 (0.09, 9.42) | 1.58 (0.20, 15.36) | 1.37 (0.16, 11.88) | 1.36 (0.20, 5.75) |
| --- | --- | --- | --- | --- |
| 0.81 (0.11, 10.93) | **Ertugliflozin** | 1.21 (0.21, 17.37) | 1.05 (0.18, 13.28) | 1.14 (0.21, 5.58) |
| 0.63 (0.07, 4.90) | 0.83 (0.06, 4.76) | **Empagliflozin** | 0.88 (0.14, 4.32) | 0.92 (0.13, 2.52) |
| 0.73 (0.08, 6.20) | 0.95 (0.08, 5.43) | 1.14 (0.23, 7.00) | **Dapagliflozin** | 1.05 (0.17, 3.01) |
| 0.74 (0.17, 4.99) | 0.88 (0.18, 4.76) | 1.09 (0.40, 7.90) | 0.95 (0.33, 5.93) | **Placebo** |

Treatment estimates are ORs and 95% CIs. Signifificant results are italicized and highlighted in bold；OR = odds ratio

# Appendix 10: Inconsistency Analysis

## 10.1 Node-splitting: [reproductive tract infections](javascript:;)


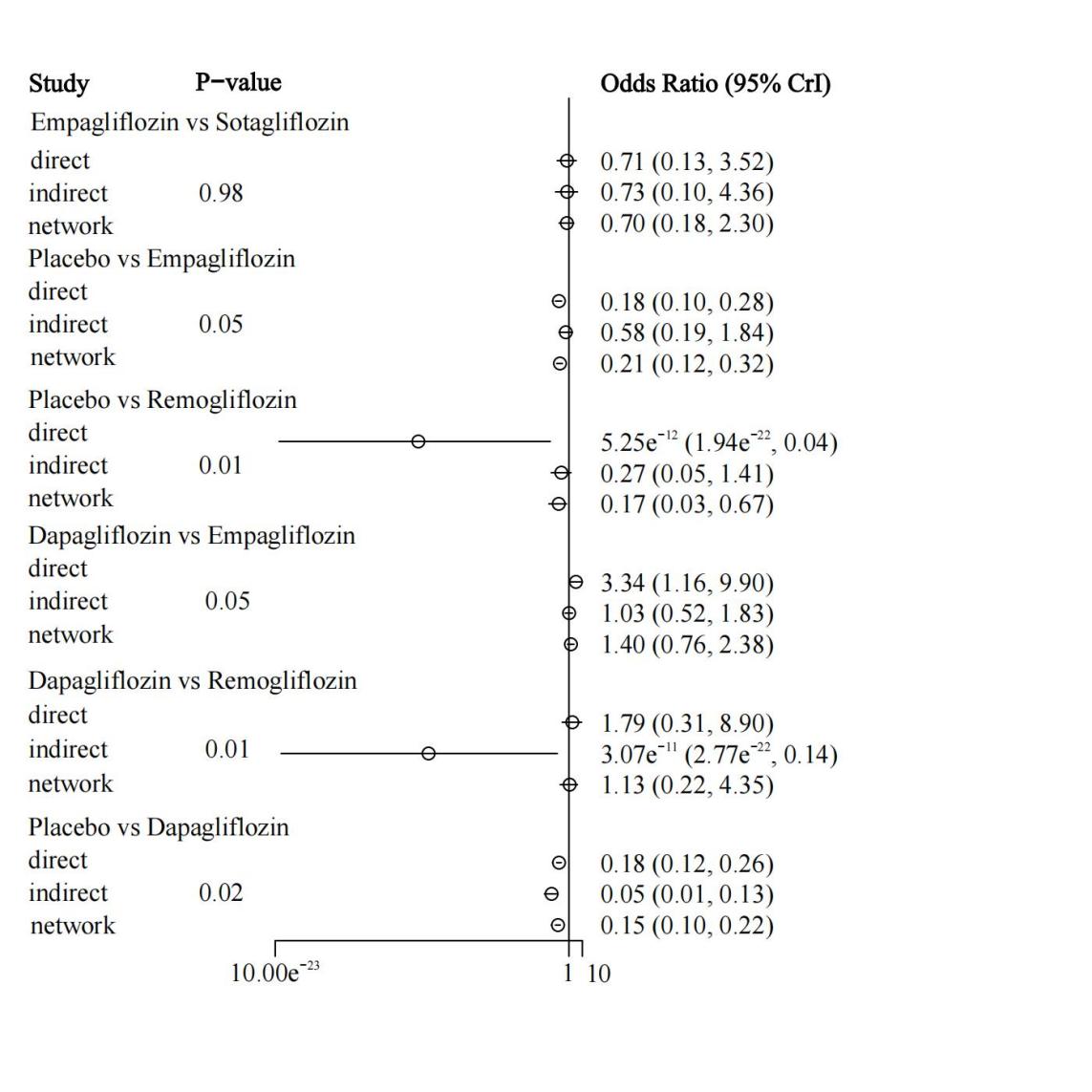


10.2 Node-splitting: pollakiuria


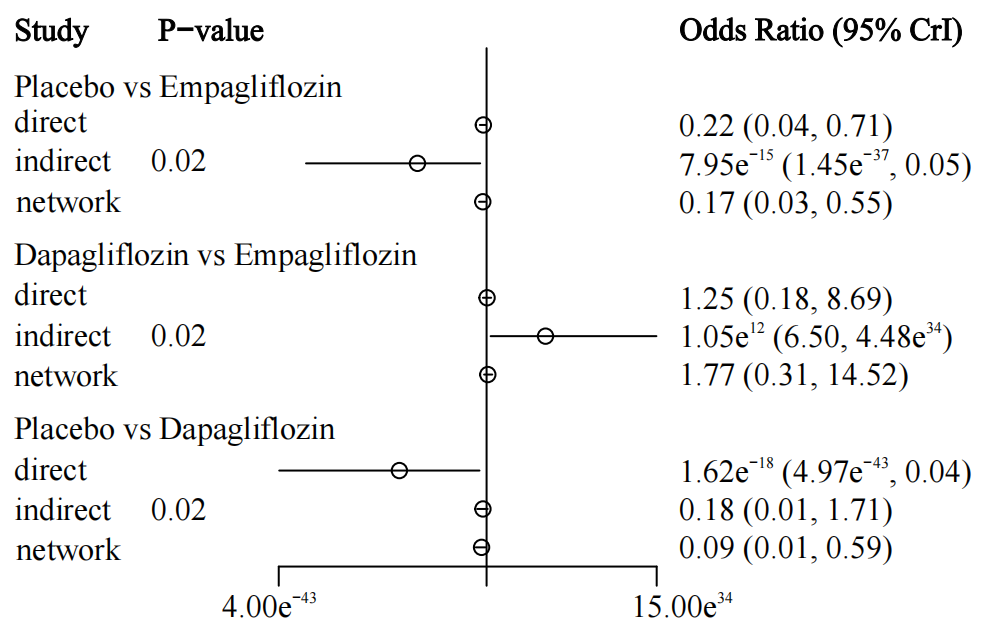


10.3 Node-splitting: hypovolemia


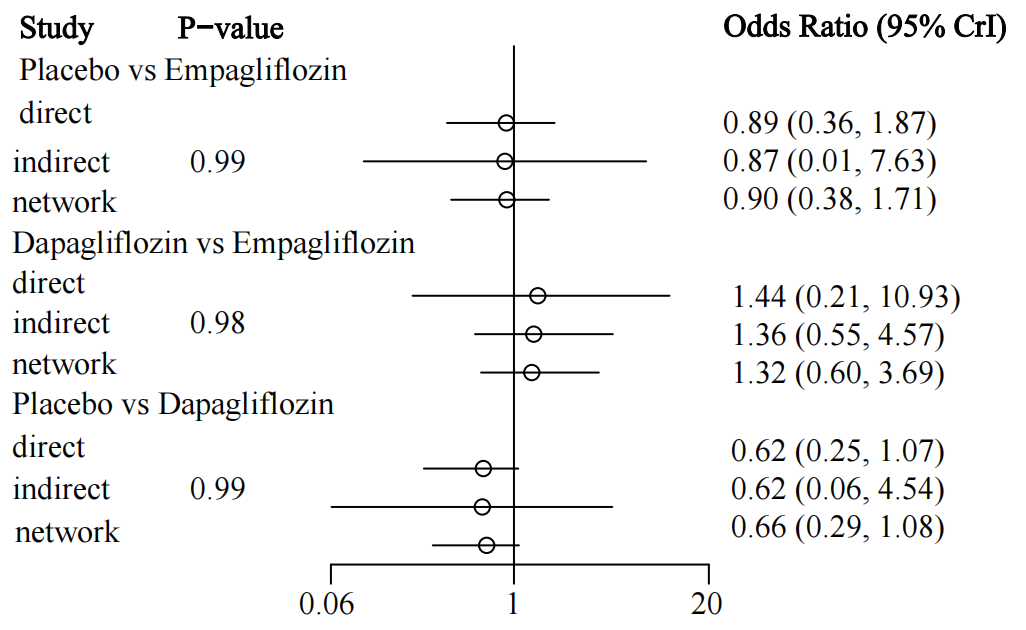


10.4 Node-splitting: [urinary tract infection](javascript:;)s


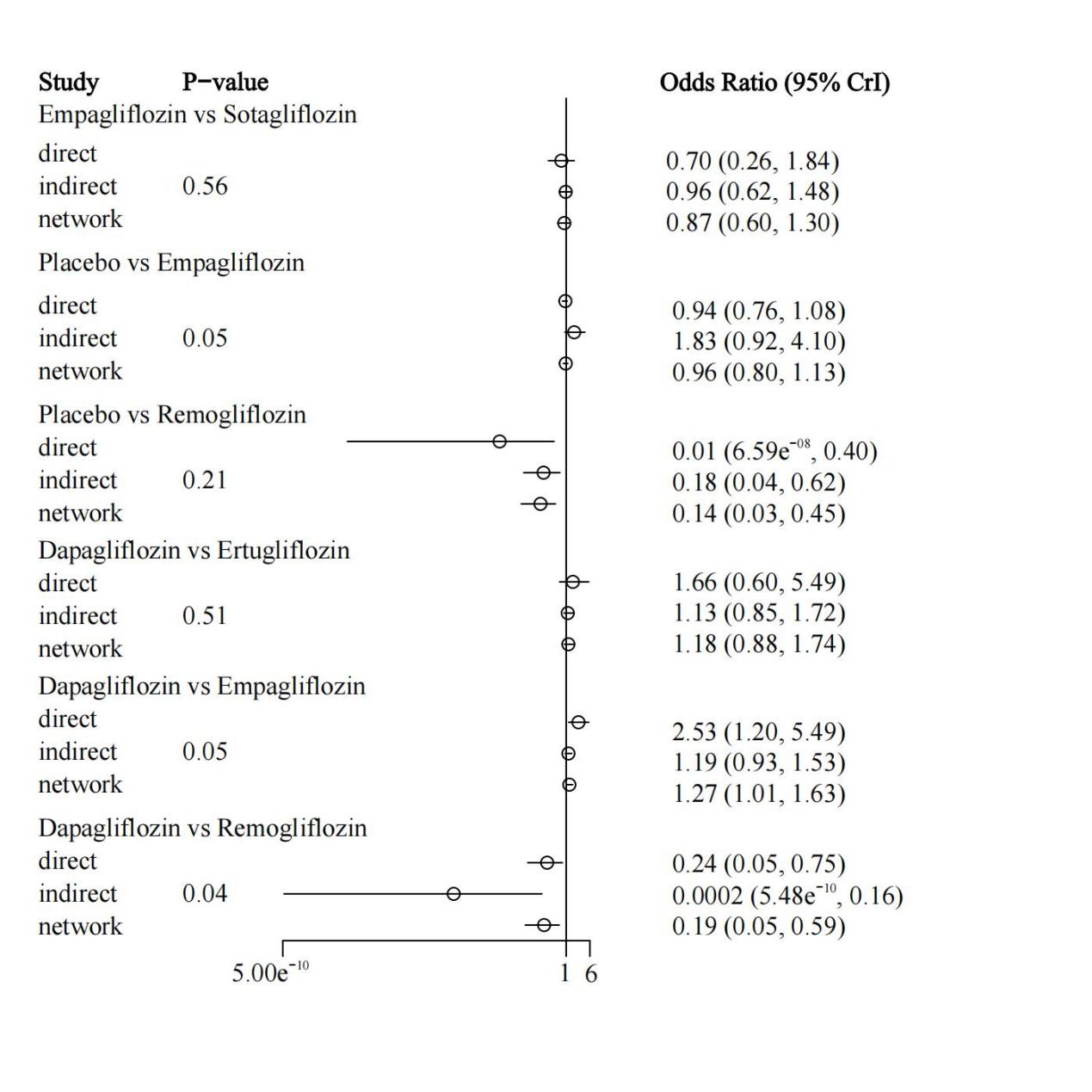


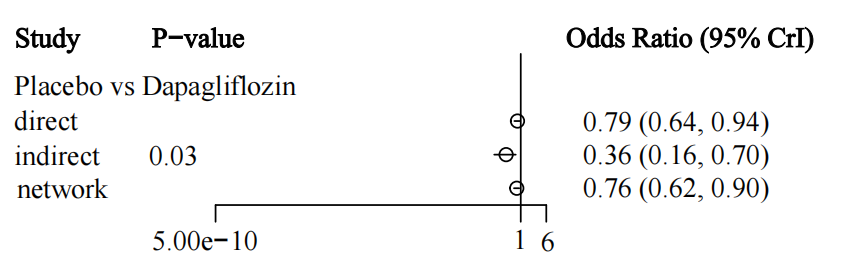


10.5 Node-splitting: severe hypoglycemia

#
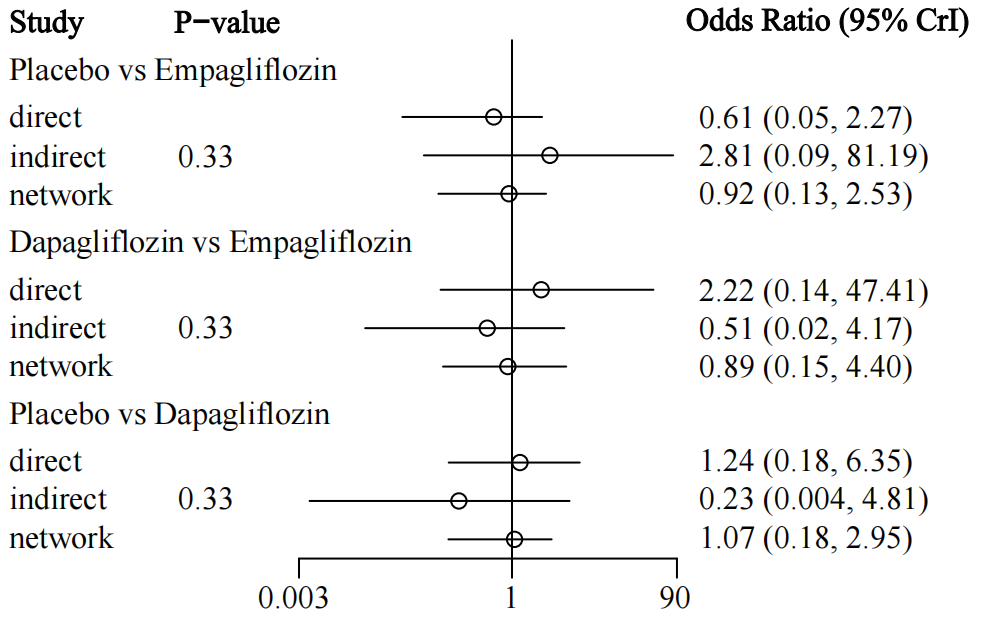
**Appendix 11: Heterogeneity Assessment**

## 11.1 Reproductive tract infections Model

Network I^2^ (from NMA in all trials, 153 data points, ratio 1.08)=8%

DIC = 266.71 (Dbar = 164.49; pD =102.22)

## 11.1 Heterogeneity score by each comparison for main model of reproductive tract infections

| **Treatment1** | **Treatment2** | ***I^2^* Pairwise** | ***I^2^* Network** | ***I^2^* Inconsistency (*p*-value)** |
| --- | --- | --- | --- | --- |
| Luseogliflozin | Placebo | 44.25 | 44.37 | NA |
| Ipragliflozin | Placebo | 47.48 | 47.50 | NA |
| Canagliflozin | Placebo | 21.08 | 20.68 | NA |
| Dapagliflozin | Empagliflozin | 42.82 | 67.09 | 0.07 |
| Dapagliflozin | Remogliflozin | NA | 75.20 | 0.05 |
| Dapagliflozin | Placebo | 40.22 | 54.72 | 0.0006 |
| Empagliflozin | Sotagliflozin | NA | 0.00 | 0.98 |
| Empagliflozin | Placebo | 38.57 | 29.12 | 0.16 |
| Ertugliflozin | Placebo | 0.00 | 0.00 | NA |
| Sotagliflozin | Placebo | 42.45 | 44.38 | NA |
| Remogliflozin | Placebo | 0.00 | 40.33 | 0.17 |
| Global | | 35.60 | 36.41 | NA |

## 11.2 Pollakiuria Model

Network I^2^ (from NMA in all trials, 50 data points, ratio 1.08)=9%

DIC = 90.40 (Dbar =51.02; pD = 39.38)

## 11.2 Heterogeneity score by each comparison for main model of pollakiuria

| **Treatment1** | **Treatment2** | ***I^2^* Pairwise** | ***I^2^* Network** | ***I^2^* Inconsistency (*p*-value)** |
| --- | --- | --- | --- | --- |
| Luseogliflozin | Placebo | 0.00 | 0.00 | NA |
| Ipragliflozin | Placebo | 63.17 | 63.11 | NA |
| Canagliflozin | Placebo | 40.36 | 40.31 | NA |
| Dapagliflozin | Empagliflozin | 0.00 | 17.72 | 0.18 |
| Dapagliflozin | Placebo | NA | 42.31 | 0.52 |
| Empagliflozin | Placebo | 25.45 | 42.23 | NA |
| Ertugliflozin | Placebo | 49.01 | 49.98 | NA |
| Sotagliflozin | Placebo | NA | NA | NA |
| Global | | 41.40 | 45.56 | NA |

## 11.3 Hypovolemia Model

Network I^2^ (from NMA in all trials, 80 data points, ratio 0.94)=0%

DIC = 130.94 (Dbar = 75.45; pD = 55.49)

## 11.3 Heterogeneity score by each comparison for main model of hypovolemia

| **Treatment1** | **Treatment2** | ***I^2^* Pairwise** | ***I^2^* Network** | ***I^2^* Inconsistency (*p*-value)** |
| --- | --- | --- | --- | --- |
| Luseogliflozin | Placebo | 0.00 | 0.00 | NA |
| Ipragliflozin | Placebo | 0.00 | 0.00 | NA |
| Canagliflozin | Placebo | 0.00 | 0.00 | NA |
| Dapagliflozin | Empagliflozin | NA | 0.00 | 0.93 |
| Dapagliflozin | Placebo | 73.95 | 72.28 | 0.90 |
| Empagliflozin | Placebo | 0.00 | 0.00 | 0.99 |
| Ertugliflozin | Placebo | 36.18 | 35.56 | NA |
| Tofogliflozin | Placebo | 0.00 | 0.00 | NA |
| Global | | 45.95 | 45.45 | NA |

11.4 Renal impairment or failure Model

Network I^2^ (from NMA in all trials, 38 data points, ratio 1.08)=10%

DIC = 67.34 (Dbar = 40.96; pD = 26.38)

## 11.4 Heterogeneity score by each comparison for main model of renal impairment or failure

| **Treatment1** | **Treatment2** | ***I^2^* Pairwise** | ***I^2^* Network** | ***I^2^* Inconsistency (*p*-value)** |
| --- | --- | --- | --- | --- |
| Luseogliflozin | Placebo | NA | NA | NA |
| Canagliflozin | Placebo | 63.95 | 63.94 | NA |
| Dapagliflozin | Placebo | 11.45 | 11.65 | NA |

## 11.5 Acute kidney injury Model

Network I^2^ (from NMA in all trials, 18 data points, ratio 1.21)=22%

DIC = 37.64 (Dbar = 21.71; pD = 15.93)

## 11.5 Heterogeneity score by each comparison for main model of acute kidney injury

| **Treatment1** | **Treatment2** | ***I^2^* Pairwise** | ***I^2^* Network** | ***I^2^* Inconsistency (*p*-value)** |
| --- | --- | --- | --- | --- |
| Canagliflozin | Placebo | 29.76 | 29.10 | NA |
| Ertugliflozin | Placebo | NA | NA | NA |
| Placebo | Sotaglifozin | 70.95 | 70.97 | NA |
| Empagliflozin | Placebo | NA | NA | NA |
| Dapagliflozin | Placebo | 83.13 | 77.20 | NA |
| Global | | 76.08 | 70.65 | NA |

## 11.6 [Urinary tract infection](javascript:;)s Model

Network I^2^ (from NMA in all trials, 172 data points, ratio 0.96)=0%

DIC = 269.09 (Dbar = 165.30; pD = 103.79)

## 11.6 Heterogeneity score by each comparison for main model of [urinary tract infection](javascript:;)s

| **Treatment1** | **Treatment2** | ***I^2^* Pairwise** | ***I^2^* Network** | ***I^2^* Inconsistency (*p*-value)** |
| --- | --- | --- | --- | --- |
| Ipragliflozin | Placebo | 0.00 | 0.00 | NA |
| Canagliflozin | Placebo | 0.00 | 0.00 | NA |
| Dapagliflozin | Empagliflozin | 0.00 | 47.60 | 0.08 |
| Dapagliflozin | Ertugliflozin | NA | 0.00 | 0.51 |
| Dapagliflozin | Remogliflozin | NA | 8.47 | 0.28 |
| Dapagliflozin | Placebo | 19.01 | 22.82 | NA |
| Empagliflozin | Sotagliflozin | NA | 0.00 | 0.65 |
| Empagliflozin | Placebo | 0.00 | 0.00 | 0.06 |
| Ertugliflozin | Placebo | 34.32 | 36.10 | NA |
| Sotagliflozin | Placebo | 5.94 | 6.78 | NA |
| Remogliflozin | Placebo | 54.56 | 35.89 | 0.21 |
| Tofogliflozin | Placebo | 57.56 | 58.23 | NA |
| Global | | 2.78 | 4.87 | NA |

## 11.7 Fracture Model

Network I^2^ (from NMA in all trials, 40 data points, ratio 1.14)=14%

DIC = 76.89 (Dbar = 45.59; pD = 31.30)

## 11.7 Heterogeneity score by each comparison for main model of fracture

| **Treatment1** | **Treatment2** | ***I^2^* Pairwise** | ***I^2^* Network** | ***I^2^* Inconsistency (*p*-value)** |
| --- | --- | --- | --- | --- |
| Canagliflozin | Placebo | 80.60 | 80.91 | NA |
| Ertugliflozin | Placebo | NA | NA | NA |
| Placebo | Sotaglifozin | 52.19 | 52.06 | NA |
| Empagliflozin | Placebo | 20.11 | 18.02 | NA |
| Dapagliflozin | Placebo | 23.85 | 23.05 | NA |
| Global | | 60.58 | 60.77 | NA |

## 11.8 [Diabetic ketoacidosis](javascript:;) Model

Network I^2^ (from NMA in all trials, 14 data points, ratio 1.14)=18%

DIC = 29.82 (Dbar = 15.91; pD = 13.90)

## 11.8 Heterogeneity score by each comparison for main model of [diabetic ketoacidosis](javascript:;)

| **Treatment1** | **Treatment2** | ***I^2^* Pairwise** | ***I^2^* Network** | ***I^2^* Inconsistency (*p*-value)** |
| --- | --- | --- | --- | --- |
| Canagliflozin | Placebo | NA | NA | NA |
| Dapagliflozin | Placebo | NA | NA | NA |
| Empagliflozin | Placebo | NA | NA | NA |
| Ertugliflozin | Placebo | NA | NA | NA |
| Placebo | Sotaglifozin | 69.37 | 69.19 | NA |
| Placebo | Tofogliflozin | NA | NA | NA |
| Global | | 61.68 | 61.24 | NA |

## 11.9 severe hypoglycemia Model

Network I^2^ (from NMA in all trials, 26 data points, ratio 1.12)=14%

DIC =49.09 (Dbar = 29.00; pD = 20.09)

## 11.9 Heterogeneity score by each comparison for main model of severe hypoglycemia

| **Treatment1** | **Treatment2** | ***I^2^* Pairwise** | ***I^2^* Network** | ***I^2^* Inconsistency (*p*-value)** |
| --- | --- | --- | --- | --- |
| Canagliflozin | Placebo | 69.78 | 67.01 | NA |
| Ertugliflozin | Placebo | 47.88 | 47.50 | NA |
| Sotaglifozin | Placebo | 62.88 | 56.23 | NA |
| Empagliflozin | Dapagliflozin | NA | 37.35 | 0.32 |
| Empagliflozin | Placebo | 71.75 | 34.28 | 0.62 |
| Dapagliflozin | Placebo | 0.00 | 0.00 | 0.67 |
| Global | | 58.61 | 48.00 | NA |

11.10 [Amputation](javascript:;) Model

Network I^2^ (from NMA in all trials, 8 data points, ratio 1.08)=19%

DIC =16.00 (Dbar =8.61; pD = 7.40)

## 11.10 Heterogeneity score by each comparison for main model of [amputation](javascript:;)

| **Treatment1** | **Treatment2** | ***I^2^* Pairwise** | ***I^2^* Network** | ***I^2^* Inconsistency (*p*-value)** |
| --- | --- | --- | --- | --- |
| Dapagliflozin | Placebo | 46.14 | 50.06 | NA |
| Ertugliflozin | Placebo | NA | NA | NA |
| Placebo | Sotaglifozin | NA | NA | NA |
| Global | | 46.66 | 50.77 | NA |

# Appendix 12: Contribution plots by study outcome

## 12.1 Contribution plot for reproductive tract infections


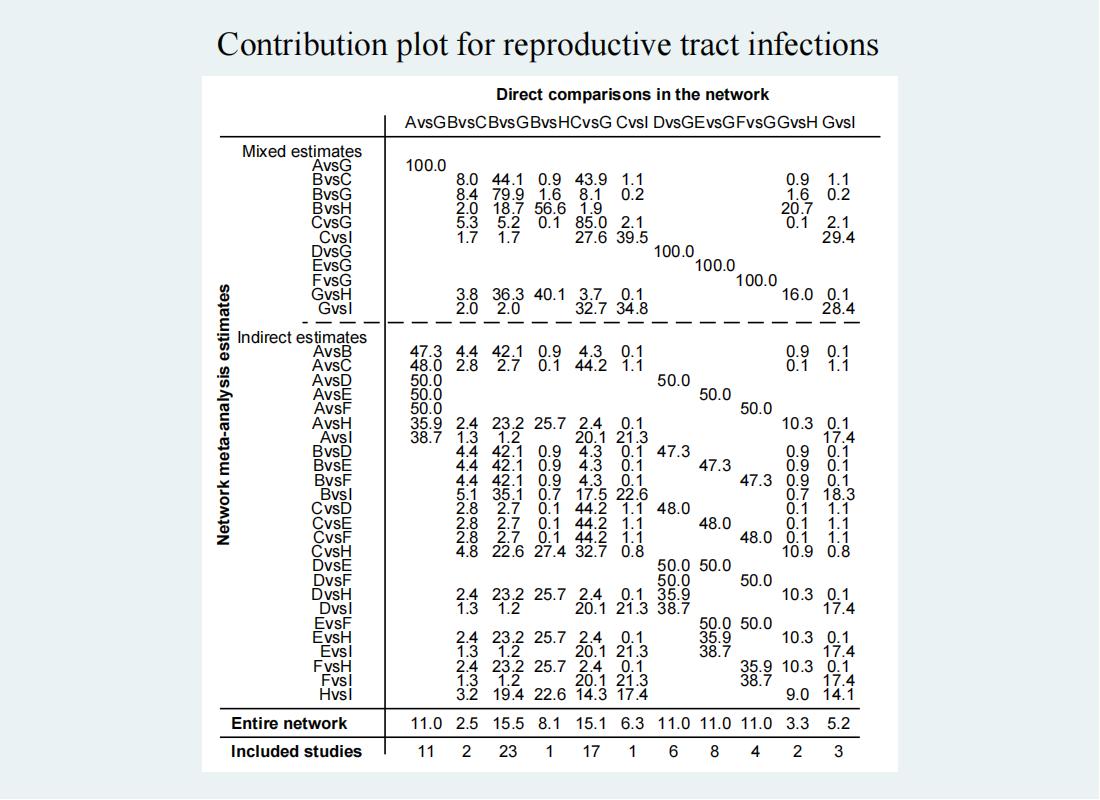


A: Treatment Canagliflozin, B: Treatment Dapagliflozin, C: Treatment Empagliflozin, D: Treatment Ertugliflozin, E: Treatment Ipragliflozin, F: Treatment Luseogliflozin, G: Treatment Placebo, H: Treatment Remogliflozin, I: Treatment Sotagliflozin

12.2 Contribution plot for pollakiuria


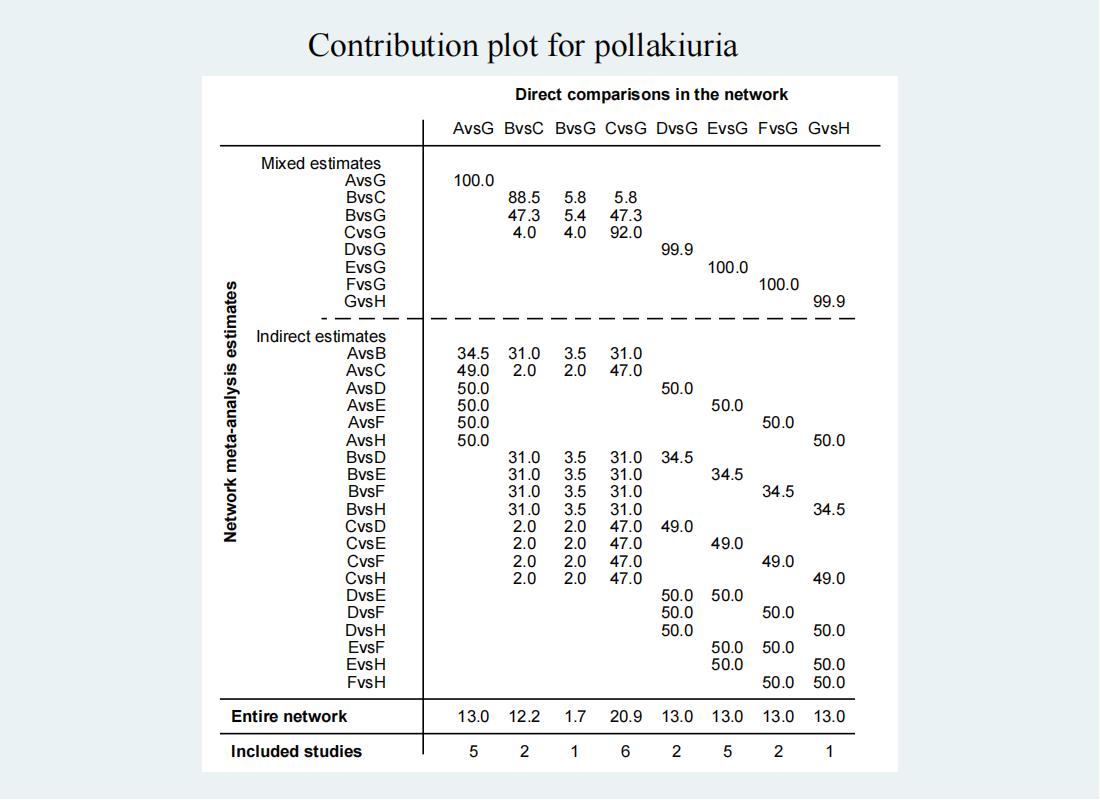


A: Treatment Canagliflozin, B: Treatment Dapagliflozin, C: Treatment Empagliflozin, D: Treatment Ertugliflozin, E: Treatment Ipragliflozin, F: Treatment Luseogliflozin, G: Treatment Placebo, H: Treatment Sotagliflozin

12.3 Contribution plot for hypovolemia


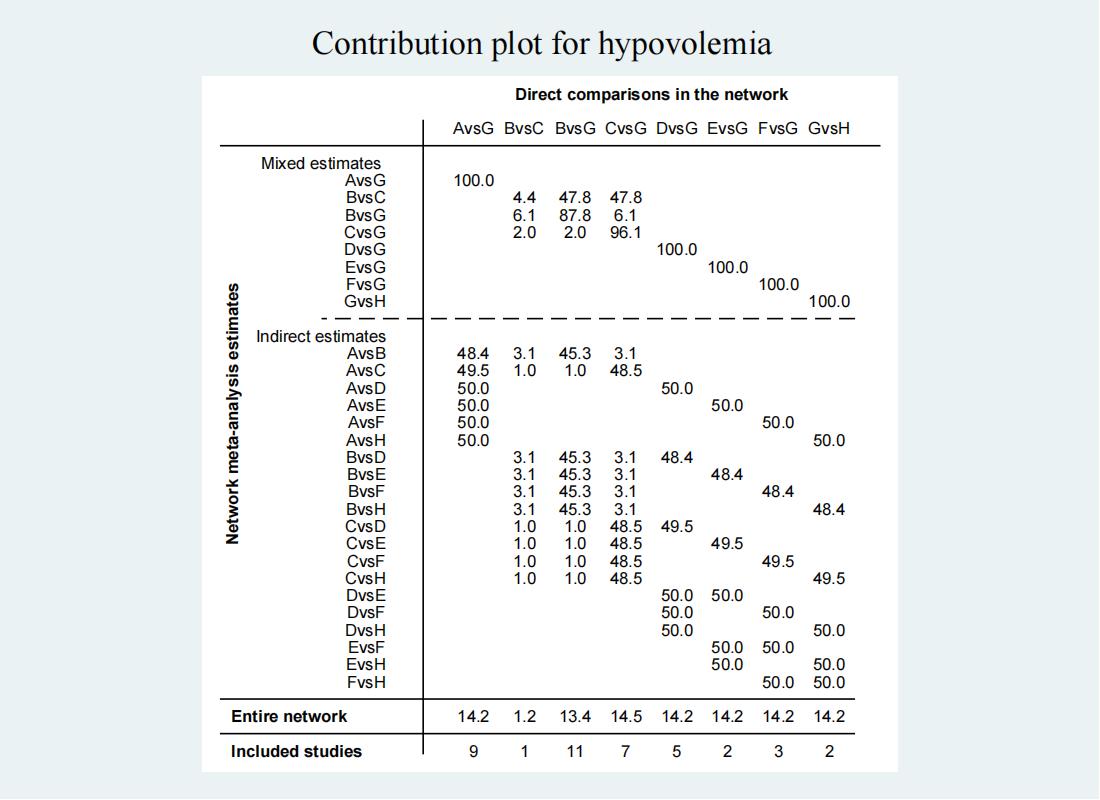


A: Treatment Canagliflozin, B: Treatment Dapagliflozin, C: Treatment Empagliflozin, D: Treatment Ertugliflozin, E: Treatment Ipragliflozin, F: Treatment Luseogliflozin, G: Treatment Placebo, H: Treatment Tofogliflozin

12.4 Contribution plot for renal impairment or failure


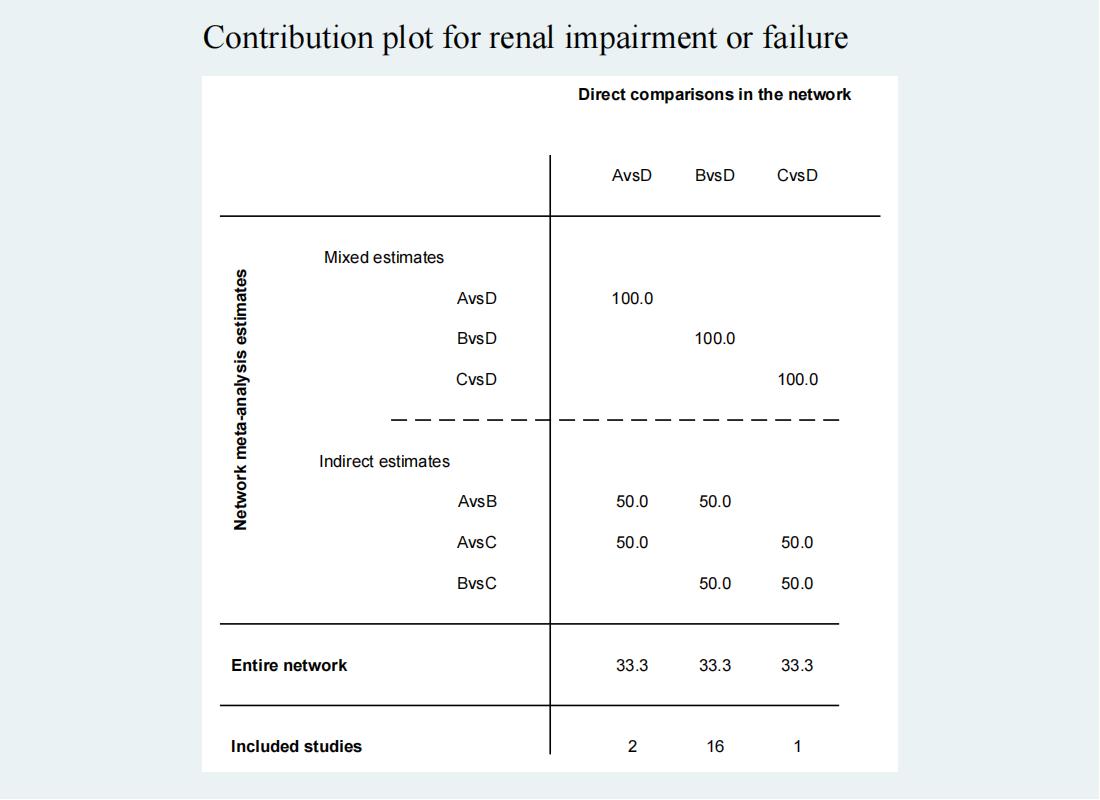


A: Treatment Canagliflozin, B: Treatment Dapagliflozin, C: Treatment Luseogliflozin, D: Treatment Placebo

12.5 Contribution plot for acute kidney injury


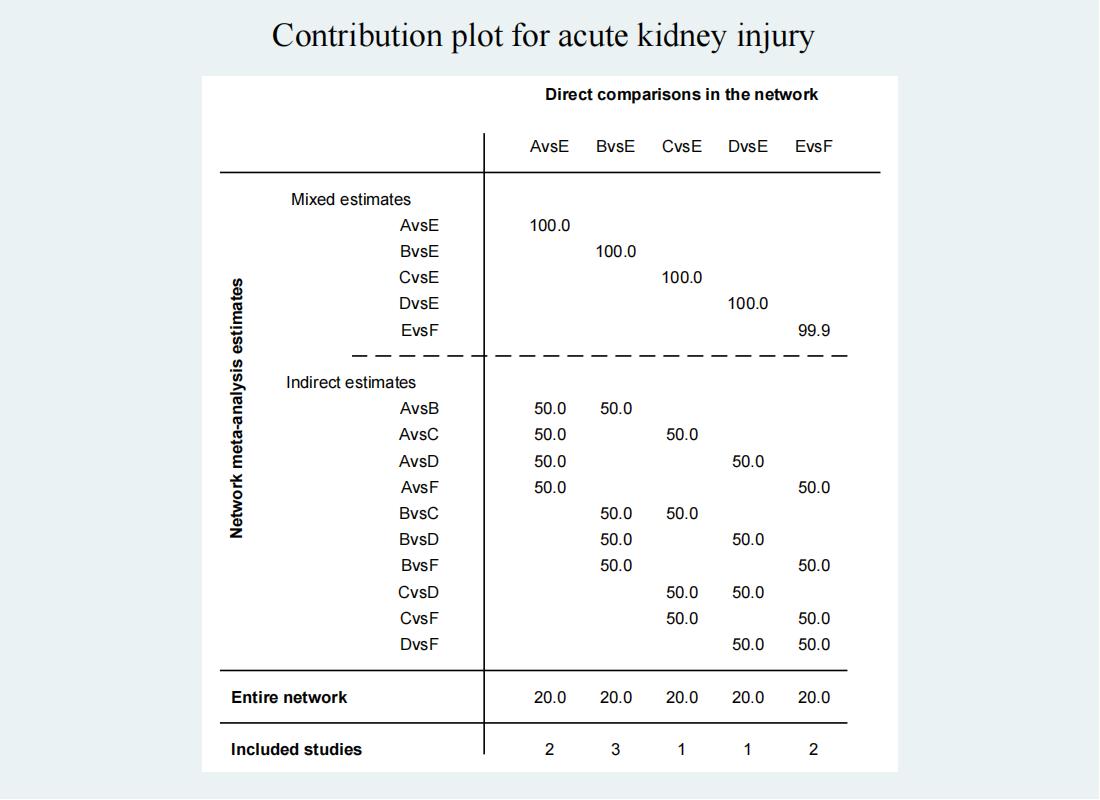


A: Treatment Canagliflozin, B: Treatment Dapagliflozin; C: Treatment Empagliflozin, D: Treatment Ertugliflozin, E: Treatment Placebo, F: Treatment Sotagliflozin

12.6 Contribution plot for urinary tract infections


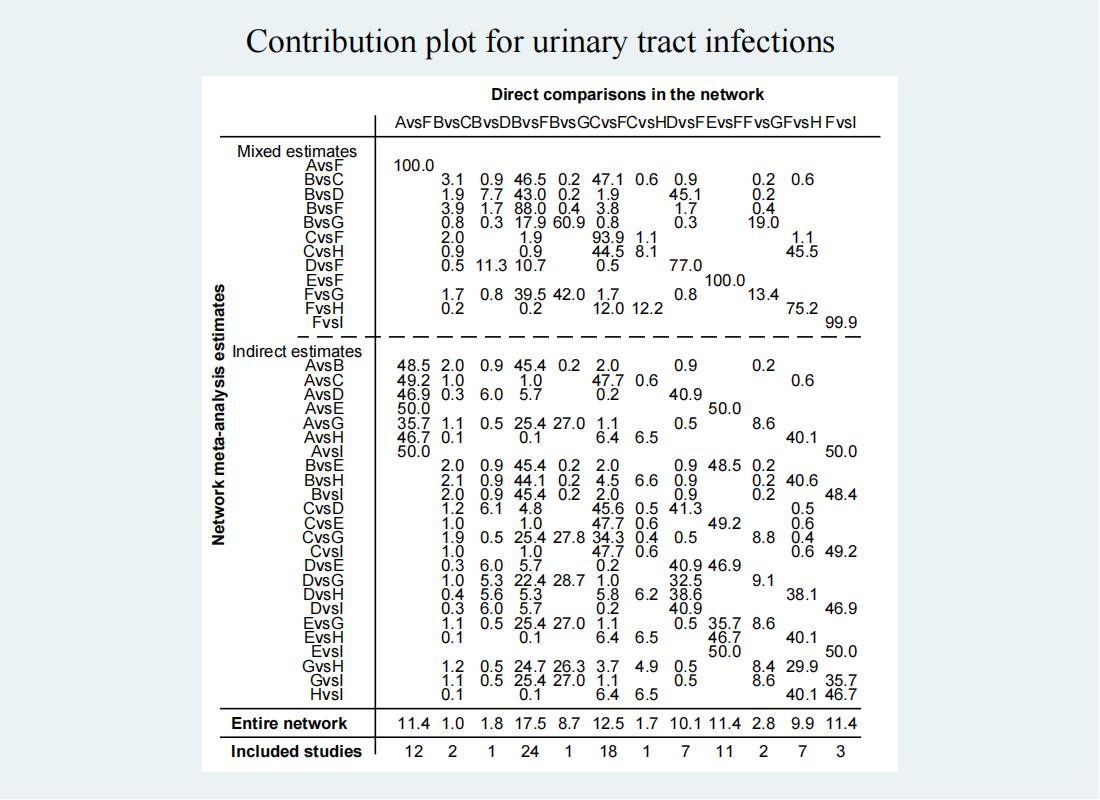


A: Treatment Canagliflozin, B: Treatment Dapagliflozin, C: Treatment Empagliflozin, D: Treatment Ertugliflozin, E: Treatment Ipragliflozin, F: Treatment Placebo, G: Treatment Remogliflozin, H: Treatment Sotagliflozin, I: Treatment Tofogliflozin

12.7 Contribution plot for amputation


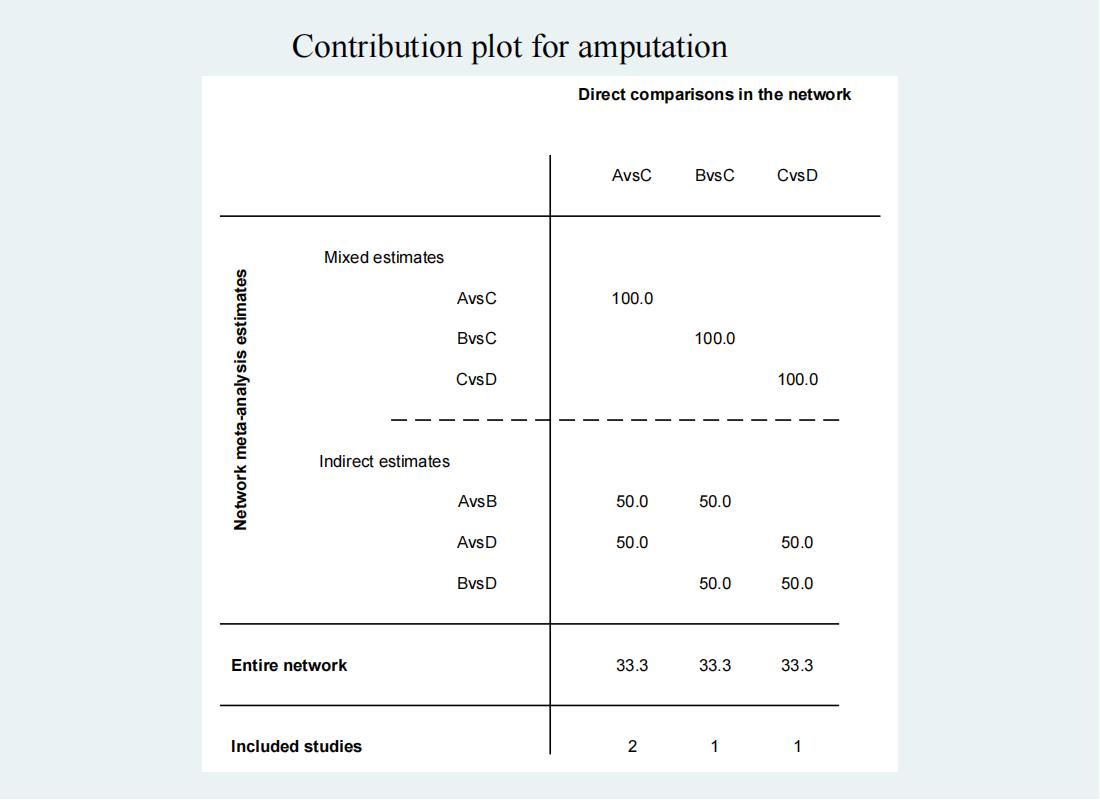


A: Treatment Dapagliflozin, B: Treatment Ertugliflozin, C: Treatment Placebo, D: Treatment Sotaglifozin

12.8 Contribution plot for diabetic ketoacidosis


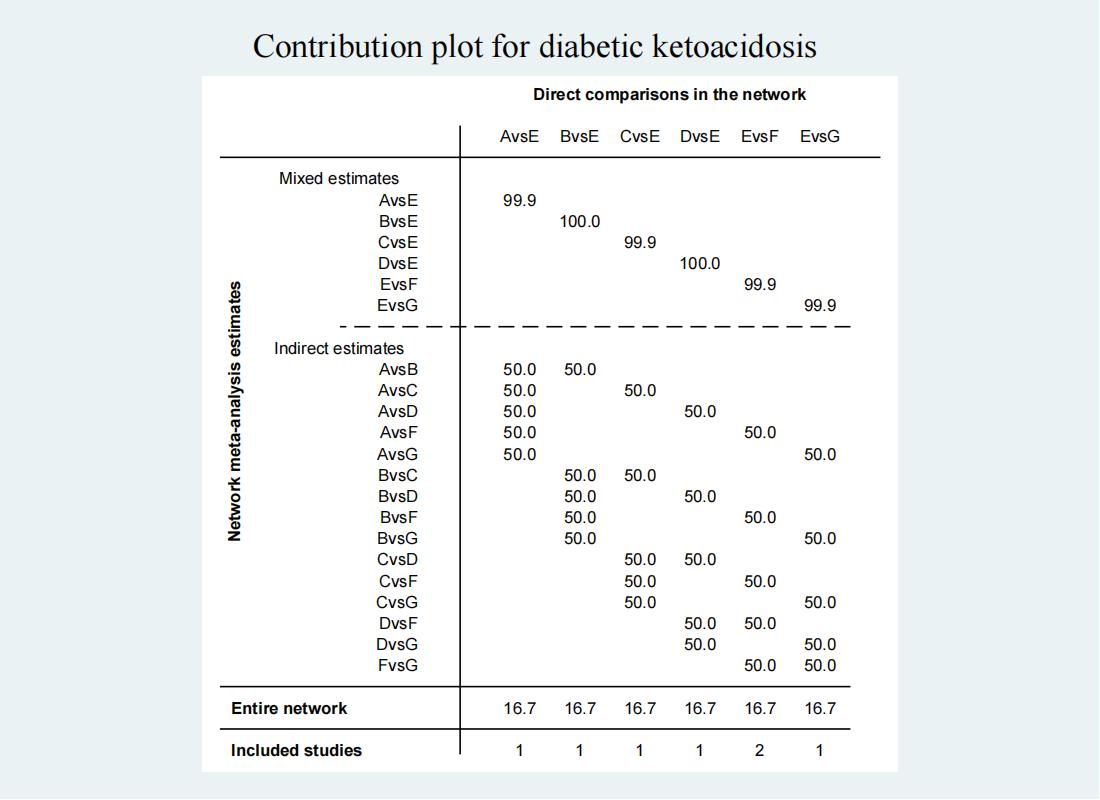


A: Treatment Canagliflozin, B: Treatment Dapagliflozin, C: Treatment Empagliflozin, D: Treatment Ertugliflozin, E: Treatment Placebo, F: Treatment Sotagliflozin, G: Treatment Tofogliflozin

12.9 Contribution plot for fracture


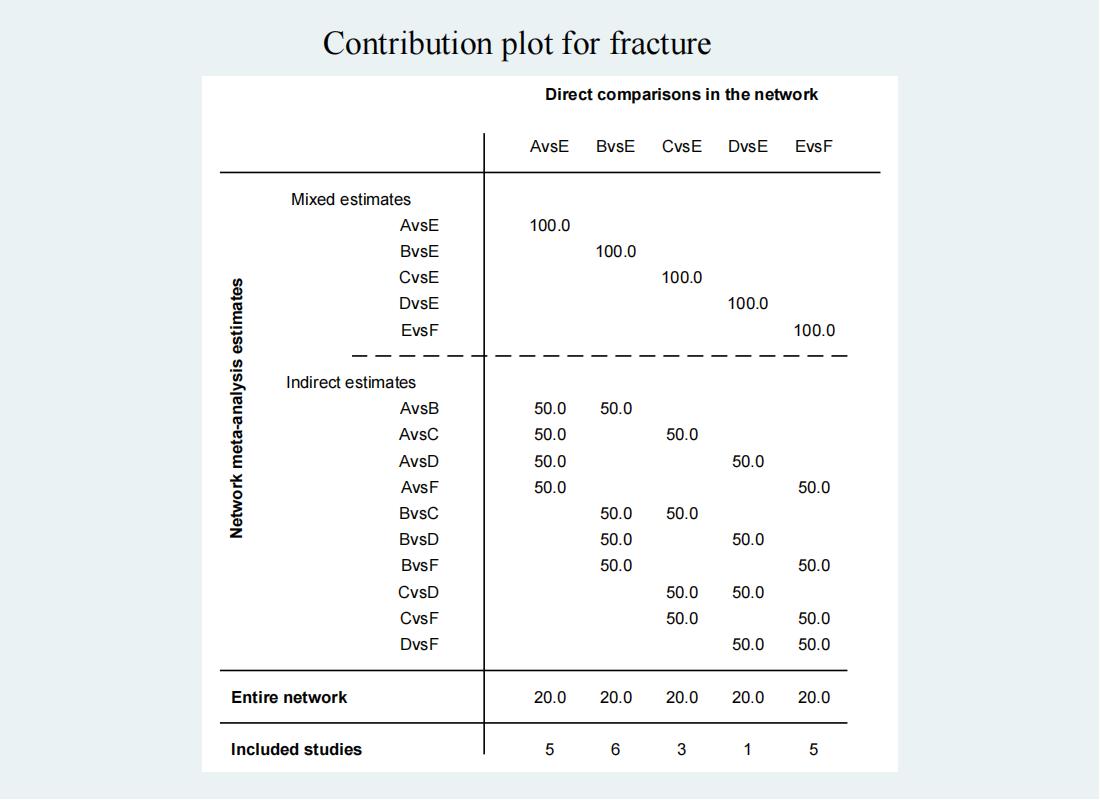


A: Treatment Canagliflozin, B: Treatment Dapagliflozin, C: Treatment Empagliflozin, D: Treatment Ertugliflozin, E: Treatment Placebo, F: Treatment Sotagliflozin

12.10 Contribution plot for severe hypoglycemia


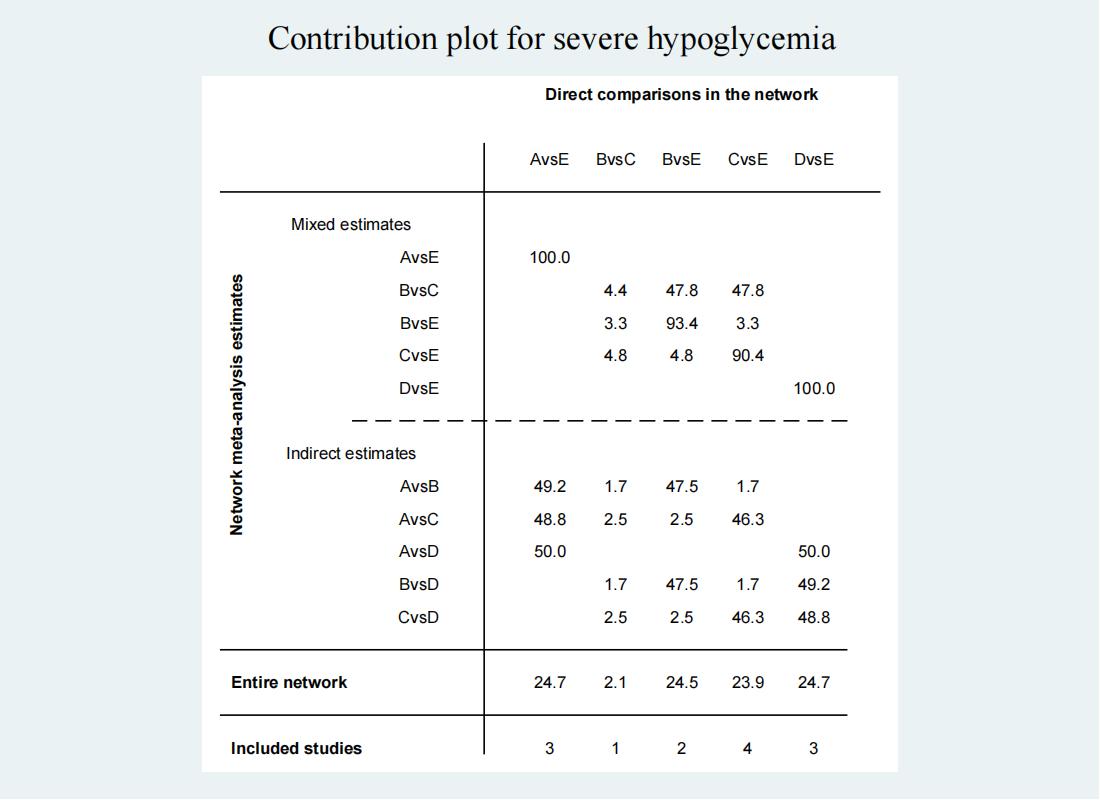


A: Treatment Canagliflozin, B: Treatment Dapagliflozin, C: Treatment Empagliflozin, D: Treatment Ertugliflozin, E: Treatment Placebo

12.11 Contribution plot for reproductive tract infections in male


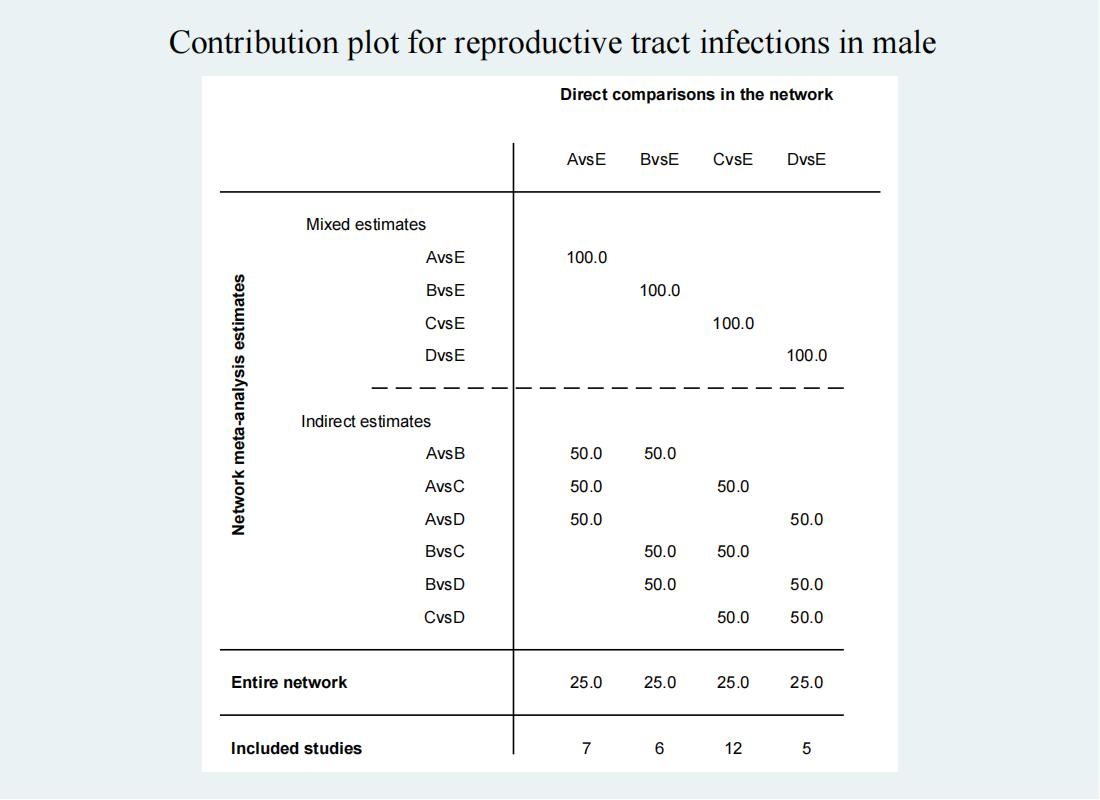


A: Treatment Canagliflozin, B: Treatment Dapagliflozin, C: Treatment Empagliflozin, D: Treatment Ertugliflozin, E: Treatment Placebo

12.12 Contribution plot for reproductive tract infections in female


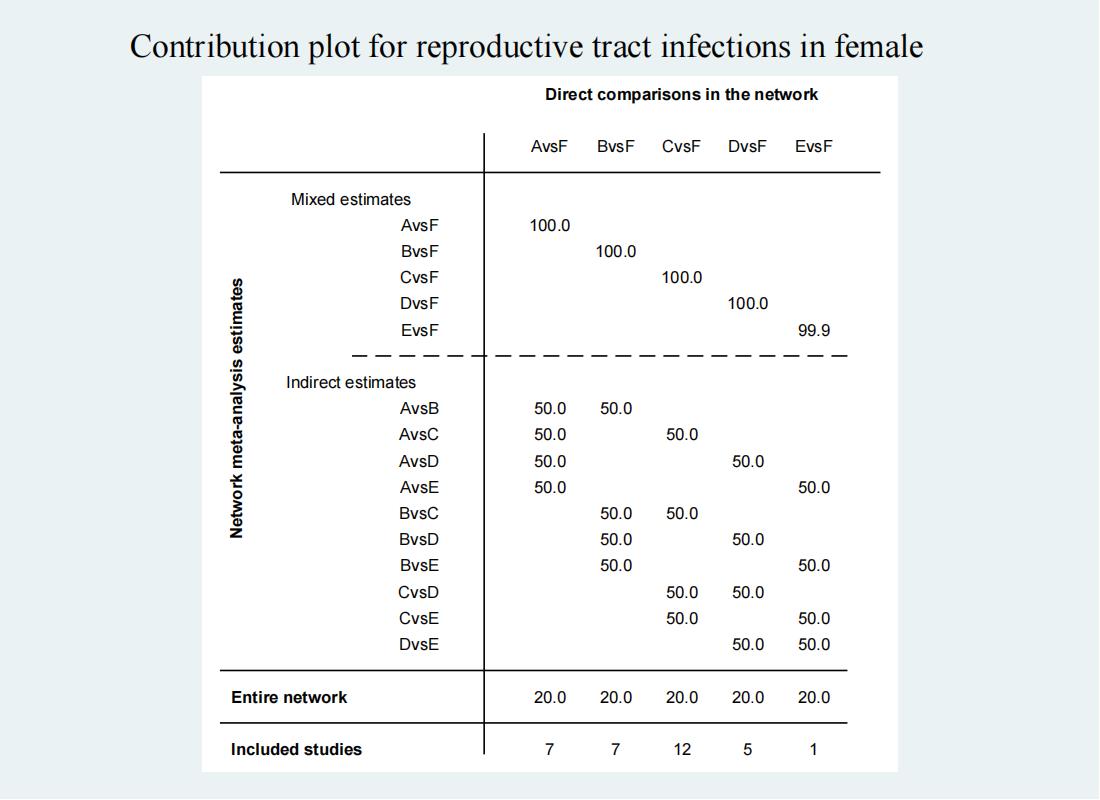


A: Treatment Canagliflozin, B: Treatment Dapagliflozin, C: Treatment Empagliflozin, D: Treatment Ertugliflozin, E: Treatment Ipragliflozin, F: Treatment Placebo

Appendix 13: Treatment Ranking using SUCRA

**13.1 Cumulative ranking curves for hypovolemia**


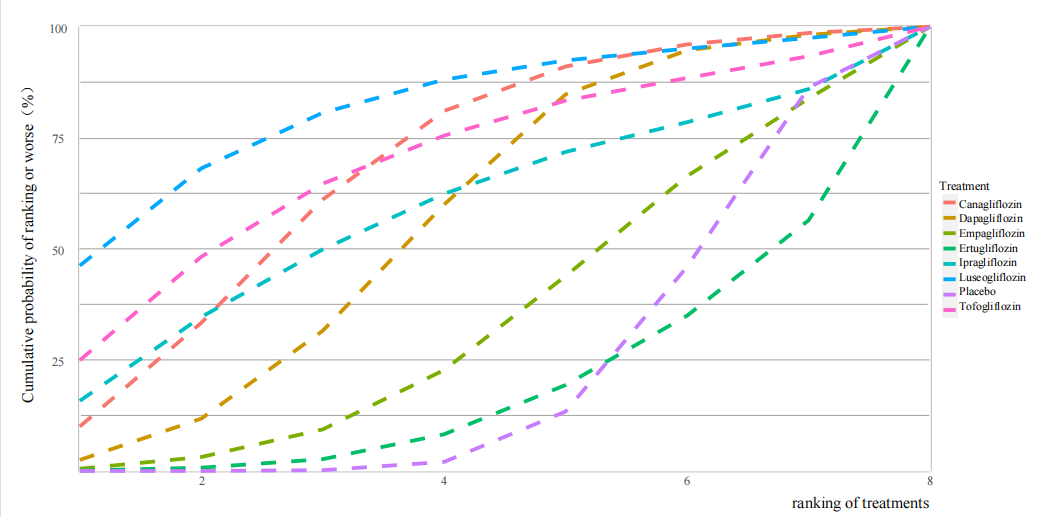


Graphs show the cumulative probability of each intervention ranking, from worst (rank 1) to best (rank 9) for each outcome. A rank indicates the probability that an intervention is worst, second worst, etc. For example, Luseogliflozin probably ranked worst for hypovolemia.

| **Treatment** | **Surface under the cumulative ranking** |
| --- | --- |
| Luseogliflozin | 0.81 |
| Tofogliflozin | 0.68 |
| Canagliflozin | 0.67 |
| Ipragliflozin | 0.57 |
| Dapagliflozin | 0.55 |
| Empagliflozin | 0.33 |
| Placebo | 0.21 |
| Ertugliflozin | 0.17 |

**13.2 Cumulative ranking curves for** **renal impairment or failure**


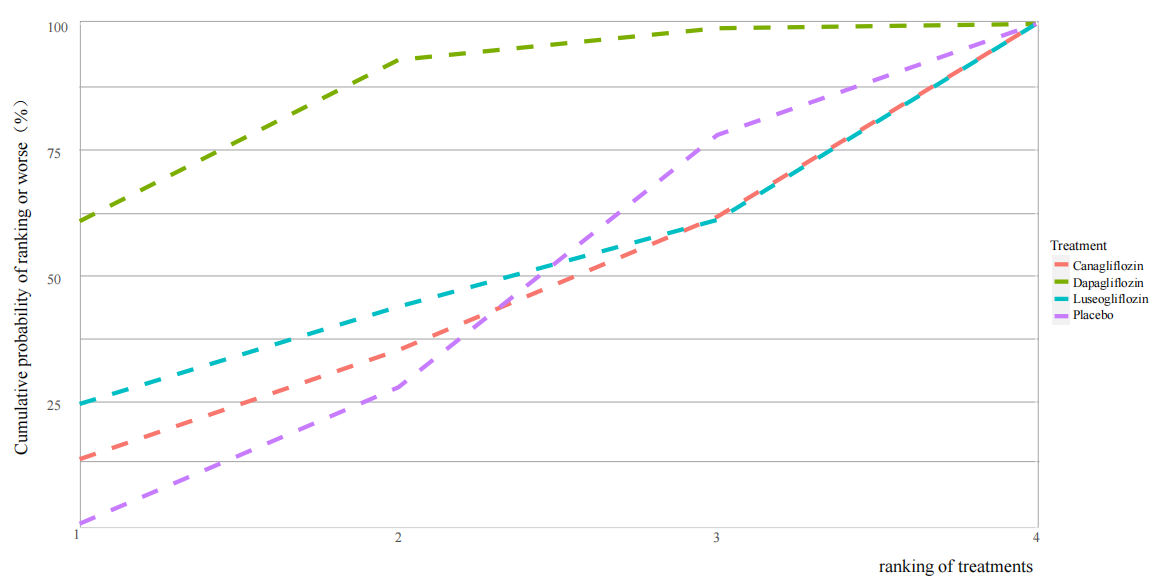


Graphs show the cumulative probability of each intervention ranking, from worst (rank 1) to best (rank 4) for each outcome. A rank indicates the probability that an intervention is worst, second worst, etc. For example, Dapagliflozin probably ranked worst for renal impairment or failure.

| **Treatment** | **Surface under the cumulative ranking** |
| --- | --- |
| Dapagliflozin | 0.84 |
| Luseogliflozin | 0.43 |
| Canagliflozin | 0.37 |
| Placebo | 0.36 |

**13.3 Cumulative ranking curves for acute kidney injury**


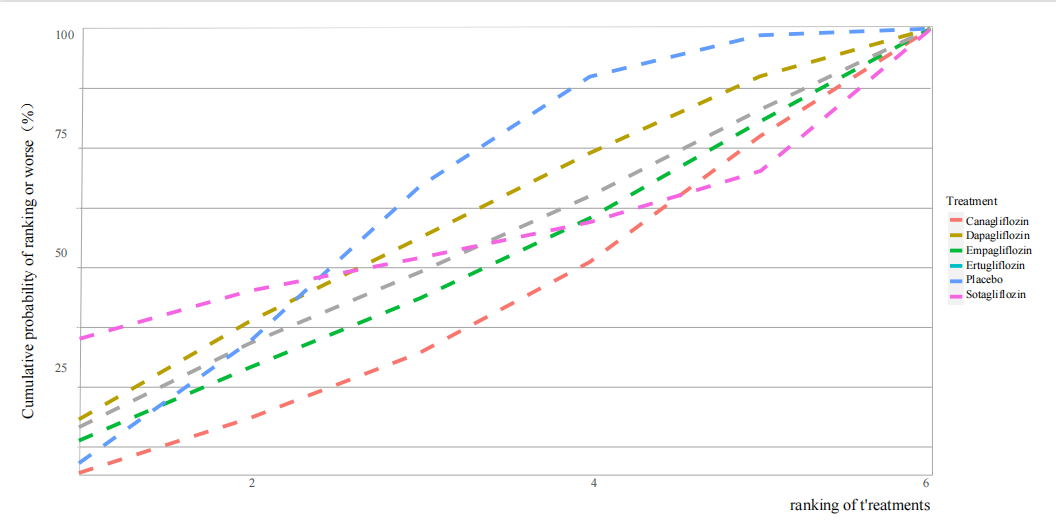


Graphs show the cumulative probability of each intervention ranking, from worst (rank 1) to best (rank 6) for each outcome. A rank indicates the probability that an intervention is worst, second worst, etc. For example, Placebo probably ranked worst for acute kidney injury.

| **Treatment** | **Surface under the cumulative ranking** |
| --- | --- |
| Placebo | 0.60 |
| Dapagliflozin | 0.55 |
| Sotaglifozin | 0.52 |
| Ertugliflozin | 0.50 |
| Empagliflozin | 0.45 |
| Canagliflozin | 0.37 |

**13.4 Cumulative ranking curves for urinary tract infections**


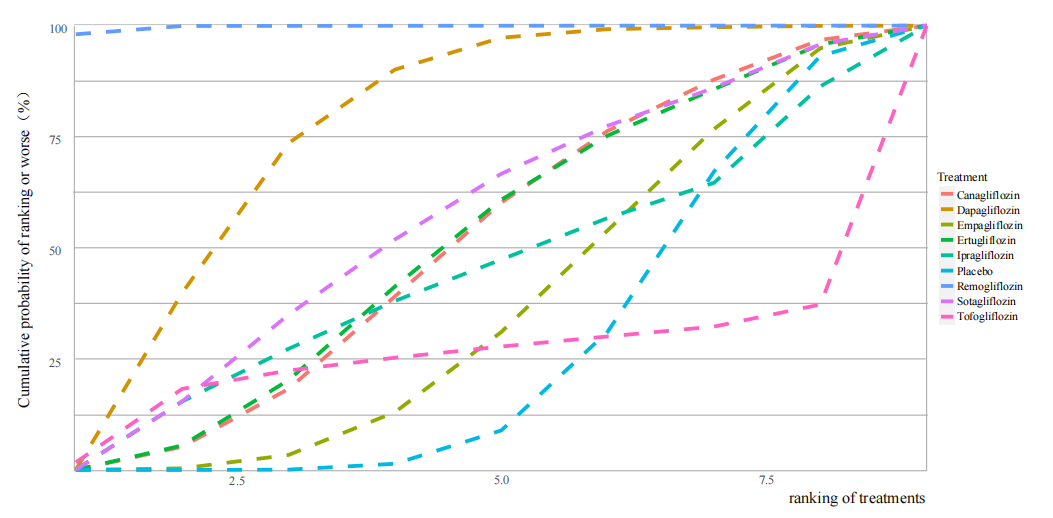


Graphs show the cumulative probability of each intervention ranking, from worst (rank 1) to best (rank 9) for each outcome. A rank indicates the probability that an intervention is worst, second worst, etc. For example, Remogliflozin probably ranked worst for urinary tract infections.

| **Treatment** | **Surface under the cumulative ranking** |
| --- | --- |
| Remogliflozin | 1.00 |
| Dapagliflozin | 0.75 |
| Sotaglifozin | 0.53 |
| Ertugliflozin | 0.48 |
| Canagliflozin | 0.47 |
| Ipragliflozin | 0.42 |
| Empagliflozin | 0.33 |
| Tofogliflozin | 0.28 |
| Placebo | 0.25 |

**13.5 Cumulative ranking curves for amputation**


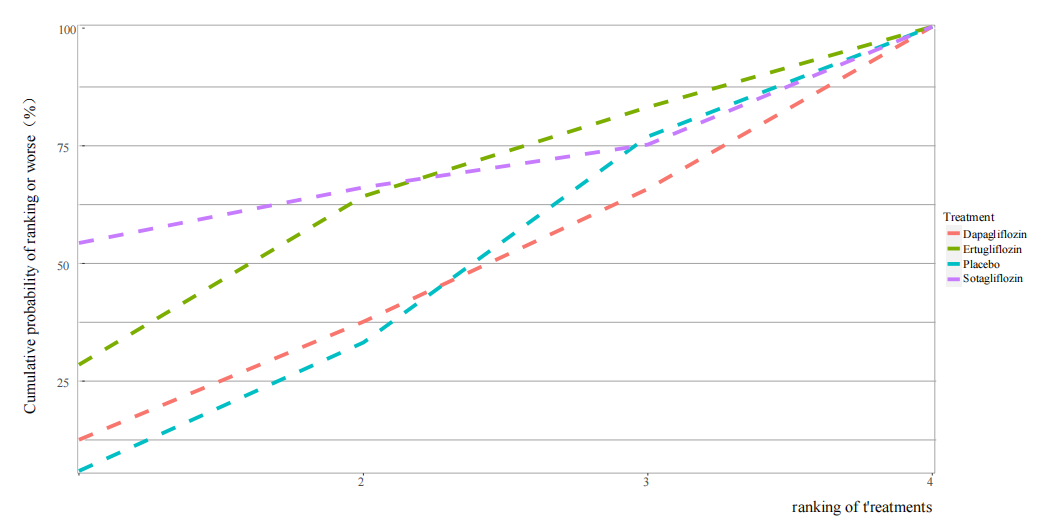


Graphs show the cumulative probability of each intervention ranking, from worst (rank 1) to best (rank 4) for each outcome. A rank indicates the probability that an intervention is worst, second worst, etc. For example, Sotaglifozin probably ranked worst for amputation.

| **Treatment** | **Surface under the cumulative ranking** |
| --- | --- |
| Sotaglifozin | 0.65 |
| Ertugliflozin | 0.58 |
| Dapagliflozin | 0.38 |
| Placebo | 0.38 |

**13.6 Cumulative ranking curves for [diabetic ketoacidosis](javascript:;)**


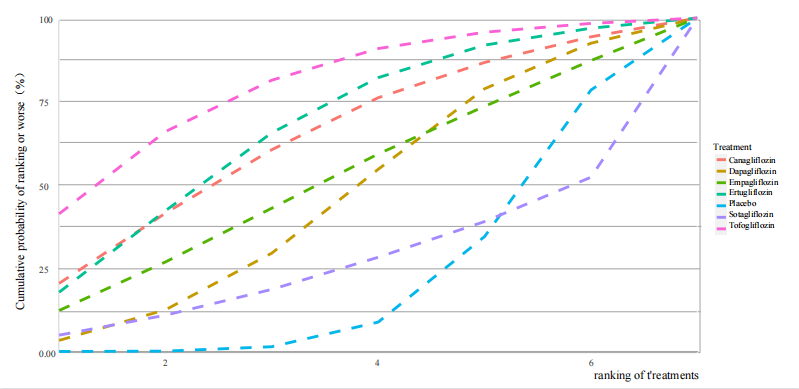


Graphs show the cumulative probability of each intervention ranking, from worst (rank 1) to best (rank 7) for each outcome. A rank indicates the probability that an intervention is worst, second worst, etc. For example, Tofogliflozin probably ranked worst for [diabetic ketoacidosis](javascript:;).

| **Treatment** | **Surface under the cumulative ranking** |
| --- | --- |
| Tofogliflozin | 0.79 |
| Ertugliflozin | 0.66 |
| Canagliflozin | 0.63 |
| Empagliflozin | 0.50 |
| Dapagliflozin | 0.45 |
| Sotaglifozin | 0.26 |
| Placebo | 0.21 |

**13.7 Cumulative ranking curves for fracture**


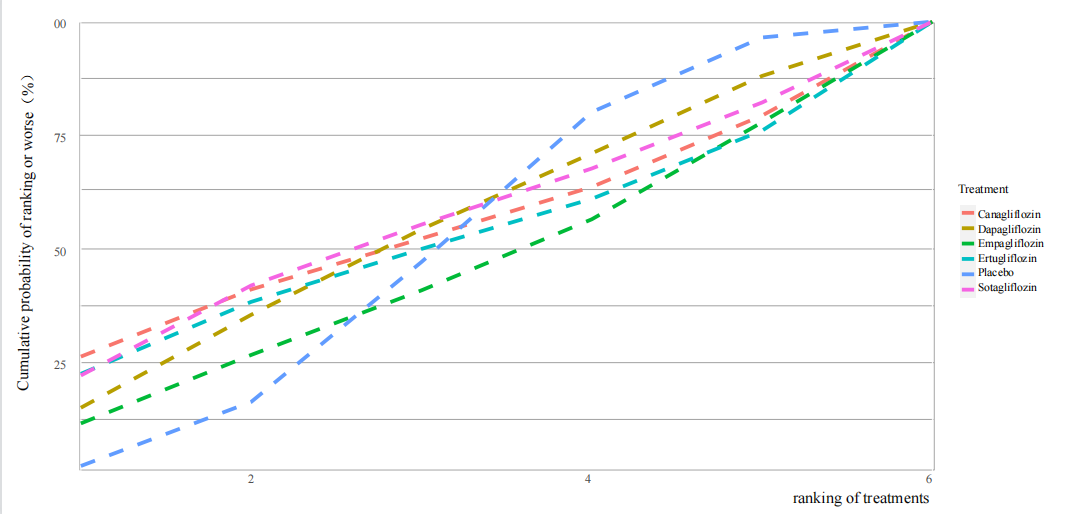


Graphs show the cumulative probability of each intervention ranking, from worst (rank 1) to best (rank 6) for each outcome. A rank indicates the probability that an intervention is worst, second worst, etc. For example, Sotaglifozin probably ranked worst for fracture.

| **Treatment** | **Surface under the cumulative ranking** |
| --- | --- |
| Sotaglifozin | 0.54 |
| Dapagliflozin | 0.528 |
| Canagliflozin | 0.525 |
| Ertugliflozin | 0.50 |
| Placebo | 0.48 |
| Empagliflozin | 0.43 |

**13.8 Cumulative ranking curves for** **severe hypoglycemia**


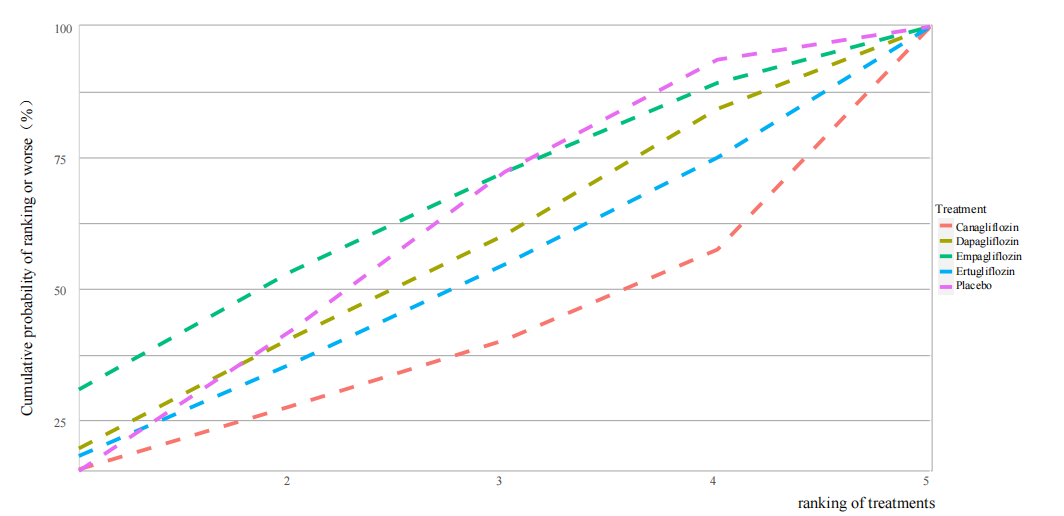


Graphs show the cumulative probability of each intervention ranking, from worst (rank 1) to best (rank 6) for each outcome. A rank indicates the probability that an intervention is worst, second worst, etc. For example, Empagliflozin probably ranked worst for severe hypoglycemia

| **Treatment** | **Surface under the cumulative ranking** |
| --- | --- |
| Empagliflozin | 0.61 |
| Placebo | 0.56 |
| Ertugliflozin | 0.46 |
| Canagliflozin | 0.35 |

# Appendix 14: Comparison-adjusted funnel plots

## 14.1 Comparison adjusted funnel plot for reproductive tract infections


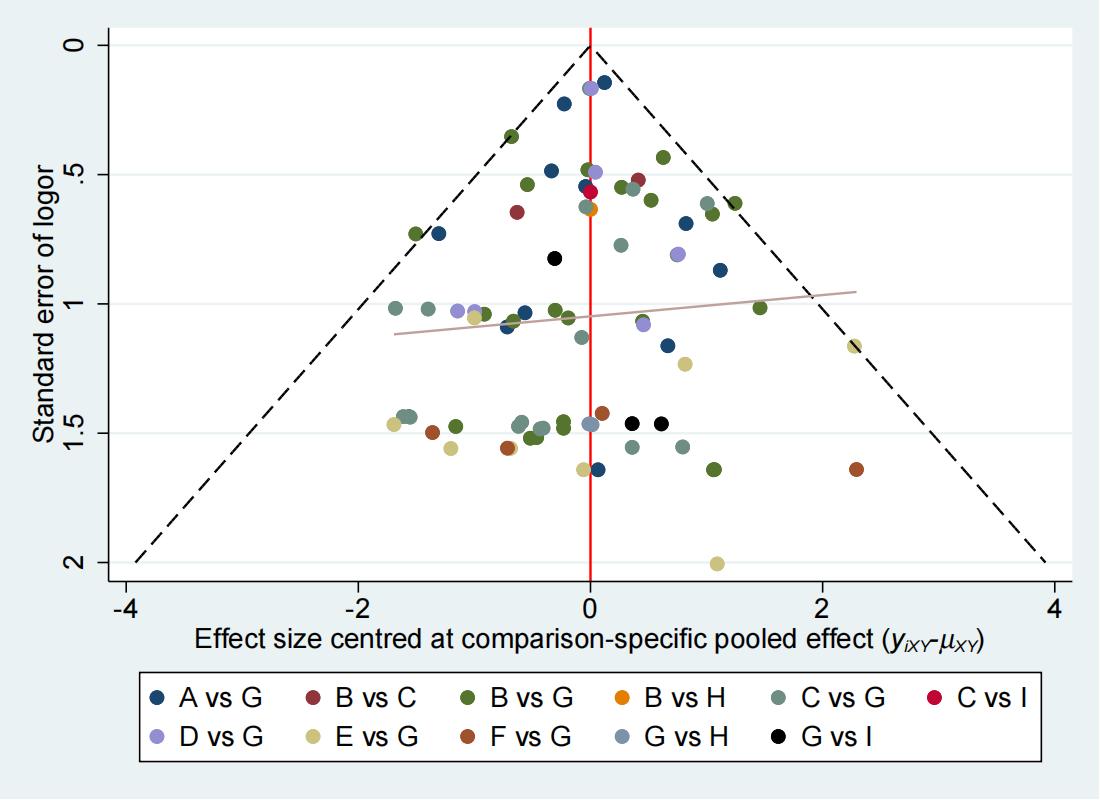


A: Treatment Canagliflozin, B: Treatment Dapagliflozin, C: Treatment Empagliflozin, D: Treatment Ertugliflozin, E: Treatment Ipragliflozin, F: Treatment Luseogliflozin, G: Treatment Placebo, H: Treatment Remogliflozin, I: Treatment Sotagliflozin

## 14.2 Comparison adjusted funnel plot for pollakiuria


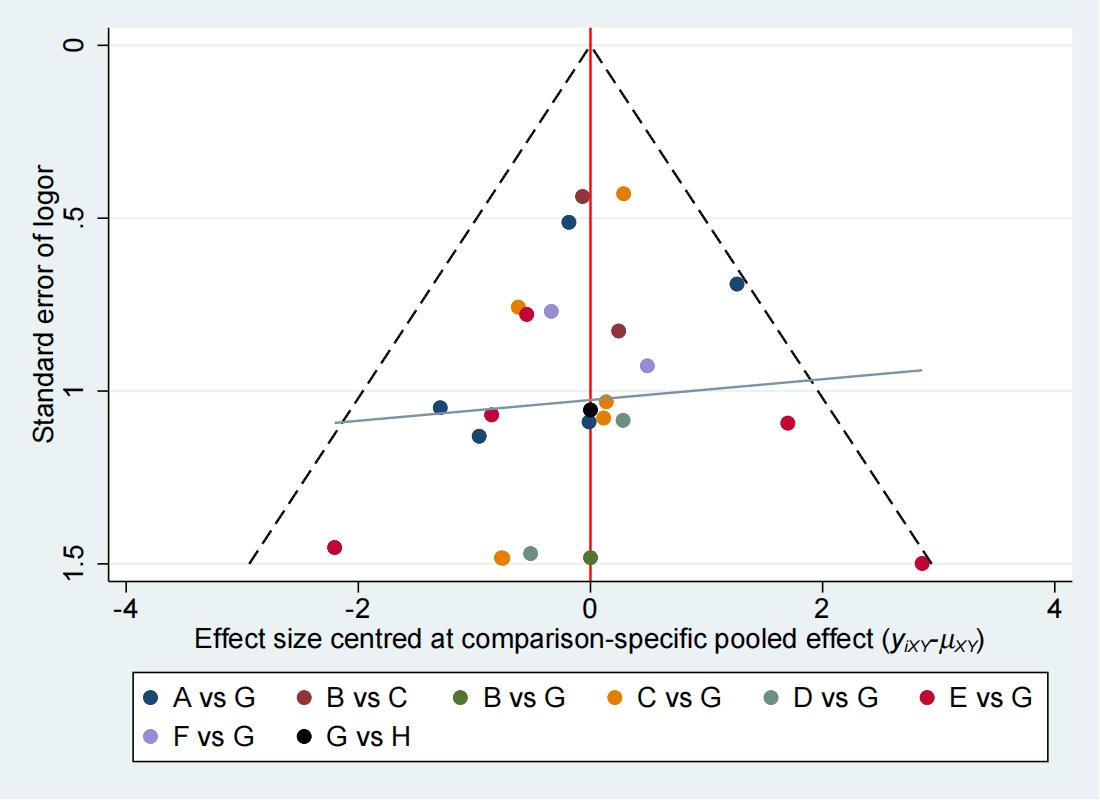


A: Treatment Canagliflozin, B: Treatment Dapagliflozin, C: Treatment Empagliflozin, D: Treatment Ertugliflozin, E: Treatment Ipragliflozin, F: Treatment Luseogliflozin, G: Treatment Placebo, H: Treatment Sotagliflozin

14.3 Comparison adjusted funnel plot for hypovolemia


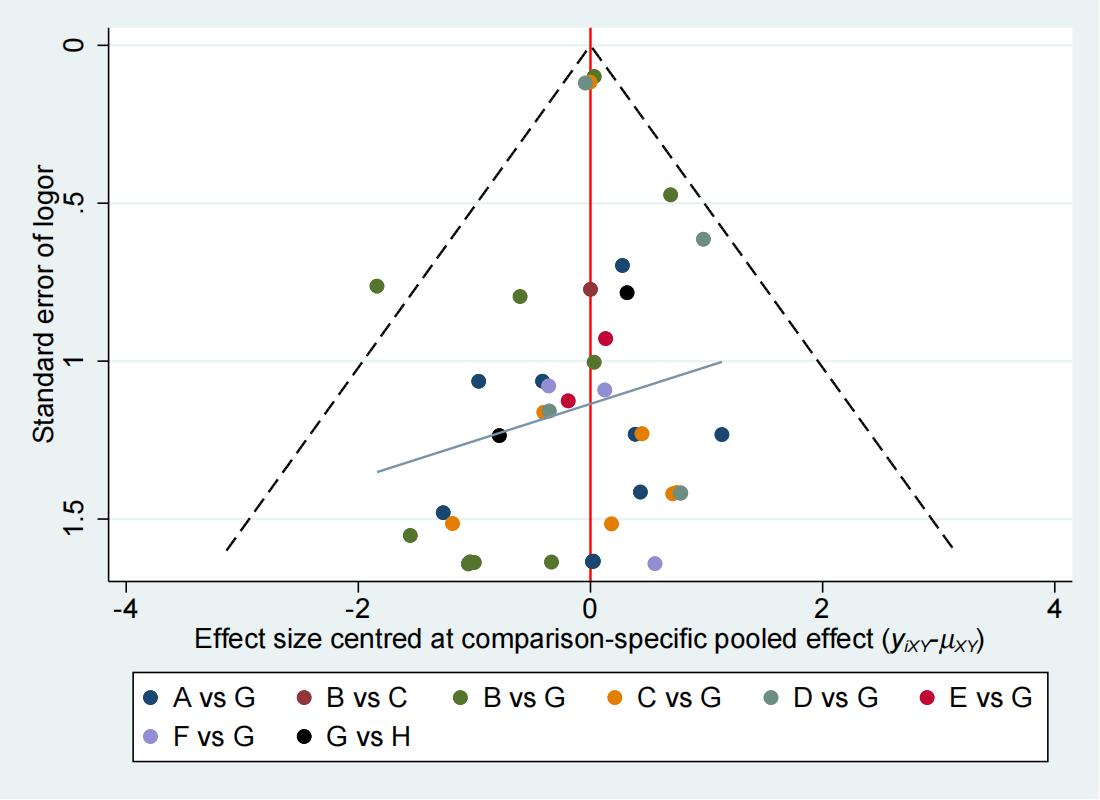


A: Treatment Canagliflozin, B: Treatment Dapagliflozin, C: Treatment Empagliflozin, D: Treatment Ertugliflozin, E: Treatment Ipragliflozin, F: Treatment Luseogliflozin, G: Treatment Placebo, H: Treatment Tofogliflozin

## 14.4 Comparison adjusted funnel plot for renal impairment or failure


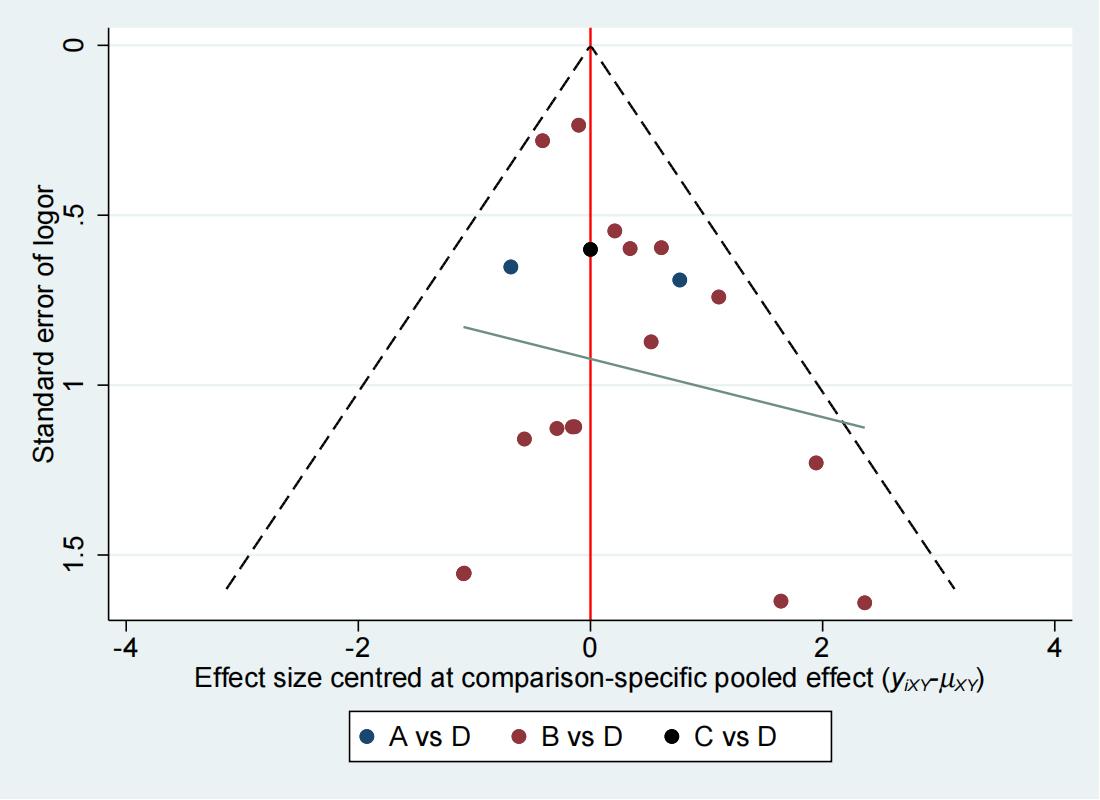


A: Treatment Canagliflozin, B: Treatment Dapagliflozin, C: Treatment Luseogliflozin, D: Treatment Placebo

14.5 Comparison adjusted funnel plot for acute kidney injury


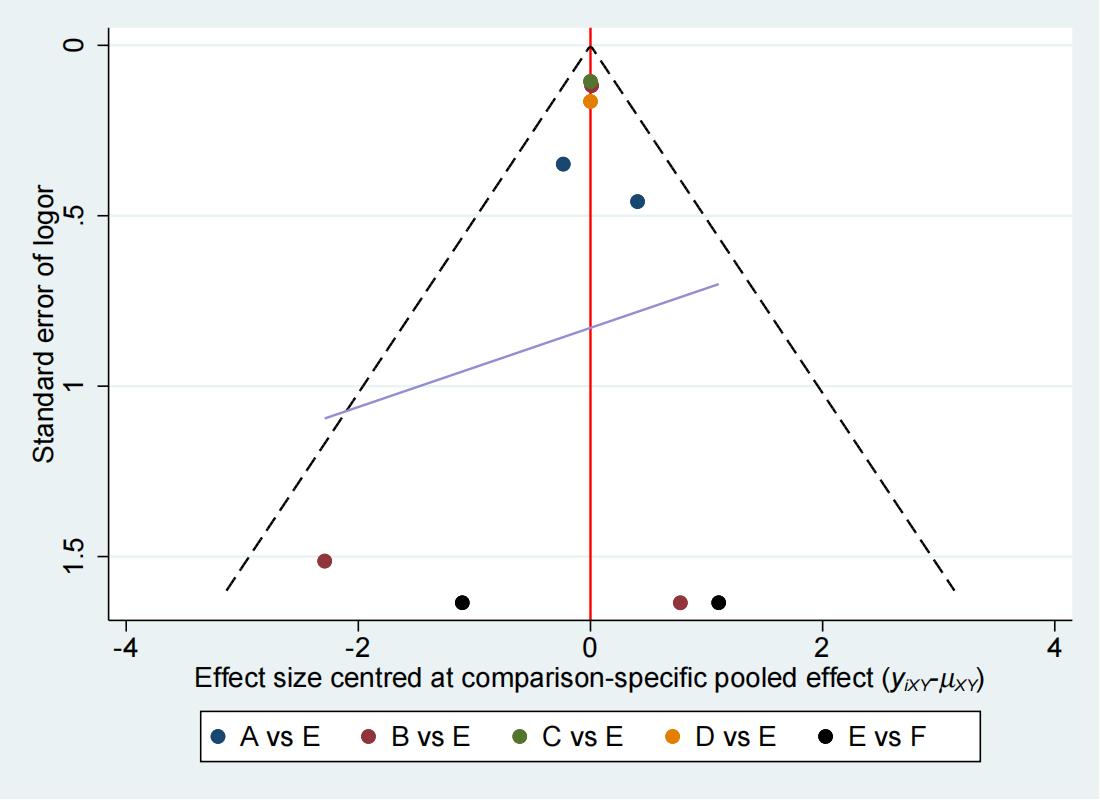


A: Treatment Canagliflozin, B: Treatment Dapagliflozin; C: Treatment Empagliflozin, D: Treatment Ertugliflozin, E: Treatment Placebo, F: Treatment Sotagliflozin

14.6 Comparison adjusted funnel plot for urinary tract infections


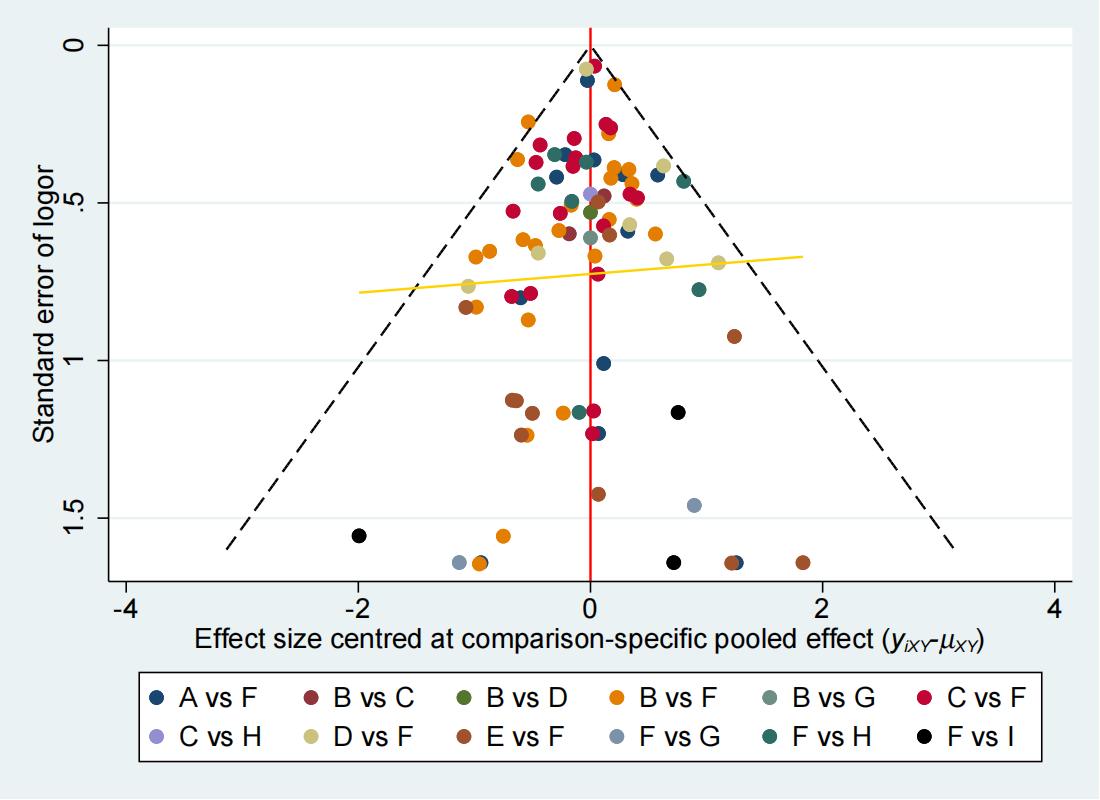


A: Treatment Canagliflozin, B: Treatment Dapagliflozin, C: Treatment Empagliflozin, D: Treatment Ertugliflozin, E: Treatment Ipragliflozin, F: Treatment Placebo, G: Treatment Remogliflozin, H: Treatment Sotagliflozin, I: Treatment Tofogliflozin

## 14.7 Comparison adjusted funnel plot for amputation


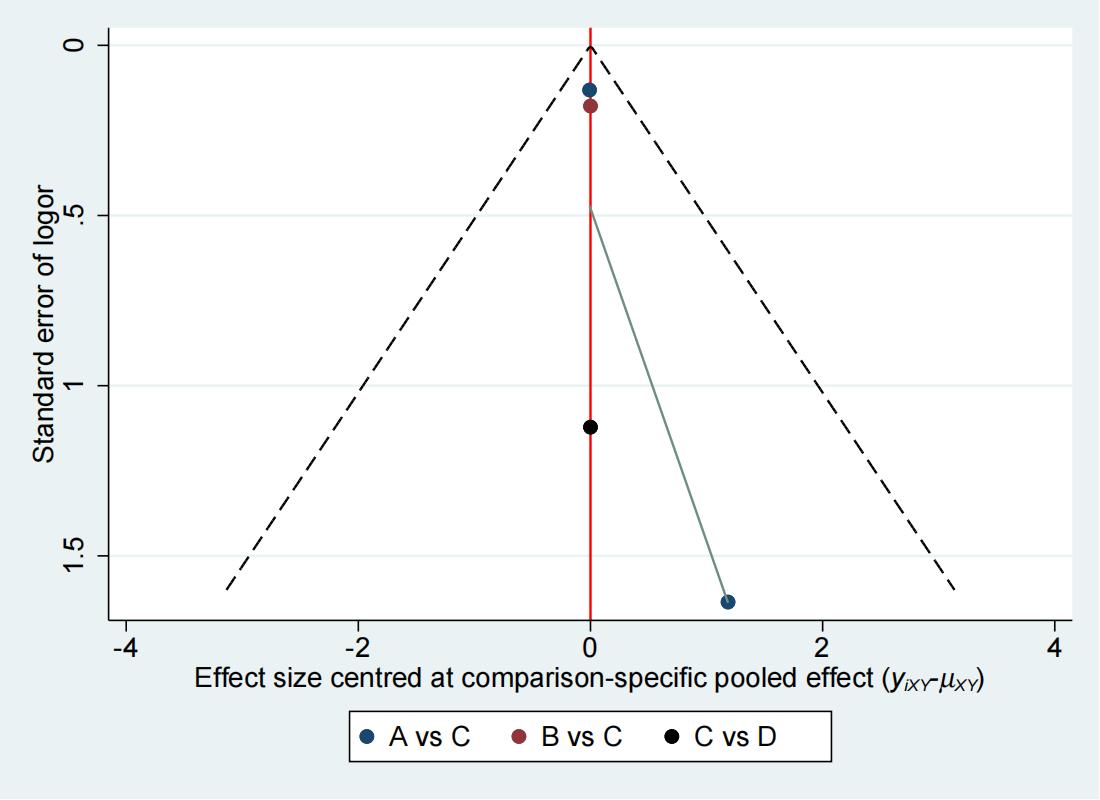


A: Treatment Dapagliflozin, B: Treatment Ertugliflozin, C: Treatment Placebo, D: Treatment Sotaglifozin

14.8 Comparison adjusted funnel plot for diabetic ketoacidosis


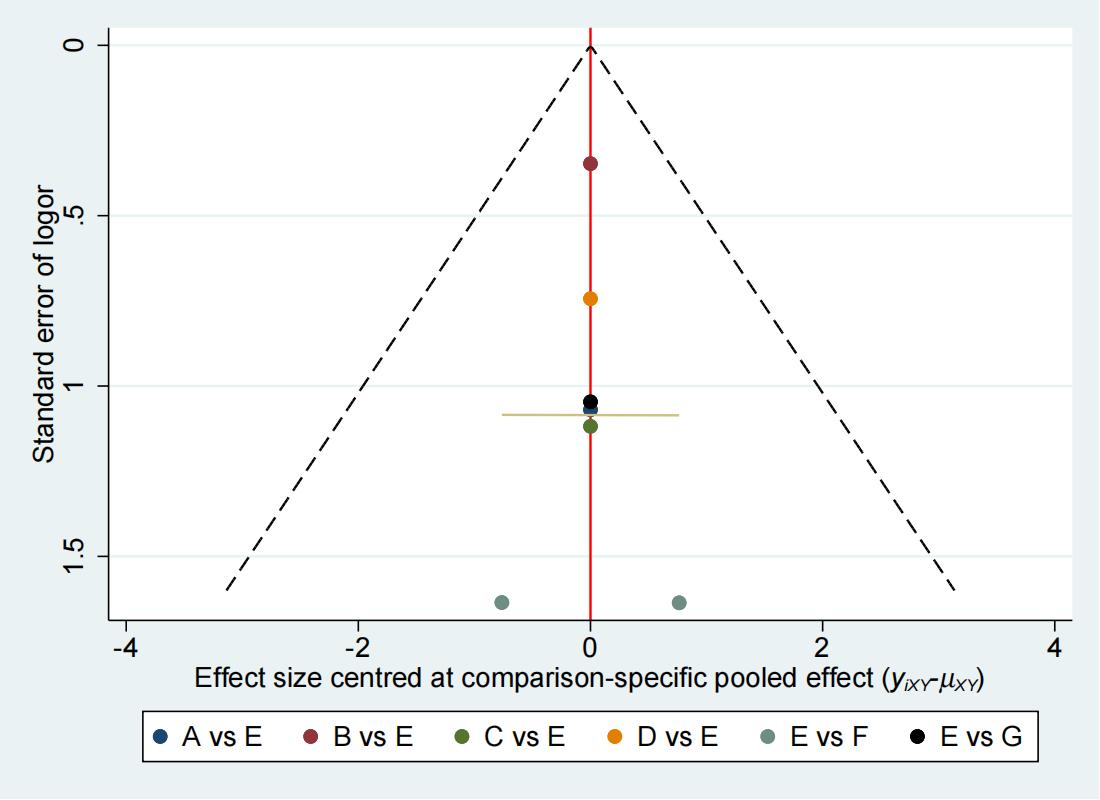


A: Treatment Canagliflozin, B: Treatment Dapagliflozin, C: Treatment Empagliflozin, D: Treatment Ertugliflozin, E: Treatment Placebo, F: Treatment Sotagliflozin, G: Treatment Tofogliflozin

## 14.9 Comparison adjusted funnel plot for fracture

A: Treatment Canagliflozin, B: Treatment Dapagliflozin, C: Treatment Empagliflozin, D: Treatment Ertugliflozin, E: Treatment Placebo, F: Treatment Sotagliflozin

14.10 Comparison adjusted funnel plot for severe hypoglycemia

A: Treatment Canagliflozin, B: Treatment Dapagliflozin, C: Treatment Empagliflozin, D: Treatment Ertugliflozin, E: Treatment Placebo

14.11 Comparison adjusted funnel plot for reproductive tract infections in male

A: Treatment Canagliflozin, B: Treatment Dapagliflozin, C: Treatment Empagliflozin, D: Treatment Ertugliflozin, E: Treatment Placebo

14.12 Comparison adjusted funnel plot for reproductive tract infections in female

A: Treatment Canagliflozin, B: Treatment Dapagliflozin, C: Treatment Empagliflozin, D: Treatment Ertugliflozin, E: Treatment Ipragliflozin, F: Treatment Placebo

# Appendix 15: Subgroup analysis

## 15.1 Subgroup according to different dose

### 15.1.1 Network plots for reproductive tract infections in different dose

### 15.1.1 Forest for reproductive tract infections in different dose

### 15.1.2 Network plots for pollakiuria in different dose

### 15.1.2 Forest for pollakiuria in different dose

### 15.1.3 Network plots for hypovolemia in different dose

### 15.1.3 Forest for hypovolemia in different dose

### 15.1.4 Network plots for renal impairment or failure in different dose

### 15.1.4 Forest for renal impairment or failure in different dose

### 15.1.5 Network plots for acute kidney injury in different dose

### 15.1.5 Forest for acute kidney injury in different dose

### 15.1.6 Network plots for urinary tract infections in different dose

### 15.1.6 Forest for urinary tract infections in different dose

### 15.1.7 Network plots for amputation in different dose

### 15.1.7 Forest for amputation in different dose

### 15.1.8 Network plots for diabetic ketoacidosis in different dose

### 15.1.8 Forest for diabetic ketoacidosis in different dose

### 15.1.9 Network plots for fracture in different dose

### 15.1.9 Forest for fracture in different dose

### 15.1.10 Network plots for severe hypoglycemia in different dose

### 15.1.10 Forest for severe hypoglycemia in different dose

## 15.2 Subgroup according to different regions

### 15.2.1 Forest for reproductive tract infections in Asia

### 15.2.2 Forest for reproductive tract infections in China

### 15.2.3 Forest for reproductive tract infections in Japan

### 15.2.4 Forest for pollakiuria in Asia

### 15.2.5 Forest for pollakiuria in Japan

### 15.2.6 Forest for hypovolemia in Asia

### 15.2.7 Forest for hypovolemia in Japan

### 15.2.8 Forest for renal impairment or failure in Asia

### 15.2.9 Forest for renal impairment or failure in Japan

### 15.2.10 Forest for urinary tract infections in Asia

### 15.2.11 Forest for urinary tract infections in China

### 15.2.12 Forest for urinary tract infections in Japan

### 15.2.13 Forest for fracture in Asia

### 15.2.14 Forest for fracture in Japan

## 15.3 Subgroup in patients with chronic kidney disease (CKD)

### 15.3.1 Network meta-analysis results for each outcome in patients with chronic kidney disease

| **Outcomes** | **Included SGLT-2i** | **Included study** | **Participants n (SGLT-2i/Placebo)** | **Events n(%) SGLT-2i/Placebo** | ***I^2^ (pairwise and consistency)%*** | **PSRF** | **Results** | **SUCRA** |
| --- | --- | --- | --- | --- | --- | --- | --- | --- |
| Reproductive tract infections | Ertugliflozin# Canagliflozin Empagliflozin Ipragliflozin# Sotagliflozin Dapagliflozin Luseogliflozin# | In total 12  2(eGFR 60-90 ) 3(eGFR 30-60) 1(eGFR 15-30) 1(eGFR 30-90) 1(eGFR＜59) 1(eGFR 45-59)  1(eGFR 25-75) 1(eGFR 25-60)  1(eGFR 30-50) | 13958/10961 | 530(3.80)/113(1.03) | 47.48/47.73 | 1.00 | Ertugliflozin,Canagliflozin was associated with an increased risk, other include SGLT-2i was not | Canagliflozin 0.77 Dapagliflozin 0.61 Sotagliflozin 0.54 Empagliflozin 0.53 Placebo 0.05 |
| Pollakiuria | Canagliflozin Luseogliflozin Ipragliflozin | In total 3 1(eGFR 30-50) 1(eGFR 30-60) 1(eGFR 30-90 ) | 393/186 | 28(7.12)/4(2.15) | 100/100 | 1.00 | all include SGLT-2i was not associated with an increased risk | Luseogliflozin 0.80 Canagliflozin 0.61 Ipragliflozin 0.45 Placebo 0.13 |
| Hypovolemia | Ertugliflozin# Canagliflozin Empagliflozin Sotagliflozin Dapagliflozin Luseogliflozin# | In total 11  3(eGFR 30-60) 1(eGFR 25-75)  1(eGFR 25-60) 1(eGFR 30-50) 1(eGFR 30-90) 1(eGFR＜59)  1(eGFR 45-59) 1(eGFR 25-75)  1(eGFR 25-60) 1(eGFR 30-50) | 13839/10915 | 718(5.19)/466(4.27) | 11.81/17.64 | 1.00 | Ertugliflozin incresed the risk, Luseogliflozin decreased the risk | Dapagliflozin 0.82 Canagliflozin 0.65 Sotagliflozin 0.51 Empagliflozin 0.29 Placebo 0.22 |
| Renal impairment or failure | Canagliflozin Dapagliflozin Sotagliflozin# | In total 5 (1 register)  2(eGFR 30-60) 1(eGFR 30-90)  1(eGFR 45-59) 1(eGFR 25-75) | 3200/2850 | 306(9.56)/302(10.6) | 0.00/0.00 | 1.01 | all include SGLT-2i was not associated with an increased risk | Sotagliflozin 0.92 Canagliflozin 0.44 Placebo 0.44 Dapagliflozin 0.19 |
| Acute kidney injury | Canagliflozin Dapagliflozin# Empagliflozin Sotagliflozin# | In total 4 (1 register)  2(eGFR 30-60) 1(eGFR 30-90) 1(eGFR＜59) | 7580/4874 | 379(5.00)/291(5.97) | 100/100 | 1.03 | Sotagliflozin incresed the risk, Dapagliflozin decreased the risk | Sotagliflozin 0.99 Placebo 0.57 Canagliflozin 0.49 Empagliflozin 0.43 Dapagliflozin 0.02 |
| Urinary tract infections | Ertugliflozin Canagliflozin Empagliflozin Ipragliflozin Sotagliflozin Dapagliflozin Luseogliflozin# | In total 13 (1 register)  4(eGFR 30-60) 2(eGFR 60-90)  1(eGFR 30-50) 1(eGFR 30-90) 1(eGFR＜59) 1(eGFR 45-59)  1(eGFR 25-75) 1(eGFR 25-60)  1(eGFR 30-50) | 14485/11221 | 1886(13.02)/1355(12.08) | 0.00/0.00 | 1.02 | all include SGLT-2i was not associated with an increased risk | Canagliflozin 0.79 Placebo 0.59 Empagliflozin 0.57 Sotagliflozin 0.57 Dapagliflozin 0.53 Ertugliflozin 0.37 Ipragliflozin 0.08 |
| Fracture | Sotaglifozin Canagliflozin Dapagliflozin Empagliflozin | In total 9 2(eGFR 30-60) 1(eGFR 25-75)  1(eGFR 25-60) 1(eGFR 45-59)  1(eGFR 15-30) 1(eGFR 60-90) 1(eGFR 30-90) 1(eGFR＜59) | 13779/10881 | 379(2.75)/295(2.71) | 87.00/87.19 | 1.00 | all include SGLT-2i was not associated with an increased risk | Dapagliflozin 0.88 Placebo 0.53 Canagliflozin 0.49 Empagliflozin 0.32 Sotaglifozin 0.27 |
| [Diabetic ketoacidosis](javascript:;) | Sotaglifozin Canagliflozin Dapagliflozin#Empagliflozin | In total 6 1(eGFR 25-75) 1(eGFR 25-60)  1(eGFR 45-59) 1(eGFR 15-30)  1(eGFR 60-90) 1(eGFR＜59) | 12665/10218 | 46(0.36)/16(0.16) | 0.00/0.00 | 1.00 | Dapagliflozin was associated with an increased risk, other included SGLT-2i was not | Canagliflozin 0.87 Empagliflozin 0.51 Sotaglifozin 0.46 Placebo 0.16 |
| Amputation | Sotagliflozin Dapagliflozin# | In total 4 (1 register)  1(eGFR 25-75) 1(eGFR 25-60)  1(eGFR 45-59) 1(eGFR 15-30) | 5780/5688 | 37(0.64)/33(0.58) | 38.88/35.66 | 1.06 | Dapagliflozin was associated with an increased risk, Sotagliflozin was not | Dapagliflozin 0.99 Placebo 0.35 Sotagliflozin 0.34 |
| Severe hypoglycemia | Sotaglifozin Dapagliflozin Empagliflozin | In total 6 2(eGFR 30-60) 1(eGFR＜59)  1(eGFR 15-30) 1(eGFR 25-75)  1(eGFR 25-60) | 11000/8204 | 138(1.25)/92(1.12) | 0.00/0.00 | 1.00 | all include SGLT-2i was not associated with an increased risk | Empagliflozin 0.89 Placebo 0.58 Sotaglifozin 0.44 Dapagliflozin 0.09 |

SGLT-2i: Sodium-glucose transporter 2 inhibitors, eGFR: estimated glomerular filtration rate, unit is mL/min/1.73 m2, PSRF: The potential scale reduction factor, SUCRA: surface under the cumulative ranking scores, #: Due to the wide confidence interval, studies related to this drug were not included in the network meta-analysis.

### 15.3.2 Forest for reproductive tract infection in patients with CKD

15.3.3 Forest for pollakiuria in patients with CKD

### 15.3.4 Forest for hypovolemia in patients with CKD

### 15.3.5 Forest for renal impairment or failure in patients with CKD

15.3.6 Forest for acute kidney injury in patients with CKD

### 15.3.7 Forest for urinary tract infections in patients with CKD

### 15.3.8 Forest for fracture in patients with CKD

### 15.3.9 Forest for amputation in patients with CKD

### 15.3.10 Forest for severe hypoglycemia in patients with CKD

### 15.3.11 Forest for diabetic ketoacidosis in patients with CKD

Appendix 16: Sensitivity analysis

## 16.1 According to the Interventions

### 16.1.1 Network meta-analysis results for each outcome in patients on metformin-based background therapy

| **Outcomes** | **Included SGLT-2i** | **Included study** | **Participants n (SGLT-2i/Placebo)** | **Events n(%) SGLT-2i/Placebo** | ***I^2^ (pairwise and consistency)%*** | **PSRF** | **SUCRA** |
| --- | --- | --- | --- | --- | --- | --- | --- |
| Reproductive tract infections | SGLT-2i | 20 (1 register) | 6146/2316 | 255(4.15)/29(1.25) | 53.58/53.23 | 1.00 | Placebo 0.18 |
|  | Canagliflozin | 3 | 663/268 | 33(4.98)/6(2.24) |  |  | 0.43 |
|  | Dapagliflozin | 4 | 1052/337 | 46(4.37)/8(2.37) |  |  | 0.65 |
|  | Empagliflozin | 4 | 1608/385 | 98(6.09)/4(1.04) |  |  | 0.83 |
|  | Ertugliflozin | 3 | 916/430 | 33(3.60)/4(0.93) |  |  | 0.60 |
|  | Ipragliflozin | 2 | 317/121 | 3(0.95)/1(0.83) |  |  | 0.40 |
|  | Remogliflozin | 1 | 465/146 | 7(1.51)/7(2.74) |  |  | 0.14 |
|  | Sotagliflozin# | 2 (1 register) | 655/219 | 33(5.04)/2(0.19) |  |  | NA |
|  | Tofogliflozin | 1 | 131/66 | 2(1.53)/0(0.00) |  |  | 0.77 |
| Pollakiuria | SGLT-2i | 8 (1 register) | 2140/763 | 47(2.20)/13(1.70) | 0.00/0.00 | 1.00 | Placebo 0.48 |
|  | Tofogliflozin# | 1 | 131/66 | 2(1.53)/0(0.00) |  |  | NA |
|  | Empagliflozin | 2 | 1147/178 | 26(2.27)/2(1.12) |  |  | 0.79 |
|  | Dapagliflozin | 1 (1 register) | 50/50 | 6(12.00)/0(0.00) |  |  | 0.03 |
|  | Ipragliflozin | 2 | 143/195 | 1(0.70)/10(5.13) |  |  | 0.70 |
|  | Canagliflozin# | 1 | 257/65 | 6(2.33)/1(1.54) |  |  | NA |
|  | Ertugliflozin# | 1 | 412/209 | 6(1.46)/0(0.00) |  |  | NA |
| Hypovolaemia | SGLT-2i | 9 | 3319/1204 | 20(0.60)/4(0.33) | 10.47/12.96 | 1.00 | Placebo 0.28 |
|  | Canagliflozin | 3 | 663/268 | 10(1.51)/1(0.37) |  |  | 0.77 |
|  | Dapagliflozin# | 2 | 598/246 | 0(0.00)/0(0.00) |  |  | NA |
|  | Empagliflozin | 2 | 1307/317 | 6(0.46)/1(0.32) |  |  | 0.59 |
|  | Ertugliflozin | 2 | 751/376 | 4(0.53)/2(0.53) |  |  | 0.36 |
| Renal impairment or failure | SGLT-2i | 4(1 register) | 988/571 | 15(1.52)/7(1.23) | 0.00/0.00 | 1.08 | Placebo 0.77 |
|  | Dapagliflozin | 2 | 598/246 | 15(2.51)/5(2.03) |  |  | 0.86 |
|  | Tofogliflozin | 1 | 131/66 | 0(0.00)/1(1.52) |  |  | 0.19 |
|  | Sotagliflozin | 1(1 register) | 259/259 | 0(0.00)/1(0.39) |  |  | 0.18 |
| Acute kidney injury | Sotaglifozin | 1(1 register) | 259/259 | 0(0.00)/1(0.39) | NA | NA | NA |
| Urinary tract infections | SGLT-2i | 23(3 register) | 6942/2656 | 360(5.19)/117(4.41) | 17.03/16.99 | 1.00 | Placebo 0.30 |
|  | Tofogliflozin | 1 | 131/66 | 3(2.29)/1(1.52) |  |  | 0.63 |
|  | Empagliflozin | 5(1 register) | 2914/699 | 192(6.59)/48(6.87) |  |  | 0.44 |
|  | Sotaglifozin | 2(1 register) | 438/319 | 19(4.34)/16(5.02) |  |  | 0.39 |
|  | Canagliflozin | 3 | 663/268 | 29(4.37)/9(3.36) |  |  | 0.53 |
|  | Ertugliflozin | 3 | 916/430 | 23(2.51)/10(2.33) |  |  | 0.37 |
|  | Ipragliflozin | 3 | 404/204 | 17(4.21)/7(3.43) |  |  | 0.48 |
|  | Dapagliflozin | 5(1 register) | 1011/524 | 54(5.34)/23(4.39) |  |  | 0.54 |
|  | Remogliflozin | 1 | 465/146 | 23(4.95)/3(2.05) |  |  | 0.82 |
| Fracture | SGLT-2i | 2(1 register) | 775/1304 | 6(0.77)/1(0.33) | 100/100 | 1.02 | Placebo 0.23 |
|  | Dapagliflozin | 1 | 299/145 | 2(0.67)/1(0.68) |  |  | 0.29 |
|  | Sotaglifozin | 1(1 register) | 476/159 | 4(0.84)/0(0.00) |  |  | 0.98 |

SGLT-2i: Sodium-glucose transporter 2 inhibitors, eGFR: estimated glomerular filtration rate, unit is mL/min/1.73 m2, PSRF: The potential scale reduction factor, SUCRA: surface under the cumulative ranking scores, #: Due to the wide confidence interval, studies related to this drug were not included in the network meta-analysis. NA: [Not Applicable](javascript:;)

#### 16.1.1.1Forest for reproductive tract infection in patients treated with metformin-based background therapy

#### 16.1.1.2 Forest for pollakiuria in patients treated with metformin-based background therapy

***16.1.1.3Forest for hypovolaemia in patients treated with metformin-based background therapy***

#### 16.1.1.4Forest for renal impairment or failure in patients treated with metformin-based background therapy

#### 16.1.1.5 Forest for urinary tract infections in patients treated with metformin-based background therapy

#### 16.1.1.6 Forest for fracture in patients treated with metformin-based background therapy

####

### 16.1.2 Network meta-analysis results for each outcome in drug-naive patients

| **Outcomes** | **Included SGLT-2i** | **Included study** | **Participants n (SGLT-2i/Placebo)** | **Events n(%) SGLT-2i/Placebo** | ***I^2^ (pairwise and consistency)%*** | **PSRF** | **SUCRA** |
| --- | --- | --- | --- | --- | --- | --- | --- |
| Reproductive tract infections | SGLT-2i | 17 | 3495/1526 | 165(4.72)/18(1.18) | 33.95/33.49 | 1.00 | Placebo 0.16 |
|  | Canagliflozin | 2 | 570/285 | 28(4.91)/5(1.75) |  |  | 0.47 |
|  | Dapagliflozin | 5 | 1052/337 | 53(4.96)/4(1.04) |  |  | 0.64 |
|  | Empagliflozin | 2 | 527/306 | 20(3.80)/1(0.33) |  |  | 0.87 |
|  | Ertugliflozin | 1 | 308/153 | 33(10.71)/5(3.27) |  |  | 0.54 |
|  | Ipragliflozin | 1 | 203/69 | 11(5.42)/1(1.45) |  |  | 0.62 |
|  | Remogliflozin# | 2 | 333/84 | 16(4.80)/0(0.00) |  |  | NA |
|  | Tofogliflozin# | 1 | 116/56 | 1(0.86)/0(0.00) |  |  | NA |
|  | Luseogliflozin | 3 | 369/190 | 3(0.81)/2(1.05) |  |  | 0.20 |
| Pollakiuria | SGLT-2i | 4 | 2140/763 | 32(3.47)/6(1.22) | 100.00/100.00 | 1.00 | Placebo 0.16 |
|  | Luseogliflozin | 1 | 79/79 | 3(3.80)/2(2.53) |  |  | 0.36 |
|  | Ipragliflozin | 1 | 144/60 | 12(8.33)/2(2.90) |  |  | 0.59 |
|  | Canagliflozin | 1 | 392/192 | 11(2.81)/1(0.52) |  |  | 0.77 |
|  | Ertugliflozin | 1 | 308/153 | 6(1.95)/1(0.65) |  |  | 0.62 |
| Hypovolaemia | SGLT-2i | 7 | 1352/673 | 22(1.63)/9(1.34) | 49.19/49.83 | 1.00 | Placebo 0.38 |
|  | Canagliflozin | 2 | 570/285 | 7(1.23)/1(0.35) |  |  | 0.79 |
|  | Dapagliflozin# | 1 | 68/68 | 1(1.47)/0(0.00) |  |  | NA |
|  | Tofogliflozin | 1 | 116/56 | 2(1.72)/1(1.79) |  |  | 0.45 |
|  | Ertugliflozin | 1 | 308/153 | 5(1.62)/6(3.92) |  |  | 0.15 |
|  | Luseogliflozin | 2 | 290/111 | 7(2.41)/1(0.90) |  |  | 0.73 |
| Renal impairment or failure | SGLT-2i | 2 | 340/211 | 10(2.94)/8(3.79) | 100.00/100.00 | 1.02 | Placebo 0.32 |
|  | Luseogliflozin | 1 | 79/79 | 6(7.59)/6(7.59) |  |  | 0.58 |
|  | Dapagliflozin | 1 | 261/132 | 4(1.53)/2(1.52) |  |  | 0.60 |
| Urinary tract infections | SGLT-2i | 16(1 register) | 3519/1555 | 188(5.34)/64(4.12) | 0.00/0.00 | 1.00 | Placebo 0.39 |
|  | Tofogliflozin# | 1 | 116/56 | 1(0.86)/0(0.00) |  |  | NA |
|  | Empagliflozin | 2 | 572/306 | 31(5.88)/16(5.23) |  |  | 0.50 |
|  | Sotaglifozin | 1(1 register) | 249/150 | 11(4.42)/7(4.67) |  |  | 0.41 |
|  | Canagliflozin | 2 | 570/285 | 26(4.56)/9(3.16) |  |  | 0.68 |
|  | Ertugliflozin | 1 | 308/153 | 17(5.52)/13(28.50) |  |  | 1.56 |
|  | Ipragliflozin | 2 | 347/138 | 21(6.05)/7(5.07) |  |  | 0.53 |
|  | Dapagliflozin | 5 | 1069/383 | 72(6.74)/12(3.13) |  |  | 0.84 |
|  | Remogliflozin# | 2 | 465/146 | 9(2.70)/0(0.00) |  |  | NA |

SGLT-2i: Sodium-glucose transporter 2 inhibitors, eGFR: estimated glomerular filtration rate, unit is mL/min/1.73 m2, PSRF: The potential scale reduction factor, SUCRA: surface under the cumulative ranking scores, #: Due to the wide confidence interval, studies related to this drug were not included in the network meta-analysis. NA: [Not Applicable](javascript:;)

#### 16.1.2.1 Forest for reproductive tract infection in drug-naive patients

#### 16.1.2.2 Forest for pollakiuria in drug-naive patients

#### 16.1.2.3 Forest for hypovolaemia in drug-naive patients

#### 16.1.2.4 Forest for renal impairment or failure in drug-naive patients

#### 16.1.2.5 Forest for urinary tract infections in drug-naive patients

## 16.2 According to the follow-up period

### 16.2.1 Network meta-analysis results according to the follow-up period

| **Outcomes** | **Included SGLT-2i** | **Included study** | **Participants n (SGLT-2i/Placebo)** | **Events n(%) SGLT-2i/Placebo** | **PSRF** | **SUCRA** |
| --- | --- | --- | --- | --- | --- | --- |
| Reproductive tract infections ***Short term*** | SGLT-2i | 10 | 3396/1812 | 214(6.30/25(1.38) | 1.00 | Placebo 0.03 |
|  | Canagliflozin | 1 | 477/237 | 42(8.81/2(0.84) |  | 0.73 |
|  | Dapagliflozin | 4 | 1207/710 | 101(8.37)/16(2.25) |  | 0.44 |
|  | Empagliflozin | 4 | 1403/712 | 49(3.49)/6(0.84) |  | 0.54 |
|  | Ertugliflozin | 1 | 309/153 | 22(7.12)/1(0.65) |  | 0.76 |
| Reproductive tract infections ***Long term*** | SGLT-2i | 10 | 3396/1812 | 298(8.78)/44(2.43) | 1.00 | Placebo 0.01 |
|  | Canagliflozin | 1 | 477/237 | 69(14.47)/12(5.06) |  | 0.45 |
|  | Dapagliflozin | 4 | 1207/710 | 126(10.44)/20(2.82) |  | 0.54 |
|  | Empagliflozin | 1 | 1403/712 | 76(5.42)/11(1.54) |  | 0.55 |
|  | Ertugliflozin | 1 | 309/153 | 27(8.74)/1(0.65) |  | 0.95 |
| Pollakiuria ***Short term*** | SGLT-2i | 2 | 659/330 | 23(3.49)/5(1.52) | 1.03 | Placebo 0.12 |
|  | Canagliflozin | 1 | 477/237 | 18(3.77)/5(2.11) |  | 0.39 |
|  | Empagliflozin | 1 | 182/93 | 5(2.75)/0(0.00) |  | 0.99 |
| Pollakiuria ***Long term*** | SGLT-2i | 2 | 659/330 | 57(8.65)/13(3.94) | 1.02 | Placebo 0.10 |
|  | Canagliflozin | 1 | 477/237 | 51(10.69)/13(25.49) |  | 0.40 |
|  | Empagliflozin | 1 | 182/93 | 6(3.30)/0(0.00) |  | 0.99 |
| Hypovolaemia ***Short term*** | SGLT-2i | 6 | 1903/1053 | 21(1.10)/5(0.47) | 1.00 | Placebo 0.36 |
|  | Canagliflozin | 1 | 477/237 | 8(1.68)/1(0.42) |  | 0.79 |
|  | Dapagliflozin | 3 | 935/573 | 11(1.18)/2(0.35) |  | 0.77 |
|  | Ertugliflozin | 1 | 309/153 | 1(0.32)/1(0.65) |  | 0.29 |
|  | Empagliflozin | 1 | 182/93 | 1(0.55)/1(1.08) |  | 0.29 |
| Hypovolaemia ***Long term*** | SGLT-2i | 10 | 3396/1812 | 60(1.77)/14(0.77) | 1.00 | Placebo 0.26 |
|  | Canagliflozin | 1 | 477/237 | 27(5.66)/4(1.69) |  | 0.81 |
|  | Dapagliflozin | 4 | 1207/710 | 18(1.49)/5(0.70) |  | 0.65 |
|  | Ertugliflozin | 1 | 309/153 | 1(0.32)/2(1.31) |  | 0.07 |
|  | Empagliflozin | 4 | 1403/712 | 14(1.00)/3(0.42) |  | 0.70 |
| Renal impairment or failure ***Short term*** | Dapagliflozin | 3 | 935/573 | 11(1.18)/7(1.22) | NA | NA |
| Renal impairment or failure ***Long term*** | Dapagliflozin | 4 | 1219/710 | 19(1.56)/10(1.41) | NA | NA |
| Acute kidney injury ***Short term*** | Dapagliflozin | 1 | 231/230 | 0(0.00)/1(0.43) | NA | NA |
| Acute kidney injury ***Long term*** | Dapagliflozin | 1 | 231/230 | 0(0.00)/2(0.87) | NA | NA |
| Urinary tract infections ***Short term*** | SGLT-2i | 10 | 3396/1812 | 261(7.69)/119(6.57) | 1.00 | Placebo 0.25 |
|  | Empagliflozin | 4 | 1403/712 | 125(8.91)/62(8.71) |  | 0.32 |
|  | Canagliflozin | 1 | 477/237 | 33(6.92)/12(5.06) |  | 0.63 |
|  | Ertugliflozin | 1 | 309/153 | 11(3.56)/3(1.96) |  | 0.76 |
|  | Dapagliflozin | 4 | 1207/710 | 92(7.62)/42(5.92) |  | 0.54 |
| Urinary tract infections ***Long term*** | SGLT-2i | 10 | 3396/1812 | 429(12.63)/194(10.71) | 1.00 | Placebo 0.38 |
|  | Empagliflozin | 4 | 1403/712 | 202(14.40)/112(15.73) |  | 0.23 |
|  | Canagliflozin | 1 | 477/237 | 74(15.51)/24(10.13) |  | 0.81 |
|  | Ertugliflozin | 1 | 309/153 | 16(5.18)/10(6.54) |  | 0.22 |
|  | Dapagliflozin | 4 | 1207/710 | 137(11.35)/48(6.76) |  | 0.86 |
| Fracture ***Short term*** | Empagliflozin | 2 | 515/258 | 7(1.36)/5(1.94) | NA | NA |
| Fracture ***Long term*** | Empagliflozin | 1 | 182/93 | 5(2.75)/2(2.15) | NA | NA |
| Severe hypoglycemia ***Short term*** | SGLT-2i | 3 | 1193/587 | 9(0.75)/10(1.70) | 1.02 | Placebo 0.75 |
|  | Canagliflozin | 1 | 477/237 | 4(0.84)/7(2.95) |  | 0.40 |
|  | Ertugliflozin | 1 | 309/153 | 0(0.00)/1(0.65) |  | 0.04 |
|  | Dapagliflozin | 1 | 408/197 | 5(1.23)/2(1.02) |  | 0.81 |
| Severe hypoglycemia ***Long term*** | SGLT-2i | 6 | 2414/1206 | 20(0.83)/12(1.00) | 1.00 | Placebo 0.56 |
|  | Empagliflozin | 3 | 1221/619 | 3(0.25)/1(0.16) |  | 0.76 |
|  | Ertugliflozin | 1 | 309/153 | 0(0.00)/1(0.65) |  | 0.03 |
|  | Canagliflozin | 1 | 477/237 | 11(2.31)/8(3.38) |  | 0.45 |
|  | Dapagliflozin | 1 | 408/197 | 6(1.47)/2(1.02) |  | 0.70 |

SGLT-2i: Sodium-glucose transporter 2 inhibitors, eGFR: estimated glomerular filtration rate, unit is mL/min/1.73 m2, PSRF: The potential scale reduction factor, SUCRA: surface under the cumulative ranking scores, NA: [Not Applicable](javascript:;)

### 16.2.2 Forest for reproductive tract infection in short term patients

16.2.3 Forest for reproductive tract infection in long term patients

### 16.2.4 Forest for pollakiuria in short term patients

### 16.2.5 Forest for pollakiuria in long term patients

16.2.6 Forest for hypovolemia in short term patients

### 16.2.7 Forest for hypovolemia in long term patients

### 16.2.8 Forest for urinary tract infections in short term patients

### 16.2.9 Forest for urinary tract infections in long term patients

### 16.2.10 Forest for severe hypoglycemia in short term patients

16.2.11 Forest for severe hypoglycemia in long term patients

# Appendix 17: CINeMA Assessment

## 17.1 Confidence in effect estimates for reproductive tract infections

| **Comparison** | **Number of studies** | **Within-study bias** | **Reporting bias** | **Indirectness** | **Imprecision** | **Heterogeneity** | **Incoherence** | **Confidence rating** | **Reason(s) for downgrading** |
| --- | --- | --- | --- | --- | --- | --- | --- | --- | --- |
| Canagliflozin:Placebo | 11 | No concerns | Low risk | No concerns | No concerns | No concerns | No concerns | High |  |
| Dapagliflozin:Empagliflozin | 2 | No concerns | Low risk | No concerns | Major concerns | No concerns | Major concerns | low | ["Imprecision","Incoherence"] |
| Dapagliflozin:Placebo | 23 | No concerns | Low risk | No concerns | No concerns | No concerns | No concerns | High |  |
| Dapagliflozin:Remogliflozin | 1 | No concerns | Low risk | No concerns | Major concerns | No concerns | No concerns | Low | ["Imprecision"] |
| Empagliflozin:Placebo | 17 | No concerns | Low risk | No concerns | No concerns | No concerns | Major concerns | Low | ["Incoherence"] |
| Empagliflozin:Sotagliflozin | 1 | No concerns | Low risk | No concerns | Major concerns | No concerns | No concerns | Low | ["Imprecision"] |
| Ertugliflozin:Placebo | 6 | Some concerns | Low risk | No concerns | No concerns | No concerns | No concerns | Moderate | ["Within-study bias"] |
| Ipragliflozin:Placebo | 8 | No concerns | Low risk | No concerns | Major concerns | No concerns | No concerns | Low | ["Imprecision"] |
| Luseogliflozin:Placebo | 4 | No concerns | Low risk | No concerns | Major concerns | No concerns | No concerns | Low | ["Imprecision"] |
| Placebo:Remogliflozin | 2 | No concerns | Low risk | No concerns | No concerns | No concerns | No concerns | High |  |
| Placebo:Sotagliflozin | 3 | No concerns | Low risk | No concerns | No concerns | No concerns | No concerns | High |  |
| Canagliflozin:Dapagliflozin | 0 | No concerns | Low risk | No concerns | No concerns | No concerns | No concerns | High |  |
| Canagliflozin:Empagliflozin | 0 | No concerns | Low risk | No concerns | Major concerns | No concerns | No concerns | Low | ["Imprecision"] |
| Canagliflozin:Ertugliflozin | 0 | Some concerns | Low risk | No concerns | Major concerns | No concerns | No concerns | Low | ["Within-study bias","Imprecision"] |
| Canagliflozin:Ipragliflozin | 0 | No concerns | Low risk | No concerns | Major concerns | No concerns | No concerns | Low | ["Imprecision"] |
| Canagliflozin:Luseogliflozin | 0 | No concerns | Low risk | No concerns | Major concerns | No concerns | No concerns | Low | ["Imprecision"] |
| Canagliflozin:Remogliflozin | 0 | No concerns | Low risk | No concerns | Major concerns | No concerns | No concerns | Low | ["Imprecision"] |
| Canagliflozin:Sotagliflozin | 0 | No concerns | Low risk | No concerns | Major concerns | No concerns | No concerns | Low | ["Imprecision"] |
| Dapagliflozin:Ertugliflozin | 0 | Some concerns | Low risk | No concerns | Major concerns | No concerns | No concerns | Low | ["Within-study bias","Imprecision"] |
| Dapagliflozin:Ipragliflozin | 0 | No concerns | Low risk | No concerns | No concerns | No concerns | No concerns | High |  |
| Dapagliflozin:Luseogliflozin | 0 | No concerns | Low risk | No concerns | Major concerns | No concerns | No concerns | Low | ["Imprecision"] |
| Dapagliflozin:Sotagliflozin | 0 | No concerns | Low risk | No concerns | Major concerns | No concerns | No concerns | Low | ["Imprecision"] |
| Empagliflozin:Ertugliflozin | 0 | Some concerns | Low risk | No concerns | Major concerns | No concerns | No concerns | Low | ["Within-study bias","Imprecision"] |
| Empagliflozin:Ipragliflozin | 0 | No concerns | Low risk | No concerns | Major concerns | No concerns | No concerns | Low | ["Imprecision"] |
| Empagliflozin:Luseogliflozin | 0 | No concerns | Low risk | No concerns | Major concerns | No concerns | No concerns | Low | ["Imprecision"] |
| Empagliflozin:Remogliflozin | 0 | No concerns | Low risk | No concerns | Major concerns | No concerns | No concerns | Low | ["Imprecision"] |
| Ertugliflozin:Ipragliflozin | 0 | No concerns | Low risk | No concerns | Major concerns | No concerns | No concerns | Low | ["Imprecision"] |
| Ertugliflozin:Luseogliflozin | 0 | No concerns | Low risk | No concerns | Major concerns | No concerns | No concerns | Low | ["Imprecision"] |
| Ertugliflozin:Remogliflozin | 0 | Some concerns | Low risk | No concerns | Major concerns | No concerns | No concerns | Low | ["Within-study bias","Imprecision"] |
| Ertugliflozin:Sotagliflozin | 0 | No concerns | Low risk | No concerns | Major concerns | No concerns | No concerns | Low | ["Imprecision"] |
| Ipragliflozin:Luseogliflozin | 0 | No concerns | Low risk | No concerns | Major concerns | No concerns | No concerns | Low | ["Imprecision"] |
| Ipragliflozin:Remogliflozin | 0 | No concerns | Low risk | No concerns | Major concerns | No concerns | No concerns | Low | ["Imprecision"] |
| Ipragliflozin:Sotagliflozin | 0 | No concerns | Low risk | No concerns | Major concerns | No concerns | No concerns | Low | ["Imprecision"] |
| Luseogliflozin:Remogliflozin | 0 | No concerns | Low risk | No concerns | Major concerns | No concerns | No concerns | Low | ["Imprecision"] |
| Luseogliflozin:Sotagliflozin | 0 | No concerns | Low risk | No concerns | Major concerns | No concerns | No concerns | Low | ["Imprecision"] |
| Remogliflozin:Sotagliflozin | 0 | No concerns | Low risk | No concerns | Major concerns | No concerns | No concerns | Low | ["Imprecision"] |

17.2 Confidence in effect estimates for pollakiuria

| **Comparison** | **Number of studies** | **Within-study bias** | **Reporting bias** | **Indirectness** | **Imprecision** | **Heterogeneity** | **Incoherence** | **Confidence rating** | **Reason(s) for downgrading** |
| --- | --- | --- | --- | --- | --- | --- | --- | --- | --- |
| Canagliflozin:Placebo | 5 | No concerns | Low risk | No concerns | Major concerns | No concerns | No concerns | Low | ["Imprecision"] |
| Dapagliflozin:Empagliflozin | 2 | Some concerns | Low risk | No concerns | Major concerns | No concerns | No concerns | Low | ["Within-study bias","Imprecision"] |
| Dapagliflozin:Placebo | 1 | Some concerns | Low risk | No concerns | No concerns | No concerns | No concerns | Moderate | ["Within-study bias"] |
| Empagliflozin:Placebo | 6 | No concerns | Low risk | No concerns | No concerns | No concerns | No concerns | High |  |
| Ertugliflozin:Placebo | 2 | Some concerns | Low risk | No concerns | Major concerns | No concerns | No concerns | Low | ["Within-study bias","Imprecision"] |
| Ipragliflozin:Placebo | 5 | No concerns | Low risk | No concerns | Major concerns | No concerns | No concerns | Low | ["Imprecision"] |
| Luseogliflozin:Placebo | 2 | No concerns | Low risk | No concerns | Major concerns | No concerns | No concerns | Low | ["Imprecision"] |
| Placebo:Sotagliflozin | 1 | No concerns | Low risk | No concerns | Major concerns | No concerns | No concerns | Low | ["Imprecision"] |
| Canagliflozin:Dapagliflozin | 0 | No concerns | Low risk | No concerns | Major concerns | No concerns | No concerns | Low | ["Imprecision"] |
| Canagliflozin:Empagliflozin | 0 | No concerns | Low risk | No concerns | Major concerns | No concerns | No concerns | Low | ["Imprecision"] |
| Canagliflozin:Ertugliflozin | 0 | No concerns | Low risk | No concerns | Major concerns | No concerns | No concerns | Low | ["Imprecision"] |
| Canagliflozin:Ipragliflozin | 0 | No concerns | Low risk | No concerns | Major concerns | No concerns | No concerns | Low | ["Imprecision"] |
| Canagliflozin:Luseogliflozin | 0 | No concerns | Low risk | No concerns | Major concerns | No concerns | No concerns | Low | ["Imprecision"] |
| Canagliflozin:Sotagliflozin | 0 | No concerns | Low risk | No concerns | Major concerns | No concerns | No concerns | Low | ["Imprecision"] |
| Dapagliflozin:Ertugliflozin | 0 | Some concerns | Low risk | No concerns | Major concerns | No concerns | No concerns | Low | ["Within-study bias","Imprecision"] |
| Dapagliflozin:Ipragliflozin | 0 | No concerns | Low risk | No concerns | Major concerns | No concerns | No concerns | Low | ["Imprecision"] |
| Dapagliflozin:Luseogliflozin | 0 | No concerns | Low risk | No concerns | Major concerns | No concerns | No concerns | Low | ["Imprecision"] |
| Dapagliflozin:Sotagliflozin | 0 | No concerns | Low risk | No concerns | Major concerns | No concerns | No concerns | Low | ["Imprecision"] |
| Empagliflozin:Ertugliflozin | 0 | Some concerns | Low risk | No concerns | Major concerns | No concerns | No concerns | Low | ["Within-study bias","Imprecision"] |
| Empagliflozin:Ipragliflozin | 0 | No concerns | Low risk | No concerns | Major concerns | No concerns | No concerns | Low | ["Imprecision"] |
| Empagliflozin:Luseogliflozin | 0 | No concerns | Low risk | No concerns | Major concerns | No concerns | No concerns | Low | ["Imprecision"] |
| Empagliflozin:Sotagliflozin | 0 | No concerns | Low risk | No concerns | Major concerns | No concerns | No concerns | Low | ["Imprecision"] |
| Ertugliflozin:Ipragliflozin | 0 | No concerns | Low risk | No concerns | Major concerns | No concerns | No concerns | Low | ["Imprecision"] |
| Ertugliflozin:Luseogliflozin | 0 | Some concerns | Low risk | No concerns | Major concerns | No concerns | No concerns | Low | ["Within-study bias","Imprecision"] |
| Ertugliflozin:Sotagliflozin | 0 | Some concerns | Low risk | No concerns | Major concerns | No concerns | No concerns | Low | ["Within-study bias","Imprecision"] |
| Ipragliflozin:Luseogliflozin | 0 | No concerns | Low risk | No concerns | Major concerns | No concerns | No concerns | Low | ["Imprecision"] |
| Ipragliflozin:Sotagliflozin | 0 | No concerns | Low risk | No concerns | Major concerns | No concerns | No concerns | Low | ["Imprecision"] |
| Luseogliflozin:Sotagliflozin | 0 | No concerns | Low risk | No concerns | Major concerns | No concerns | No concerns | Low | ["Imprecision"] |

## 17.3 Confidence in effect estimates for hypovolemia

| **Comparison** | **Number of studies** | **Within-study bias** | **Reporting bias** | **Indirectness** | **Imprecision** | **Heterogeneity** | **Incoherence** | **Confidence rating** | **Reason(s) for downgrading** |
| --- | --- | --- | --- | --- | --- | --- | --- | --- | --- |
| Canagliflozin:Placebo | 9 | No concerns | Low risk | No concerns | Major concerns | No concerns | No concerns | Low | ["Imprecision"] |
| Dapagliflozin:Empagliflozin | 1 | No concerns | Low risk | No concerns | Major concerns | No concerns | No concerns | Low | ["Imprecision"] |
| Dapagliflozin:Placebo | 11 | No concerns | Low risk | No concerns | Major concerns | No concerns | No concerns | Low | ["Imprecision"] |
| Empagliflozin:Placebo | 7 | No concerns | Low risk | No concerns | Major concerns | No concerns | No concerns | Low | ["Imprecision"] |
| Ertugliflozin:Placebo | 5 | Some concerns | Low risk | No concerns | Major concerns | No concerns | No concerns | Low | ["Within-study bias","Imprecision"] |
| Ipragliflozin:Placebo | 2 | No concerns | Low risk | No concerns | Major concerns | No concerns | No concerns | Low | ["Imprecision"] |
| Luseogliflozin:Placebo | 3 | No concerns | Low risk | No concerns | Major concerns | No concerns | No concerns | Low | ["Imprecision"] |
| Placebo:Tofogliflozin | 2 | Some concerns | Low risk | No concerns | Major concerns | No concerns | No concerns | Low | ["Within-study bias","Imprecision"] |
| Canagliflozin:Dapagliflozin | 0 | No concerns | Low risk | No concerns | Major concerns | No concerns | No concerns | Low | ["Imprecision"] |
| Canagliflozin:Empagliflozin | 0 | No concerns | Low risk | No concerns | Major concerns | No concerns | No concerns | Low | ["Imprecision"] |
| Canagliflozin:Ertugliflozin | 0 | Some concerns | Low risk | No concerns | Major concerns | No concerns | No concerns | Low | ["Within-study bias","Imprecision"] |
| Canagliflozin:Ipragliflozin | 0 | No concerns | Low risk | No concerns | Major concerns | No concerns | No concerns | Low | ["Imprecision"] |
| Canagliflozin:Luseogliflozin | 0 | No concerns | Low risk | No concerns | Major concerns | No concerns | No concerns | Low | ["Imprecision"] |
| Canagliflozin:Tofogliflozin | 0 | No concerns | Low risk | No concerns | Major concerns | No concerns | No concerns | Low | ["Imprecision"] |
| Dapagliflozin:Ertugliflozin | 0 | Some concerns | Low risk | No concerns | Major concerns | No concerns | No concerns | Low | ["Within-study bias","Imprecision"] |
| Dapagliflozin:Ipragliflozin | 0 | No concerns | Low risk | No concerns | Major concerns | No concerns | No concerns | Low | ["Imprecision"] |
| Dapagliflozin:Luseogliflozin | 0 | No concerns | Low risk | No concerns | Major concerns | No concerns | No concerns | Low | ["Imprecision"] |
| Dapagliflozin:Tofogliflozin | 0 | No concerns | Low risk | No concerns | Major concerns | No concerns | No concerns | Low | ["Imprecision"] |
| Empagliflozin:Ertugliflozin | 0 | Some concerns | Low risk | No concerns | Major concerns | No concerns | No concerns | Low | ["Within-study bias","Imprecision"] |
| Empagliflozin:Ipragliflozin | 0 | No concerns | Low risk | No concerns | Major concerns | No concerns | No concerns | Low | ["Imprecision"] |
| Empagliflozin:Luseogliflozin | 0 | No concerns | Low risk | No concerns | Major concerns | No concerns | No concerns | Low | ["Imprecision"] |
| Empagliflozin:Tofogliflozin | 0 | No concerns | Low risk | No concerns | Major concerns | No concerns | No concerns | Low | ["Imprecision"] |
| Ertugliflozin:Ipragliflozin | 0 | Some concerns | Low risk | No concerns | Major concerns | No concerns | No concerns | Low | ["Within-study bias","Imprecision"] |
| Ertugliflozin:Luseogliflozin | 0 | Some concerns | Low risk | No concerns | Major concerns | No concerns | No concerns | Low | ["Within-study bias","Imprecision"] |
| Ertugliflozin:Tofogliflozin | 0 | Some concerns | Low risk | No concerns | Major concerns | No concerns | No concerns | Low | ["Within-study bias","Imprecision"] |
| Ipragliflozin:Luseogliflozin | 0 | No concerns | Low risk | No concerns | Major concerns | No concerns | No concerns | Low | ["Imprecision"] |
| Ipragliflozin:Tofogliflozin | 0 | No concerns | Low risk | No concerns | Major concerns | No concerns | No concerns | Low | ["Imprecision"] |
| Luseogliflozin:Tofogliflozin | 0 | No concerns | Low risk | No concerns | Major concerns | No concerns | No concerns | Low | ["Imprecision"] |

17.4 Confidence in effect estimates for renal impairment or failure

| **Comparison** | **Number of studies** | **Within-study bias** | **Reporting bias** | **Indirectness** | **Imprecision** | **Heterogeneity** | **Incoherence** | **Confidence rating** | **Reason(s) for downgrading** |
| --- | --- | --- | --- | --- | --- | --- | --- | --- | --- |
| Canagliflozin:Placebo | 2 | No concerns | Low risk | No concerns | Major concerns | No concerns | Major concerns | Low | ["Imprecision","Incoherence"] |
| Dapagliflozin:Placebo | 16 | Some concerns | Low risk | No concerns | No concerns | No concerns | Major concerns | Low | ["Within-study bias","Incoherence"] |
| Luseogliflozin:Placebo | 1 | No concerns | Low risk | No concerns | Major concerns | No concerns | Major concerns | Low | ["Imprecision","Incoherence"] |
| Canagliflozin:Dapagliflozin | 0 | No concerns | Low risk | No concerns | Major concerns | No concerns | Major concerns | Low | ["Imprecision","Incoherence"] |
| Canagliflozin:Luseogliflozin | 0 | No concerns | Low risk | No concerns | Major concerns | No concerns | Major concerns | Low | ["Imprecision","Incoherence"] |
| Dapagliflozin:Luseogliflozin | 0 | No concerns | Low risk | No concerns | Major concerns | No concerns | Major concerns | Low | ["Imprecision","Incoherence"] |

17.5 Confidence in effect estimates for Acute kidney injury

| **Comparison** | **Number of studies** | **Within-study bias** | **Reporting bias** | **Indirectness** | **Imprecision** | **Heterogeneity** | **Incoherence** | **Confidence rating** | **Reason(s) for downgrading** |
| --- | --- | --- | --- | --- | --- | --- | --- | --- | --- |
| Canagliflozin:Placebo | 2 | No concerns | Low risk | No concerns | Major concerns | No concerns | Major concerns | Low | ["Imprecision","Incoherence"] |
| Dapagliflozin:Placebo | 3 | No concerns | Low risk | No concerns | Major concerns | No concerns | Major concerns | Low | ["Imprecision","Incoherence"] |
| Empagliflozin:Placebo | 1 | No concerns | Low risk | No concerns | Major concerns | No concerns | Major concerns | Low | ["Imprecision","Incoherence"] |
| Ertugliflozin:Placebo | 1 | Some concerns | Low risk | No concerns | Major concerns | No concerns | Major concerns | Very low | ["Within-study bias","Imprecision","Incoherence"] |
| Placebo:Sotagliflozin | 2 | No concerns | Low risk | No concerns | Major concerns | No concerns | Major concerns | Low | ["Imprecision","Incoherence"] |
| Canagliflozin:Dapagliflozin | 0 | No concerns | Low risk | No concerns | Major concerns | No concerns | Major concerns | Low | ["Imprecision","Incoherence"] |
| Canagliflozin:Empagliflozin | 0 | No concerns | Low risk | No concerns | Major concerns | No concerns | Major concerns | Low | ["Imprecision","Incoherence"] |
| Canagliflozin:Ertugliflozin | 0 | Some concerns | Low risk | No concerns | Major concerns | No concerns | Major concerns | Very low | ["Within-study bias","Imprecision","Incoherence"] |
| Canagliflozin:Sotagliflozin | 0 | No concerns | Low risk | No concerns | Major concerns | No concerns | Major concerns | Low | ["Imprecision","Incoherence"] |
| Dapagliflozin:Empagliflozin | 0 | No concerns | Low risk | No concerns | Major concerns | No concerns | Major concerns | Low | ["Imprecision","Incoherence"] |
| Dapagliflozin:Ertugliflozin | 0 | Some concerns | Low risk | No concerns | Major concerns | No concerns | Major concerns | Very low | ["Within-study bias","Imprecision","Incoherence"] |
| Dapagliflozin:Sotagliflozin | 0 | No concerns | Low risk | No concerns | Major concerns | No concerns | Major concerns | Low | ["Imprecision","Incoherence"] |
| Empagliflozin:Ertugliflozin | 0 | Some concerns | Low risk | No concerns | Major concerns | No concerns | Major concerns | Very low | ["Within-study bias","Imprecision","Incoherence"] |
| Empagliflozin:Sotagliflozin | 0 | No concerns | Low risk | No concerns | Major concerns | No concerns | Major concerns | Low | ["Imprecision","Incoherence"] |
| Ertugliflozin:Sotagliflozin | 0 | Some concerns | Low risk | No concerns | Major concerns | No concerns | Major concerns | Very low | ["Within-study bias","Imprecision","Incoherence"] |

17.6 Confidence in effect estimates for urinary tract infections

| **Comparison** | **Number of studies** | **Within-study bias** | **Reporting bias** | **Indirectness** | **Imprecision** | **Heterogeneity** | **Incoherence** | **Confidence rating** | **Reason(s) for downgrading** |
| --- | --- | --- | --- | --- | --- | --- | --- | --- | --- |
| Canagliflozin:Placebo | 12 | No concerns | Low risk | No concerns | Major concerns | No concerns | No concerns | Low | ["Imprecision"] |
| Dapagliflozin:Empagliflozin | 2 | No concerns | Low risk | No concerns | No concerns | No concerns | Major concerns | Low | ["Incoherence"] |
| Dapagliflozin:Ertugliflozin | 1 | Some concerns | Low risk | No concerns | Major concerns | No concerns | No concerns | Low | ["Within-study bias","Imprecision"] |
| Dapagliflozin:Placebo | 24 | No concerns | Low risk | No concerns | No concerns | No concerns | No concerns | High |  |
| Dapagliflozin:Remogliflozin | 1 | No concerns | Low risk | No concerns | No concerns | No concerns | No concerns | High |  |
| Empagliflozin:Placebo | 18 | No concerns | Low risk | No concerns | Major concerns | No concerns | Major concerns | Low | ["Imprecision","Incoherence"] |
| Empagliflozin:Sotagliflozin | 1 | No concerns | Low risk | No concerns | Major concerns | No concerns | No concerns | Low | ["Imprecision"] |
| Ertugliflozin:Placebo | 7 | Some concerns | Low risk | No concerns | No concerns | No concerns | No concerns | Moderate | ["Within-study bias"] |
| Ipragliflozin:Placebo | 11 | No concerns | Low risk | No concerns | Major concerns | No concerns | No concerns | Low | ["Imprecision"] |
| Placebo:Remogliflozin | 2 | No concerns | Low risk | No concerns | No concerns | No concerns | No concerns | High |  |
| Placebo:Sotagliflozin | 7 | No concerns | Low risk | No concerns | Major concerns | No concerns | No concerns | Low | ["Imprecision"] |
| Placebo:Tofogliflozin | 3 | Some concerns | Low risk | No concerns | Major concerns | No concerns | No concerns | Low | ["Within-study bias","Imprecision"] |
| Canagliflozin:Dapagliflozin | 0 | No concerns | Low risk | No concerns | Major concerns | No concerns | No concerns | Low | ["Imprecision"] |
| Canagliflozin:Empagliflozin | 0 | No concerns | Low risk | No concerns | Major concerns | No concerns | No concerns | Low | ["Imprecision"] |
| Canagliflozin:Ertugliflozin | 0 | No concerns | Low risk | No concerns | Major concerns | No concerns | No concerns | Low | ["Imprecision"] |
| Canagliflozin:Ipragliflozin | 0 | No concerns | Low risk | No concerns | Major concerns | No concerns | No concerns | Low | ["Imprecision"] |
| Canagliflozin:Remogliflozin | 0 | No concerns | Low risk | No concerns | No concerns | No concerns | No concerns | High |  |
| Canagliflozin:Sotagliflozin | 0 | No concerns | Low risk | No concerns | Major concerns | No concerns | No concerns | Low | ["Imprecision"] |
| Canagliflozin:Tofogliflozin | 0 | No concerns | Low risk | No concerns | Major concerns | No concerns | No concerns | Low | ["Imprecision"] |
| Dapagliflozin:Ipragliflozin | 0 | No concerns | Low risk | No concerns | Major concerns | No concerns | No concerns | Low | ["Imprecision"] |
| Dapagliflozin:Sotagliflozin | 0 | No concerns | Low risk | No concerns | Major concerns | No concerns | No concerns | Low | ["Imprecision"] |
| Dapagliflozin:Tofogliflozin | 0 | Some concerns | Low risk | No concerns | Major concerns | No concerns | No concerns | Low | ["Within-study bias","Imprecision"] |
| Empagliflozin:Ertugliflozin | 0 | No concerns | Low risk | No concerns | Major concerns | No concerns | No concerns | Low | ["Imprecision"] |
| Empagliflozin:Ipragliflozin | 0 | No concerns | Low risk | No concerns | Major concerns | No concerns | No concerns | Low | ["Imprecision"] |
| Empagliflozin:Remogliflozin | 0 | No concerns | Low risk | No concerns | No concerns | No concerns | No concerns | High |  |
| Empagliflozin:Tofogliflozin | 0 | No concerns | Low risk | No concerns | Major concerns | No concerns | No concerns | Low | ["Imprecision"] |
| Ertugliflozin:Ipragliflozin | 0 | No concerns | Low risk | No concerns | Major concerns | No concerns | No concerns | Low | ["Imprecision"] |
| Ertugliflozin:Remogliflozin | 0 | Some concerns | Low risk | No concerns | No concerns | No concerns | No concerns | Moderate | ["Within-study bias"] |
| Ertugliflozin:Sotagliflozin | 0 | No concerns | Low risk | No concerns | Major concerns | No concerns | No concerns | Low | ["Imprecision"] |
| Ertugliflozin:Tofogliflozin | 0 | Some concerns | Low risk | No concerns | Major concerns | No concerns | No concerns | Low | ["Within-study bias","Imprecision"] |
| Ipragliflozin:Remogliflozin | 0 | No concerns | Low risk | No concerns | No concerns | No concerns | No concerns | High |  |
| Ipragliflozin:Sotagliflozin | 0 | No concerns | Low risk | No concerns | Major concerns | No concerns | No concerns | Low | ["Imprecision"] |
| Ipragliflozin:Tofogliflozin | 0 | No concerns | Low risk | No concerns | Major concerns | No concerns | No concerns | Low | ["Imprecision"] |
| Remogliflozin:Sotagliflozin | 0 | No concerns | Low risk | No concerns | No concerns | No concerns | No concerns | High |  |
| Remogliflozin:Tofogliflozin | 0 | Some concerns | Low risk | No concerns | Major concerns | No concerns | No concerns | Low | ["Within-study bias","Imprecision"] |
| Sotagliflozin:Tofogliflozin | 0 | No concerns | Low risk | No concerns | Major concerns | No concerns | No concerns | Low | ["Imprecision"] |

17.7 Confidence in effect estimates for amputation

| **Comparison** | **Number of studies** | **Within-study bias** | **Reporting bias** | **Indirectness** | **Imprecision** | **Heterogeneity** | **Incoherence** | **Confidence rating** | **Reason(s) for downgrading** |
| --- | --- | --- | --- | --- | --- | --- | --- | --- | --- |
| Dapagliflozin:Placebo | 2 | No concerns | Low risk | No concerns | Major concerns | No concerns | Major concerns | Low | ["Imprecision","Incoherence"] |
| Ertugliflozin:Placebo | 1 | Some concerns | Low risk | No concerns | Major concerns | No concerns | Major concerns | Very low | ["Within-study bias","Imprecision","Incoherence"] |
| Placebo:Sotagliflozin | 1 | No concerns | Low risk | No concerns | Major concerns | No concerns | Major concerns | Low | ["Imprecision","Incoherence"] |
| Dapagliflozin:Ertugliflozin | 0 | Some concerns | Low risk | No concerns | Major concerns | No concerns | Major concerns | Very low | ["Within-study bias","Imprecision","Incoherence"] |
| Dapagliflozin:Sotagliflozin | 0 | No concerns | Low risk | No concerns | Major concerns | No concerns | Major concerns | Low | ["Imprecision","Incoherence"] |
| Ertugliflozin:Sotagliflozin | 0 | Some concerns | Low risk | No concerns | Major concerns | No concerns | Major concerns | Very low | ["Within-study bias","Imprecision","Incoherence"] |

17.8 Confidence in effect estimates for [diabetic ketoacidosis](javascript:;)

| **Comparison** | **Number of studies** | **Within-study bias** | **Reporting bias** | **Indirectness** | **Imprecision** | **Heterogeneity** | **Incoherence** | **Confidence rating** | **Reason(s) for downgrading** |
| --- | --- | --- | --- | --- | --- | --- | --- | --- | --- |
| Canagliflozin:Placebo | 1 | No concerns | Low risk | No concerns | Major concerns | No concerns | Major concerns | Low | ["Imprecision","Incoherence"] |
| Dapagliflozin:Placebo | 1 | No concerns | Low risk | No concerns | No concerns | Major concerns | Major concerns | Low | ["Heterogeneity","Incoherence"] |
| Empagliflozin:Placebo | 1 | No concerns | Low risk | No concerns | Major concerns | No concerns | Major concerns | Low | ["Imprecision","Incoherence"] |
| Ertugliflozin:Placebo | 1 | Some concerns | Low risk | No concerns | No concerns | Major concerns | Major concerns | Very low | ["Within-study bias","Heterogeneity","Incoherence"] |
| Placebo:Sotagliflozin | 2 | No concerns | Low risk | No concerns | Major concerns | No concerns | Major concerns | Low | ["Imprecision","Incoherence"] |
| Placebo:Tofogliflozin | 1 | No concerns | Low risk | No concerns | No concerns | Major concerns | Major concerns | Low | ["Heterogeneity","Incoherence"] |
| Canagliflozin:Dapagliflozin | 0 | No concerns | Low risk | No concerns | Major concerns | No concerns | Major concerns | Low | ["Imprecision","Incoherence"] |
| Canagliflozin:Empagliflozin | 0 | No concerns | Low risk | No concerns | Major concerns | No concerns | Major concerns | Low | ["Imprecision","Incoherence"] |
| Canagliflozin:Ertugliflozin | 0 | Some concerns | Low risk | No concerns | Major concerns | No concerns | Major concerns | Very low | ["Within-study bias","Imprecision","Incoherence"] |
| Canagliflozin:Sotagliflozin | 0 | No concerns | Low risk | No concerns | Major concerns | No concerns | Major concerns | Low | ["Imprecision","Incoherence"] |
| Canagliflozin:Tofogliflozin | 0 | No concerns | Low risk | No concerns | Major concerns | No concerns | Major concerns | Low | ["Imprecision","Incoherence"] |
| Dapagliflozin:Empagliflozin | 0 | No concerns | Low risk | No concerns | Major concerns | No concerns | Major concerns | Low | ["Imprecision","Incoherence"] |
| Dapagliflozin:Ertugliflozin | 0 | Some concerns | Low risk | No concerns | Major concerns | No concerns | Major concerns | Very low | ["Within-study bias","Imprecision","Incoherence"] |
| Dapagliflozin:Sotagliflozin | 0 | No concerns | Low risk | No concerns | Major concerns | No concerns | Major concerns | Low | ["Imprecision","Incoherence"] |
| Dapagliflozin:Tofogliflozin | 0 | No concerns | Low risk | No concerns | Major concerns | No concerns | Major concerns | Low | ["Imprecision","Incoherence"] |
| Empagliflozin:Ertugliflozin | 0 | Some concerns | Low risk | No concerns | Major concerns | No concerns | Major concerns | Very low | ["Within-study bias","Imprecision","Incoherence"] |
| Empagliflozin:Sotagliflozin | 0 | No concerns | Low risk | No concerns | Major concerns | No concerns | Major concerns | Low | ["Imprecision","Incoherence"] |
| Empagliflozin:Tofogliflozin | 0 | No concerns | Low risk | No concerns | Major concerns | No concerns | Major concerns | Low | ["Imprecision","Incoherence"] |
| Ertugliflozin:Sotagliflozin | 0 | Some concerns | Low risk | No concerns | Major concerns | No concerns | Major concerns | Very low | ["Within-study bias","Imprecision","Incoherence"] |
| Ertugliflozin:Tofogliflozin | 0 | Some concerns | Low risk | No concerns | Major concerns | No concerns | Major concerns | Very low | ["Within-study bias","Imprecision","Incoherence"] |
| Sotagliflozin:Tofogliflozin | 0 | No concerns | Low risk | No concerns | Major concerns | No concerns | Major concerns | Low | ["Imprecision","Incoherence"] |

17.9 Confidence in effect estimates for fracture

| **Comparison** | **Number of studies** | **Within-study bias** | **Reporting bias** | **Indirectness** | **Imprecision** | **Heterogeneity** | **Incoherence** | **Confidence rating** | **Reason(s) for downgrading** |
| --- | --- | --- | --- | --- | --- | --- | --- | --- | --- |
| Canagliflozin:Placebo | 5 | No concerns | Low risk | No concerns | No concerns | Major concerns | Major concerns | Low | ["Heterogeneity","Incoherence"] |
| Dapagliflozin:Placebo | 6 | No concerns | Low risk | No concerns | Major concerns | No concerns | Major concerns | Low | ["Imprecision","Incoherence"] |
| Empagliflozin:Placebo | 3 | No concerns | Low risk | No concerns | Major concerns | No concerns | Major concerns | Low | ["Imprecision","Incoherence"] |
| Ertugliflozin:Placebo | 1 | Some concerns | Low risk | No concerns | Major concerns | No concerns | Major concerns | Very low | ["Within-study bias","Imprecision","Incoherence"] |
| Placebo:Sotagliflozin | 5 | No concerns | Low risk | No concerns | Major concerns | No concerns | Major concerns | Low | ["Imprecision","Incoherence"] |
| Canagliflozin:Dapagliflozin | 0 | No concerns | Low risk | No concerns | Major concerns | No concerns | Major concerns | Low | ["Imprecision","Incoherence"] |
| Canagliflozin:Empagliflozin | 0 | No concerns | Low risk | No concerns | Major concerns | No concerns | Major concerns | Low | ["Imprecision","Incoherence"] |
| Canagliflozin:Ertugliflozin | 0 | Some concerns | Low risk | No concerns | Major concerns | No concerns | Major concerns | Very low | ["Within-study bias","Imprecision","Incoherence"] |
| Canagliflozin:Sotagliflozin | 0 | No concerns | Low risk | No concerns | Major concerns | No concerns | Major concerns | Low | ["Imprecision","Incoherence"] |
| Dapagliflozin:Empagliflozin | 0 | No concerns | Low risk | No concerns | Major concerns | No concerns | Major concerns | Low | ["Imprecision","Incoherence"] |
| Dapagliflozin:Ertugliflozin | 0 | Some concerns | Low risk | No concerns | Major concerns | No concerns | Major concerns | Very low | ["Within-study bias","Imprecision","Incoherence"] |
| Dapagliflozin:Sotagliflozin | 0 | No concerns | Low risk | No concerns | Major concerns | No concerns | Major concerns | Low | ["Imprecision","Incoherence"] |
| Empagliflozin:Ertugliflozin | 0 | Some concerns | Low risk | No concerns | Major concerns | No concerns | Major concerns | Very low | ["Within-study bias","Imprecision","Incoherence"] |
| Empagliflozin:Sotagliflozin | 0 | No concerns | Low risk | No concerns | Major concerns | No concerns | Major concerns | Low | ["Imprecision","Incoherence"] |
| Ertugliflozin:Sotagliflozin | 0 | Some concerns | Low risk | No concerns | Major concerns | No concerns | Major concerns | Very low | ["Within-study bias","Imprecision","Incoherence"] |

17.10 Confidence in effect estimates for severe hypoglycemia

| **Comparison** | **Number of studies** | **Within-study bias** | **Reporting bias** | **Indirectness** | **Imprecision** | **Heterogeneity** | **Incoherence** | **Confidence rating** | **Reason(s) for downgrading** |
| --- | --- | --- | --- | --- | --- | --- | --- | --- | --- |
| Canagliflozin:Placebo | 3 | No concerns | Low risk | No concerns | Major concerns | No concerns | No concerns | Low | ["Imprecision"] |
| Dapagliflozin:Empagliflozin | 1 | No concerns | Low risk | No concerns | Major concerns | No concerns | No concerns | Low | ["Imprecision"] |
| Dapagliflozin:Placebo | 2 | No concerns | Low risk | No concerns | Major concerns | No concerns | No concerns | Low | ["Imprecision"] |
| Empagliflozin:Placebo | 4 | No concerns | Low risk | No concerns | Major concerns | No concerns | No concerns | Low | ["Imprecision"] |
| Ertugliflozin:Placebo | 3 | Some concerns | Low risk | No concerns | Major concerns | No concerns | No concerns | Low | ["Within-study bias","Imprecision"] |
| Canagliflozin:Dapagliflozin | 0 | No concerns | Low risk | No concerns | Major concerns | No concerns | No concerns | Low | ["Imprecision"] |
| Canagliflozin:Empagliflozin | 0 | No concerns | Low risk | No concerns | Major concerns | No concerns | No concerns | Low | ["Imprecision"] |
| Canagliflozin:Ertugliflozin | 0 | No concerns | Low risk | No concerns | Major concerns | No concerns | No concerns | Low | ["Imprecision"] |
| Dapagliflozin:Ertugliflozin | 0 | Some concerns | Low risk | No concerns | Major concerns | No concerns | No concerns | Low | ["Within-study bias","Imprecision"] |
| Empagliflozin:Ertugliflozin | 0 | Some concerns | Low risk | No concerns | Major concerns | No concerns | No concerns | Low | ["Within-study bias","Imprecision"] |

17.11 Confidence in effect estimates for reproductive tract infections in male

| **Comparison** | **Number of studies** | **Within-study bias** | **Reporting bias** | **Indirectness** | **Imprecision** | **Heterogeneity** | **Incoherence** | **Confidence rating** | **Reason(s) for downgrading** |
| --- | --- | --- | --- | --- | --- | --- | --- | --- | --- |
| Canagliflozin:Placebo | 7 | No concerns | Low risk | No concerns | Major concerns | No concerns | Major concerns | Low | ["Imprecision","Incoherence"] |
| Dapagliflozin:Placebo | 6 | Some concerns | Low risk | No concerns | No concerns | No concerns | Major concerns | Low | ["Within-study bias","Incoherence"] |
| Empagliflozin:Placebo | 12 | No concerns | Low risk | No concerns | No concerns | No concerns | Major concerns | Low | ["Incoherence"] |
| Ertugliflozin:Placebo | 5 | Some concerns | Low risk | No concerns | No concerns | No concerns | Major concerns | Low | ["Within-study bias","Incoherence"] |
| Canagliflozin:Dapagliflozin | 0 | Some concerns | Low risk | No concerns | Major concerns | No concerns | Major concerns | Very low | ["Within-study bias","Imprecision","Incoherence"] |
| Canagliflozin:Empagliflozin | 0 | No concerns | Low risk | No concerns | Major concerns | No concerns | Major concerns | Low | ["Imprecision","Incoherence"] |
| Canagliflozin:Ertugliflozin | 0 | Some concerns | Low risk | No concerns | Major concerns | No concerns | Major concerns | Very low | ["Within-study bias","Imprecision","Incoherence"] |
| Dapagliflozin:Empagliflozin | 0 | No concerns | Low risk | No concerns | Major concerns | No concerns | Major concerns | Low | ["Imprecision","Incoherence"] |
| Dapagliflozin:Ertugliflozin | 0 | Some concerns | Low risk | No concerns | Major concerns | No concerns | Major concerns | Very low | ["Within-study bias","Imprecision","Incoherence"] |
| Empagliflozin:Ertugliflozin | 0 | Some concerns | Low risk | No concerns | Major concerns | No concerns | Major concerns | Very low | ["Within-study bias","Imprecision","Incoherence"] |

17.12 Confidence in effect estimates for reproductive tract infections in female

| **Comparison** | **Number of studies** | **Within-study bias** | **Reporting bias** | **Indirectness** | **Imprecision** | **Heterogeneity** | **Incoherence** | **Confidence rating** | **Reason(s) for downgrading** |
| --- | --- | --- | --- | --- | --- | --- | --- | --- | --- |
| Canagliflozin:Placebo | 7 | No concerns | Low risk | No concerns | No concerns | No concerns | Major concerns | Low | ["Incoherence"] |
| Dapagliflozin:Placebo | 7 | Some concerns | Low risk | No concerns | No concerns | No concerns | Major concerns | Low | ["Within-study bias","Incoherence"] |
| Empagliflozin:Placebo | 12 | No concerns | Low risk | No concerns | No concerns | No concerns | Major concerns | Low | ["Incoherence"] |
| Ertugliflozin:Placebo | 5 | Some concerns | Low risk | No concerns | No concerns | No concerns | Major concerns | Low | ["Within-study bias","Incoherence"] |
| Ipragliflozin:Placebo | 1 | No concerns | Low risk | No concerns | Major concerns | No concerns | Major concerns | Low | ["Imprecision","Incoherence"] |
| Canagliflozin:Dapagliflozin | 0 | Some concerns | Low risk | No concerns | Major concerns | No concerns | Major concerns | Very low | ["Within-study bias","Imprecision","Incoherence"] |
| Canagliflozin:Empagliflozin | 0 | No concerns | Low risk | No concerns | Major concerns | No concerns | Major concerns | Low | ["Imprecision","Incoherence"] |
| Canagliflozin:Ertugliflozin | 0 | Some concerns | Low risk | No concerns | Major concerns | No concerns | Major concerns | Very low | ["Within-study bias","Imprecision","Incoherence"] |
| Canagliflozin:Ipragliflozin | 0 | No concerns | Low risk | No concerns | Major concerns | No concerns | Major concerns | Low | ["Imprecision","Incoherence"] |
| Dapagliflozin:Empagliflozin | 0 | No concerns | Low risk | No concerns | Major concerns | No concerns | Major concerns | Low | ["Imprecision","Incoherence"] |
| Dapagliflozin:Ertugliflozin | 0 | Some concerns | Low risk | No concerns | Major concerns | No concerns | Major concerns | Very low | ["Within-study bias","Imprecision","Incoherence"] |
| Dapagliflozin:Ipragliflozin | 0 | No concerns | Low risk | No concerns | No concerns | No concerns | Major concerns | Low | ["Incoherence"] |
| Empagliflozin:Ertugliflozin | 0 | Some concerns | Low risk | No concerns | Major concerns | No concerns | Major concerns | Very low | ["Within-study bias","Imprecision","Incoherence"] |
| Empagliflozin:Ipragliflozin | 0 | No concerns | Low risk | No concerns | No concerns | No concerns | Major concerns | Low | ["Incoherence"] |
| Ertugliflozin:Ipragliflozin | 0 | Some concerns | Low risk | No concerns | No concerns | Major concerns | Major concerns | Very low | ["Within-study bias","Heterogeneity","Incoherence"] |
